# Supplementary material for: An evaluation of Roluperidone as a promising repurposing candidate for Alzheimer’s Disease: A Computational Investigation
Source: PLoS One. 2025 Dec 17;20(12):e0338211. doi: 10.1371/journal.pone.0338211 (PMC12711050; doi:10.1371/journal.pone.0338211)
Supplement: S3 File — (DOCX) [file pone.0338211.s003.docx]

import pandas as pd

import numpy as np

data=pd.read_csv("//content//desc_bioassay.csv")

data.head()

X_data=data.drop(["Name","IC50(micromolar)","Status"],axis=1)

# Replace infinite values with NaN

X_data.replace([np.inf, -np.inf], np.nan, inplace=True)

X_data.round(decimals=3)

from sklearn.impute import SimpleImputer

imputer = SimpleImputer(strategy='median')  # strategy='mean',"median"

X_data = imputer.fit_transform(X_data)

# Convert the NumPy array back to a Pandas DataFrame

# Get the column names before imputation

original_columns = data.drop(["Name","IC50(micromolar)","Status"],axis=1).columns

#Adjust the columns for the DataFrame

X_data = pd.DataFrame(X_data, columns=original_columns)  # Keep original column names, adjusted to match X_data shape

X_data.head()

y_data=data['Status']

# Classify y_data based on 0 and 1

#y_data = y_data.apply(lambda x: 'active' if x == 1 else 'inactive')

#y_data = y_data.iloc[:len(X_data)]  # Update y_data_classified to match X_data

from sklearn.model_selection import KFold

# Initialize KFold cross-validation (5 splits)

cv = KFold(n_splits=5, shuffle=True, random_state=500)

cnt = 1

cv_scores = []

#from sklearn.feature_selection import SelectKBest, f_classif

# Feature selection using SelectFpr

from sklearn.feature_selection import SelectFpr, f_classif

p_value_threshold = 0.05

selector = SelectFpr(score_func=f_classif, alpha=p_value_threshold)

#selector = SelectKBest(score_func=f_classif,k=250)  # Select top 15 features

from sklearn.model_selection import train_test_split

import pandas as pd # Import pandas

# Split data into training (80%) and temporary set (20%) using stratification

X_train, X_temp, y_train, y_temp = train_test_split(

X_data, y_data, test_size=0.10, random_state=500, stratify=y_data, shuffle=True

)

# Split temporary set into test (10%) and external (10%) sets using stratification

X_test, X_external, y_test, y_external = train_test_split(

X_temp, y_temp, test_size=0.5, random_state=42, stratify=y_temp, shuffle=True

)

from sklearn.svm import SVC

from sklearn.metrics import accuracy_score, precision_score, recall_score, f1_score, roc_curve, roc_auc_score, confusion_matrix, ConfusionMatrixDisplay

import matplotlib.pyplot as plt

from sklearn.svm import SVC

# Instantiate a Support Vector Classifier (SVC)

svc_model = SVC(random_state=500, probability=True)

from sklearn.model_selection import GridSearchCV

# Define the parameter grid for the SVC model

param_grid_svc = {

    'C': [1, 10, 100, 500, 1000],  # Regularization parameter

    'gamma': ['scale', 'auto'], # Kernel coefficient

    'kernel': ['linear', 'rbf']  # Kernel type

}

# Instantiate GridSearchCV with the SVC model and parameter grid

grid_search_svc = GridSearchCV(estimator=svc_model, param_grid=param_grid_svc, cv=cv, scoring='accuracy', n_jobs=-1)

# Fit the grid search to the scaled training data

grid_search_svc.fit(X_train_scaled, y_train)

# Access the best model from the fitted GridSearchCV

best_svc_model = grid_search_svc.best_estimator_
