# Supplementary figures and images for: An evaluation of Roluperidone as a promising repurposing candidate for Alzheimer’s Disease: A Computational Investigation (part 1 of 2)
Source: PLoS One. 2025 Dec 17;20(12):e0338211. doi: 10.1371/journal.pone.0338211 (PMC12711050; doi:10.1371/journal.pone.0338211)

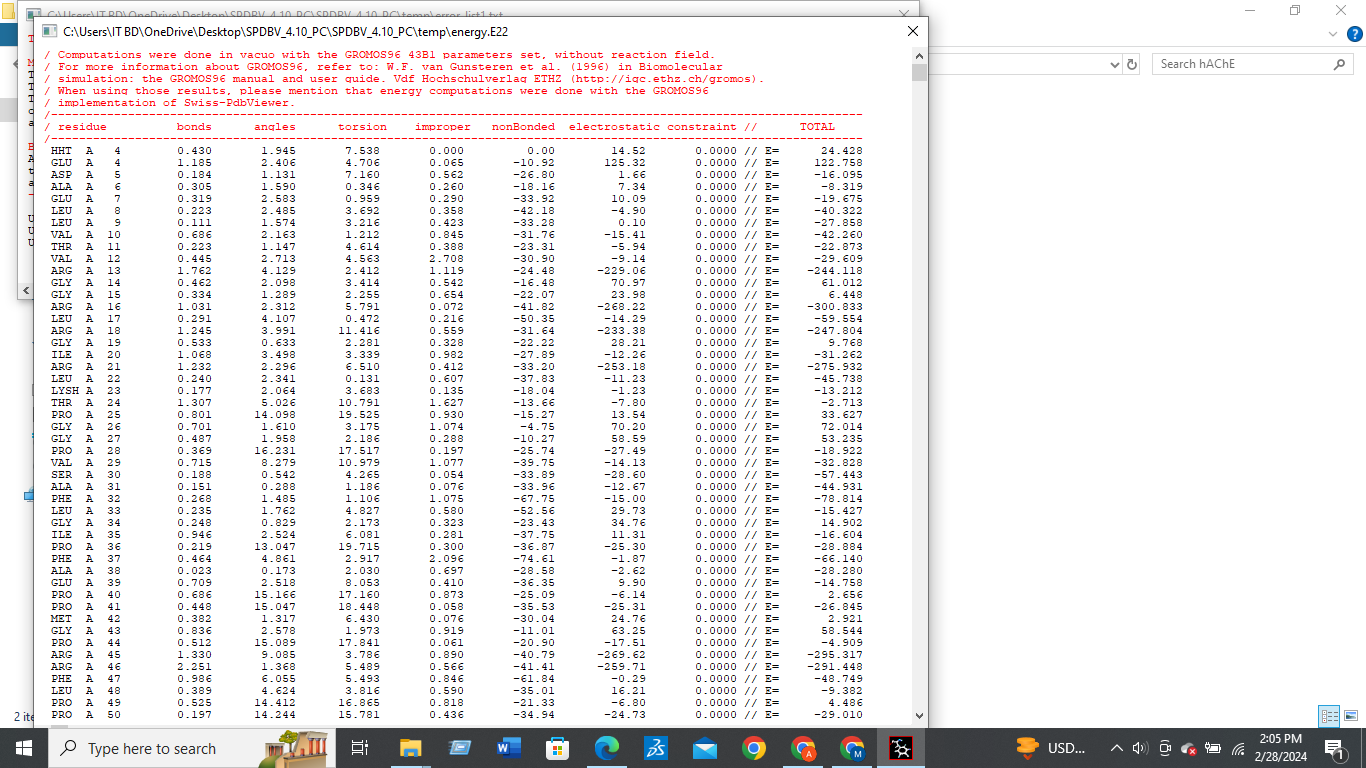

Supplement: S6 File — (ZIP) [file pone.0338211.s006.zip › S5.Binding Affinity/19.Roluperidone-12/hAChE/hAChE optimized data/Screenshot (304).png]

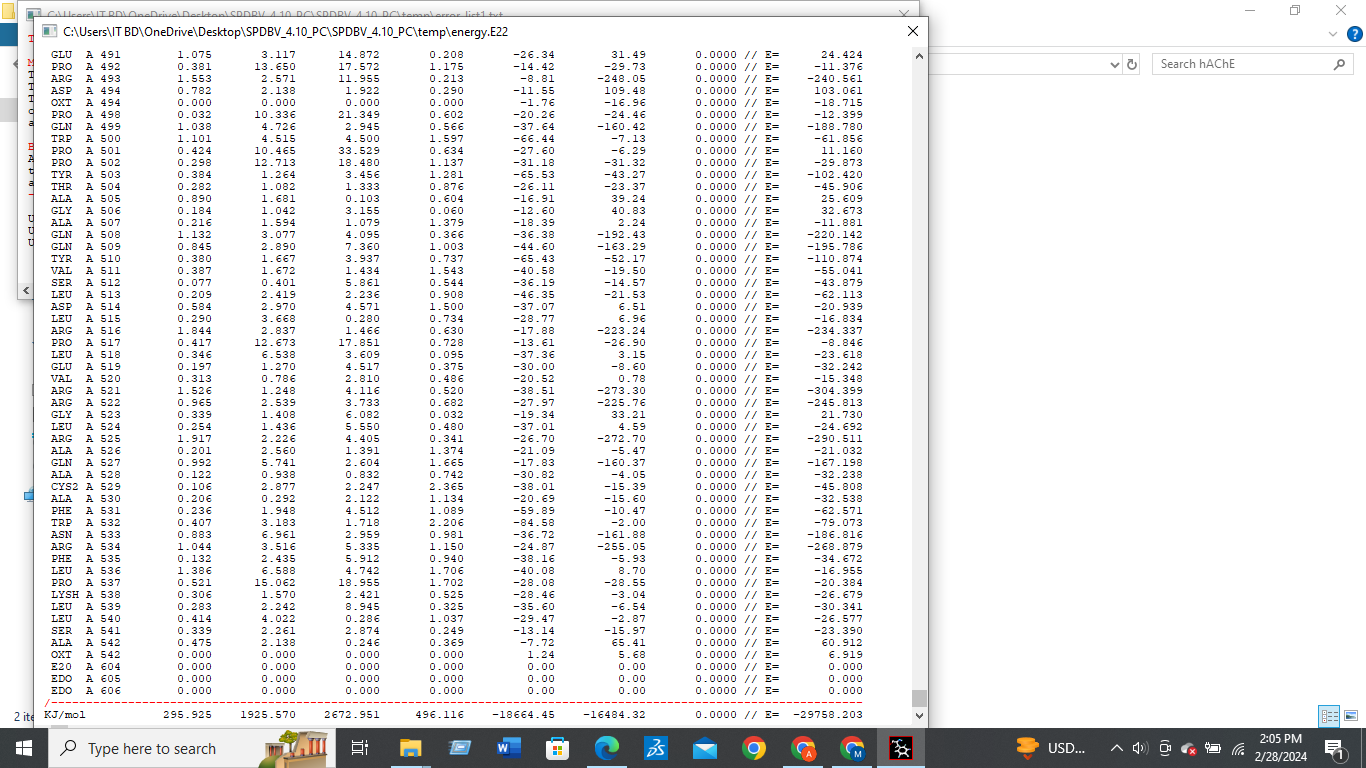

Supplement: S6 File — (ZIP) [file pone.0338211.s006.zip › S5.Binding Affinity/19.Roluperidone-12/hAChE/hAChE optimized data/Screenshot (305).png]

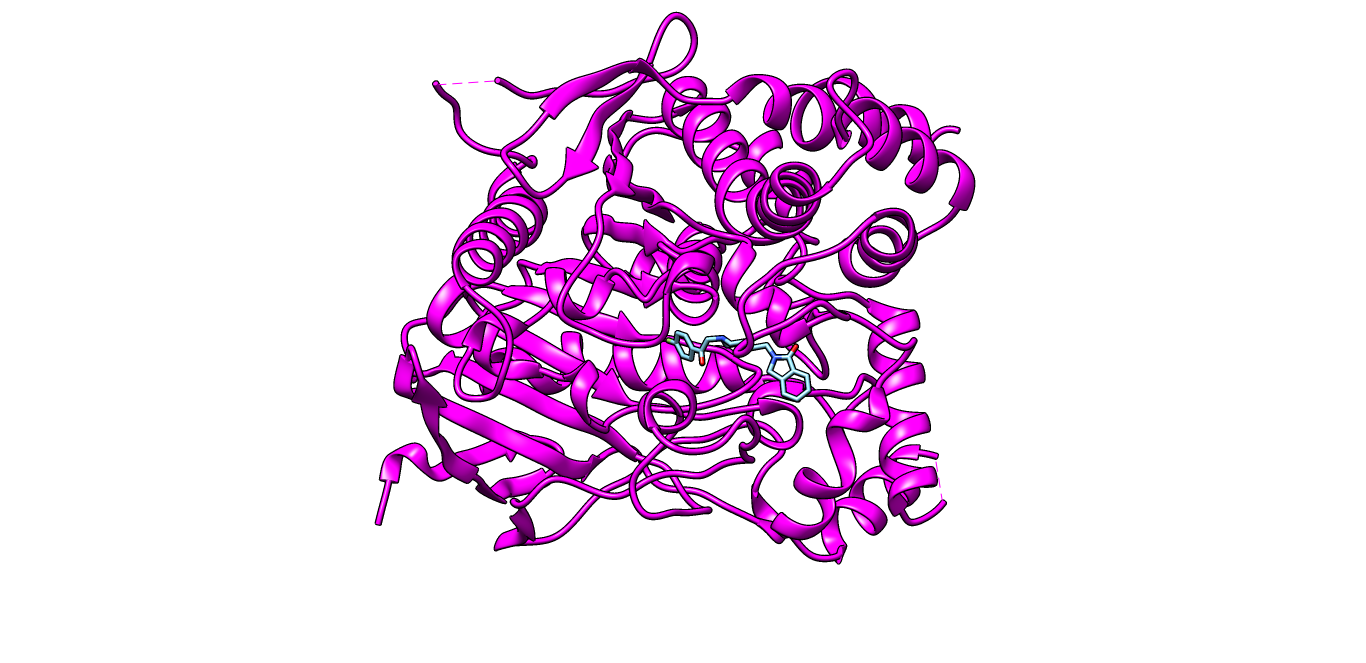

Supplement: S6 File — (ZIP) [file pone.0338211.s006.zip › S5.Binding Affinity/19.Roluperidone-12/roluperidone-hAChE interaction figure/ach-roluperidone.png]

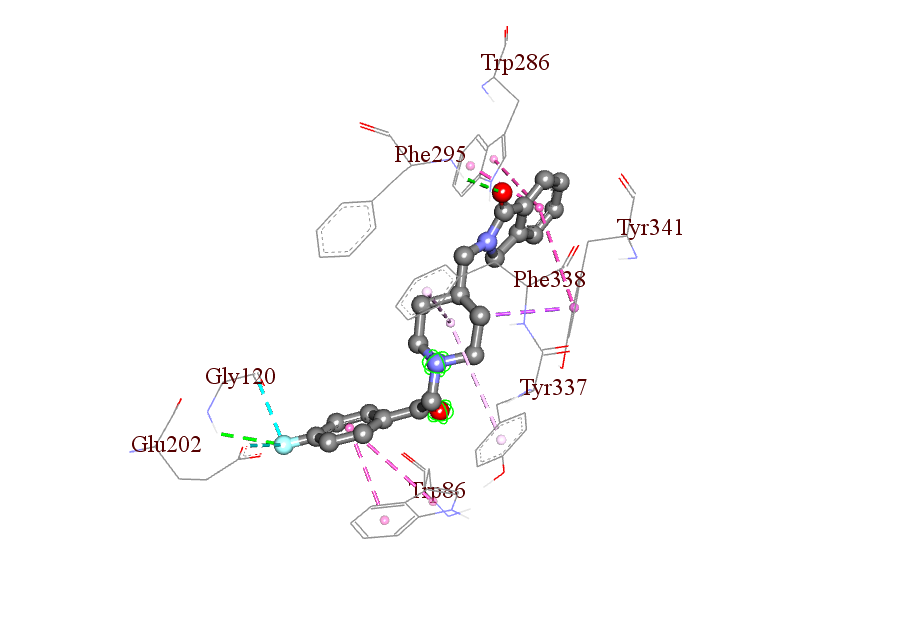

Supplement: S6 File — (ZIP) [file pone.0338211.s006.zip › S5.Binding Affinity/19.Roluperidone-12/roluperidone-hAChE interaction figure/rolup-hAChE.png]

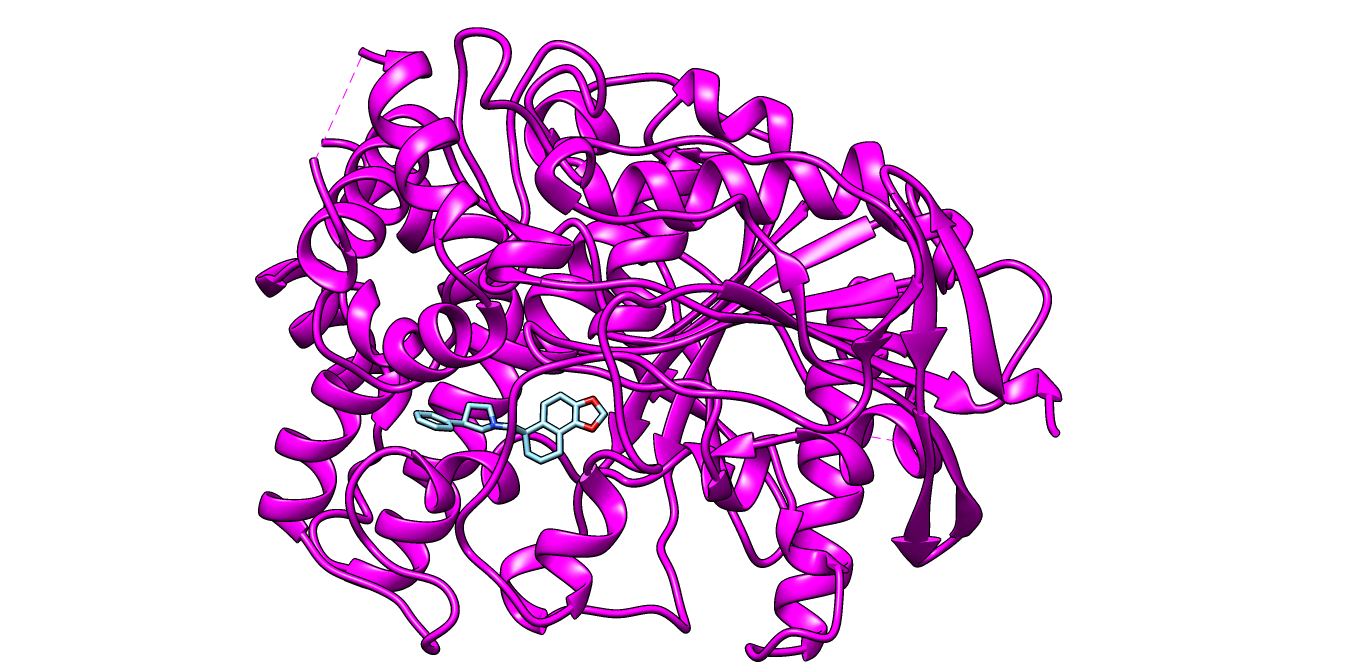

Supplement: S6 File — (ZIP) [file pone.0338211.s006.zip › S5.Binding Affinity/3.Napitane-11.9/napitane-hAChE interaction figure/ach-napitane.png]

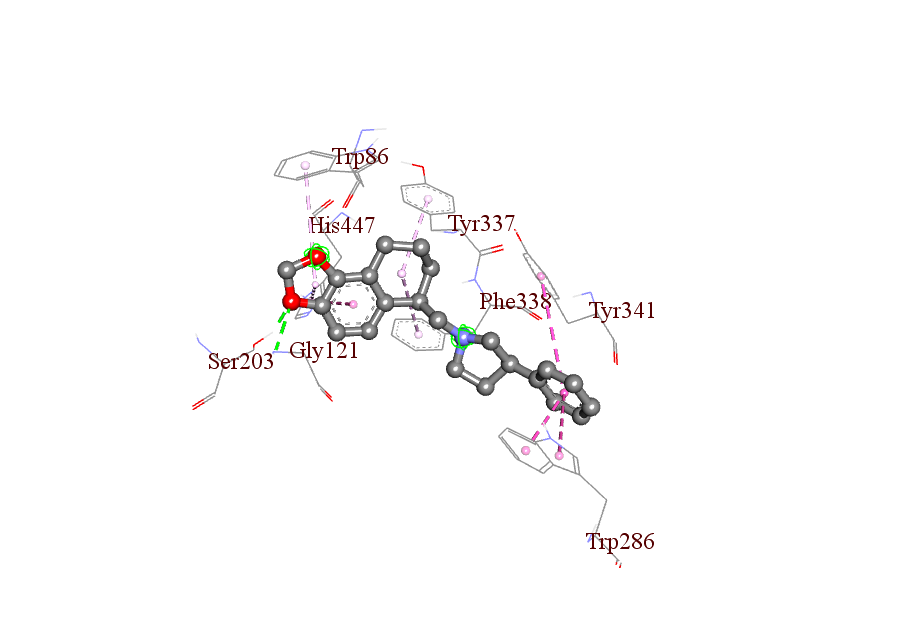

Supplement: S6 File — (ZIP) [file pone.0338211.s006.zip › S5.Binding Affinity/3.Napitane-11.9/napitane-hAChE interaction figure/napitine-ach-1.png]

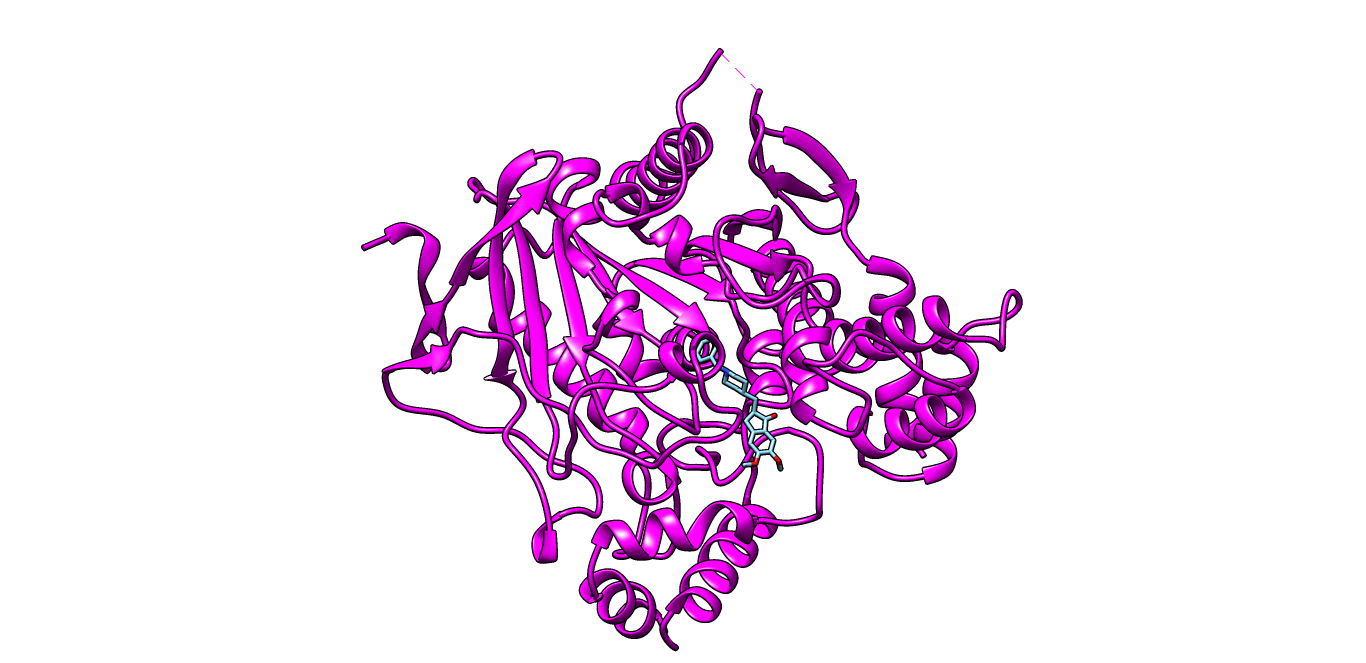

Supplement: S6 File — (ZIP) [file pone.0338211.s006.zip › S5.Binding Affinity/8.donepezil-11.8/interaction plot/ach-donipezel.png]

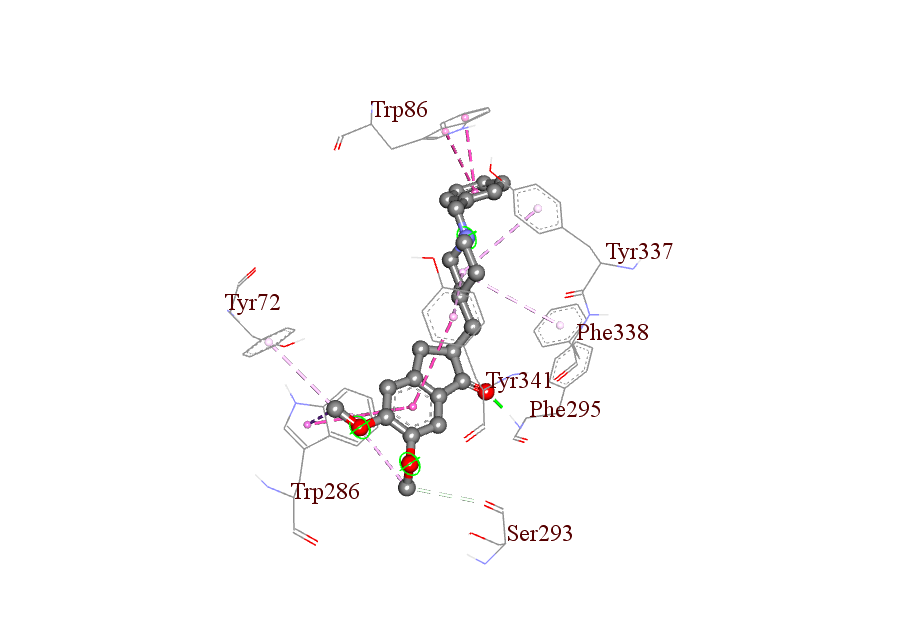

Supplement: S6 File — (ZIP) [file pone.0338211.s006.zip › S5.Binding Affinity/8.donepezil-11.8/interaction plot/done-hAchE.png]

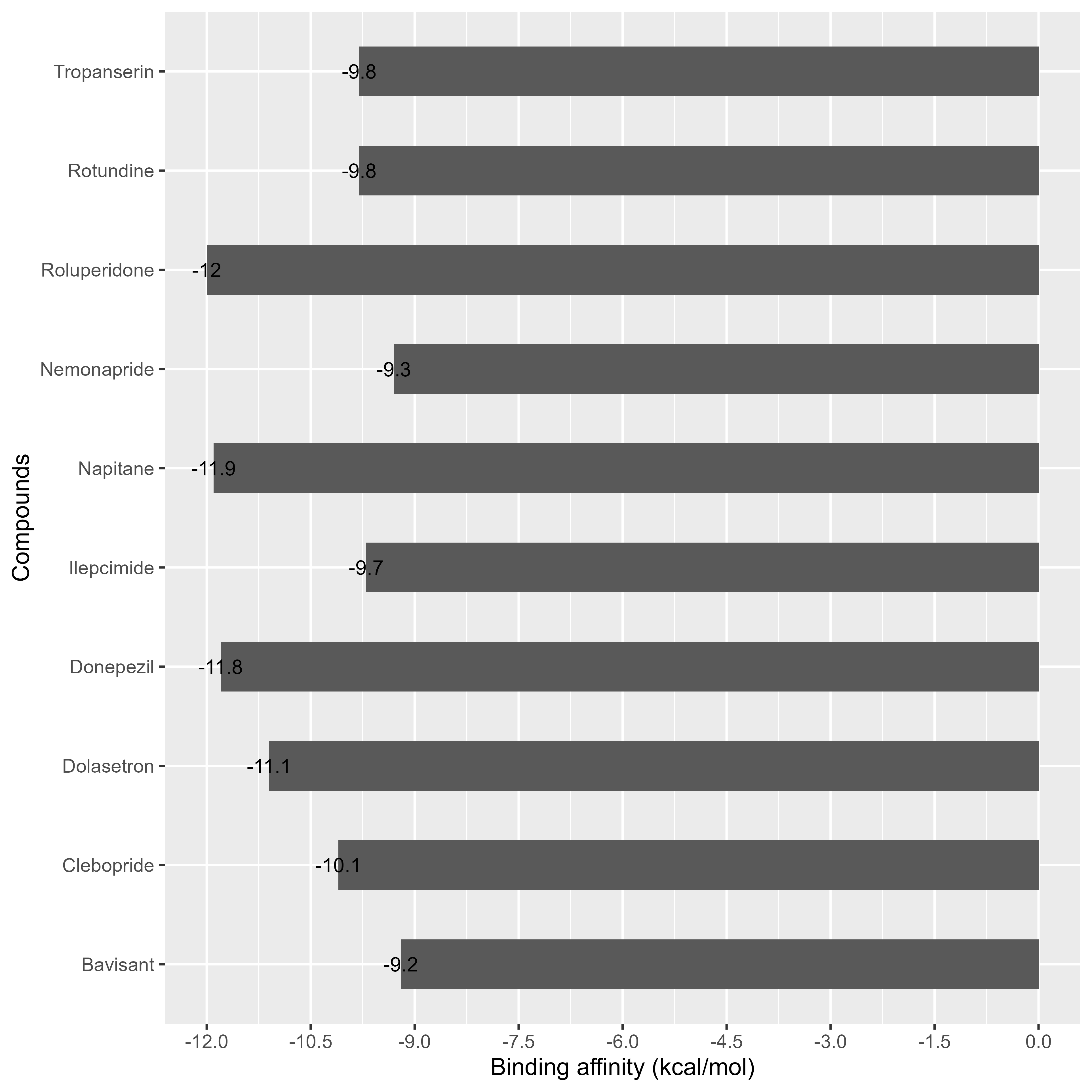

Supplement: S6 File — (ZIP) [file pone.0338211.s006.zip › S5.Binding Affinity/Molecular Docking/docking_scores.tiff]

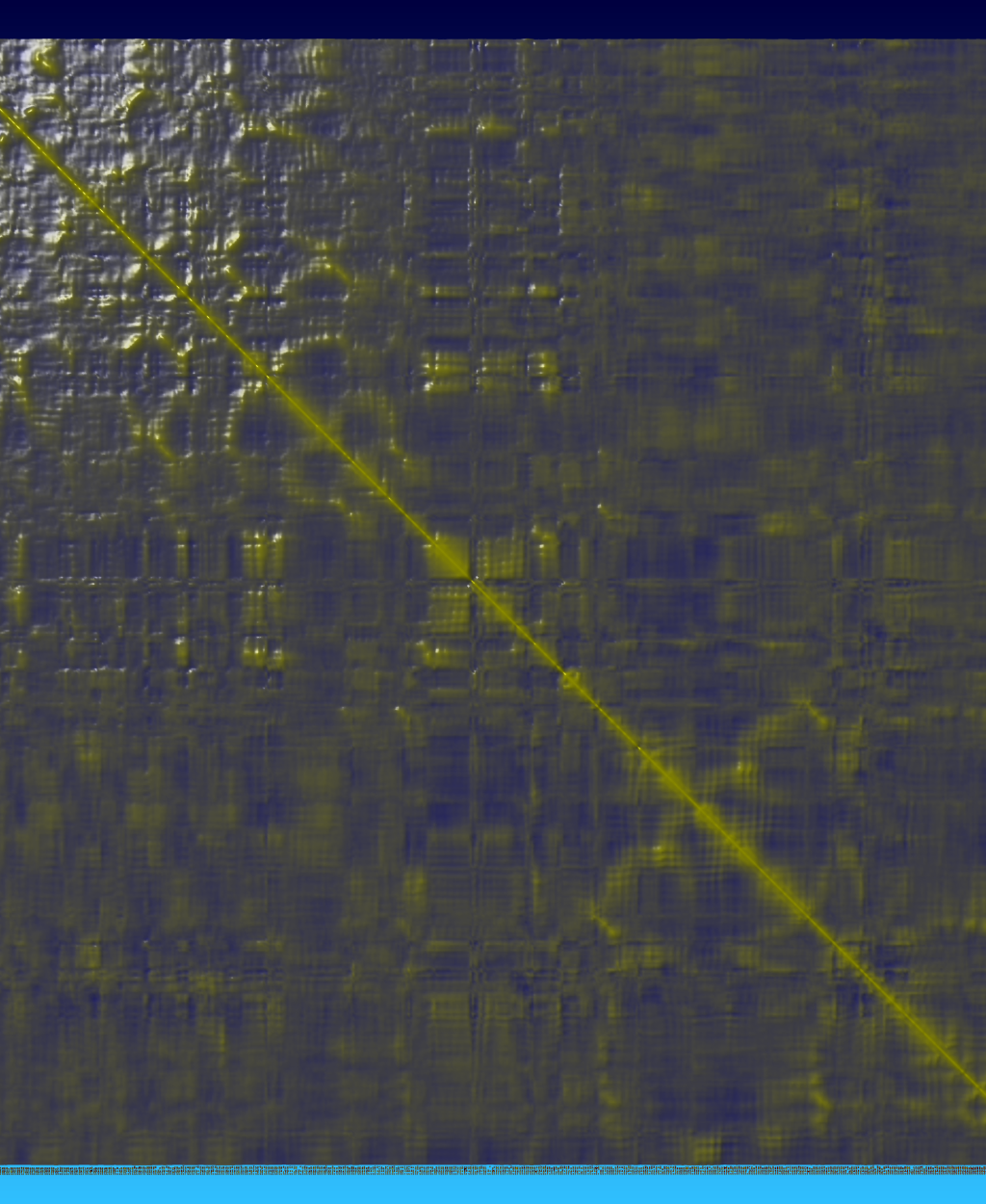

Supplement: S7 File — (ZIP) [file pone.0338211.s007.zip › S6.Molecular Dynamic Simulation/S6.Molecular Dynamic Simulation/MDS result for Donepezil_hAChE/Donepezil & hAChE - Al amin Afendy_dccm1.png]

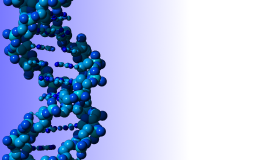

Supplement: S7 File — (ZIP) [file pone.0338211.s007.zip › S6.Molecular Dynamic Simulation/S6.Molecular Dynamic Simulation/MDS result for Donepezil_hAChE/Donepezil & hAChE - Al amin Afendy_report_background.png]

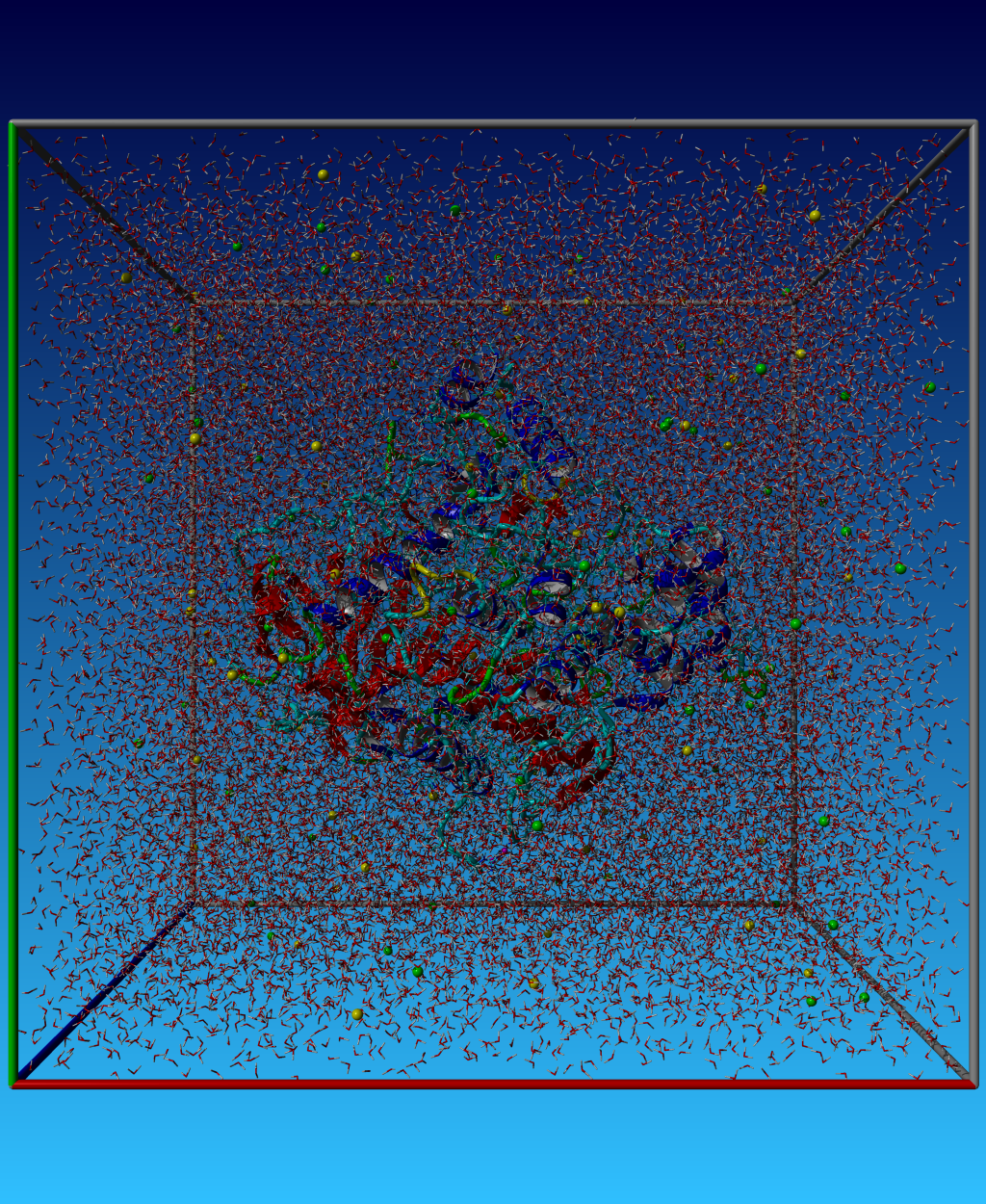

Supplement: S7 File — (ZIP) [file pone.0338211.s007.zip › S6.Molecular Dynamic Simulation/S6.Molecular Dynamic Simulation/MDS result for Donepezil_hAChE/Donepezil & hAChE - Al amin Afendy_report_figure1.png]

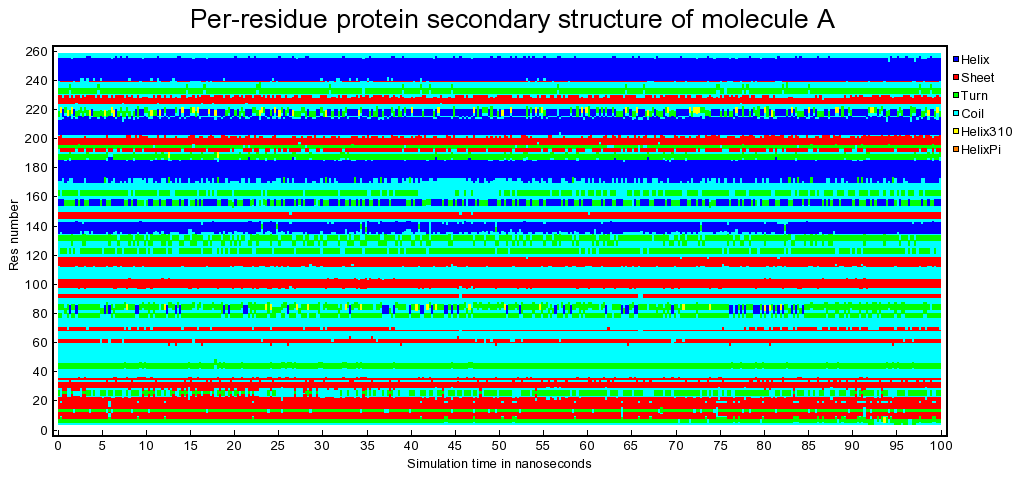

Supplement: S7 File — (ZIP) [file pone.0338211.s007.zip › S6.Molecular Dynamic Simulation/S6.Molecular Dynamic Simulation/MDS result for Donepezil_hAChE/Donepezil & hAChE - Al amin Afendy_report_figure10.png]

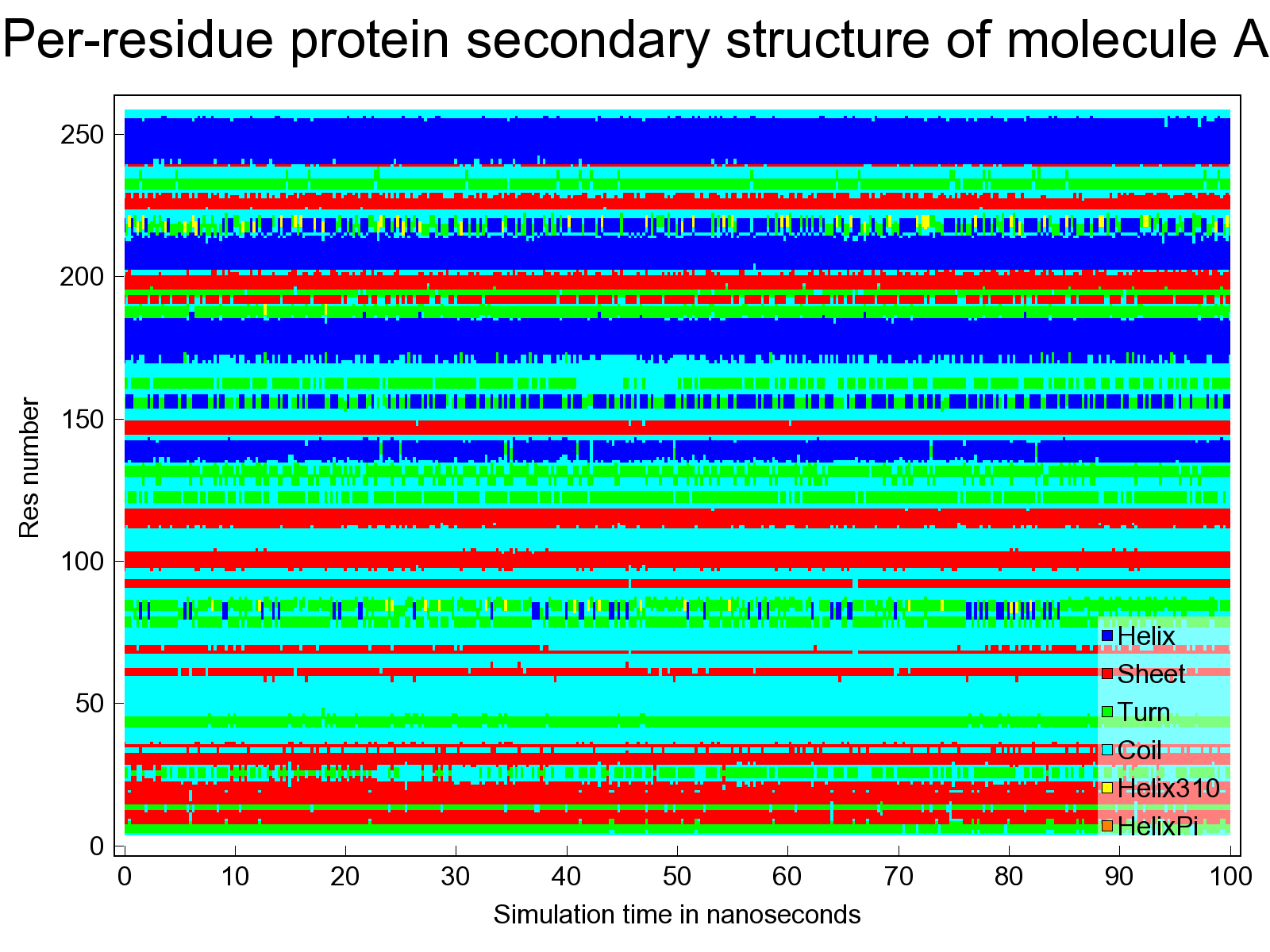

Supplement: S7 File — (ZIP) [file pone.0338211.s007.zip › S6.Molecular Dynamic Simulation/S6.Molecular Dynamic Simulation/MDS result for Donepezil_hAChE/Donepezil & hAChE - Al amin Afendy_report_figure10_hires.png]

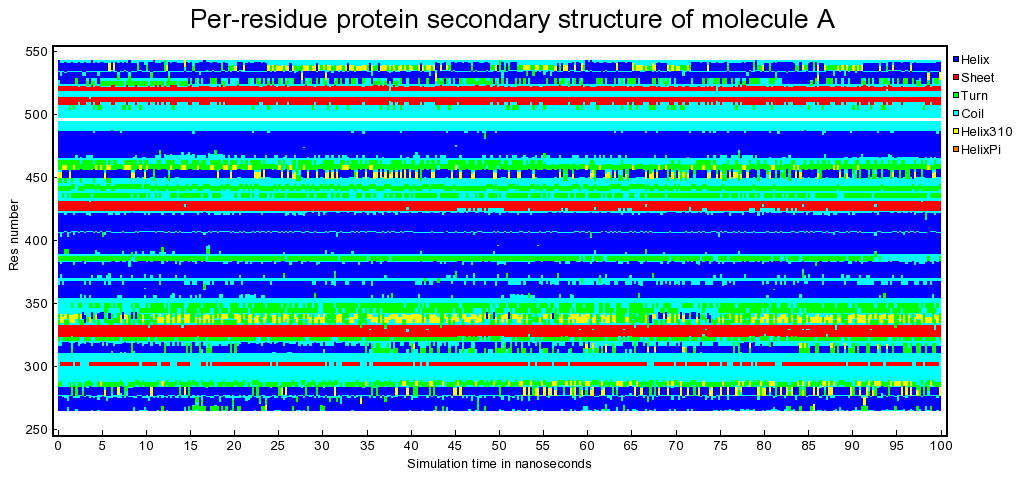

Supplement: S7 File — (ZIP) [file pone.0338211.s007.zip › S6.Molecular Dynamic Simulation/S6.Molecular Dynamic Simulation/MDS result for Donepezil_hAChE/Donepezil & hAChE - Al amin Afendy_report_figure11.png]

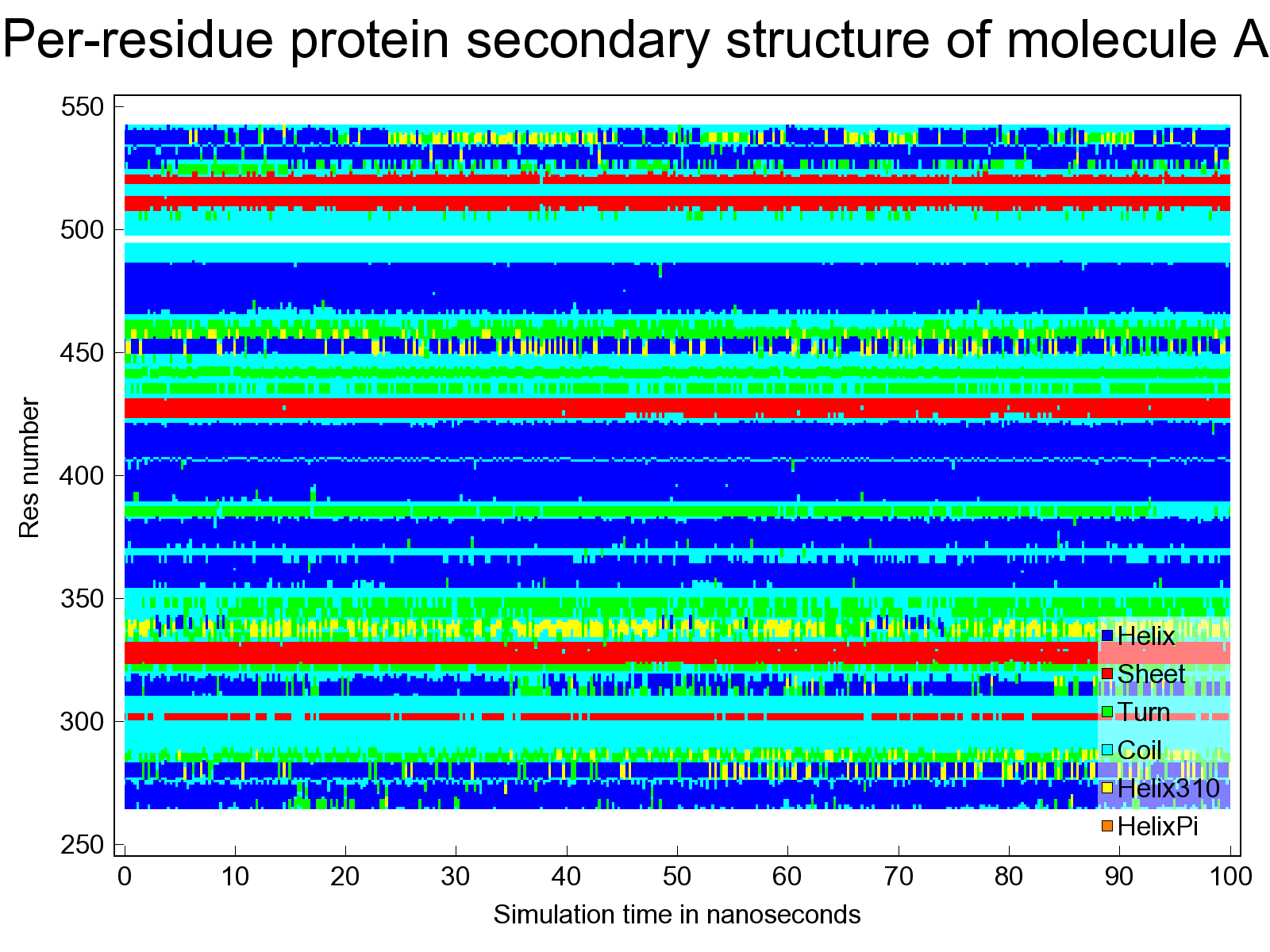

Supplement: S7 File — (ZIP) [file pone.0338211.s007.zip › S6.Molecular Dynamic Simulation/S6.Molecular Dynamic Simulation/MDS result for Donepezil_hAChE/Donepezil & hAChE - Al amin Afendy_report_figure11_hires.png]

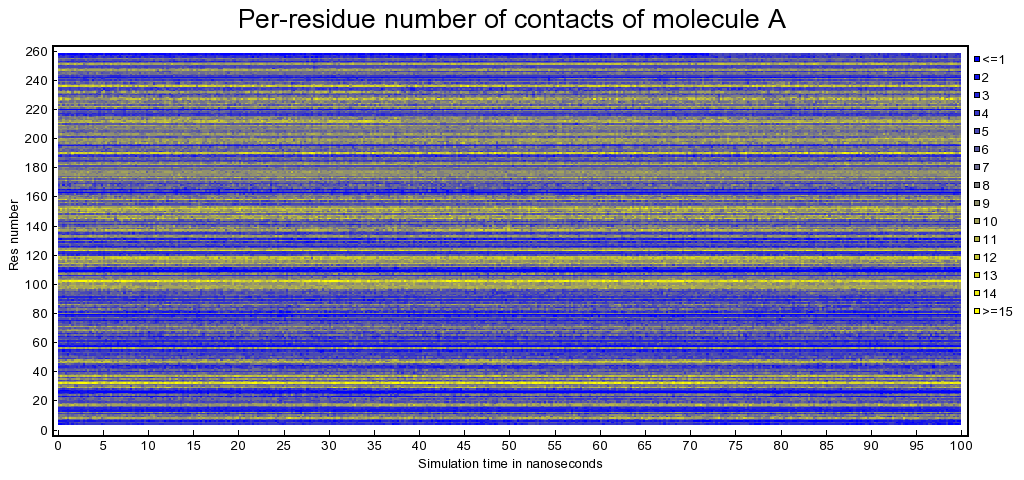

Supplement: S7 File — (ZIP) [file pone.0338211.s007.zip › S6.Molecular Dynamic Simulation/S6.Molecular Dynamic Simulation/MDS result for Donepezil_hAChE/Donepezil & hAChE - Al amin Afendy_report_figure12.png]

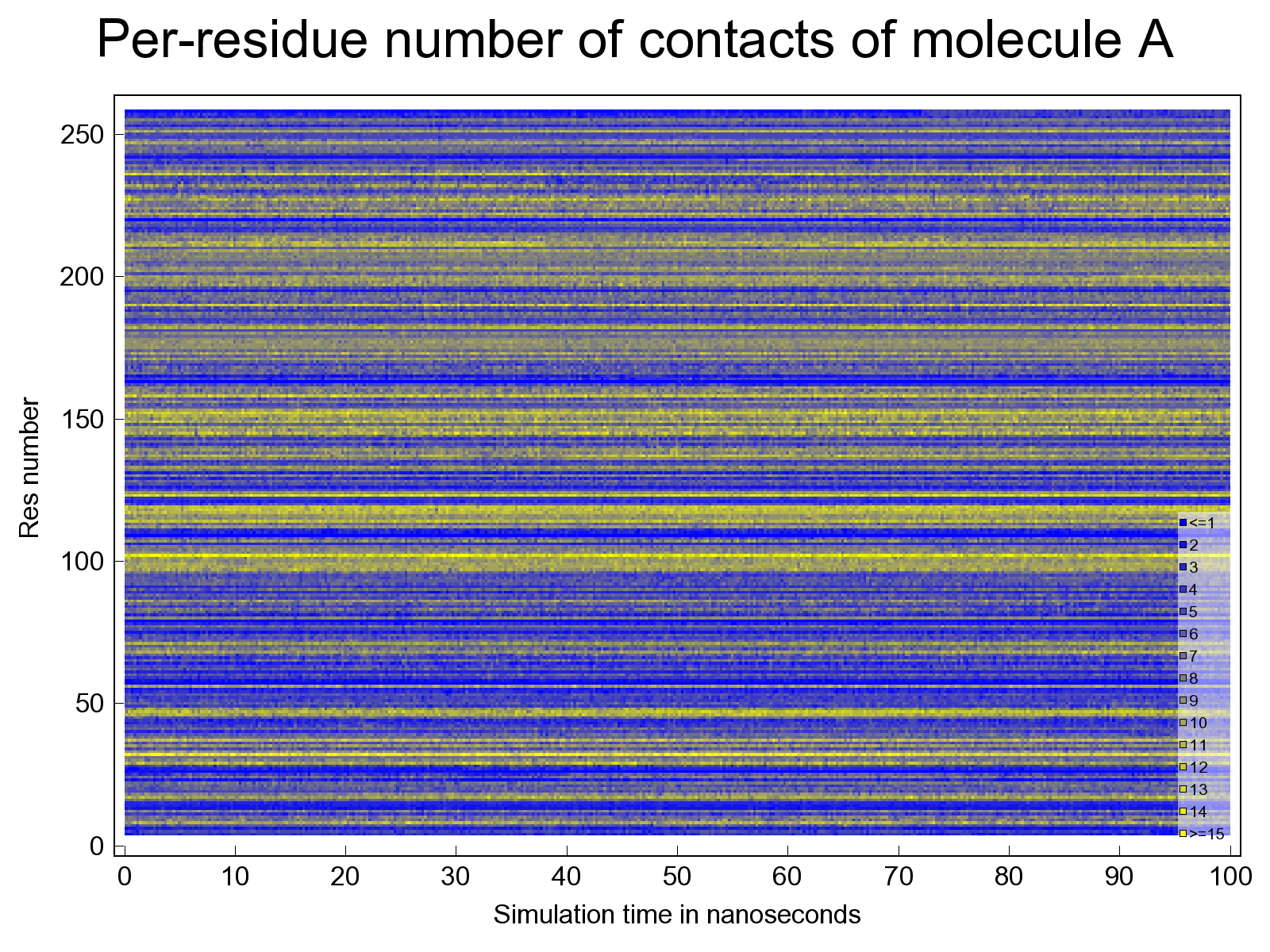

Supplement: S7 File — (ZIP) [file pone.0338211.s007.zip › S6.Molecular Dynamic Simulation/S6.Molecular Dynamic Simulation/MDS result for Donepezil_hAChE/Donepezil & hAChE - Al amin Afendy_report_figure12_hires.png]

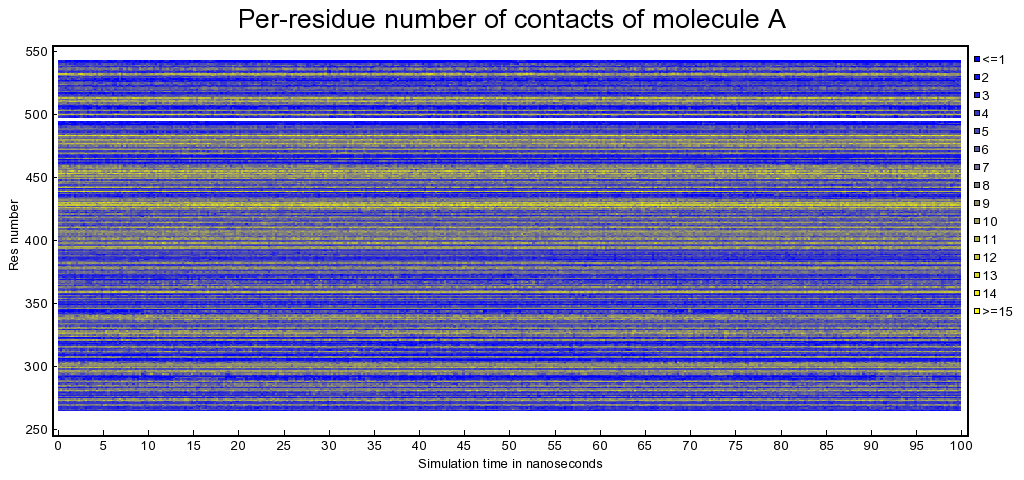

Supplement: S7 File — (ZIP) [file pone.0338211.s007.zip › S6.Molecular Dynamic Simulation/S6.Molecular Dynamic Simulation/MDS result for Donepezil_hAChE/Donepezil & hAChE - Al amin Afendy_report_figure13.png]

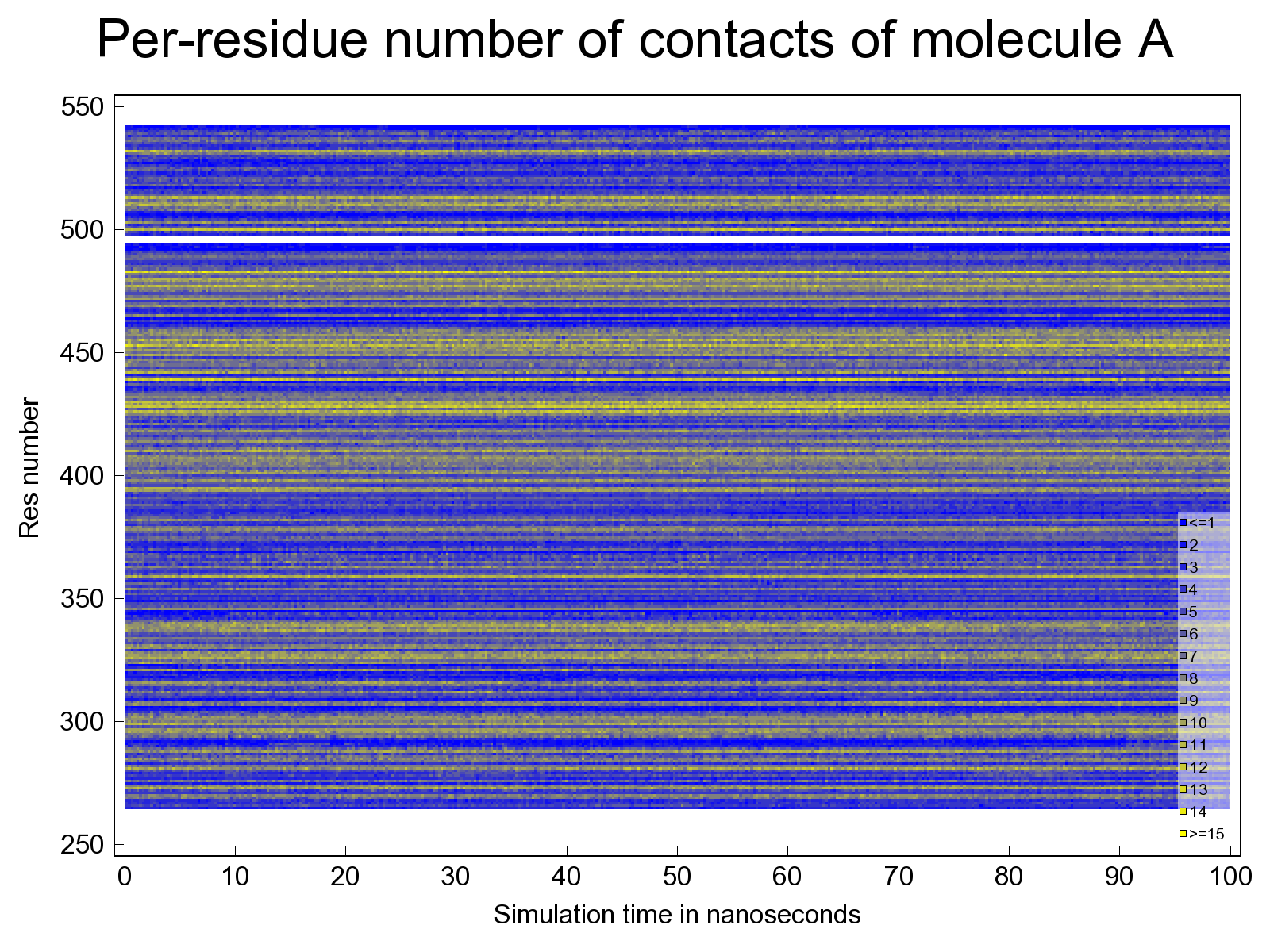

Supplement: S7 File — (ZIP) [file pone.0338211.s007.zip › S6.Molecular Dynamic Simulation/S6.Molecular Dynamic Simulation/MDS result for Donepezil_hAChE/Donepezil & hAChE - Al amin Afendy_report_figure13_hires.png]

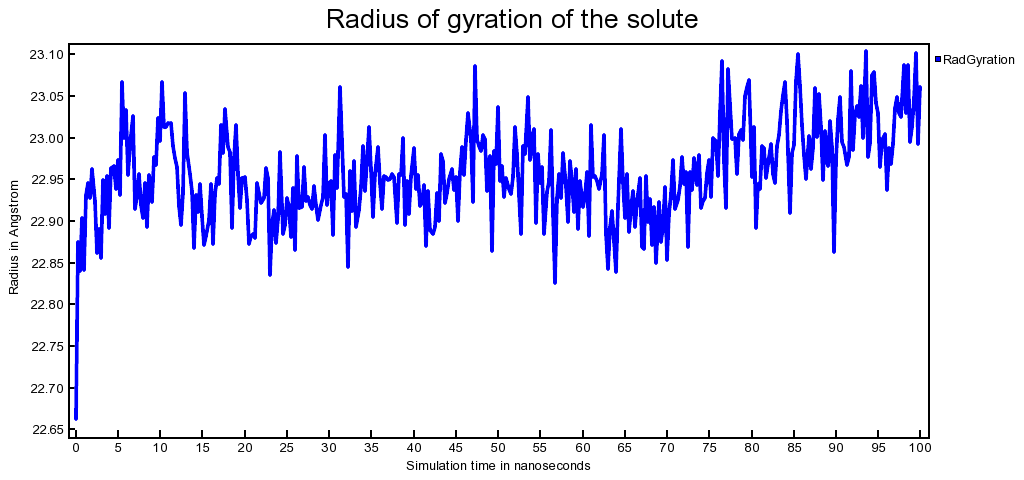

Supplement: S7 File — (ZIP) [file pone.0338211.s007.zip › S6.Molecular Dynamic Simulation/S6.Molecular Dynamic Simulation/MDS result for Donepezil_hAChE/Donepezil & hAChE - Al amin Afendy_report_figure14.png]

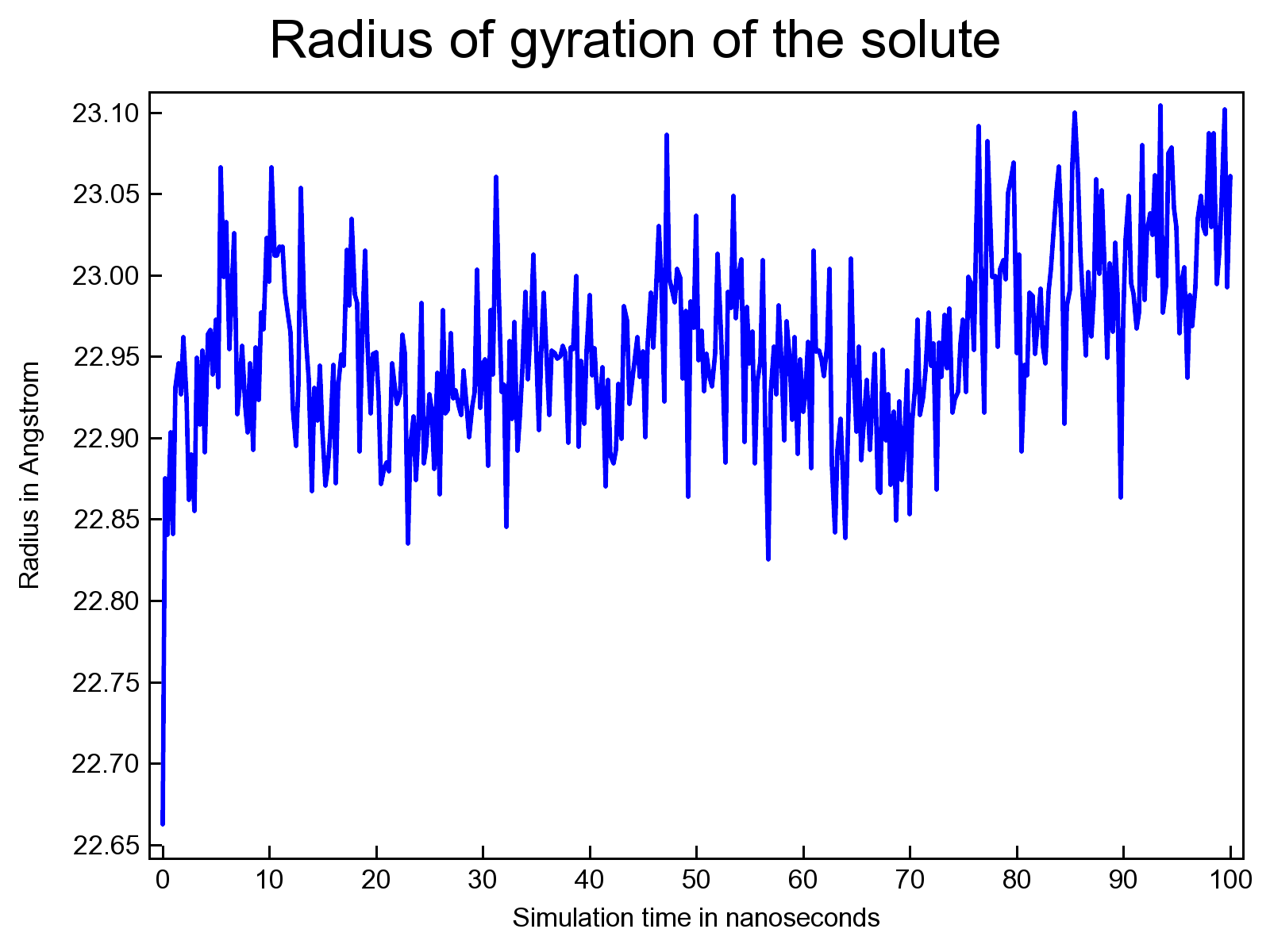

Supplement: S7 File — (ZIP) [file pone.0338211.s007.zip › S6.Molecular Dynamic Simulation/S6.Molecular Dynamic Simulation/MDS result for Donepezil_hAChE/Donepezil & hAChE - Al amin Afendy_report_figure14_hires.png]

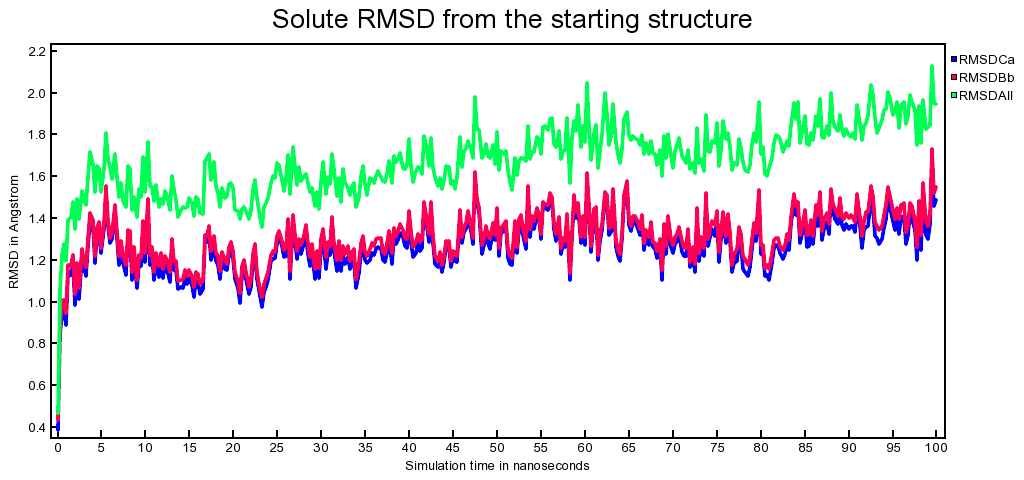

Supplement: S7 File — (ZIP) [file pone.0338211.s007.zip › S6.Molecular Dynamic Simulation/S6.Molecular Dynamic Simulation/MDS result for Donepezil_hAChE/Donepezil & hAChE - Al amin Afendy_report_figure15.png]

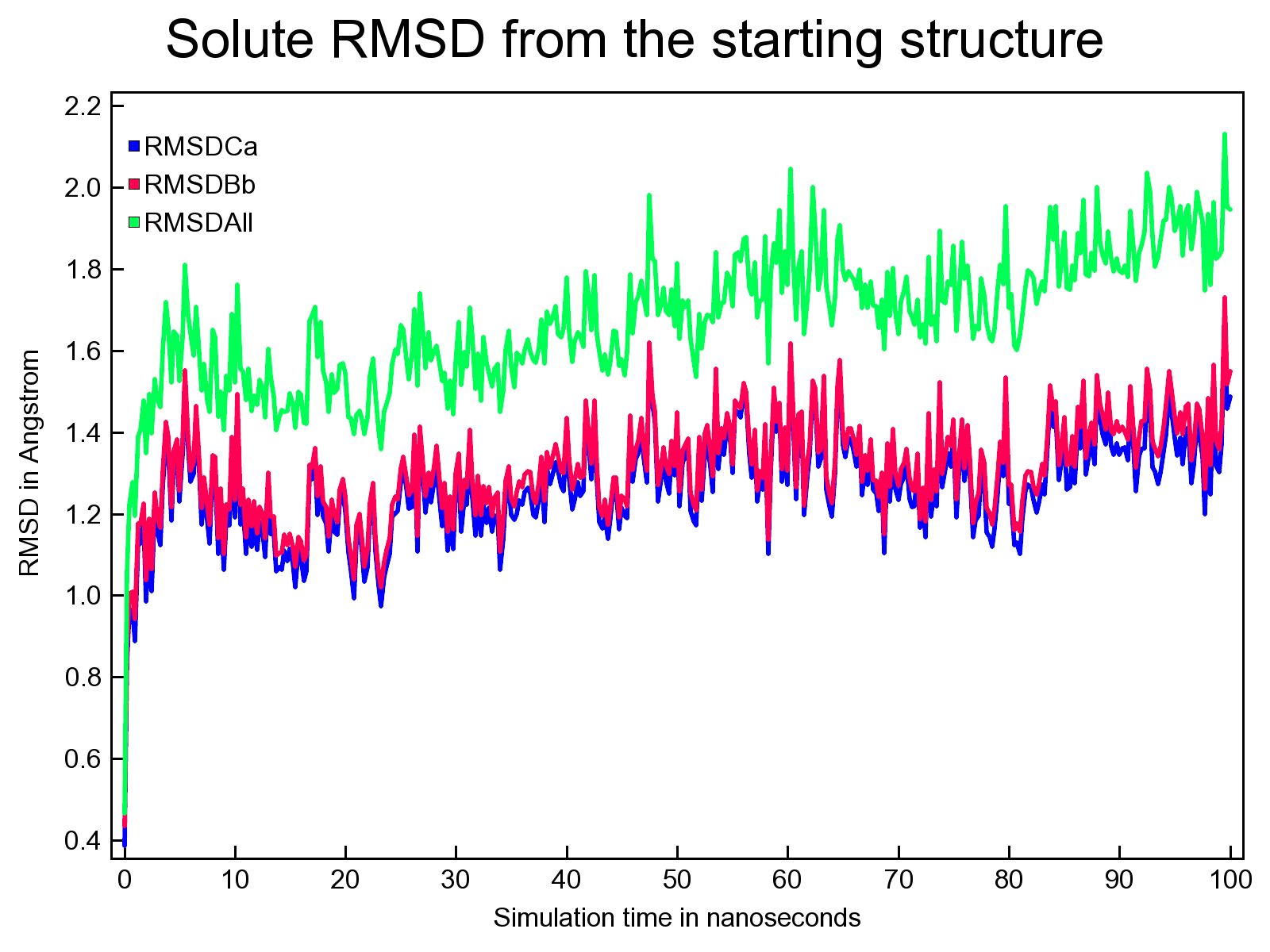

Supplement: S7 File — (ZIP) [file pone.0338211.s007.zip › S6.Molecular Dynamic Simulation/S6.Molecular Dynamic Simulation/MDS result for Donepezil_hAChE/Donepezil & hAChE - Al amin Afendy_report_figure15_hires.png]

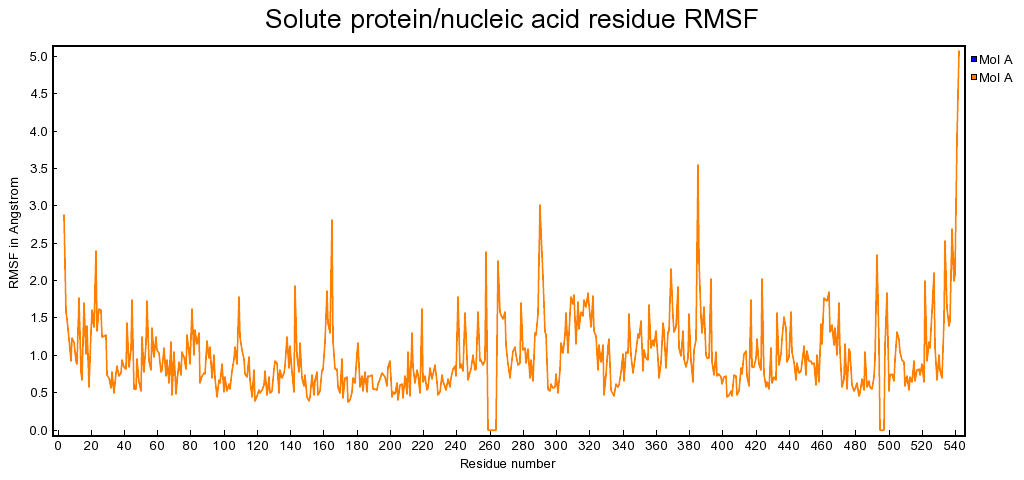

Supplement: S7 File — (ZIP) [file pone.0338211.s007.zip › S6.Molecular Dynamic Simulation/S6.Molecular Dynamic Simulation/MDS result for Donepezil_hAChE/Donepezil & hAChE - Al amin Afendy_report_figure16.png]

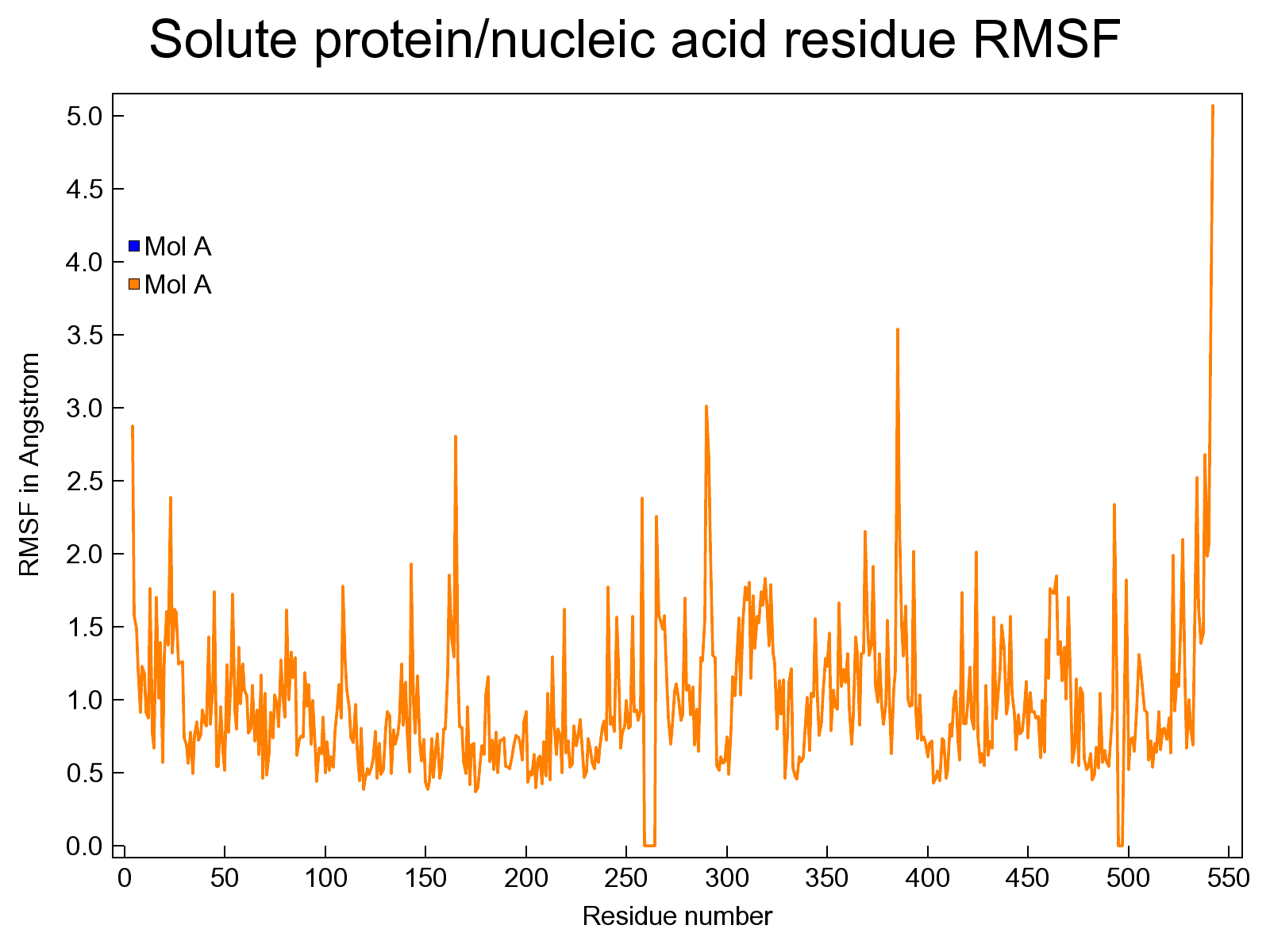

Supplement: S7 File — (ZIP) [file pone.0338211.s007.zip › S6.Molecular Dynamic Simulation/S6.Molecular Dynamic Simulation/MDS result for Donepezil_hAChE/Donepezil & hAChE - Al amin Afendy_report_figure16_hires.png]

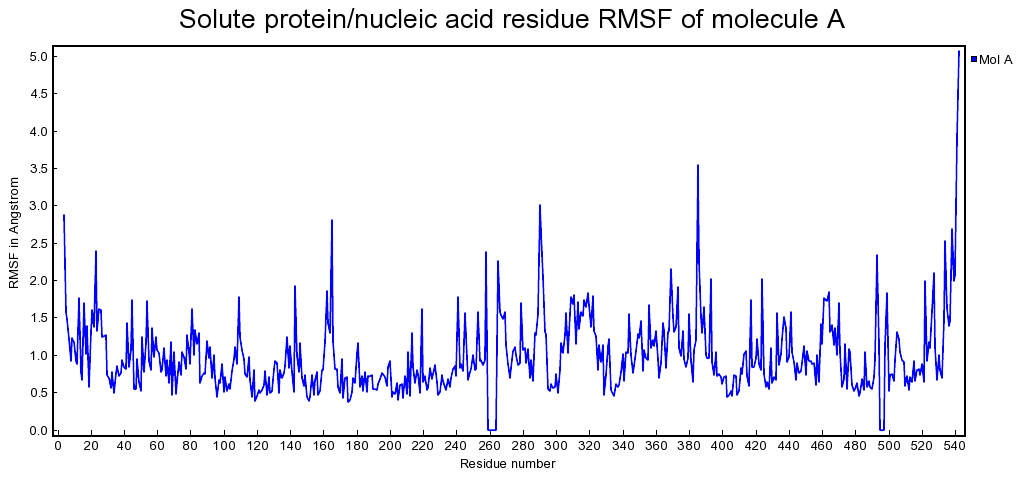

Supplement: S7 File — (ZIP) [file pone.0338211.s007.zip › S6.Molecular Dynamic Simulation/S6.Molecular Dynamic Simulation/MDS result for Donepezil_hAChE/Donepezil & hAChE - Al amin Afendy_report_figure17.png]

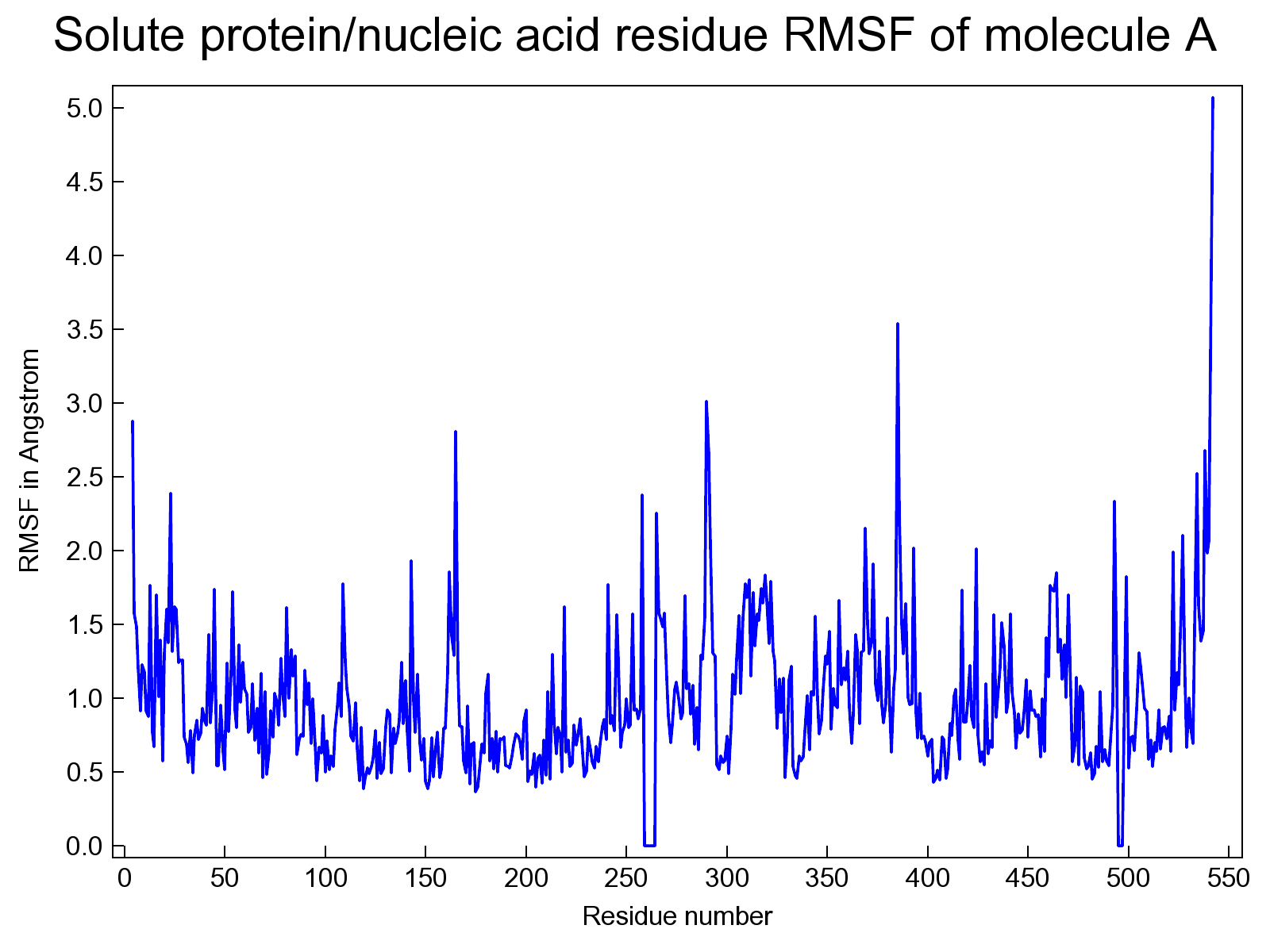

Supplement: S7 File — (ZIP) [file pone.0338211.s007.zip › S6.Molecular Dynamic Simulation/S6.Molecular Dynamic Simulation/MDS result for Donepezil_hAChE/Donepezil & hAChE - Al amin Afendy_report_figure17_hires.png]

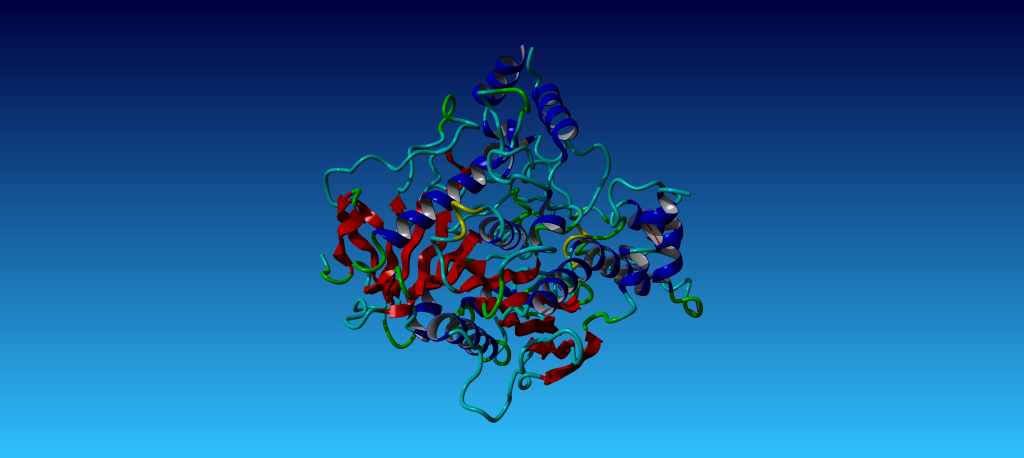

Supplement: S7 File — (ZIP) [file pone.0338211.s007.zip › S6.Molecular Dynamic Simulation/S6.Molecular Dynamic Simulation/MDS result for Donepezil_hAChE/Donepezil & hAChE - Al amin Afendy_report_figure19.png]

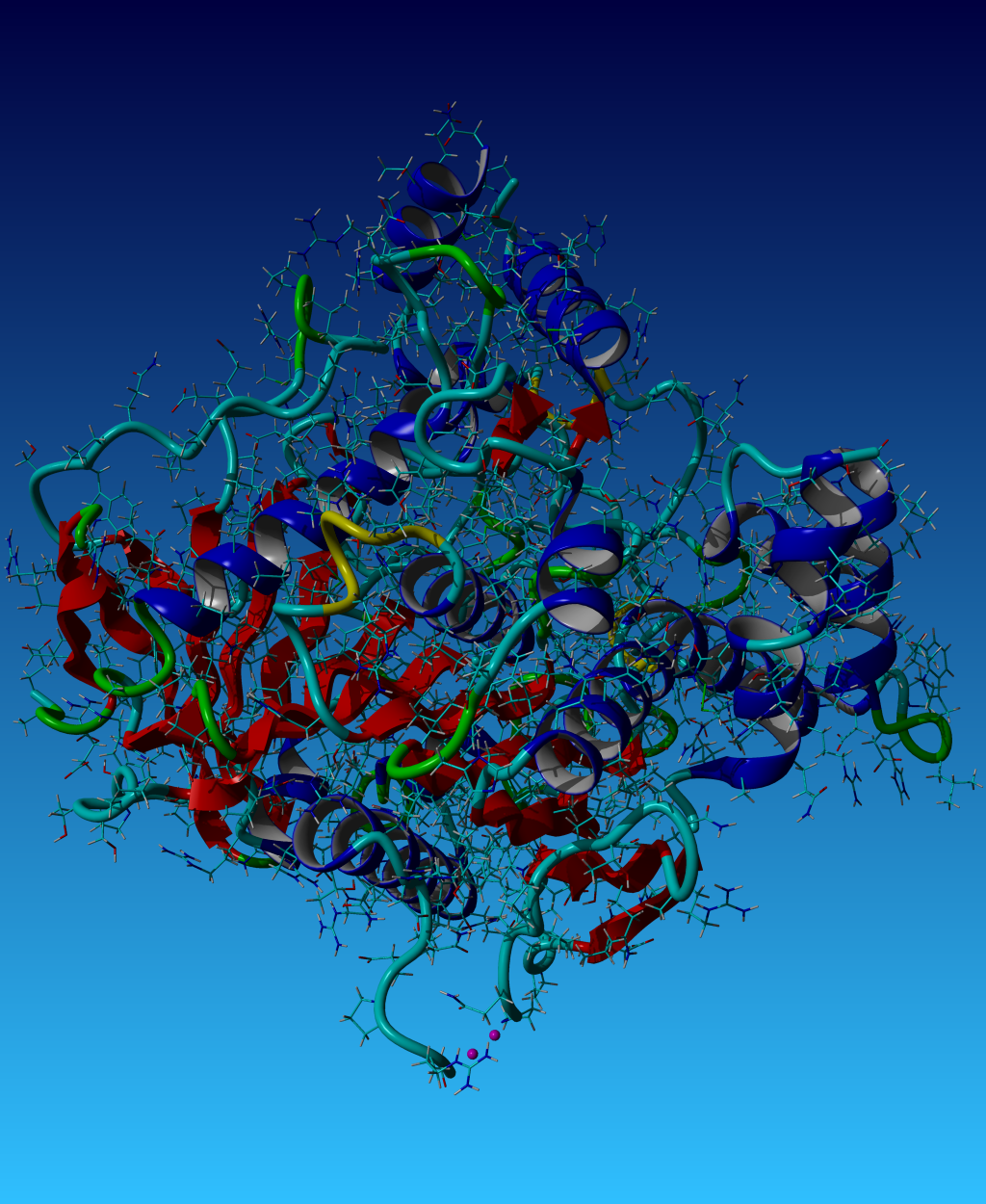

Supplement: S7 File — (ZIP) [file pone.0338211.s007.zip › S6.Molecular Dynamic Simulation/S6.Molecular Dynamic Simulation/MDS result for Donepezil_hAChE/Donepezil & hAChE - Al amin Afendy_report_figure2.png]

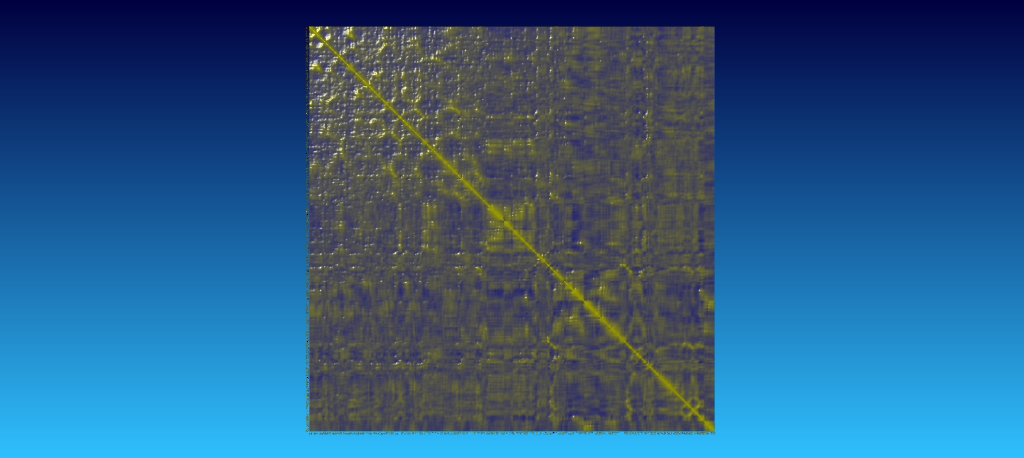

Supplement: S7 File — (ZIP) [file pone.0338211.s007.zip › S6.Molecular Dynamic Simulation/S6.Molecular Dynamic Simulation/MDS result for Donepezil_hAChE/Donepezil & hAChE - Al amin Afendy_report_figure20.png]

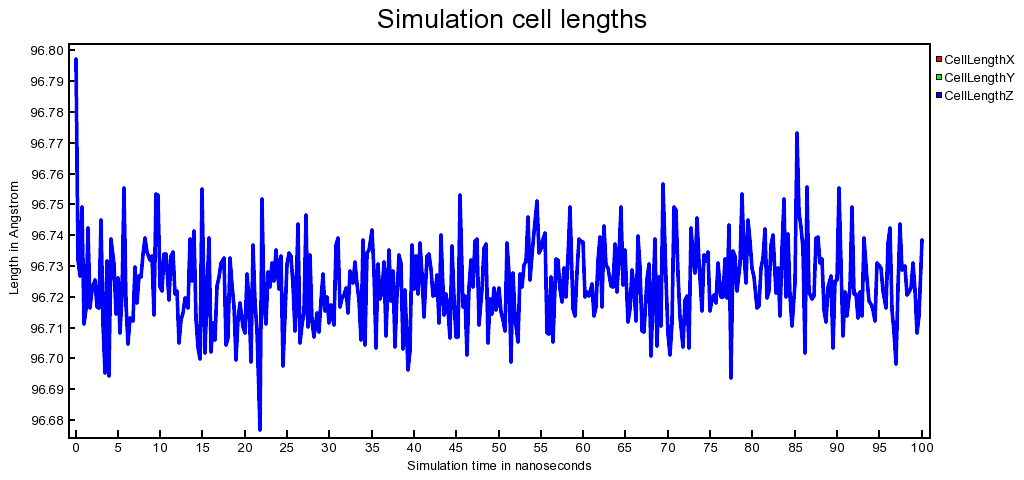

Supplement: S7 File — (ZIP) [file pone.0338211.s007.zip › S6.Molecular Dynamic Simulation/S6.Molecular Dynamic Simulation/MDS result for Donepezil_hAChE/Donepezil & hAChE - Al amin Afendy_report_figure3.png]

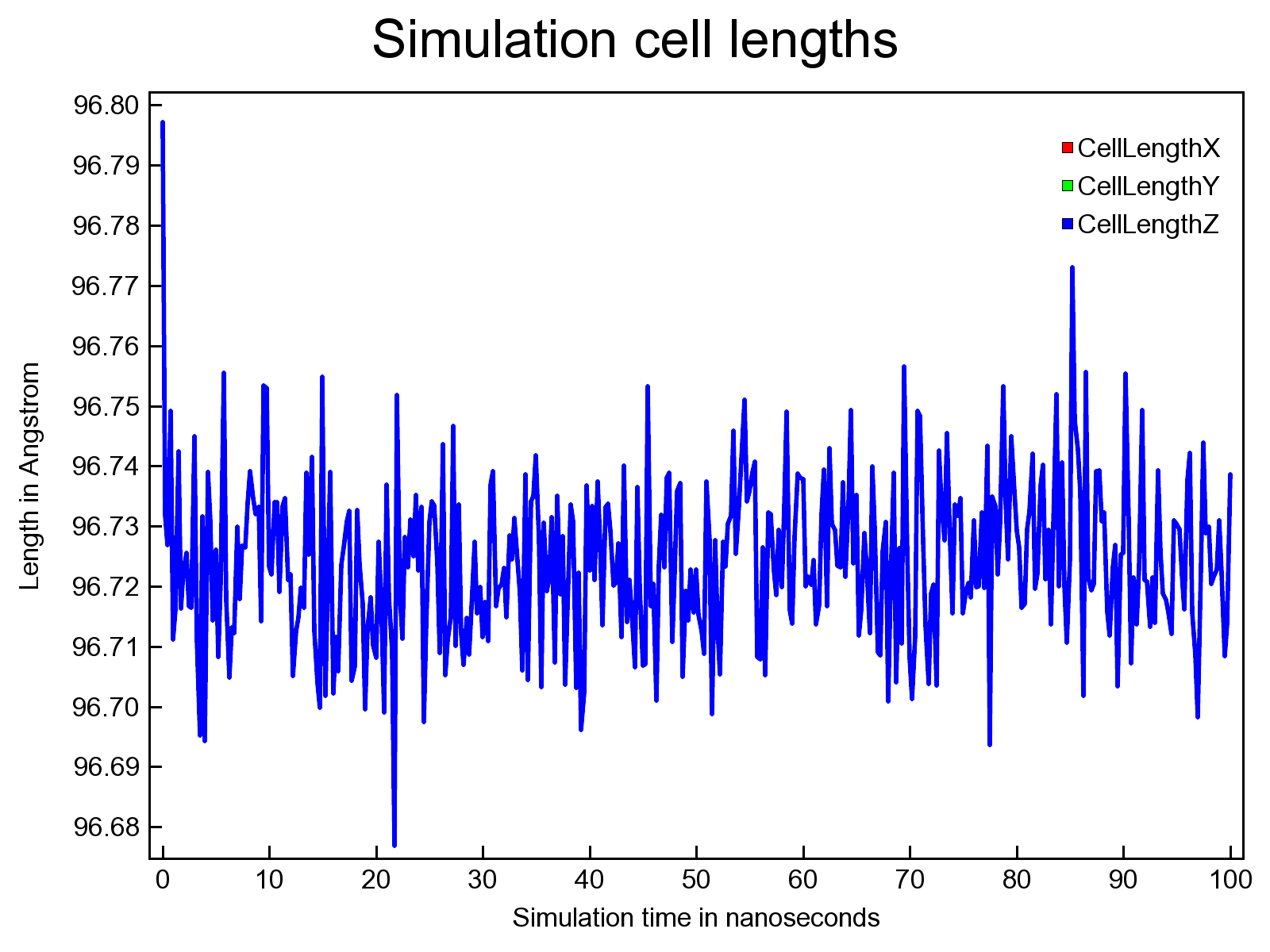

Supplement: S7 File — (ZIP) [file pone.0338211.s007.zip › S6.Molecular Dynamic Simulation/S6.Molecular Dynamic Simulation/MDS result for Donepezil_hAChE/Donepezil & hAChE - Al amin Afendy_report_figure3_hires.png]

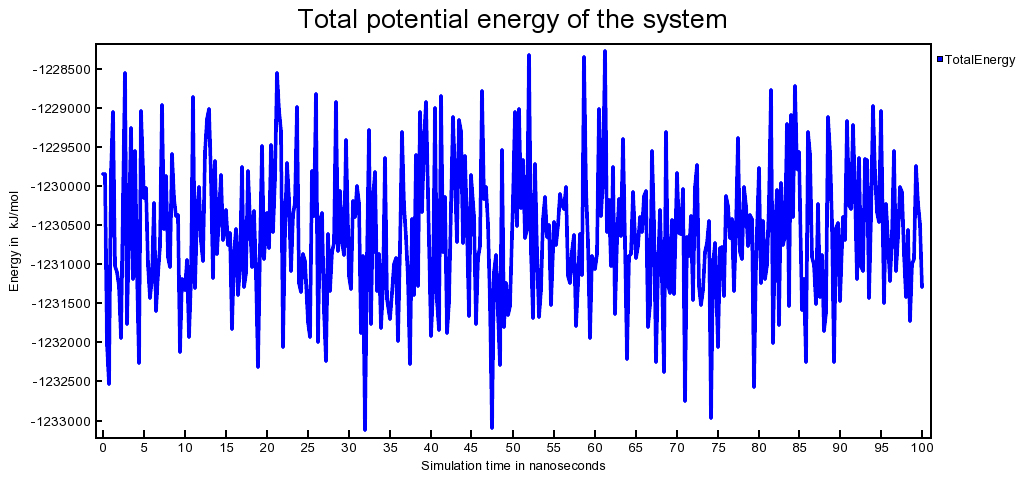

Supplement: S7 File — (ZIP) [file pone.0338211.s007.zip › S6.Molecular Dynamic Simulation/S6.Molecular Dynamic Simulation/MDS result for Donepezil_hAChE/Donepezil & hAChE - Al amin Afendy_report_figure4.png]

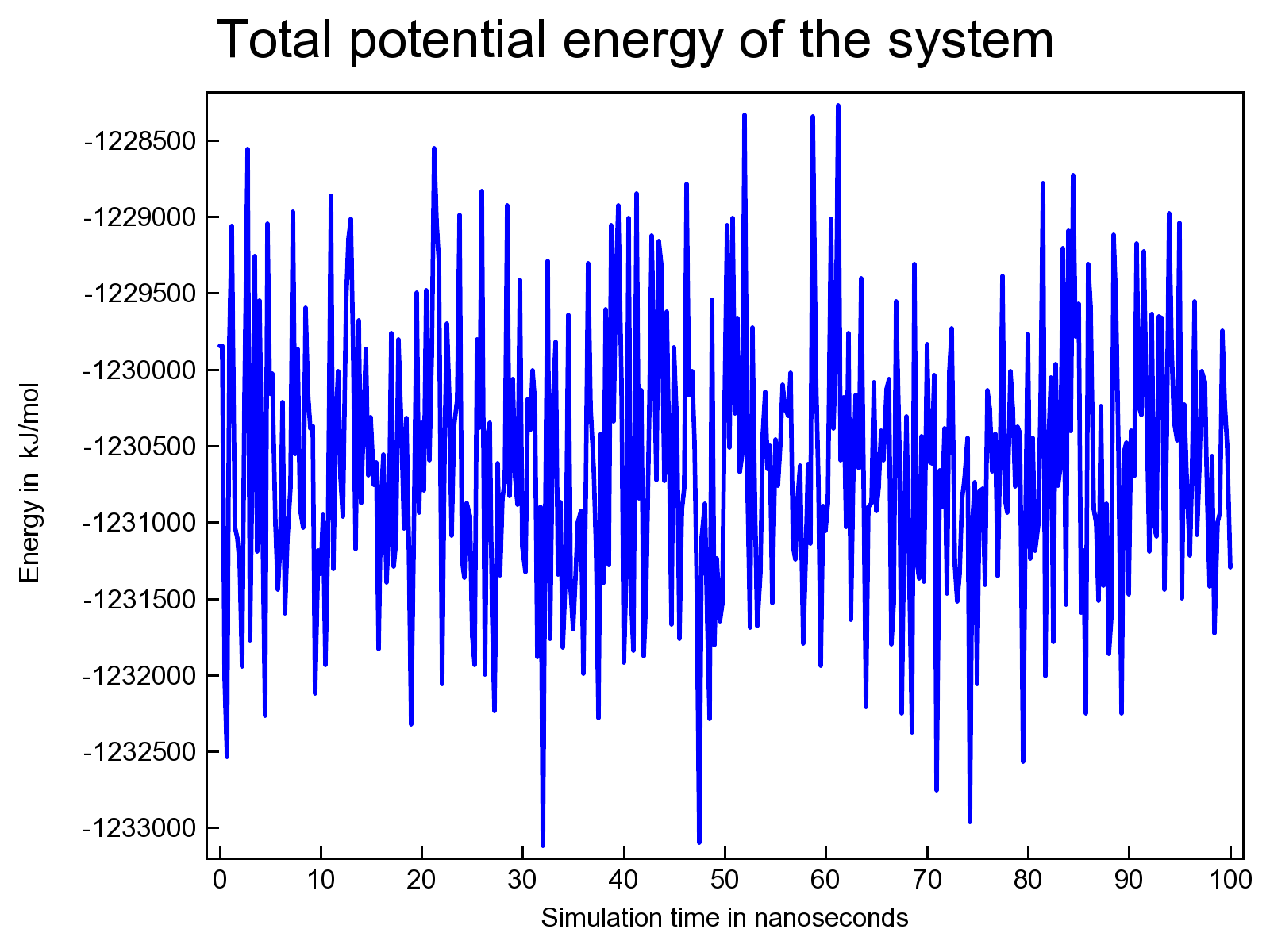

Supplement: S7 File — (ZIP) [file pone.0338211.s007.zip › S6.Molecular Dynamic Simulation/S6.Molecular Dynamic Simulation/MDS result for Donepezil_hAChE/Donepezil & hAChE - Al amin Afendy_report_figure4_hires.png]

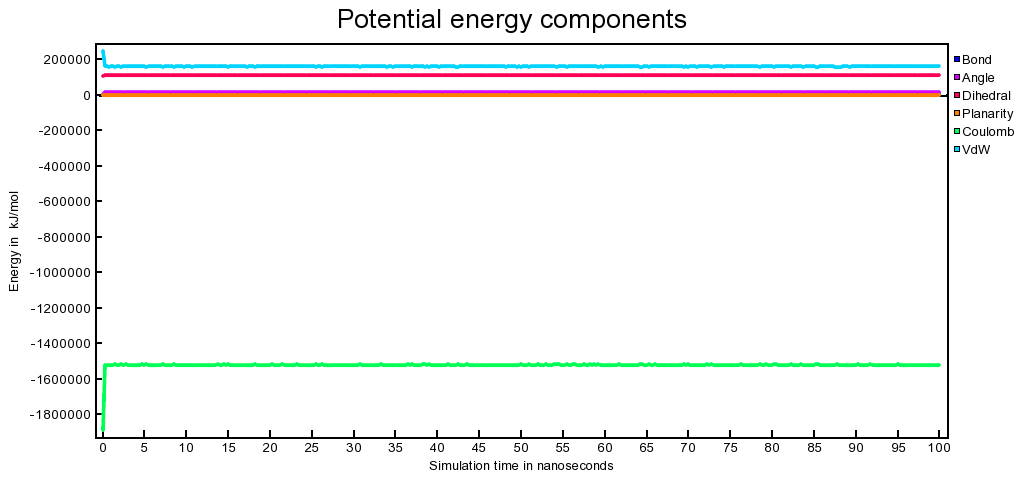

Supplement: S7 File — (ZIP) [file pone.0338211.s007.zip › S6.Molecular Dynamic Simulation/S6.Molecular Dynamic Simulation/MDS result for Donepezil_hAChE/Donepezil & hAChE - Al amin Afendy_report_figure5.png]

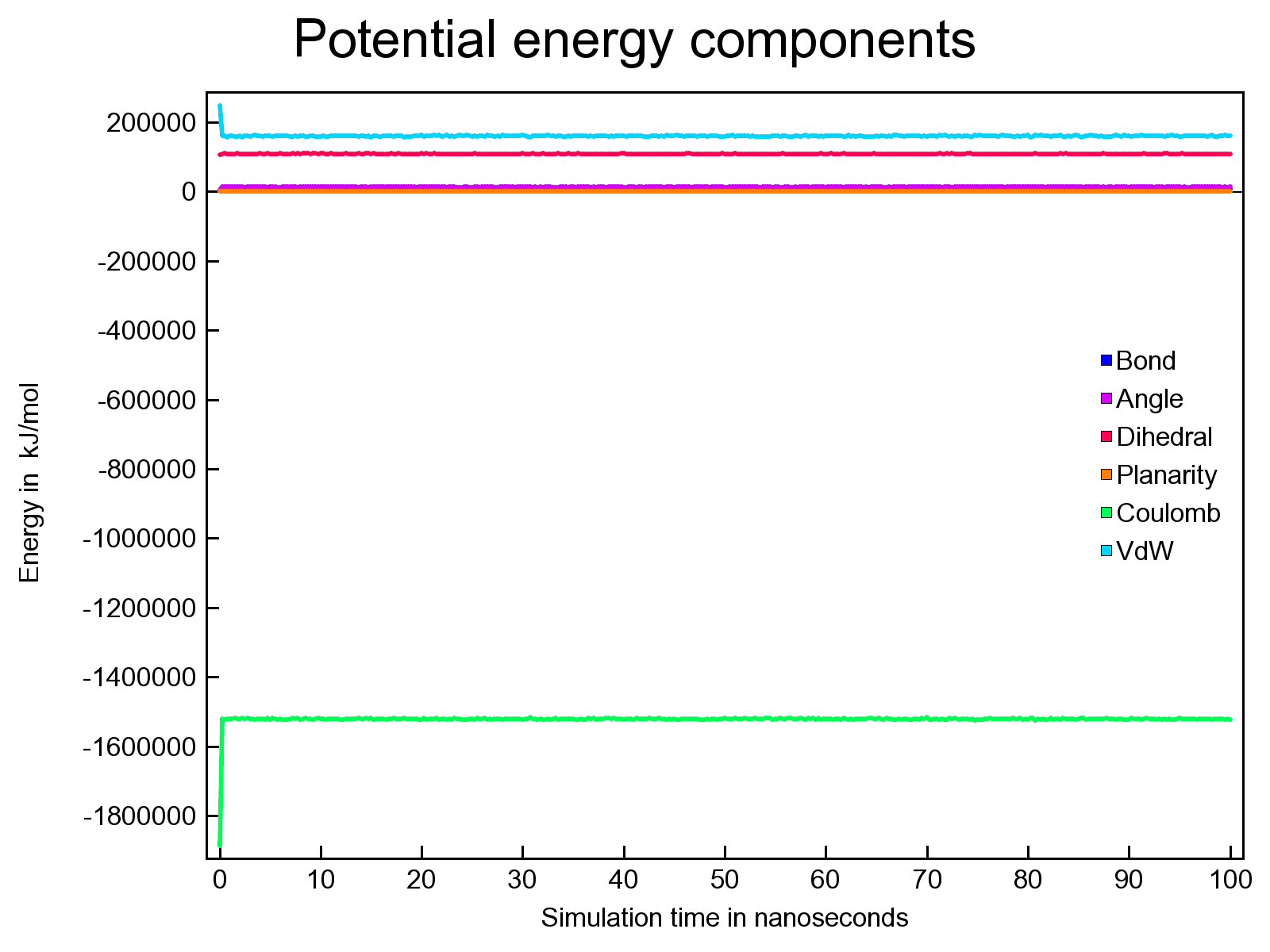

Supplement: S7 File — (ZIP) [file pone.0338211.s007.zip › S6.Molecular Dynamic Simulation/S6.Molecular Dynamic Simulation/MDS result for Donepezil_hAChE/Donepezil & hAChE - Al amin Afendy_report_figure5_hires.png]

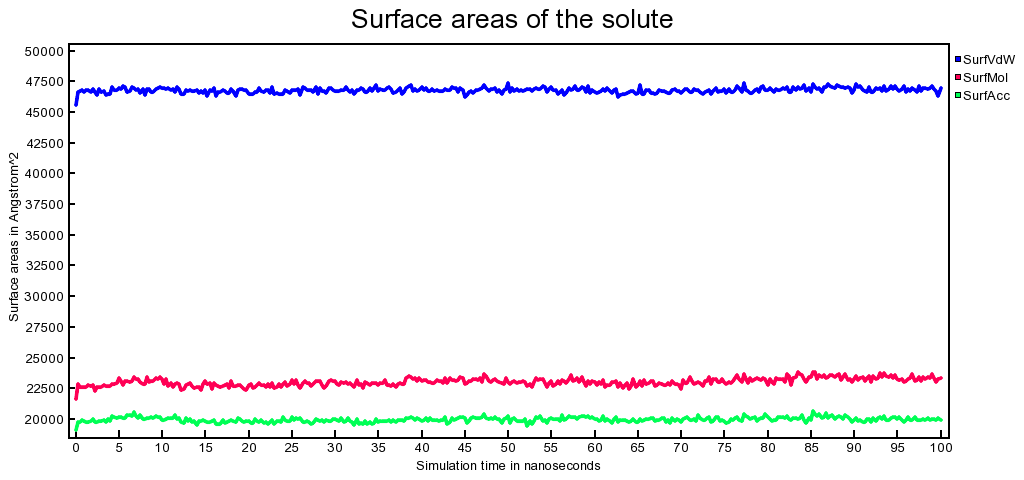

Supplement: S7 File — (ZIP) [file pone.0338211.s007.zip › S6.Molecular Dynamic Simulation/S6.Molecular Dynamic Simulation/MDS result for Donepezil_hAChE/Donepezil & hAChE - Al amin Afendy_report_figure6.png]

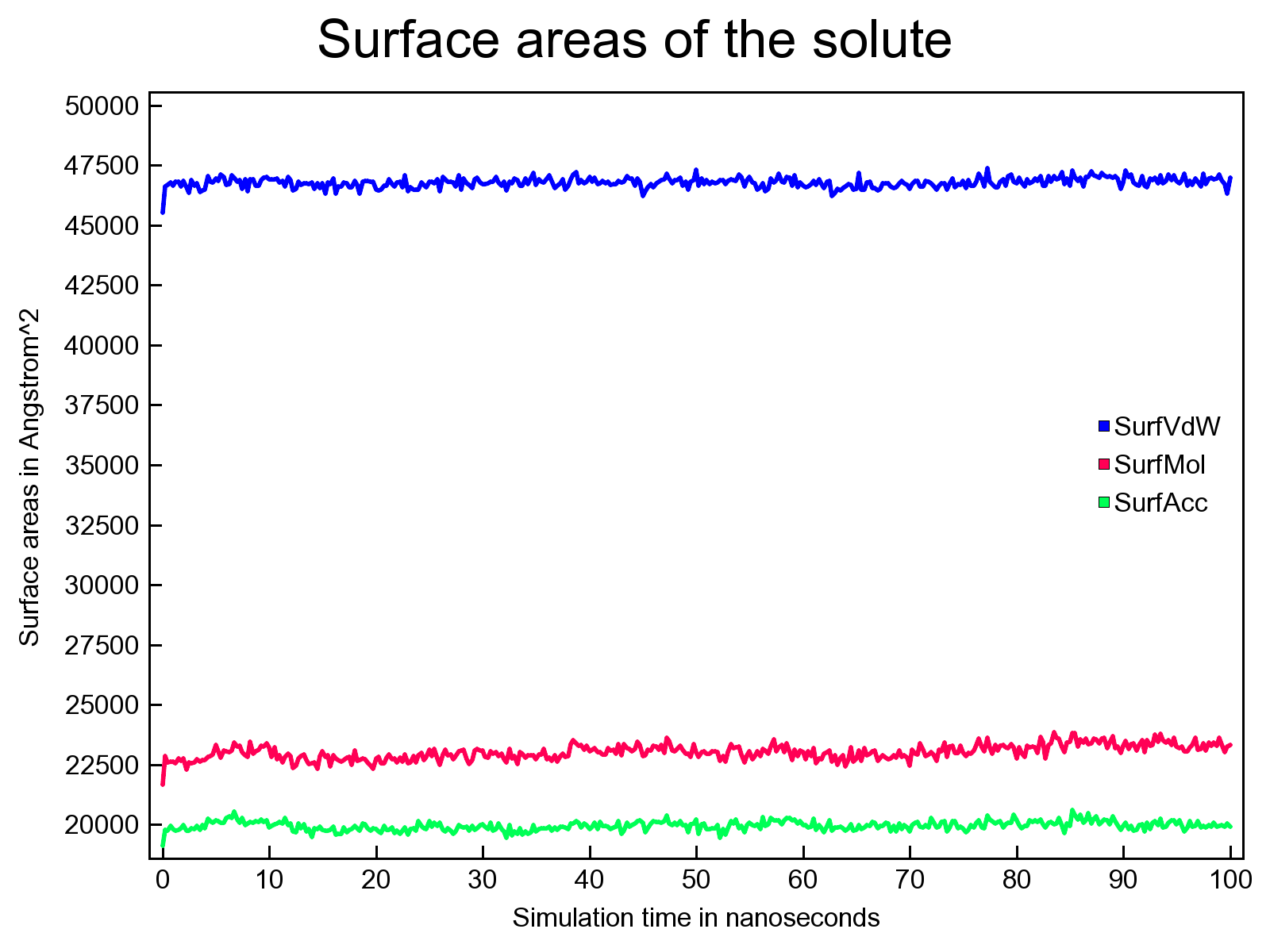

Supplement: S7 File — (ZIP) [file pone.0338211.s007.zip › S6.Molecular Dynamic Simulation/S6.Molecular Dynamic Simulation/MDS result for Donepezil_hAChE/Donepezil & hAChE - Al amin Afendy_report_figure6_hires.png]

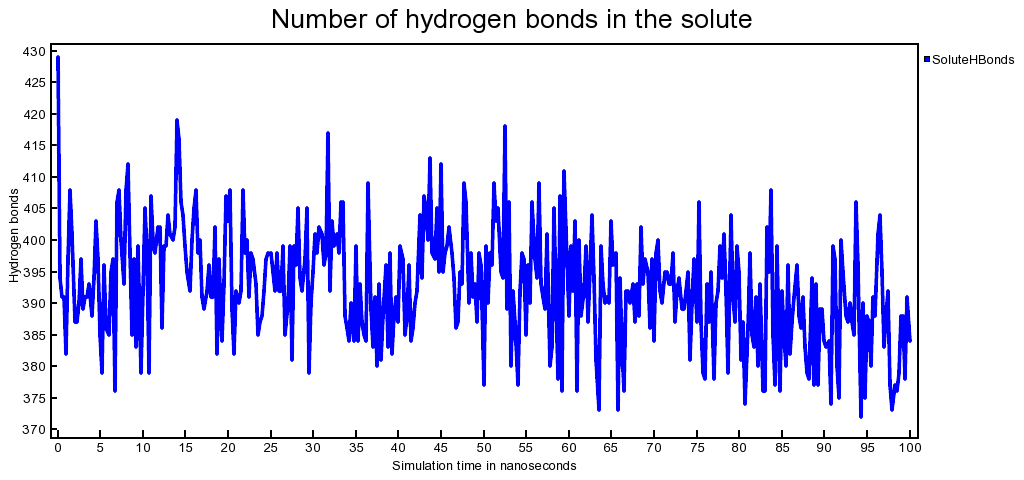

Supplement: S7 File — (ZIP) [file pone.0338211.s007.zip › S6.Molecular Dynamic Simulation/S6.Molecular Dynamic Simulation/MDS result for Donepezil_hAChE/Donepezil & hAChE - Al amin Afendy_report_figure7.png]

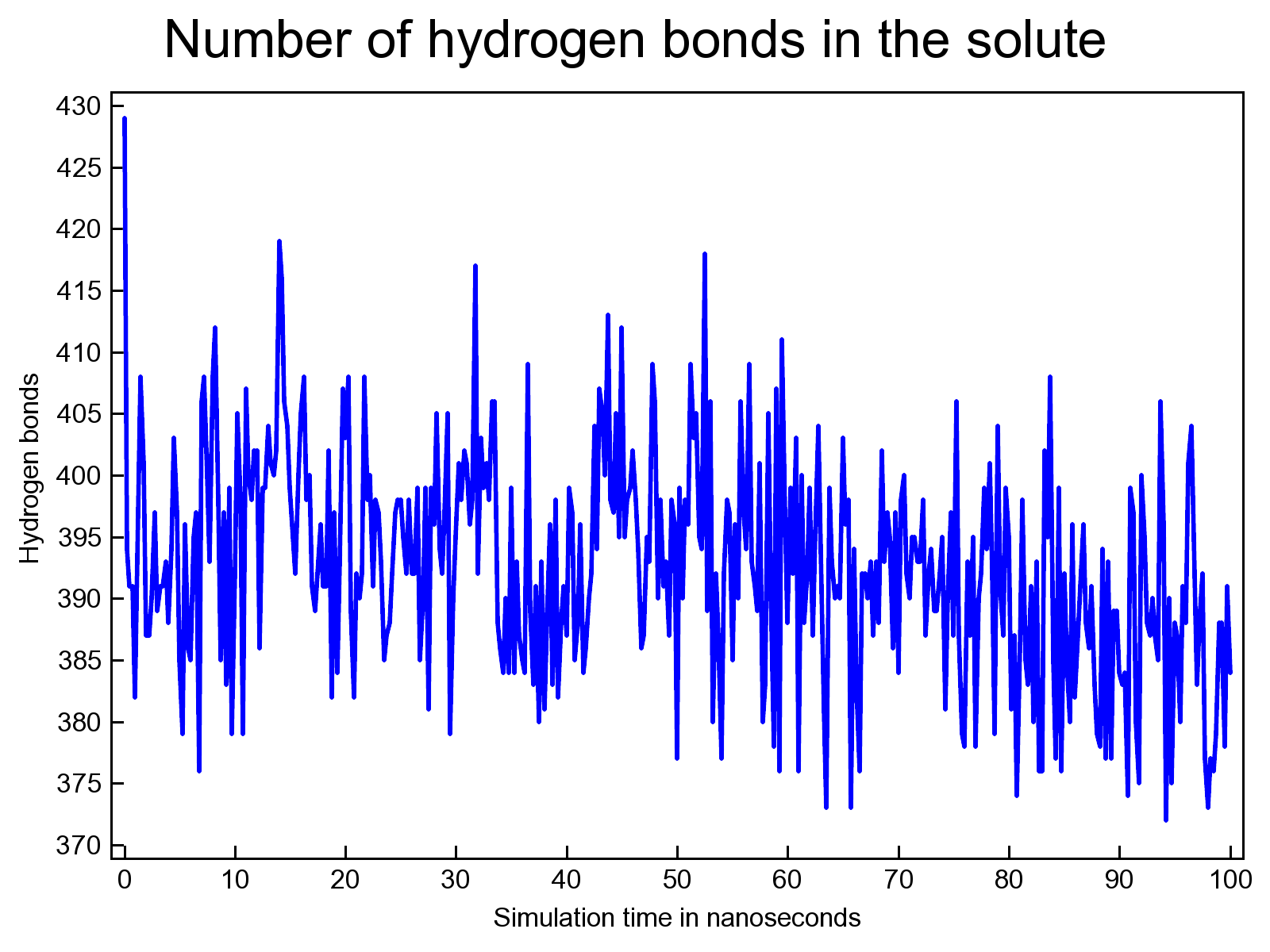

Supplement: S7 File — (ZIP) [file pone.0338211.s007.zip › S6.Molecular Dynamic Simulation/S6.Molecular Dynamic Simulation/MDS result for Donepezil_hAChE/Donepezil & hAChE - Al amin Afendy_report_figure7_hires.png]

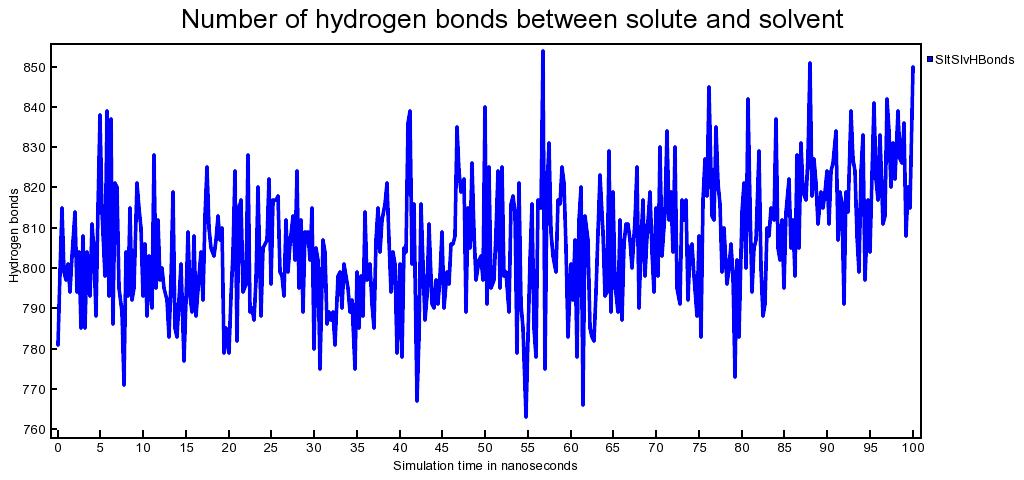

Supplement: S7 File — (ZIP) [file pone.0338211.s007.zip › S6.Molecular Dynamic Simulation/S6.Molecular Dynamic Simulation/MDS result for Donepezil_hAChE/Donepezil & hAChE - Al amin Afendy_report_figure8.png]

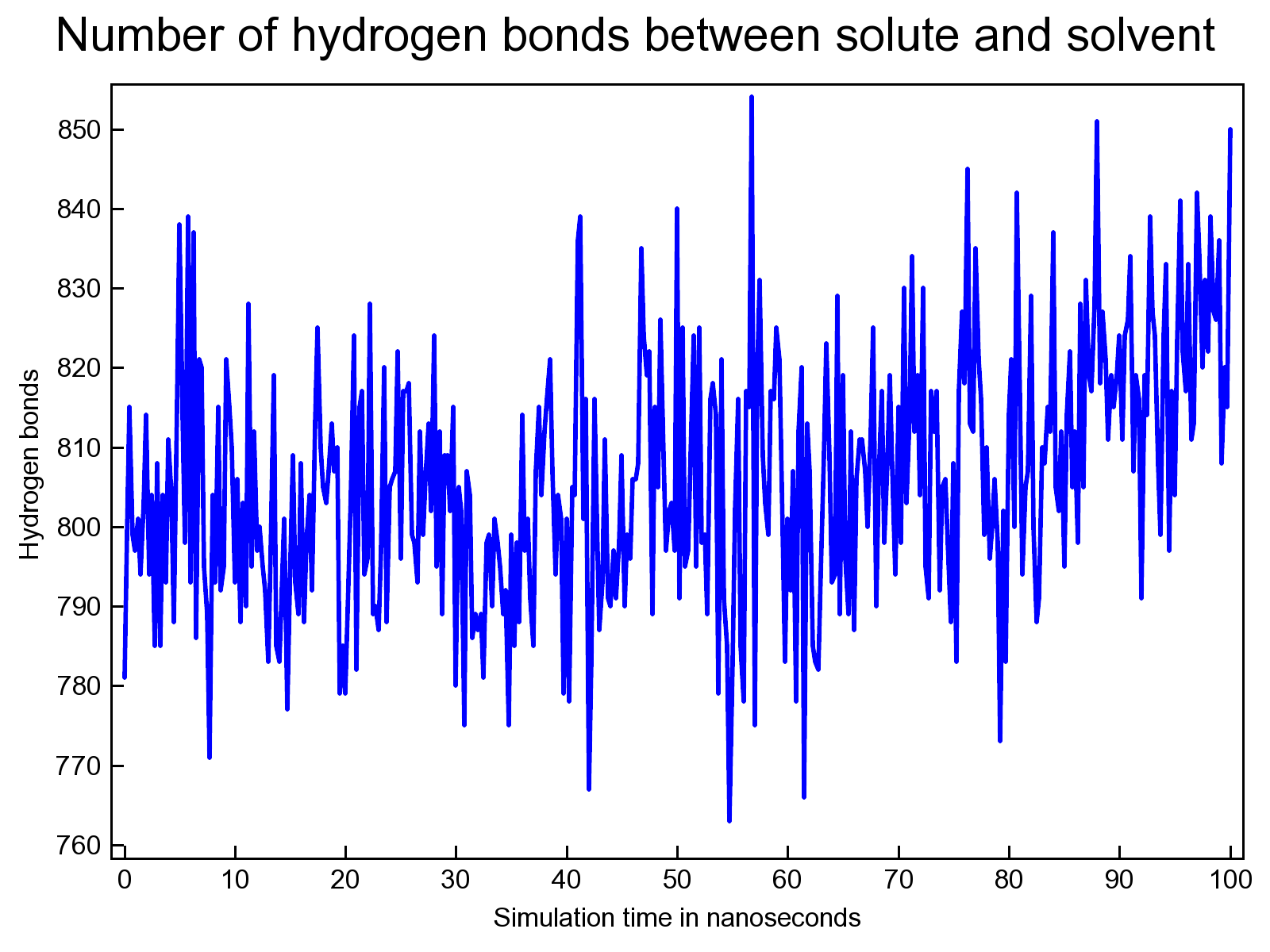

Supplement: S7 File — (ZIP) [file pone.0338211.s007.zip › S6.Molecular Dynamic Simulation/S6.Molecular Dynamic Simulation/MDS result for Donepezil_hAChE/Donepezil & hAChE - Al amin Afendy_report_figure8_hires.png]

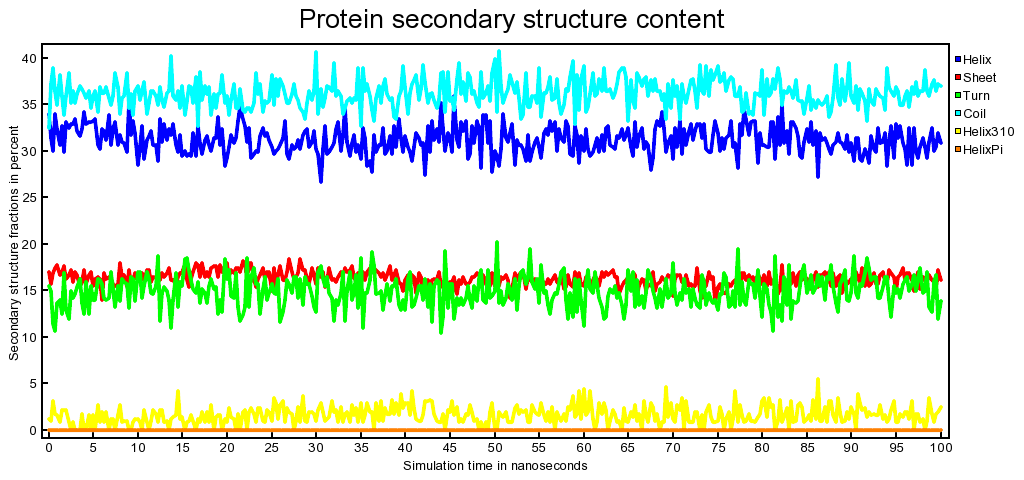

Supplement: S7 File — (ZIP) [file pone.0338211.s007.zip › S6.Molecular Dynamic Simulation/S6.Molecular Dynamic Simulation/MDS result for Donepezil_hAChE/Donepezil & hAChE - Al amin Afendy_report_figure9.png]

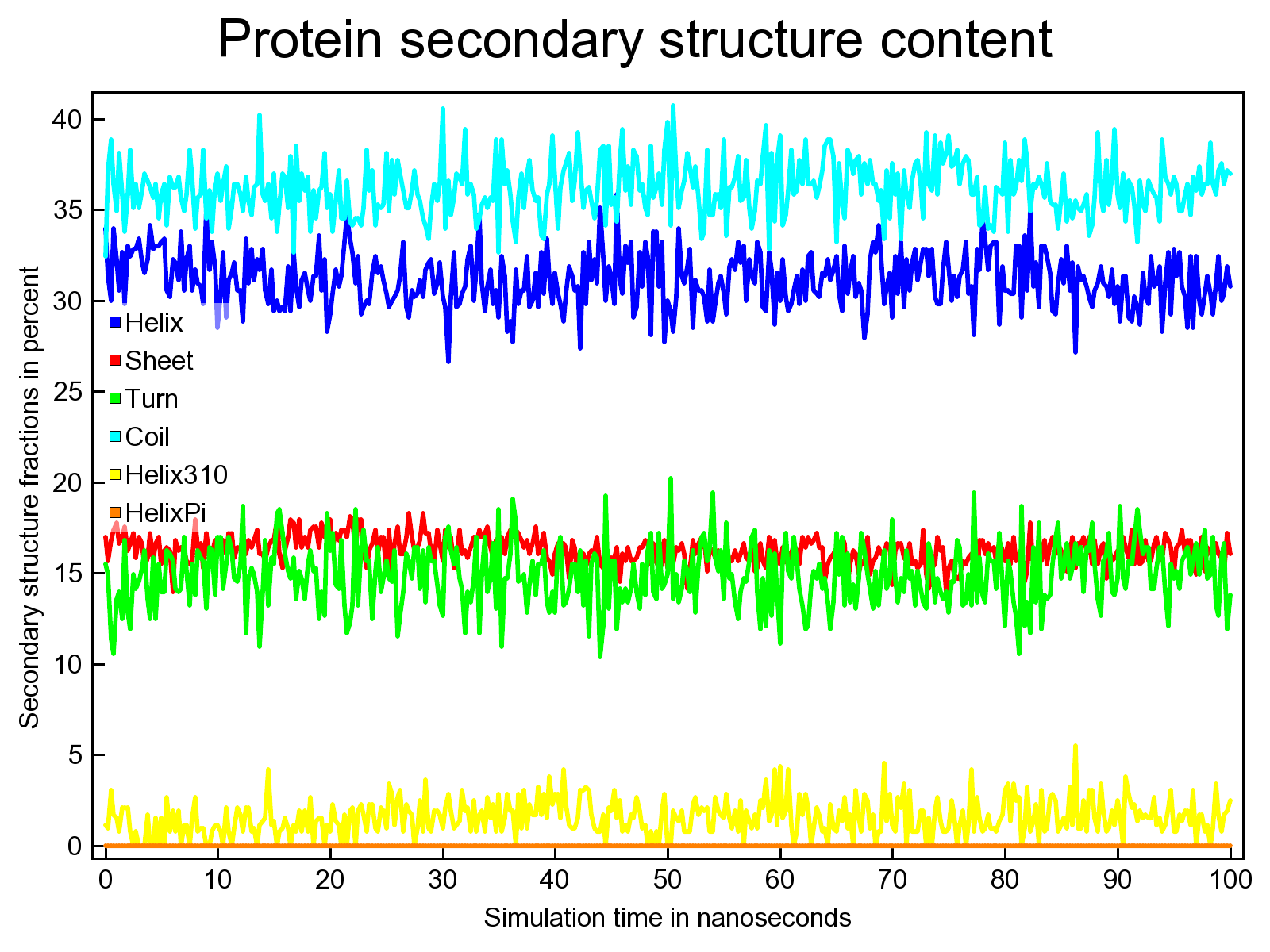

Supplement: S7 File — (ZIP) [file pone.0338211.s007.zip › S6.Molecular Dynamic Simulation/S6.Molecular Dynamic Simulation/MDS result for Donepezil_hAChE/Donepezil & hAChE - Al amin Afendy_report_figure9_hires.png]

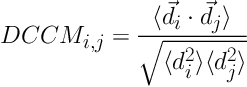

Supplement: S7 File — (ZIP) [file pone.0338211.s007.zip › S6.Molecular Dynamic Simulation/S6.Molecular Dynamic Simulation/MDS result for Donepezil_hAChE/Donepezil & hAChE - Al amin Afendy_report_formula_dccm.png]

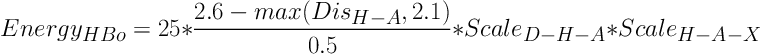

Supplement: S7 File — (ZIP) [file pone.0338211.s007.zip › S6.Molecular Dynamic Simulation/S6.Molecular Dynamic Simulation/MDS result for Donepezil_hAChE/Donepezil & hAChE - Al amin Afendy_report_formula_energyhbo0.png]

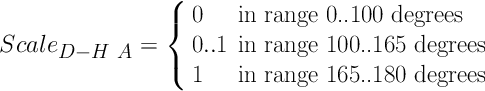

Supplement: S7 File — (ZIP) [file pone.0338211.s007.zip › S6.Molecular Dynamic Simulation/S6.Molecular Dynamic Simulation/MDS result for Donepezil_hAChE/Donepezil & hAChE - Al amin Afendy_report_formula_energyhbo1.png]

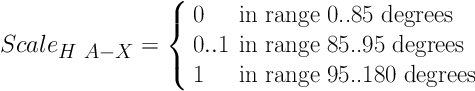

Supplement: S7 File — (ZIP) [file pone.0338211.s007.zip › S6.Molecular Dynamic Simulation/S6.Molecular Dynamic Simulation/MDS result for Donepezil_hAChE/Donepezil & hAChE - Al amin Afendy_report_formula_energyhbo2.png]

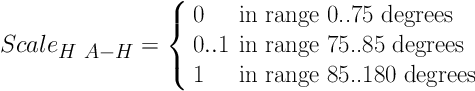

Supplement: S7 File — (ZIP) [file pone.0338211.s007.zip › S6.Molecular Dynamic Simulation/S6.Molecular Dynamic Simulation/MDS result for Donepezil_hAChE/Donepezil & hAChE - Al amin Afendy_report_formula_energyhbo3.png]

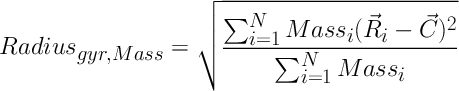

Supplement: S7 File — (ZIP) [file pone.0338211.s007.zip › S6.Molecular Dynamic Simulation/S6.Molecular Dynamic Simulation/MDS result for Donepezil_hAChE/Donepezil & hAChE - Al amin Afendy_report_formula_gyrrad.png]

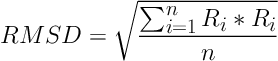

Supplement: S7 File — (ZIP) [file pone.0338211.s007.zip › S6.Molecular Dynamic Simulation/S6.Molecular Dynamic Simulation/MDS result for Donepezil_hAChE/Donepezil & hAChE - Al amin Afendy_report_formula_rmsd.png]

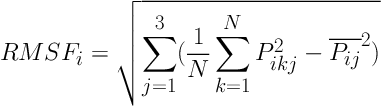

Supplement: S7 File — (ZIP) [file pone.0338211.s007.zip › S6.Molecular Dynamic Simulation/S6.Molecular Dynamic Simulation/MDS result for Donepezil_hAChE/Donepezil & hAChE - Al amin Afendy_report_formula_rmsf.png]

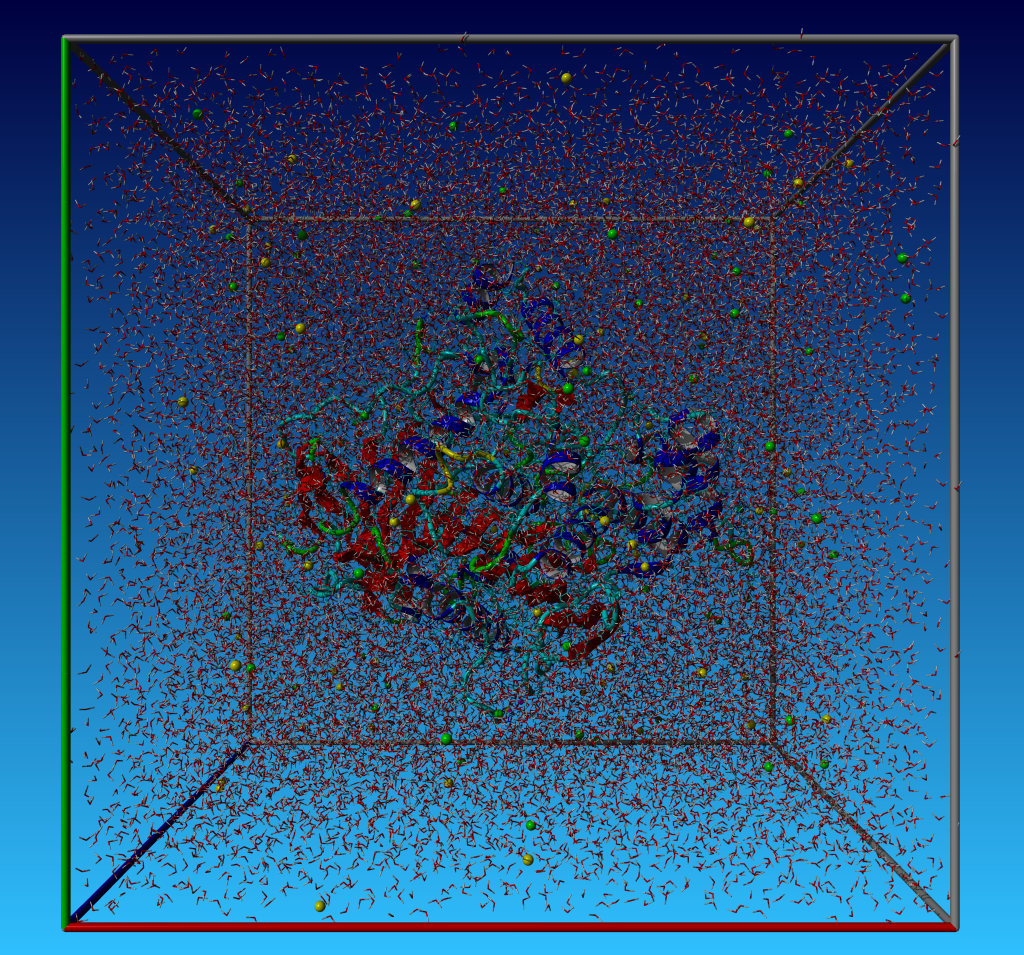

Supplement: S7 File — (ZIP) [file pone.0338211.s007.zip › S6.Molecular Dynamic Simulation/S6.Molecular Dynamic Simulation/Result napitane+roluperidone/Result 2/Complex one/Complex one_report_figure1.png]

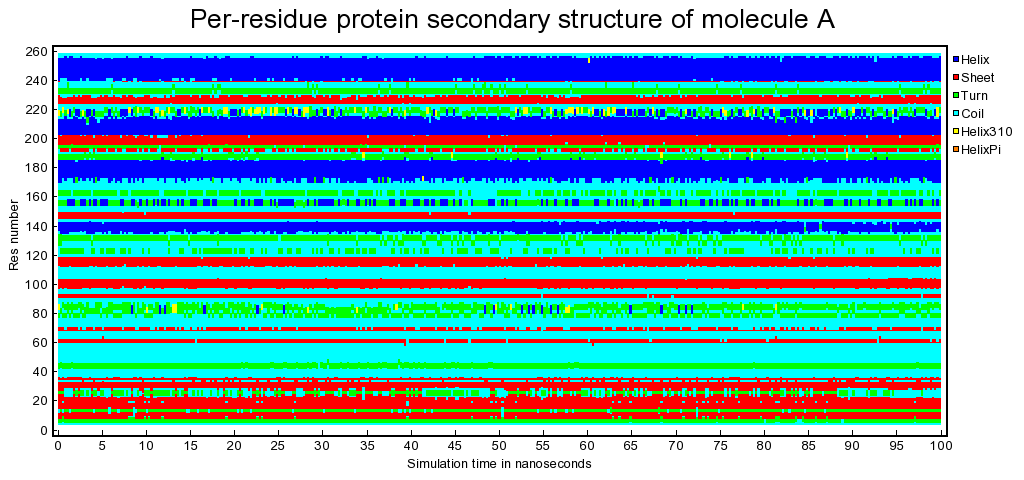

Supplement: S7 File — (ZIP) [file pone.0338211.s007.zip › S6.Molecular Dynamic Simulation/S6.Molecular Dynamic Simulation/Result napitane+roluperidone/Result 2/Complex one/Complex one_report_figure10.png]

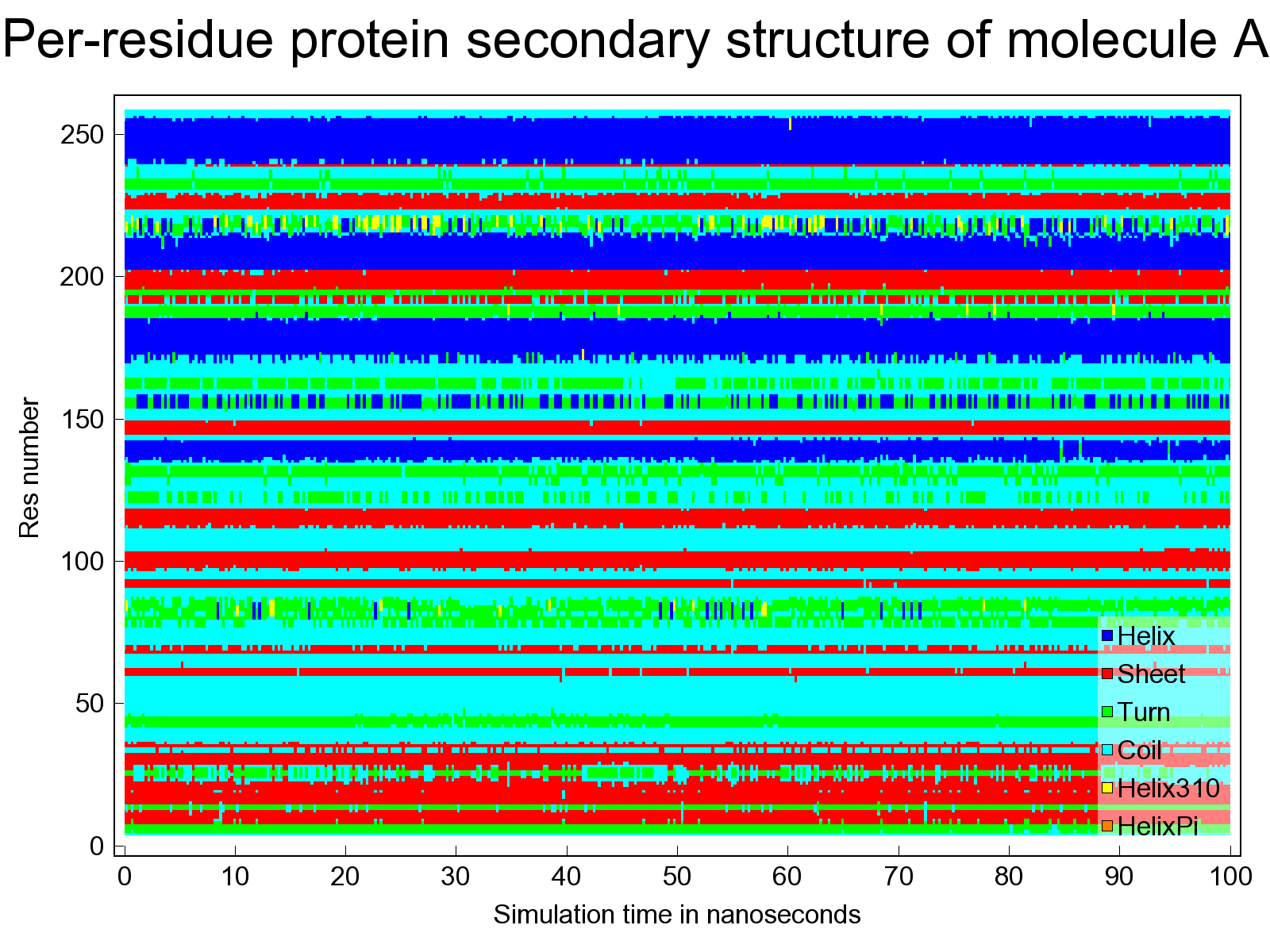

Supplement: S7 File — (ZIP) [file pone.0338211.s007.zip › S6.Molecular Dynamic Simulation/S6.Molecular Dynamic Simulation/Result napitane+roluperidone/Result 2/Complex one/Complex one_report_figure10_hires.png]

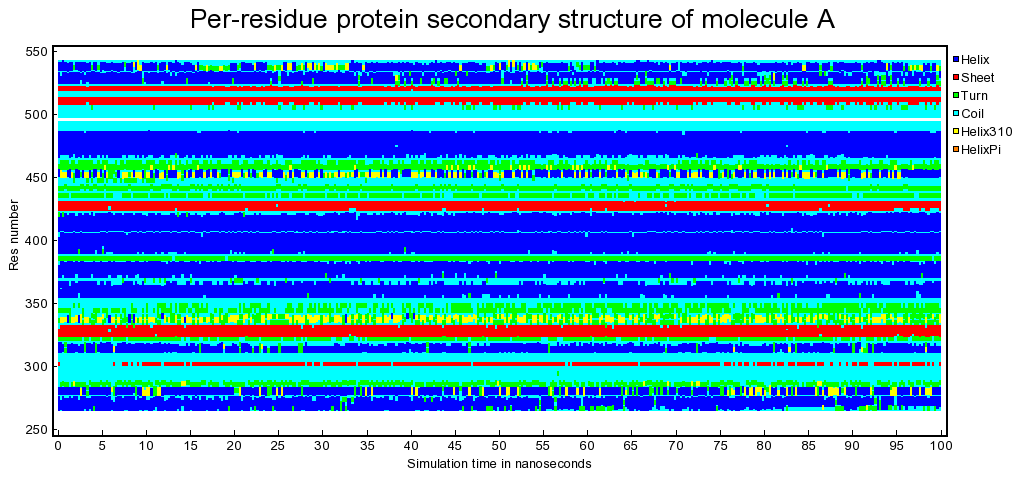

Supplement: S7 File — (ZIP) [file pone.0338211.s007.zip › S6.Molecular Dynamic Simulation/S6.Molecular Dynamic Simulation/Result napitane+roluperidone/Result 2/Complex one/Complex one_report_figure11.png]

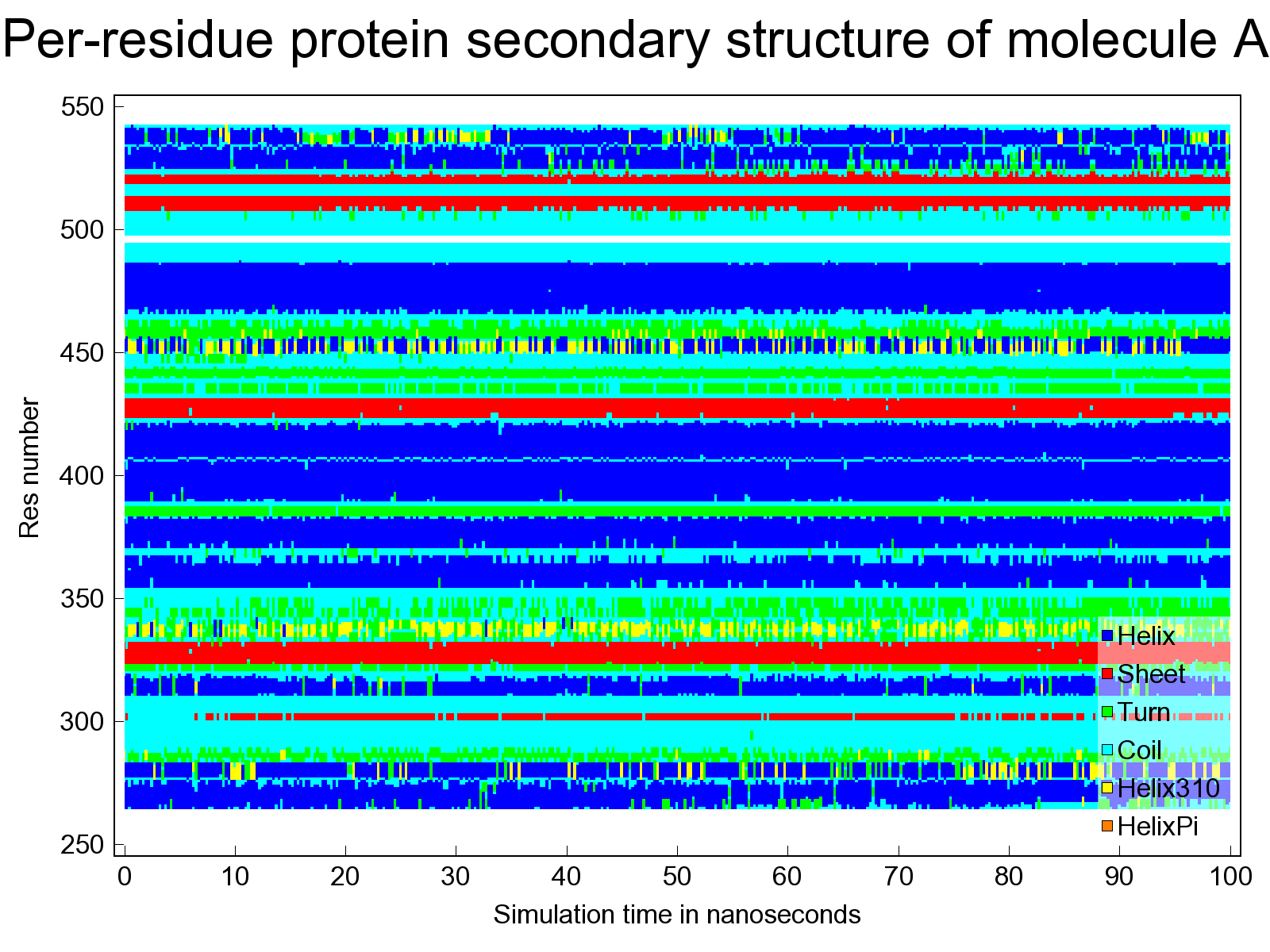

Supplement: S7 File — (ZIP) [file pone.0338211.s007.zip › S6.Molecular Dynamic Simulation/S6.Molecular Dynamic Simulation/Result napitane+roluperidone/Result 2/Complex one/Complex one_report_figure11_hires.png]

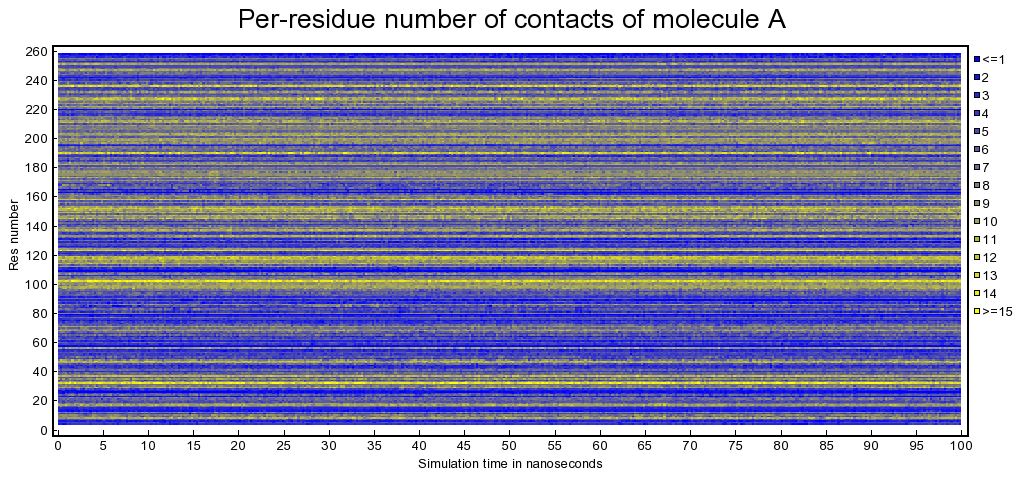

Supplement: S7 File — (ZIP) [file pone.0338211.s007.zip › S6.Molecular Dynamic Simulation/S6.Molecular Dynamic Simulation/Result napitane+roluperidone/Result 2/Complex one/Complex one_report_figure12.png]

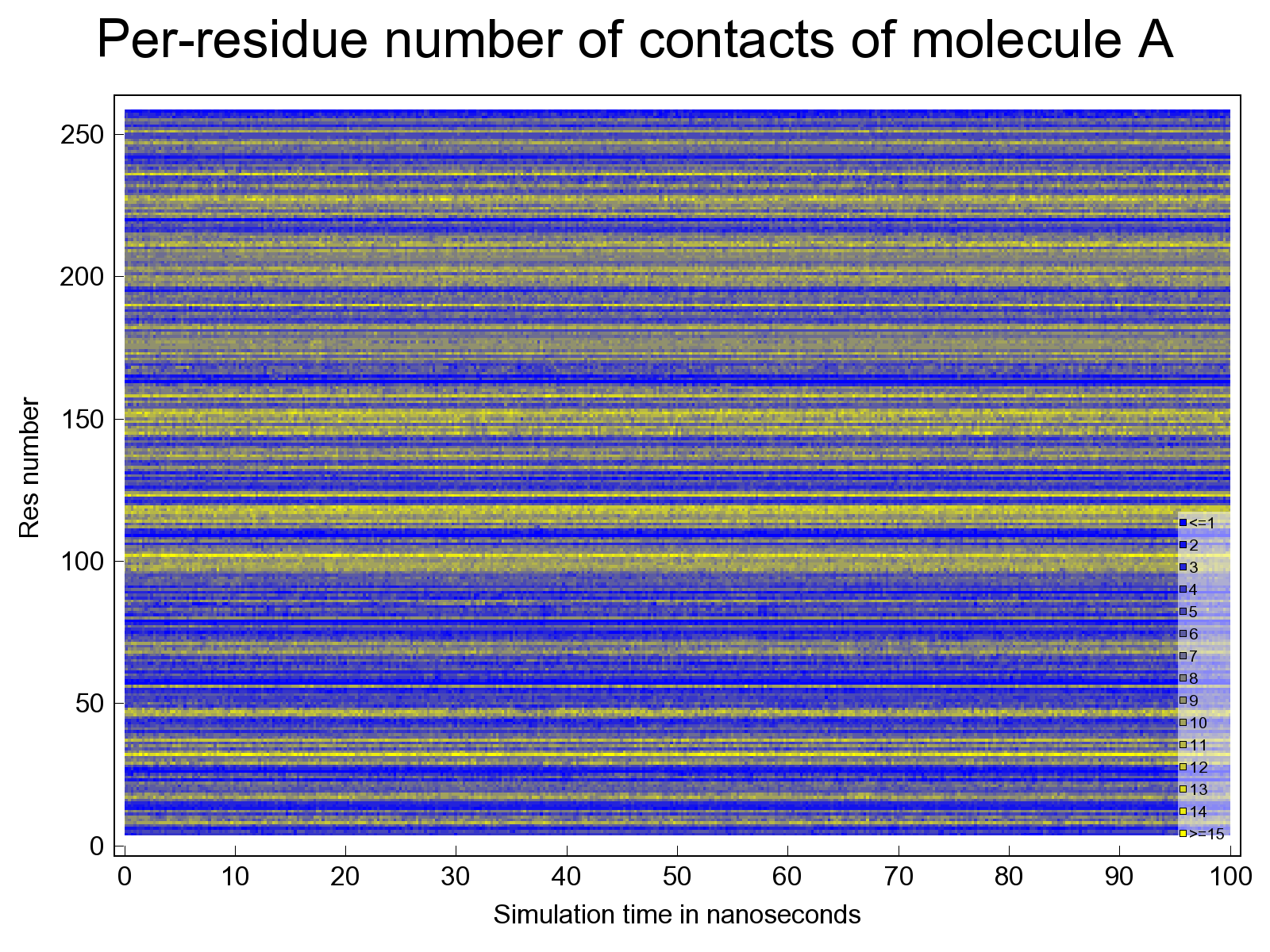

Supplement: S7 File — (ZIP) [file pone.0338211.s007.zip › S6.Molecular Dynamic Simulation/S6.Molecular Dynamic Simulation/Result napitane+roluperidone/Result 2/Complex one/Complex one_report_figure12_hires.png]

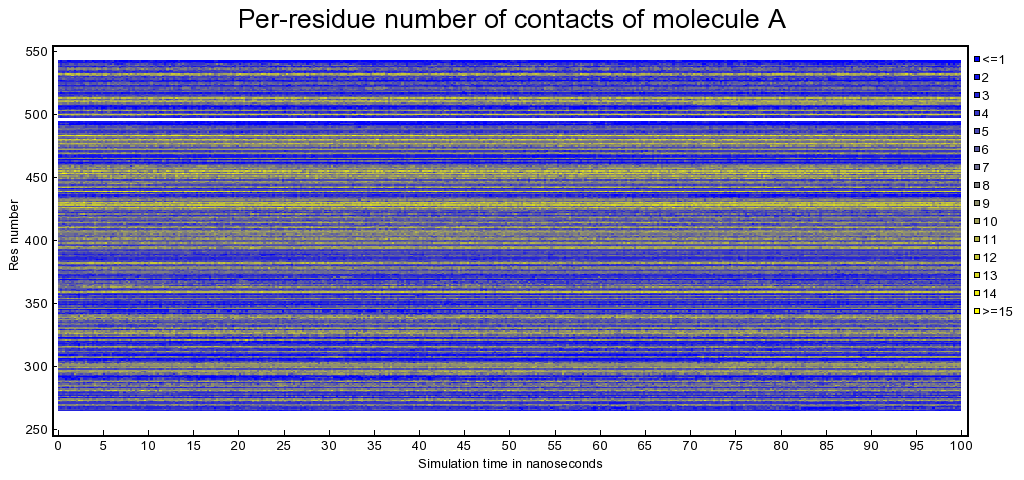

Supplement: S7 File — (ZIP) [file pone.0338211.s007.zip › S6.Molecular Dynamic Simulation/S6.Molecular Dynamic Simulation/Result napitane+roluperidone/Result 2/Complex one/Complex one_report_figure13.png]

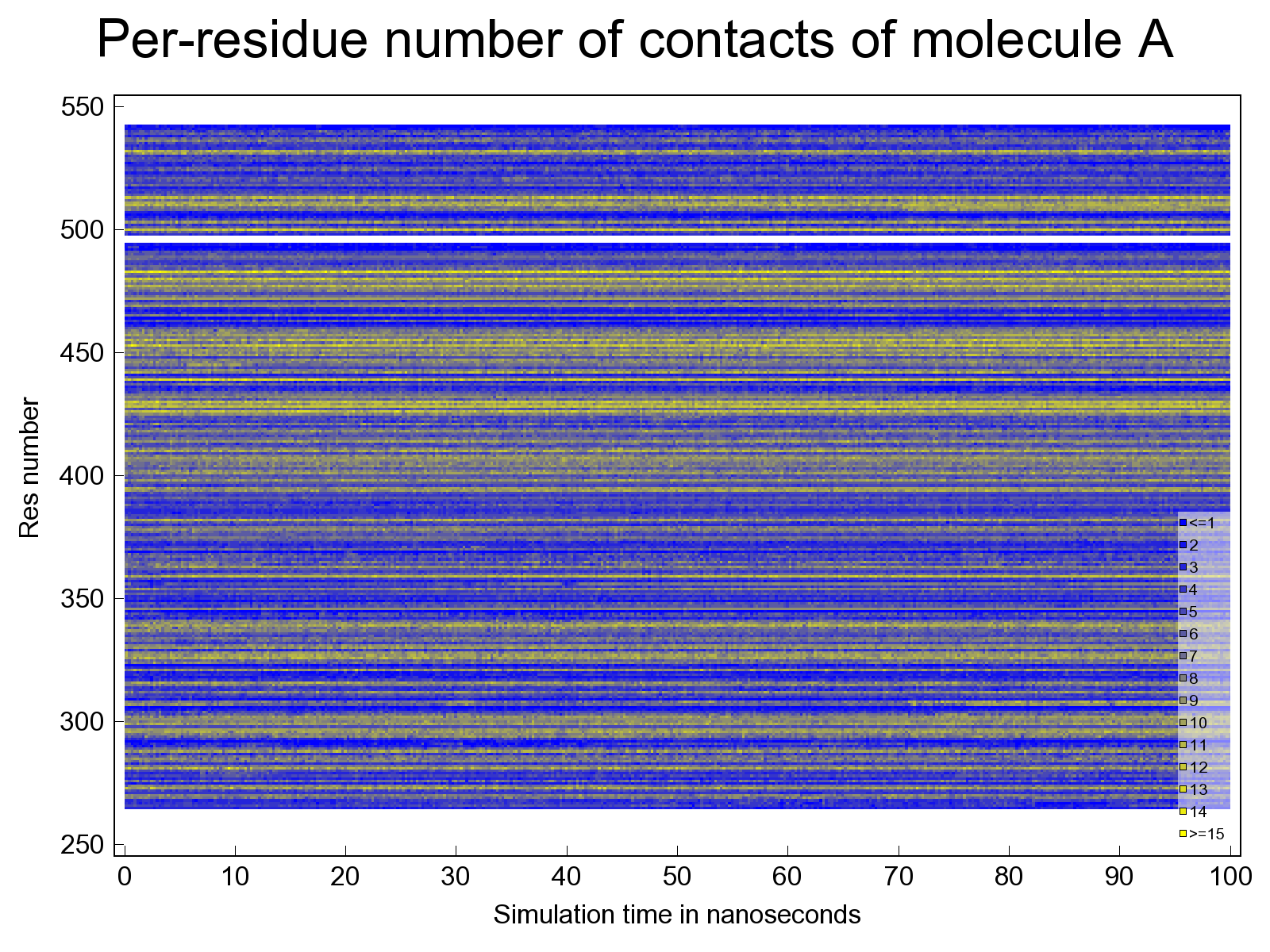

Supplement: S7 File — (ZIP) [file pone.0338211.s007.zip › S6.Molecular Dynamic Simulation/S6.Molecular Dynamic Simulation/Result napitane+roluperidone/Result 2/Complex one/Complex one_report_figure13_hires.png]

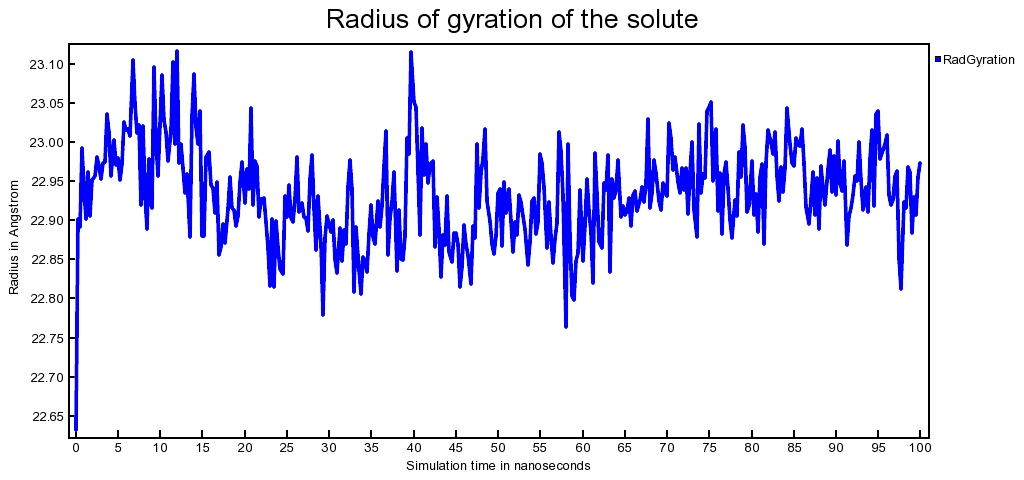

Supplement: S7 File — (ZIP) [file pone.0338211.s007.zip › S6.Molecular Dynamic Simulation/S6.Molecular Dynamic Simulation/Result napitane+roluperidone/Result 2/Complex one/Complex one_report_figure14.png]

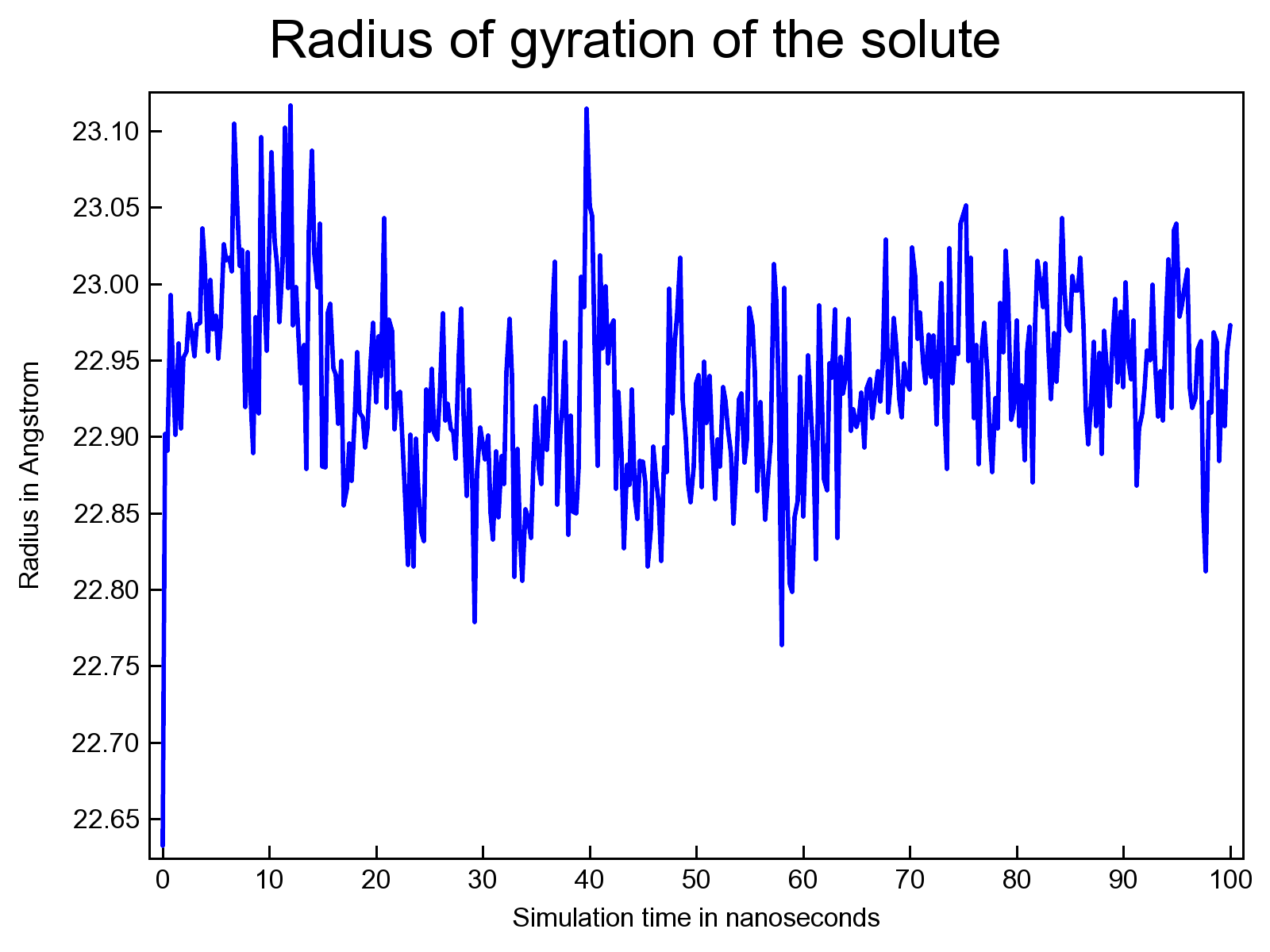

Supplement: S7 File — (ZIP) [file pone.0338211.s007.zip › S6.Molecular Dynamic Simulation/S6.Molecular Dynamic Simulation/Result napitane+roluperidone/Result 2/Complex one/Complex one_report_figure14_hires.png]

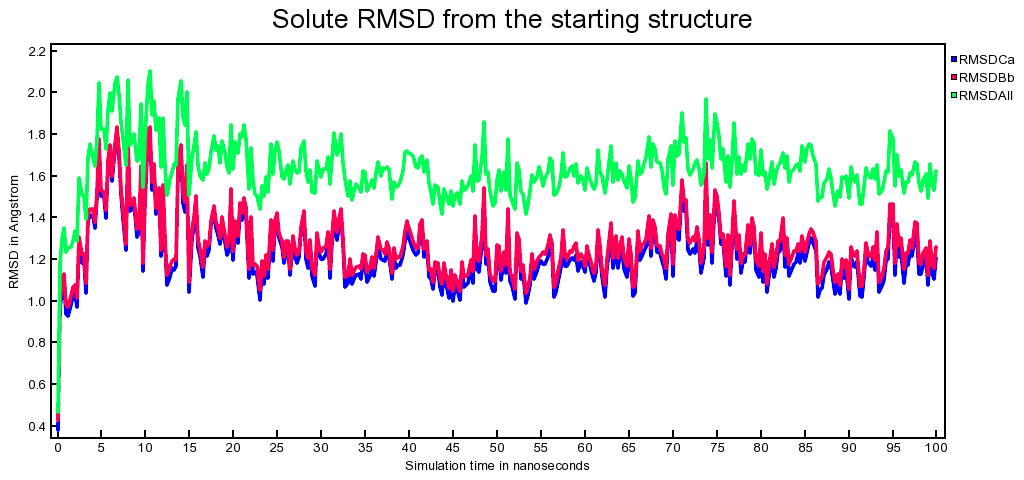

Supplement: S7 File — (ZIP) [file pone.0338211.s007.zip › S6.Molecular Dynamic Simulation/S6.Molecular Dynamic Simulation/Result napitane+roluperidone/Result 2/Complex one/Complex one_report_figure15.png]

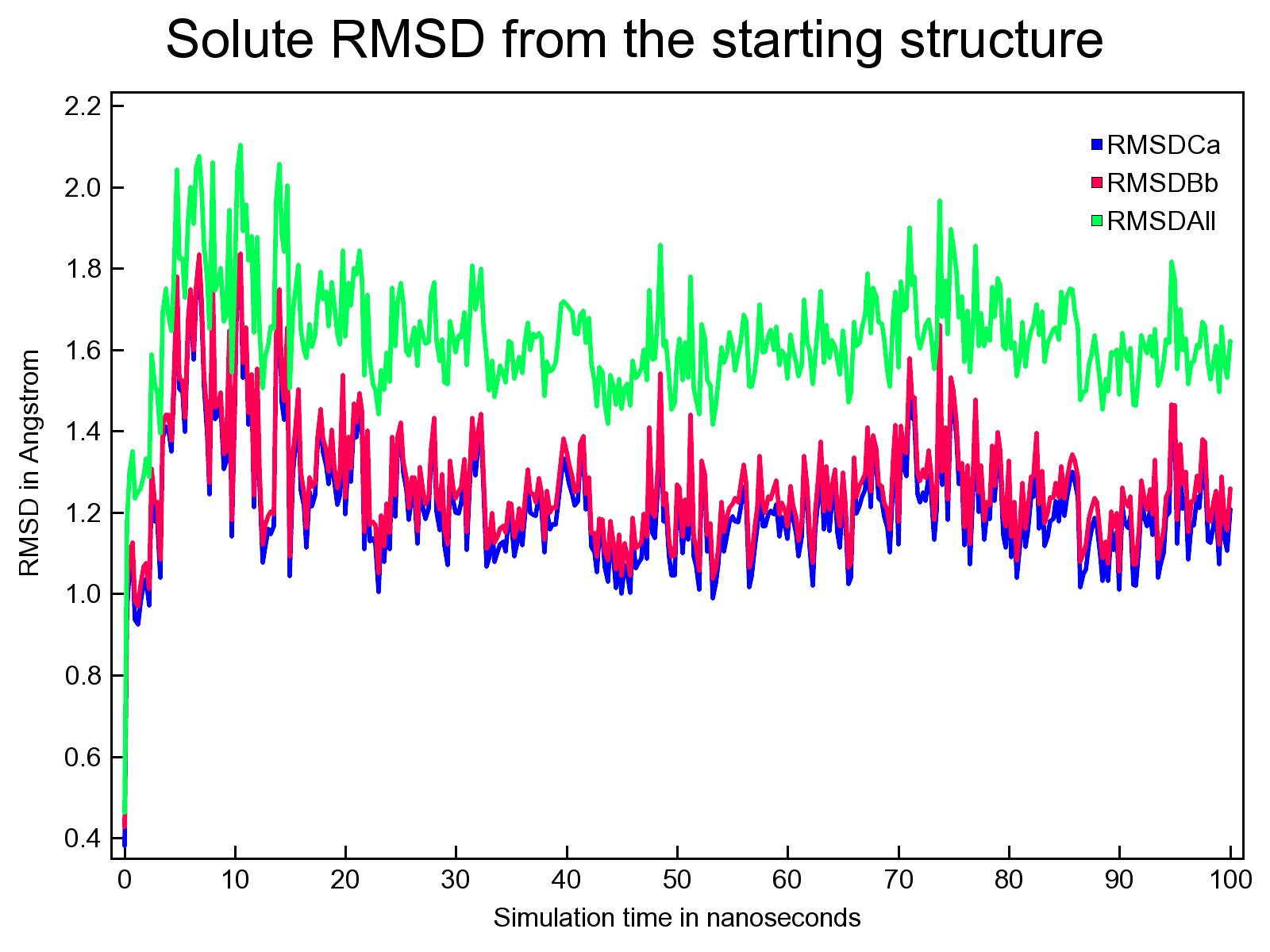

Supplement: S7 File — (ZIP) [file pone.0338211.s007.zip › S6.Molecular Dynamic Simulation/S6.Molecular Dynamic Simulation/Result napitane+roluperidone/Result 2/Complex one/Complex one_report_figure15_hires.png]

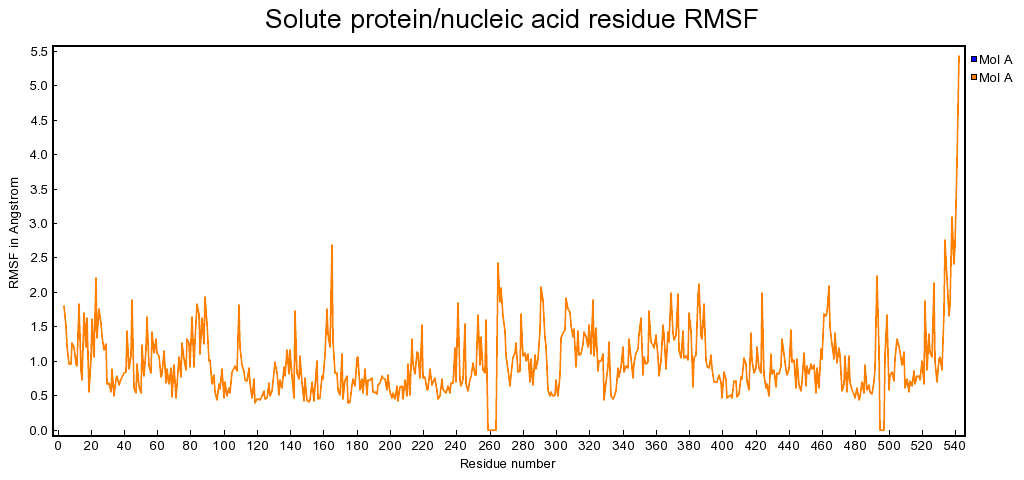

Supplement: S7 File — (ZIP) [file pone.0338211.s007.zip › S6.Molecular Dynamic Simulation/S6.Molecular Dynamic Simulation/Result napitane+roluperidone/Result 2/Complex one/Complex one_report_figure16.png]

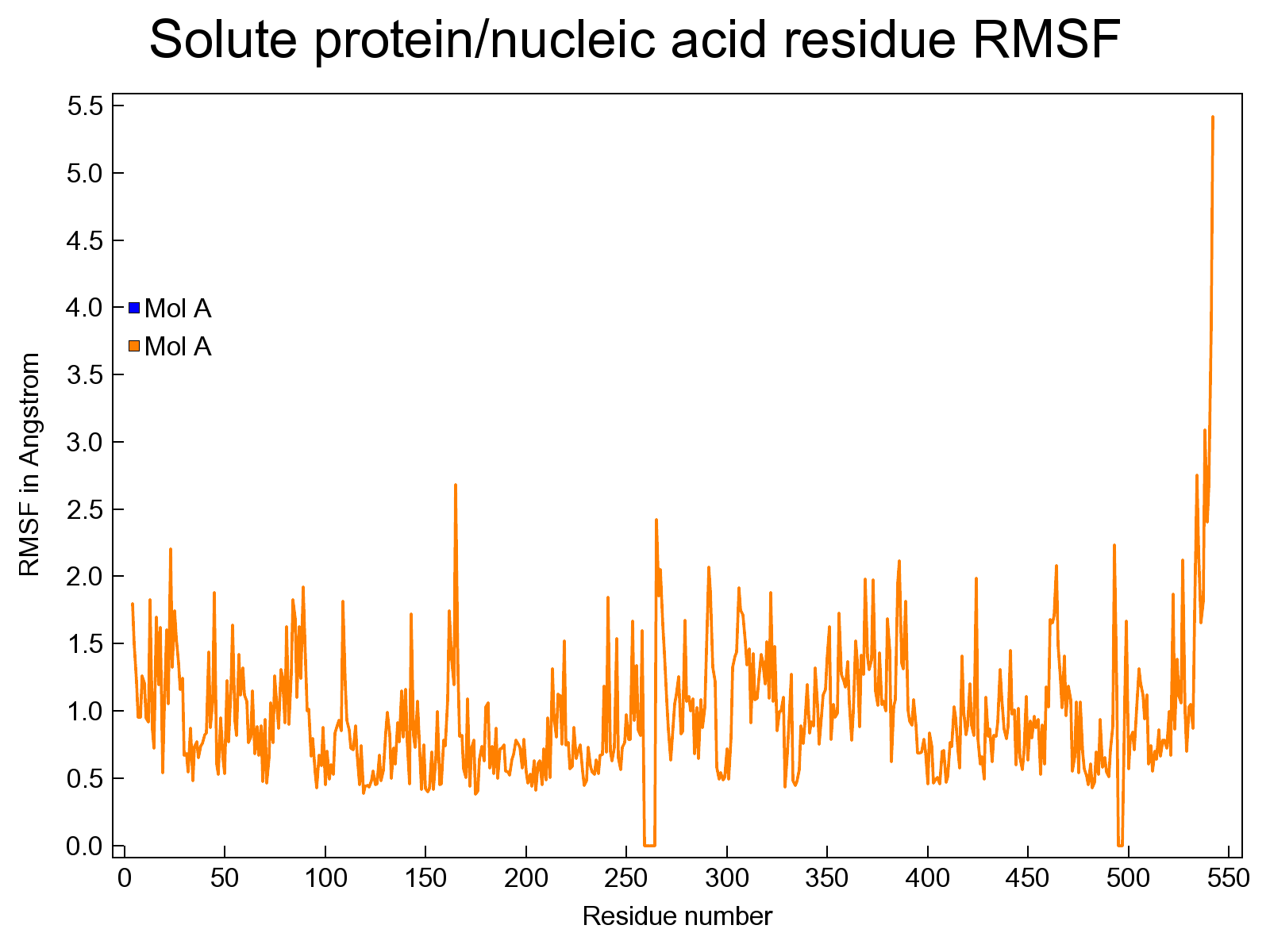

Supplement: S7 File — (ZIP) [file pone.0338211.s007.zip › S6.Molecular Dynamic Simulation/S6.Molecular Dynamic Simulation/Result napitane+roluperidone/Result 2/Complex one/Complex one_report_figure16_hires.png]

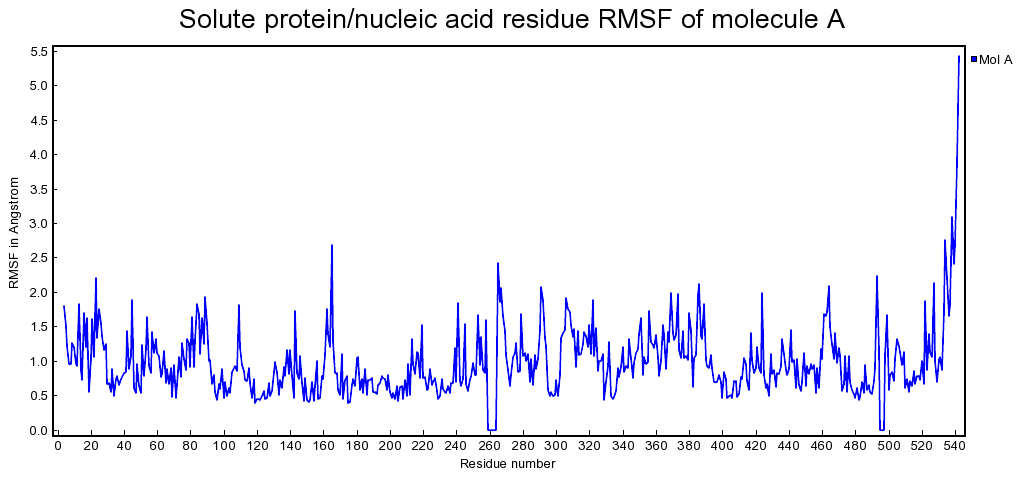

Supplement: S7 File — (ZIP) [file pone.0338211.s007.zip › S6.Molecular Dynamic Simulation/S6.Molecular Dynamic Simulation/Result napitane+roluperidone/Result 2/Complex one/Complex one_report_figure17.png]

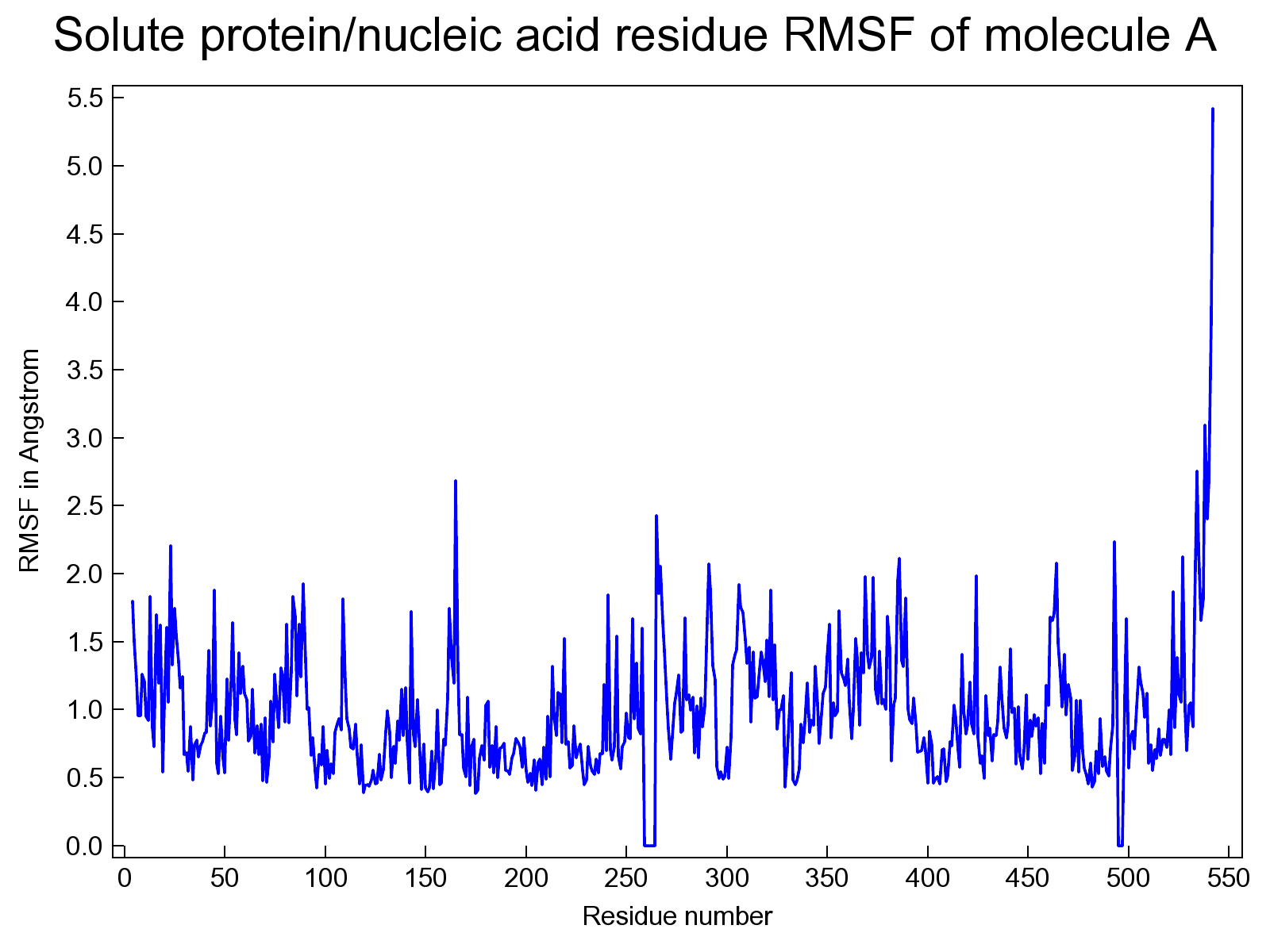

Supplement: S7 File — (ZIP) [file pone.0338211.s007.zip › S6.Molecular Dynamic Simulation/S6.Molecular Dynamic Simulation/Result napitane+roluperidone/Result 2/Complex one/Complex one_report_figure17_hires.png]

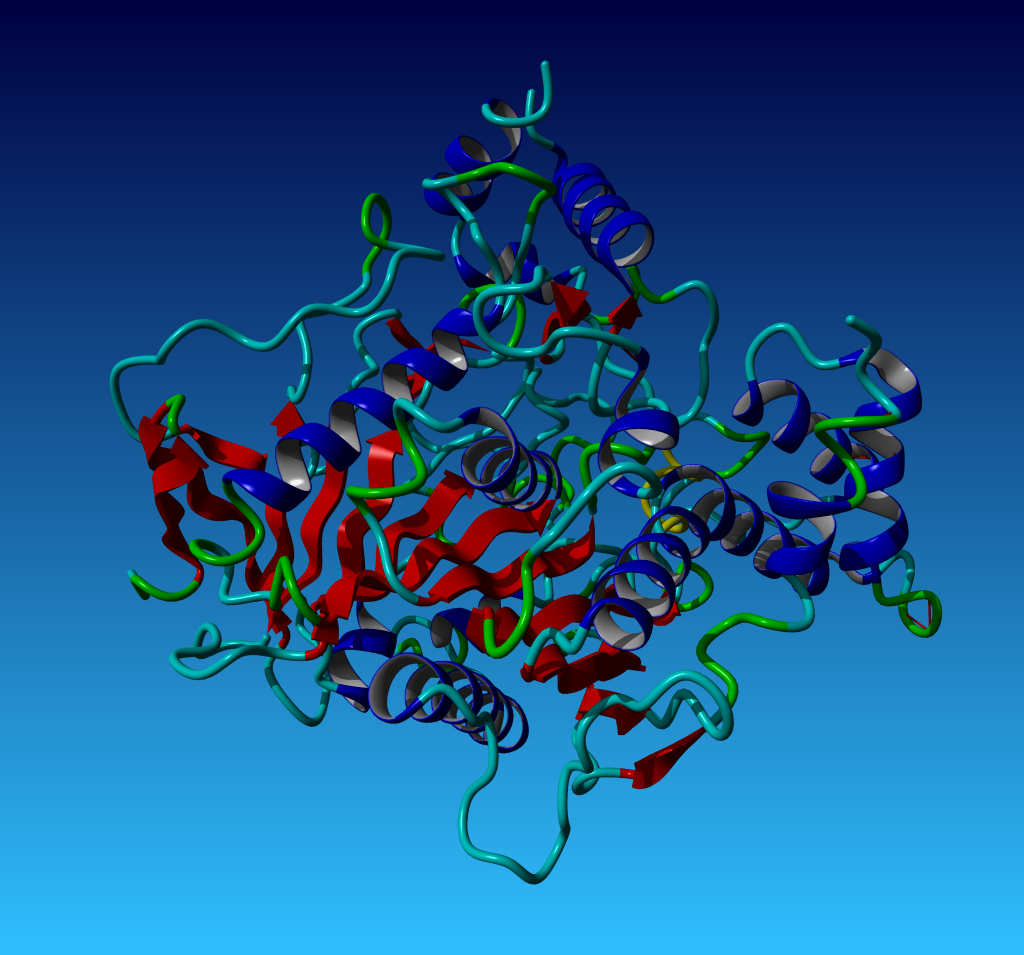

Supplement: S7 File — (ZIP) [file pone.0338211.s007.zip › S6.Molecular Dynamic Simulation/S6.Molecular Dynamic Simulation/Result napitane+roluperidone/Result 2/Complex one/Complex one_report_figure19.png]

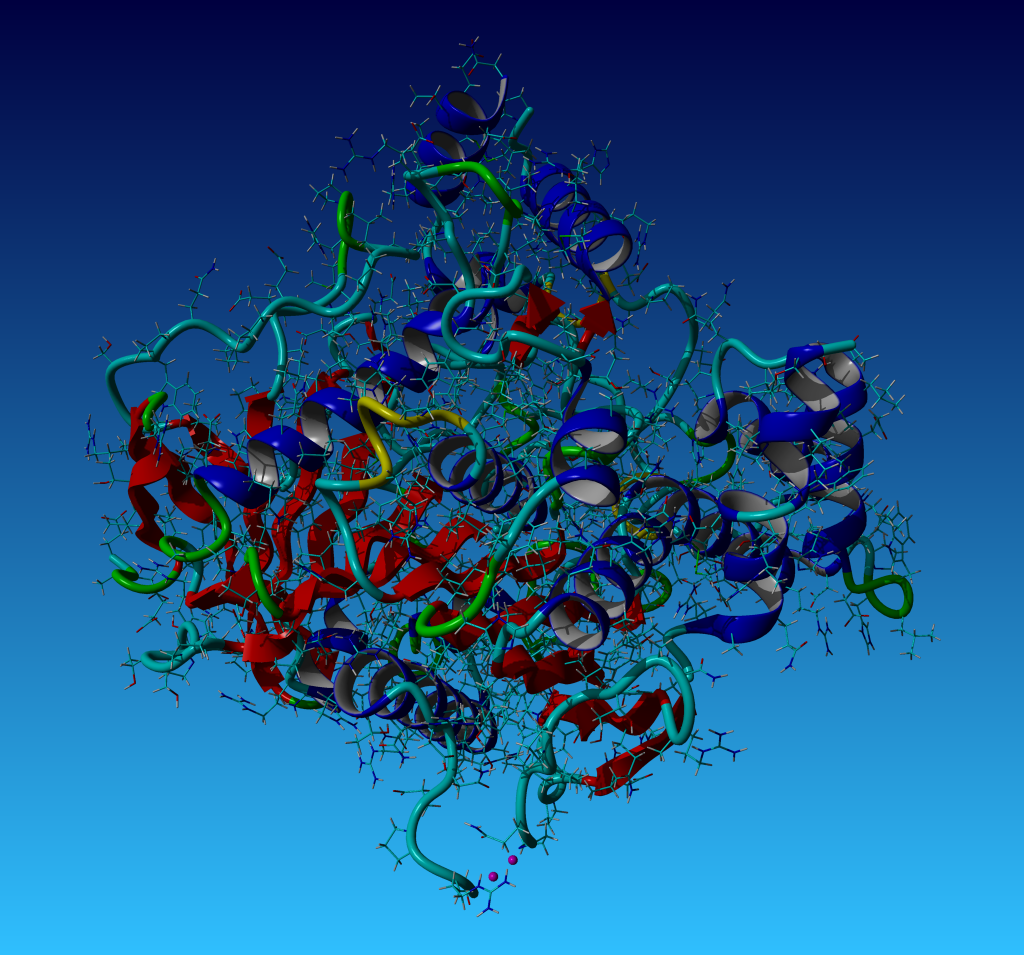

Supplement: S7 File — (ZIP) [file pone.0338211.s007.zip › S6.Molecular Dynamic Simulation/S6.Molecular Dynamic Simulation/Result napitane+roluperidone/Result 2/Complex one/Complex one_report_figure2.png]

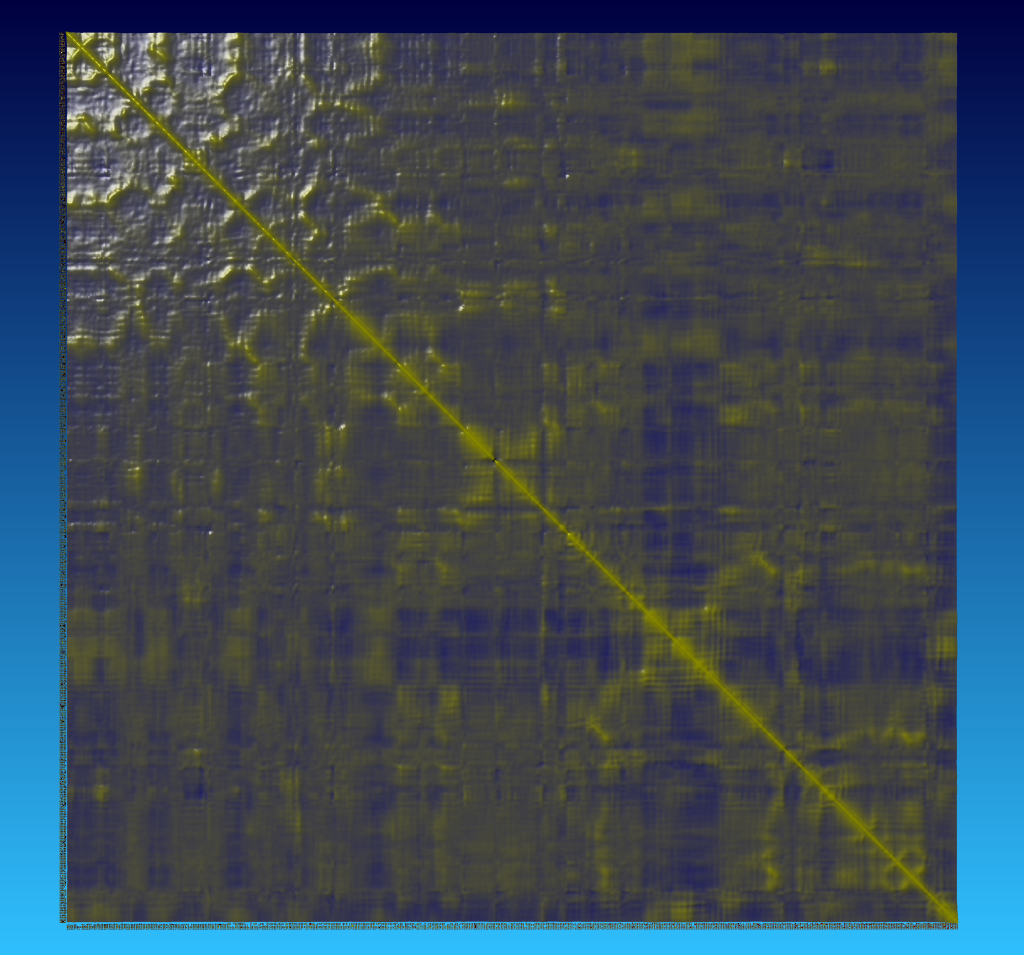

Supplement: S7 File — (ZIP) [file pone.0338211.s007.zip › S6.Molecular Dynamic Simulation/S6.Molecular Dynamic Simulation/Result napitane+roluperidone/Result 2/Complex one/Complex one_report_figure20.png]

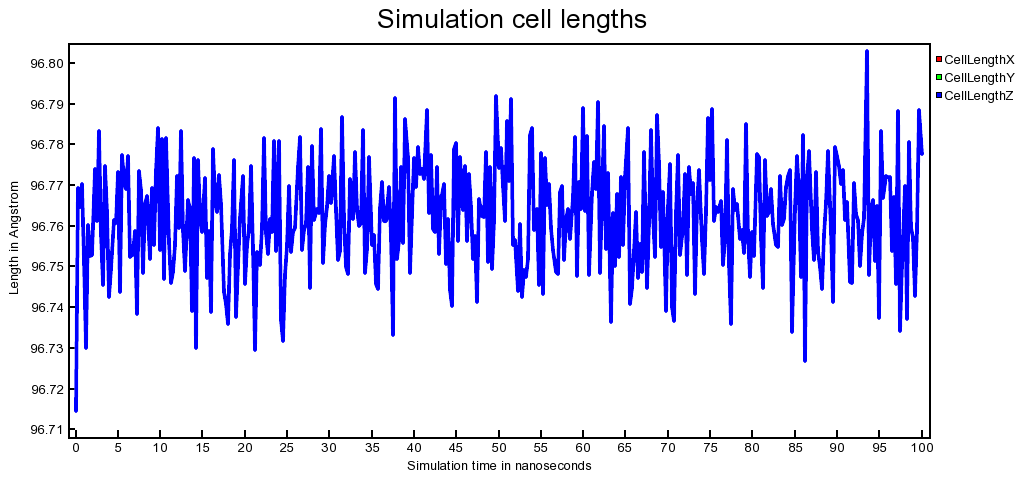

Supplement: S7 File — (ZIP) [file pone.0338211.s007.zip › S6.Molecular Dynamic Simulation/S6.Molecular Dynamic Simulation/Result napitane+roluperidone/Result 2/Complex one/Complex one_report_figure3.png]

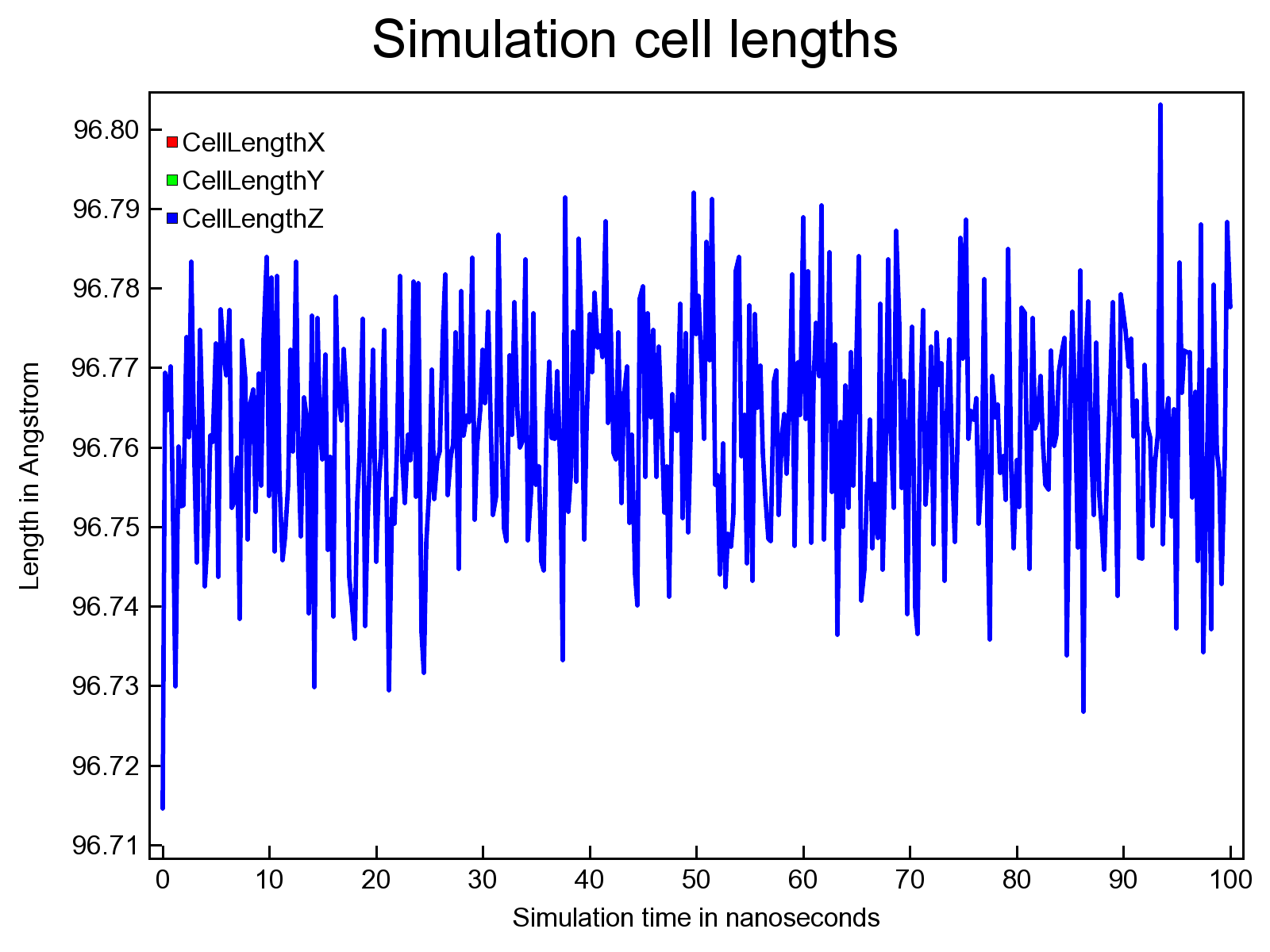

Supplement: S7 File — (ZIP) [file pone.0338211.s007.zip › S6.Molecular Dynamic Simulation/S6.Molecular Dynamic Simulation/Result napitane+roluperidone/Result 2/Complex one/Complex one_report_figure3_hires.png]

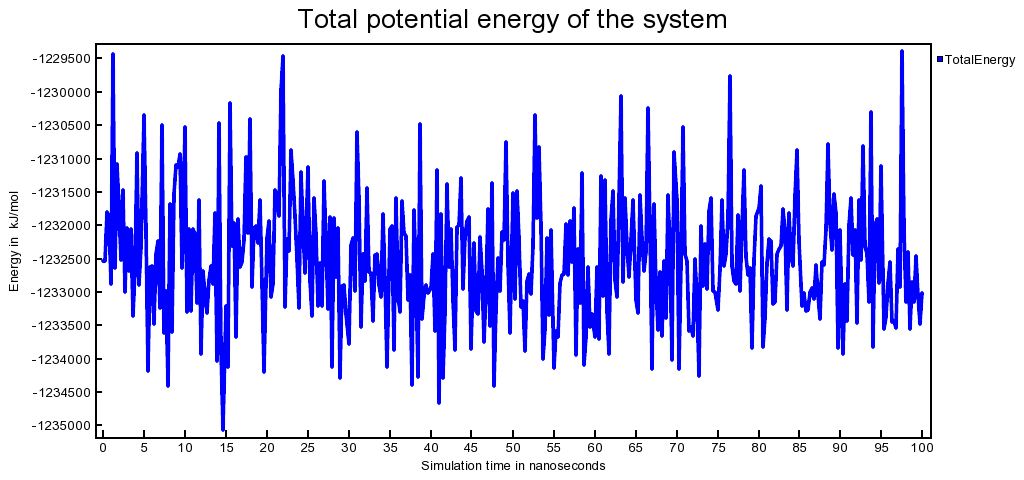

Supplement: S7 File — (ZIP) [file pone.0338211.s007.zip › S6.Molecular Dynamic Simulation/S6.Molecular Dynamic Simulation/Result napitane+roluperidone/Result 2/Complex one/Complex one_report_figure4.png]

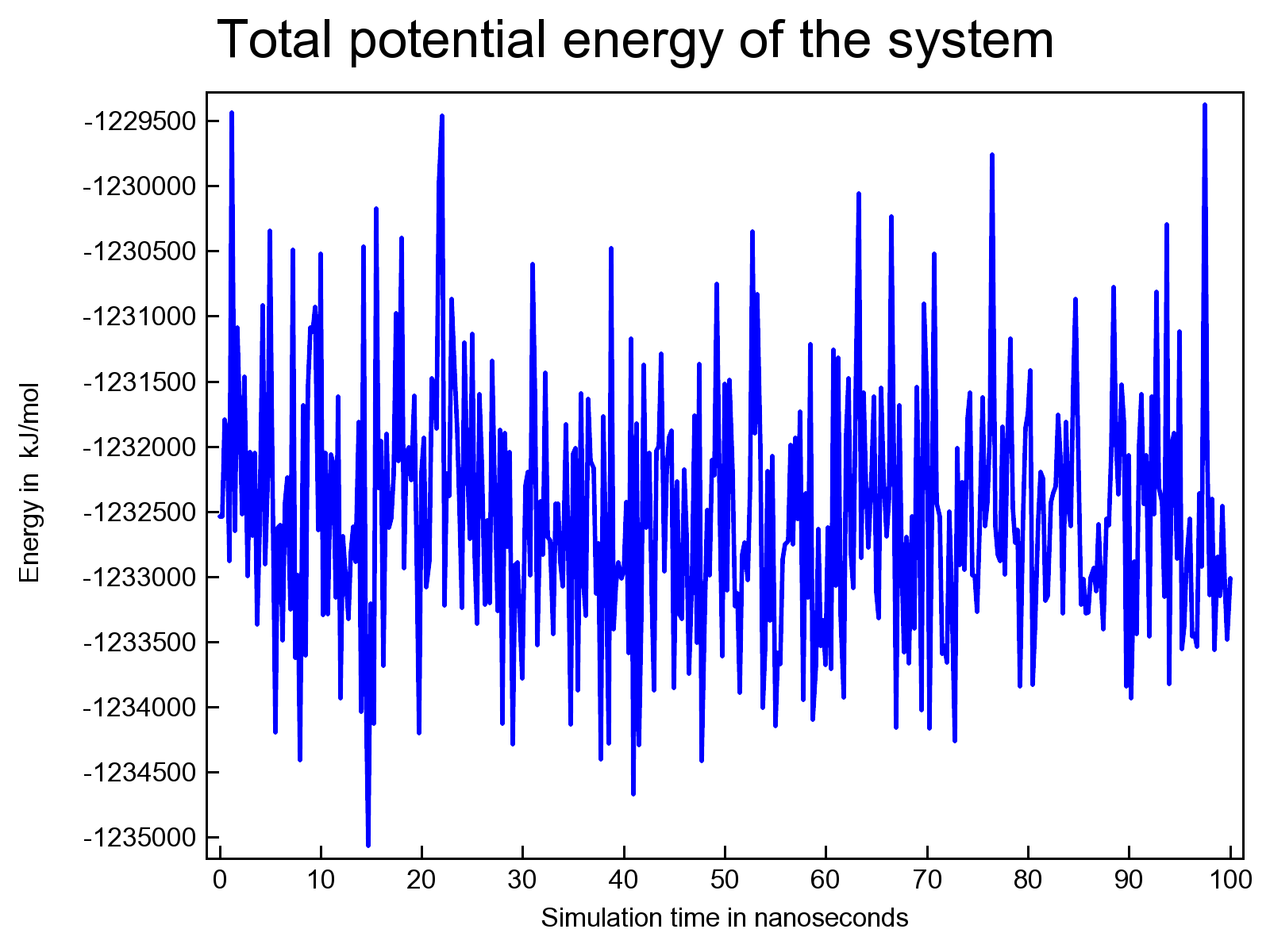

Supplement: S7 File — (ZIP) [file pone.0338211.s007.zip › S6.Molecular Dynamic Simulation/S6.Molecular Dynamic Simulation/Result napitane+roluperidone/Result 2/Complex one/Complex one_report_figure4_hires.png]

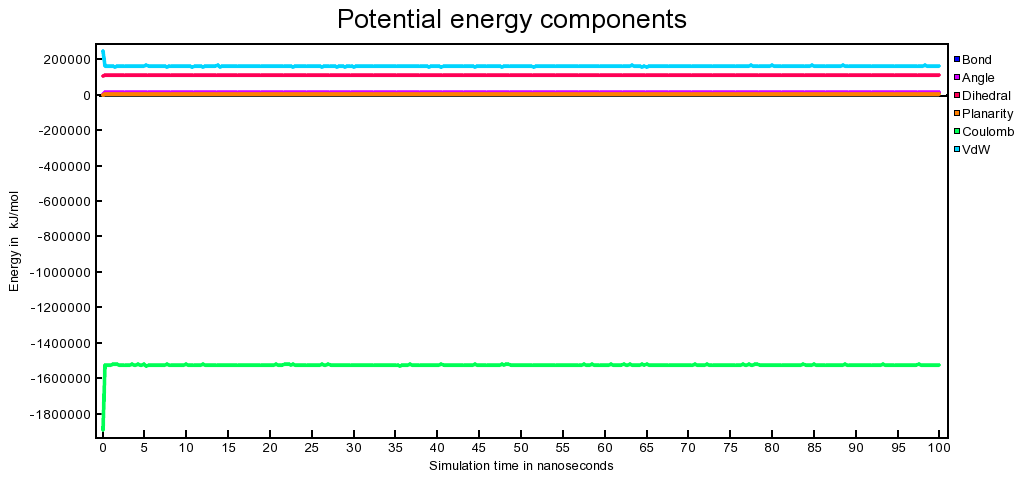

Supplement: S7 File — (ZIP) [file pone.0338211.s007.zip › S6.Molecular Dynamic Simulation/S6.Molecular Dynamic Simulation/Result napitane+roluperidone/Result 2/Complex one/Complex one_report_figure5.png]

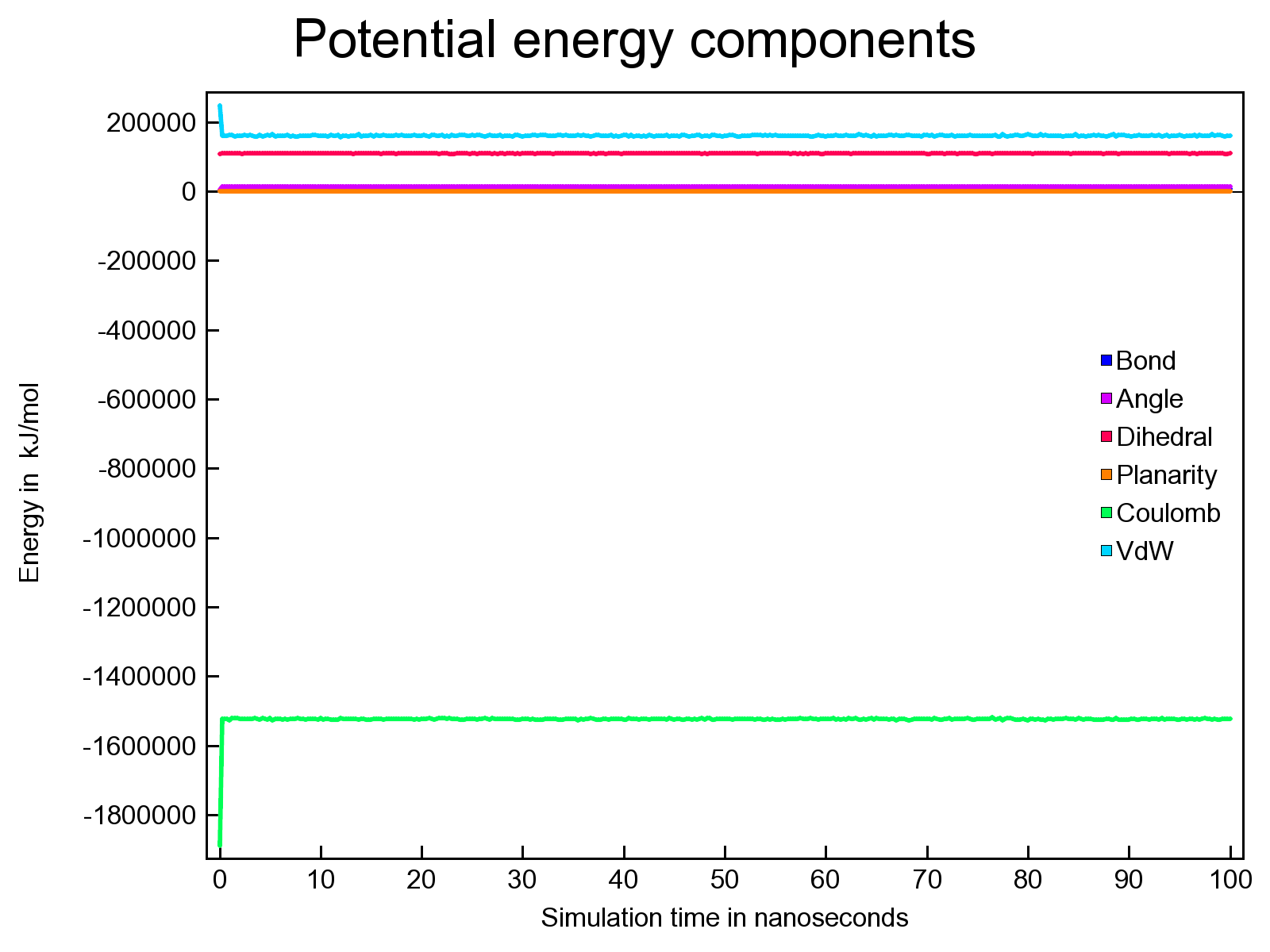

Supplement: S7 File — (ZIP) [file pone.0338211.s007.zip › S6.Molecular Dynamic Simulation/S6.Molecular Dynamic Simulation/Result napitane+roluperidone/Result 2/Complex one/Complex one_report_figure5_hires.png]

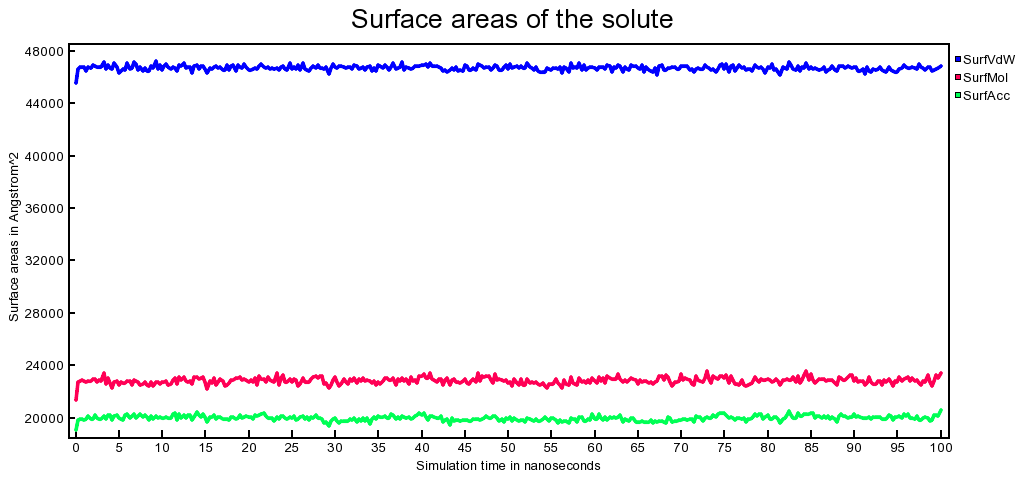

Supplement: S7 File — (ZIP) [file pone.0338211.s007.zip › S6.Molecular Dynamic Simulation/S6.Molecular Dynamic Simulation/Result napitane+roluperidone/Result 2/Complex one/Complex one_report_figure6.png]

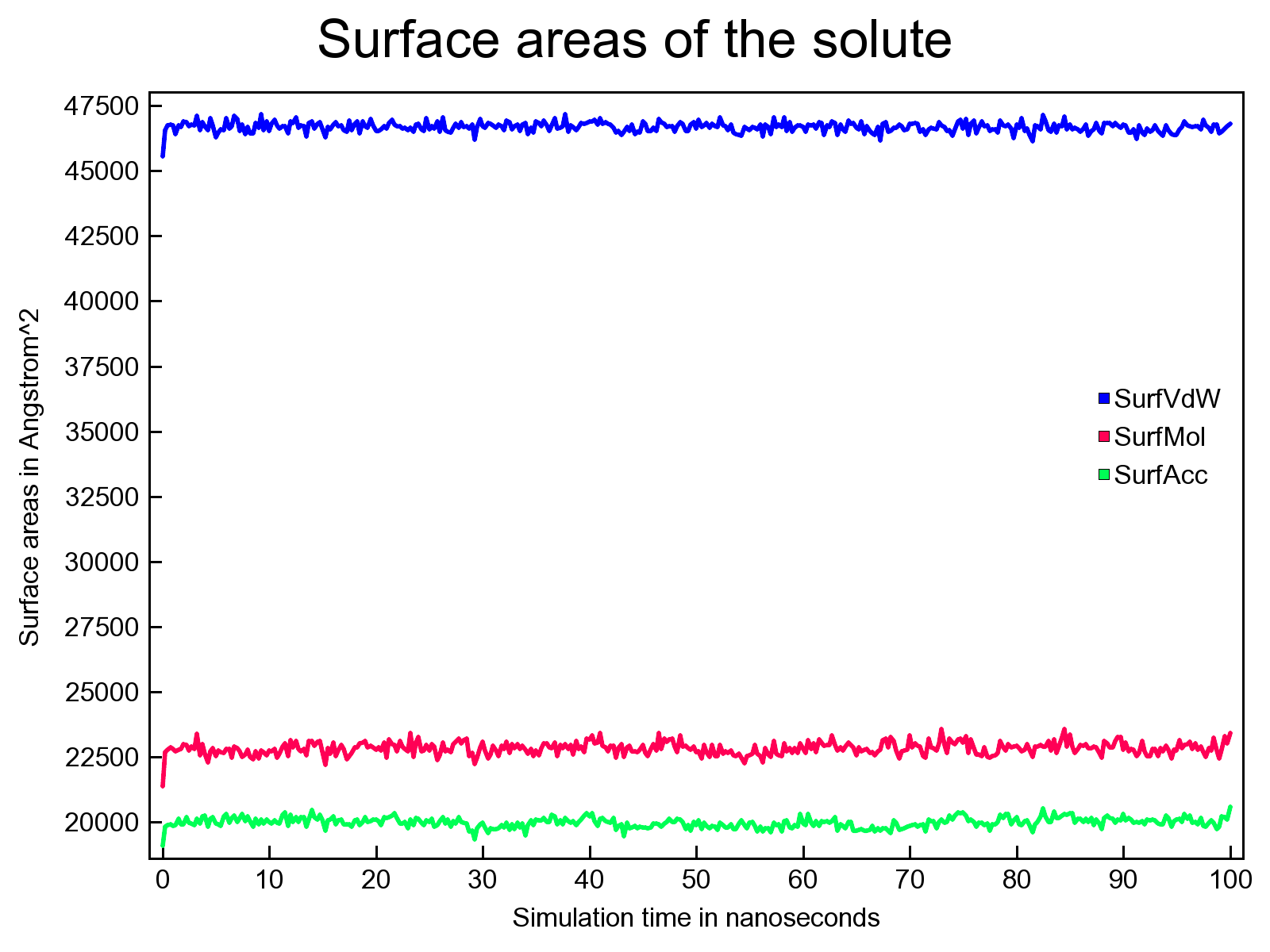

Supplement: S7 File — (ZIP) [file pone.0338211.s007.zip › S6.Molecular Dynamic Simulation/S6.Molecular Dynamic Simulation/Result napitane+roluperidone/Result 2/Complex one/Complex one_report_figure6_hires.png]

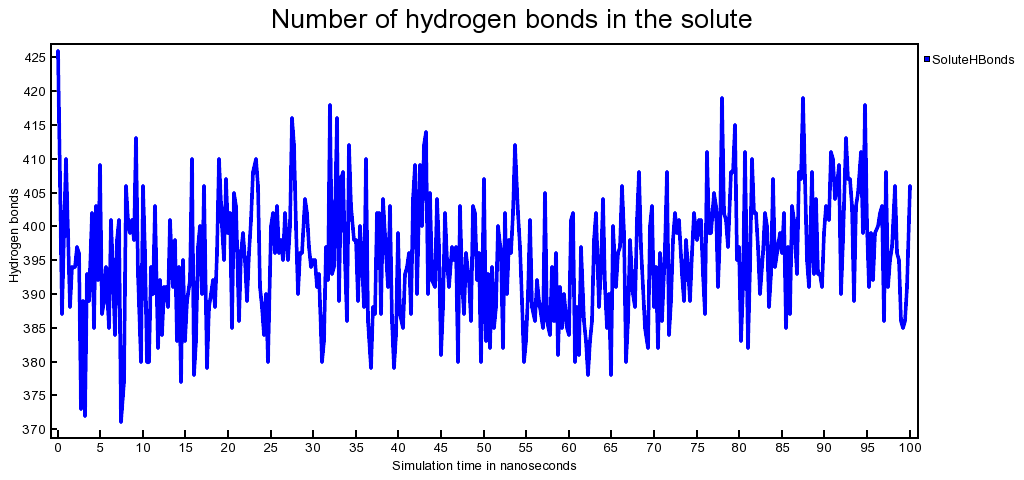

Supplement: S7 File — (ZIP) [file pone.0338211.s007.zip › S6.Molecular Dynamic Simulation/S6.Molecular Dynamic Simulation/Result napitane+roluperidone/Result 2/Complex one/Complex one_report_figure7.png]

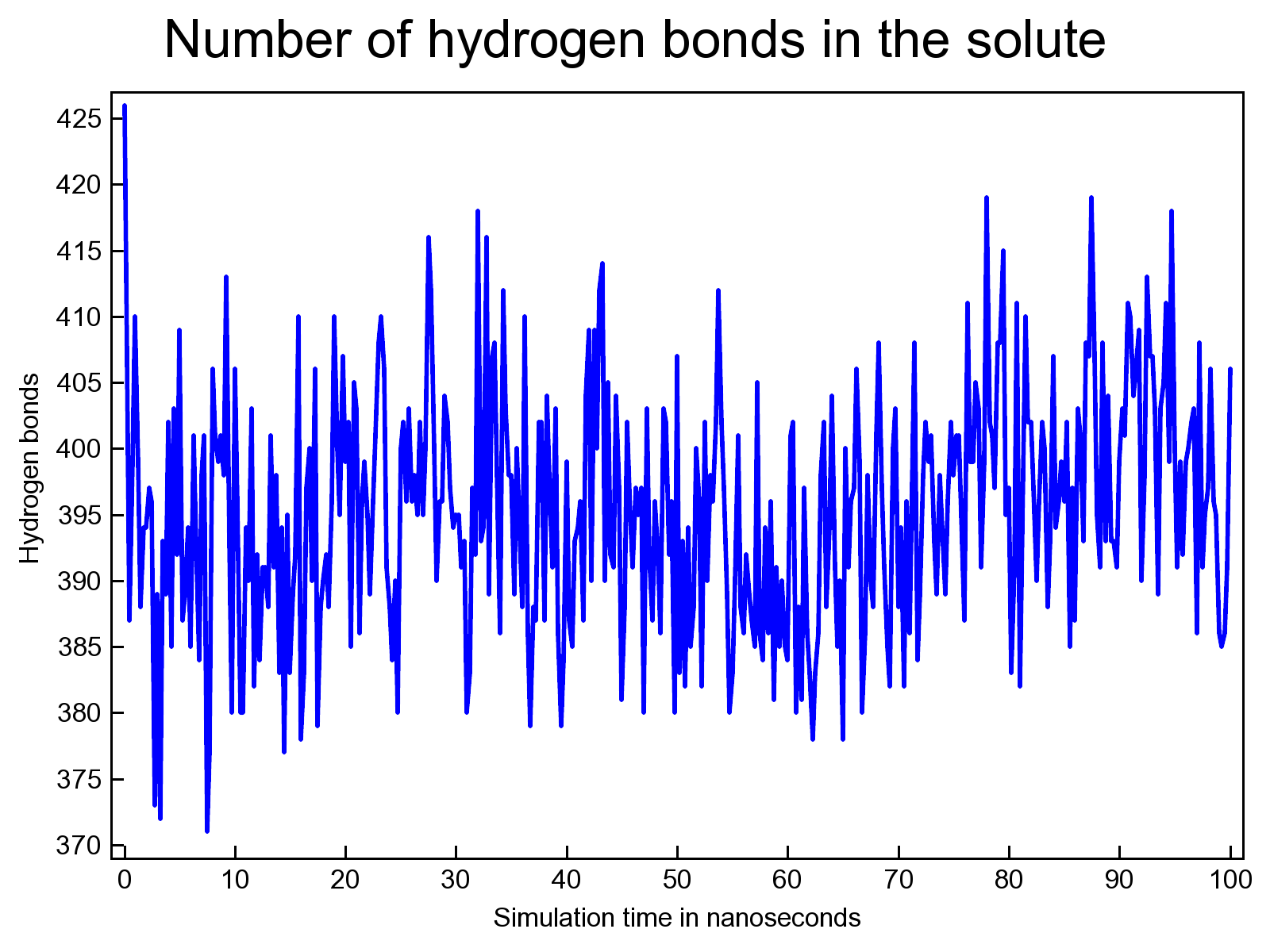

Supplement: S7 File — (ZIP) [file pone.0338211.s007.zip › S6.Molecular Dynamic Simulation/S6.Molecular Dynamic Simulation/Result napitane+roluperidone/Result 2/Complex one/Complex one_report_figure7_hires.png]

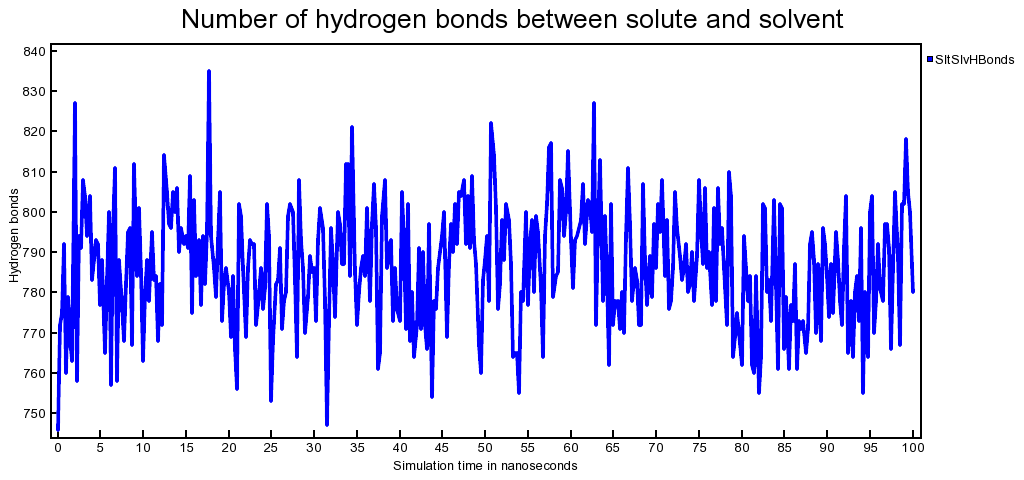

Supplement: S7 File — (ZIP) [file pone.0338211.s007.zip › S6.Molecular Dynamic Simulation/S6.Molecular Dynamic Simulation/Result napitane+roluperidone/Result 2/Complex one/Complex one_report_figure8.png]

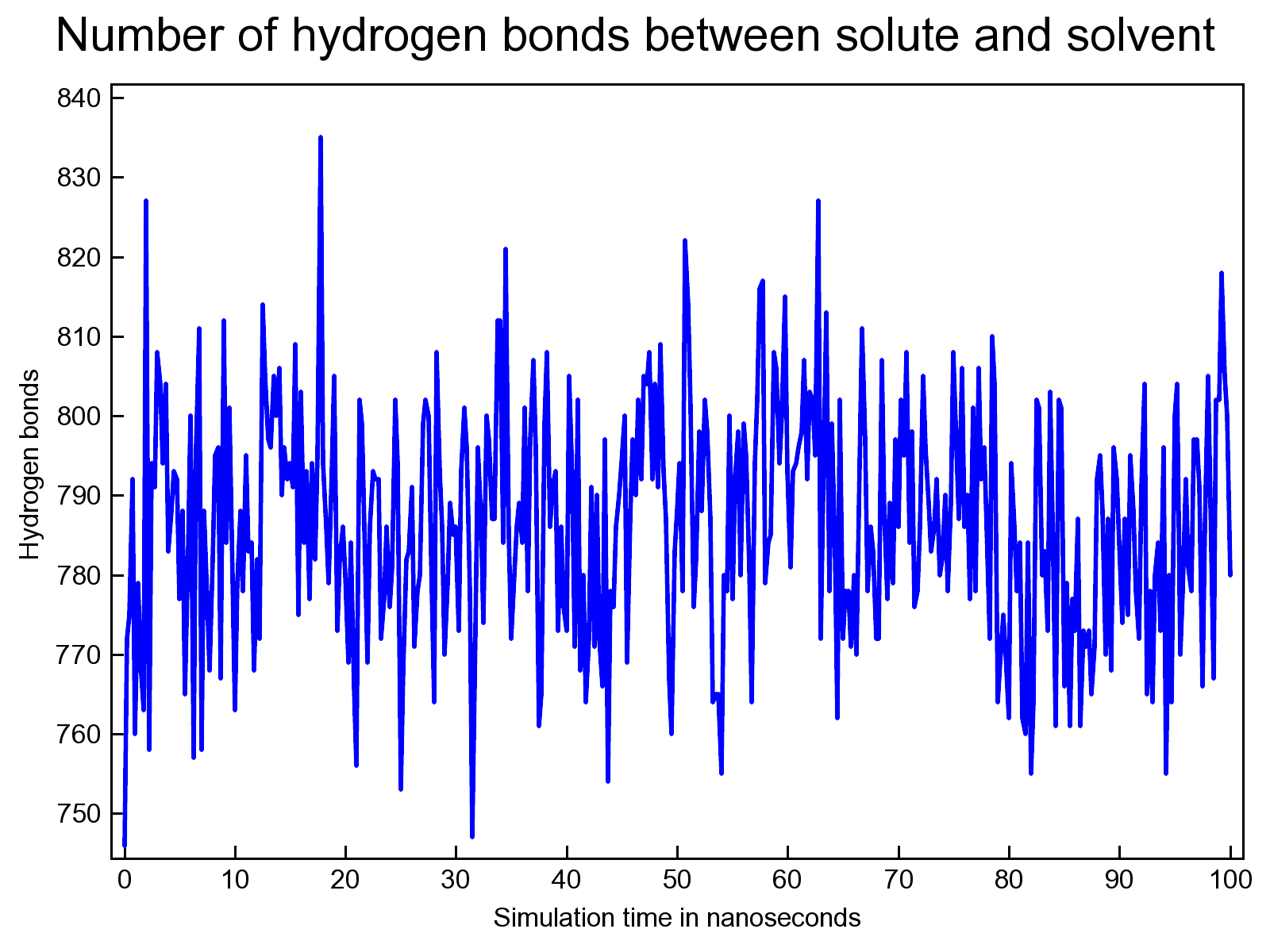

Supplement: S7 File — (ZIP) [file pone.0338211.s007.zip › S6.Molecular Dynamic Simulation/S6.Molecular Dynamic Simulation/Result napitane+roluperidone/Result 2/Complex one/Complex one_report_figure8_hires.png]

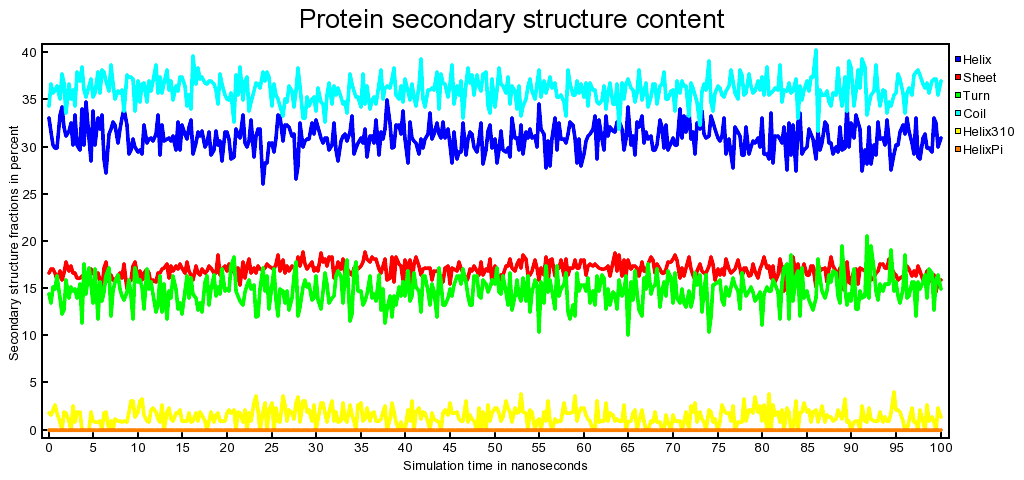

Supplement: S7 File — (ZIP) [file pone.0338211.s007.zip › S6.Molecular Dynamic Simulation/S6.Molecular Dynamic Simulation/Result napitane+roluperidone/Result 2/Complex one/Complex one_report_figure9.png]

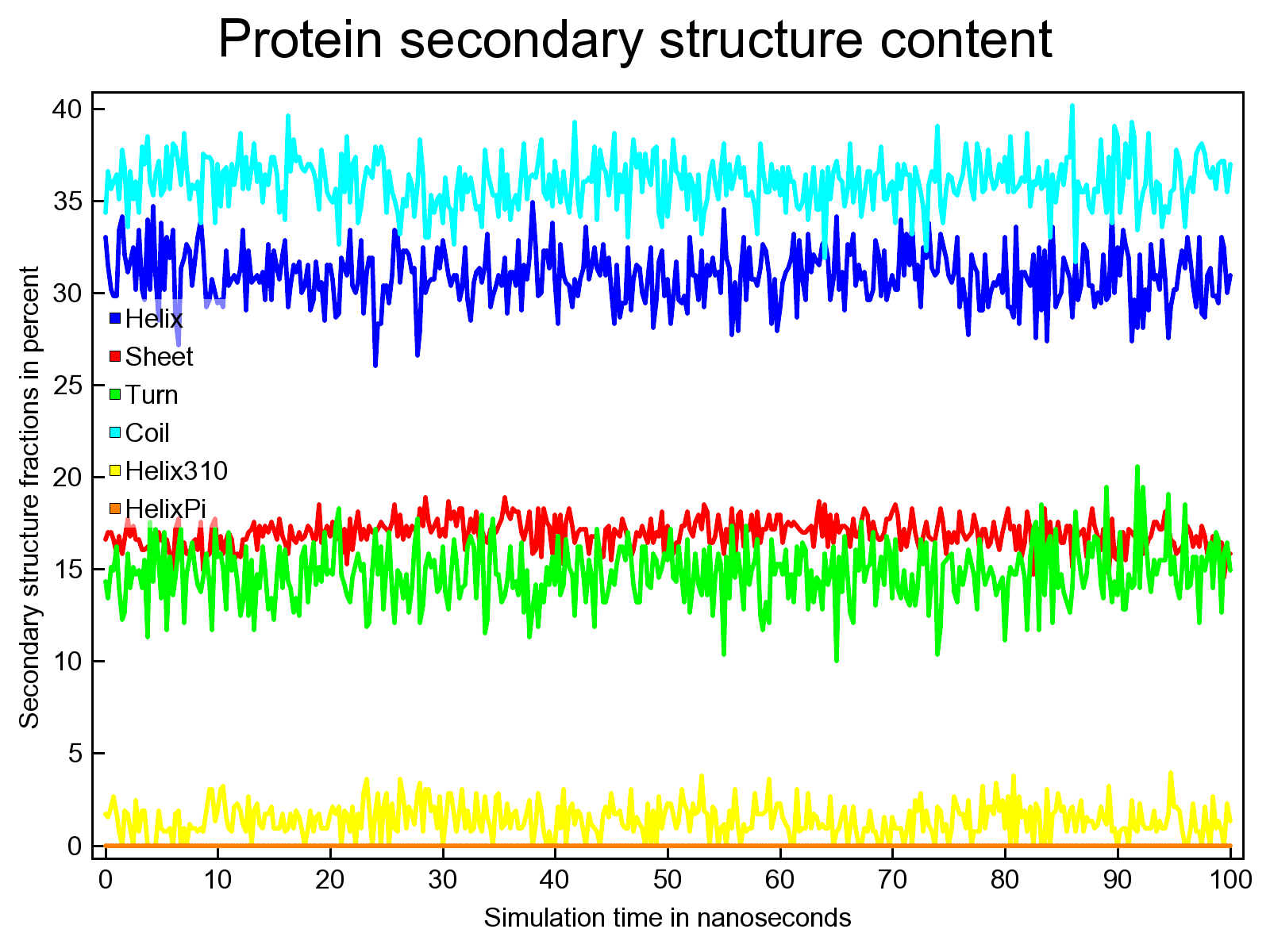

Supplement: S7 File — (ZIP) [file pone.0338211.s007.zip › S6.Molecular Dynamic Simulation/S6.Molecular Dynamic Simulation/Result napitane+roluperidone/Result 2/Complex one/Complex one_report_figure9_hires.png]

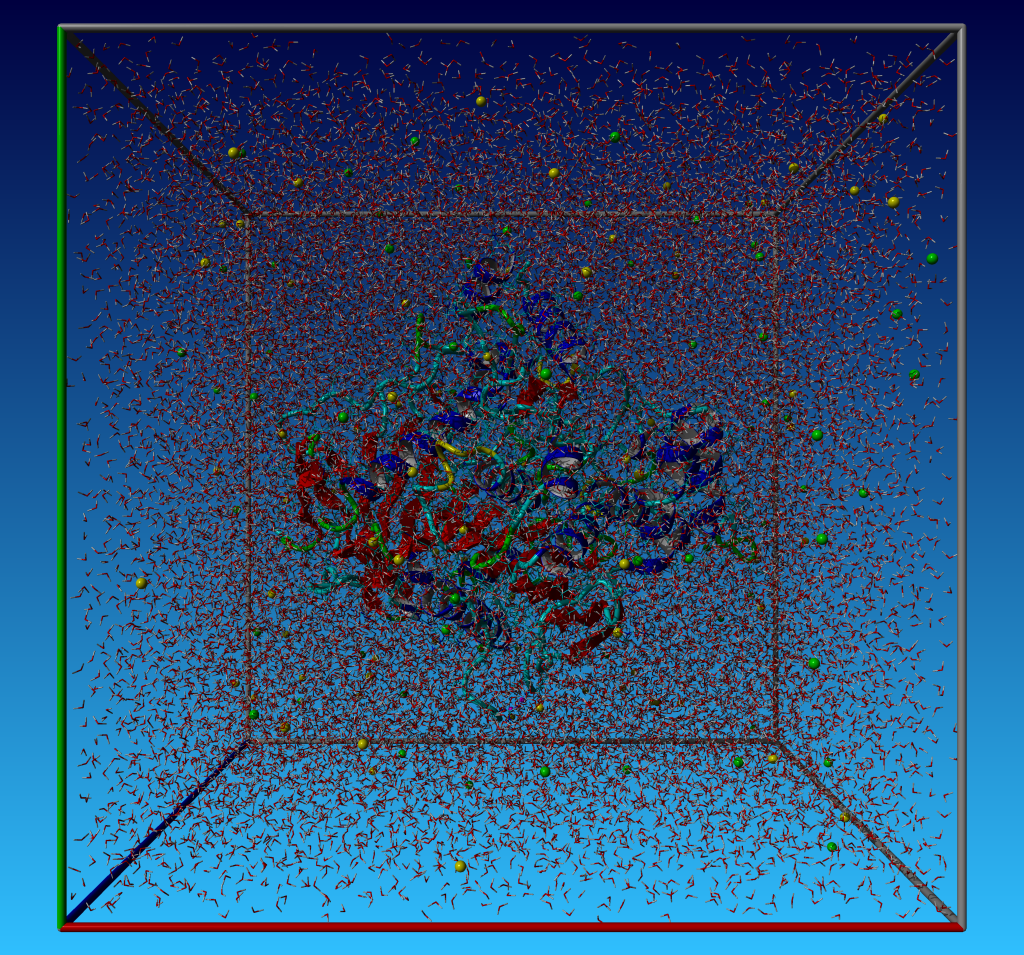

Supplement: S7 File — (ZIP) [file pone.0338211.s007.zip › S6.Molecular Dynamic Simulation/S6.Molecular Dynamic Simulation/Result napitane+roluperidone/Result 2/Complex two/Complex two_report_figure1.png]

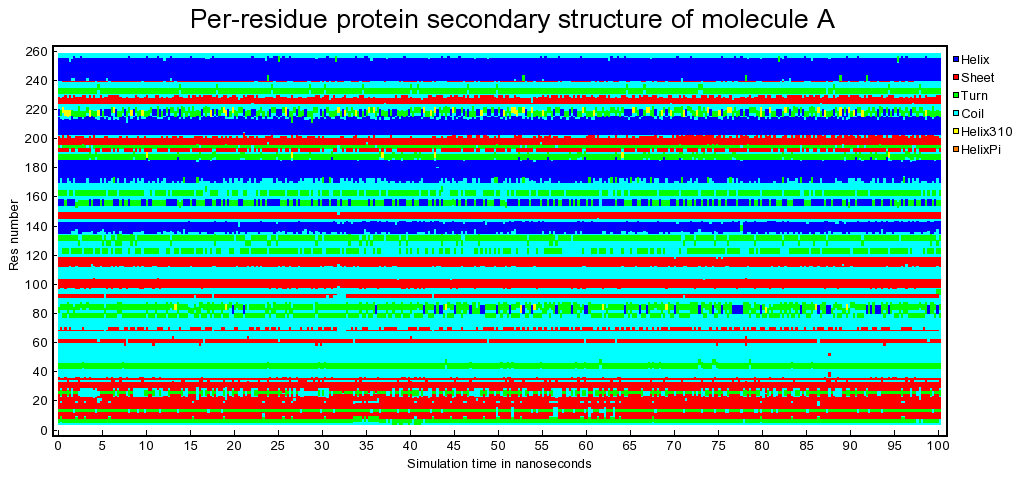

Supplement: S7 File — (ZIP) [file pone.0338211.s007.zip › S6.Molecular Dynamic Simulation/S6.Molecular Dynamic Simulation/Result napitane+roluperidone/Result 2/Complex two/Complex two_report_figure10.png]

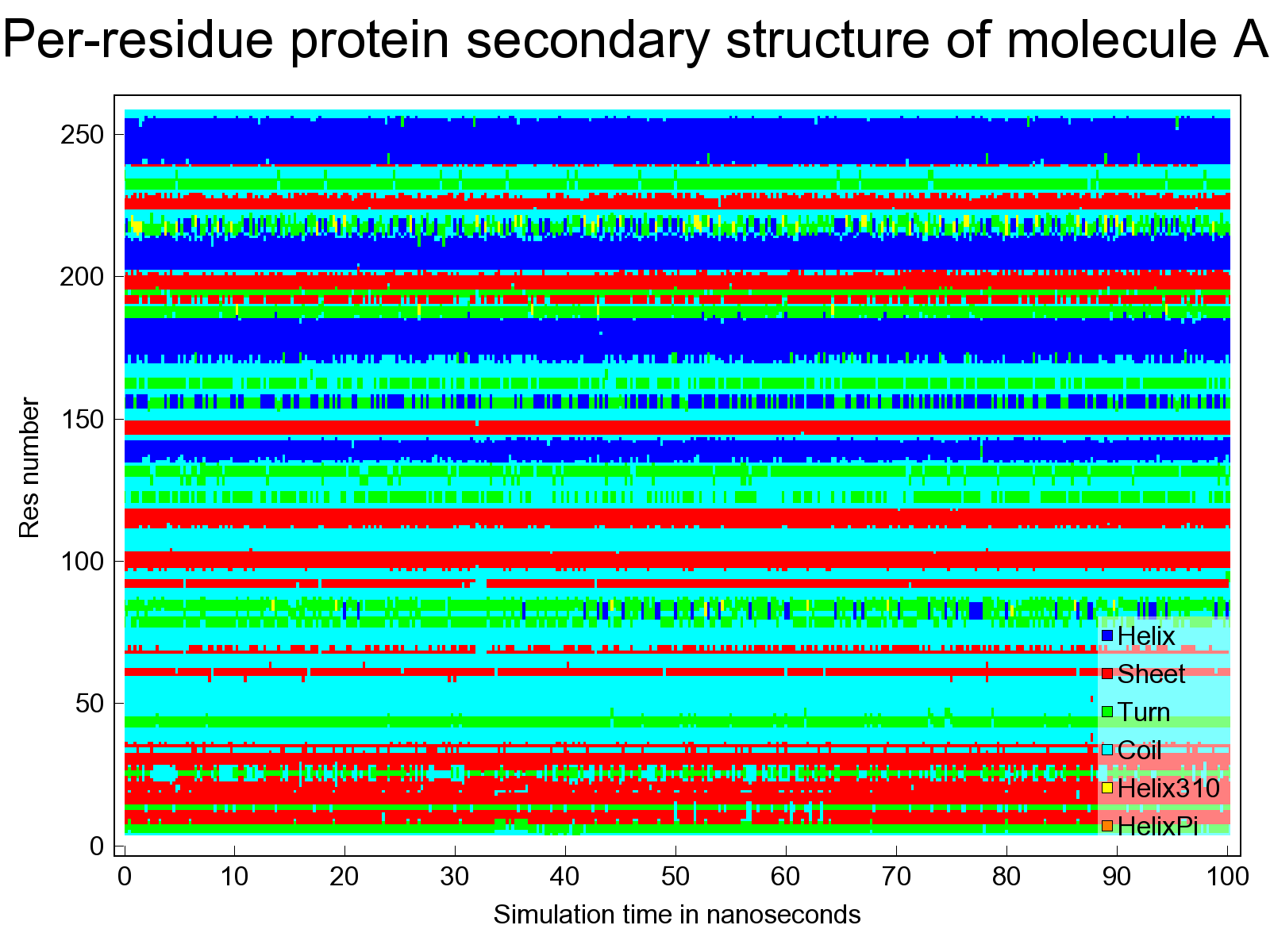

Supplement: S7 File — (ZIP) [file pone.0338211.s007.zip › S6.Molecular Dynamic Simulation/S6.Molecular Dynamic Simulation/Result napitane+roluperidone/Result 2/Complex two/Complex two_report_figure10_hires.png]

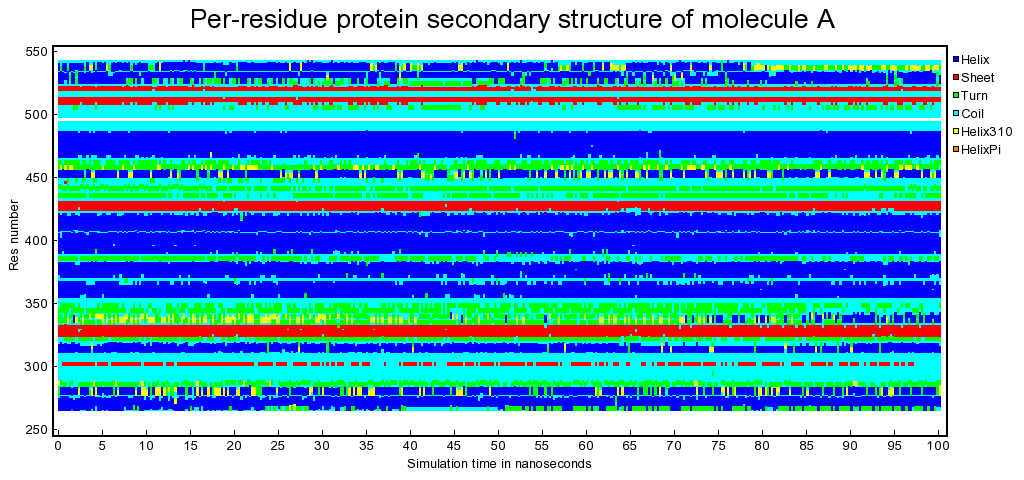

Supplement: S7 File — (ZIP) [file pone.0338211.s007.zip › S6.Molecular Dynamic Simulation/S6.Molecular Dynamic Simulation/Result napitane+roluperidone/Result 2/Complex two/Complex two_report_figure11.png]

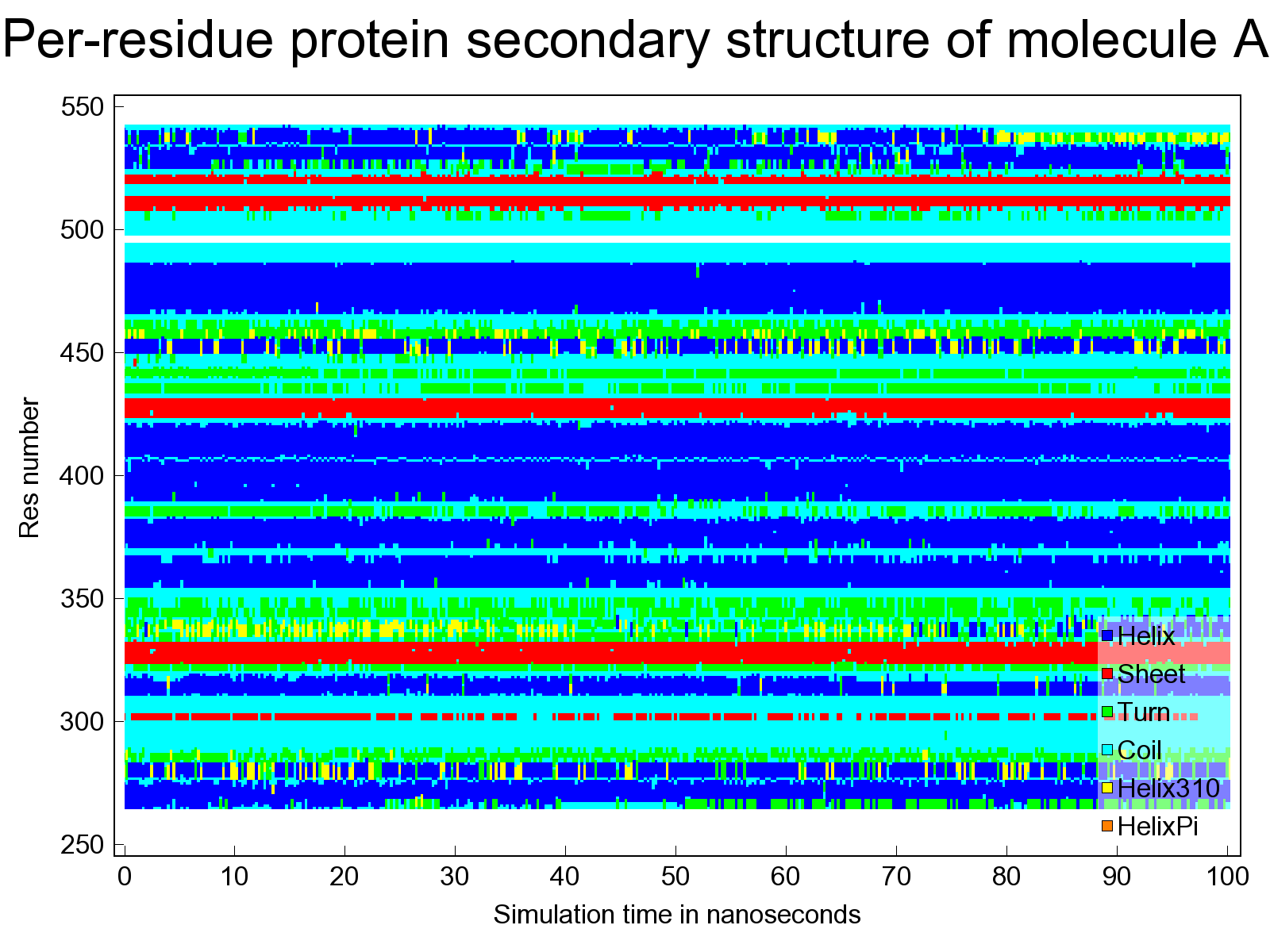

Supplement: S7 File — (ZIP) [file pone.0338211.s007.zip › S6.Molecular Dynamic Simulation/S6.Molecular Dynamic Simulation/Result napitane+roluperidone/Result 2/Complex two/Complex two_report_figure11_hires.png]

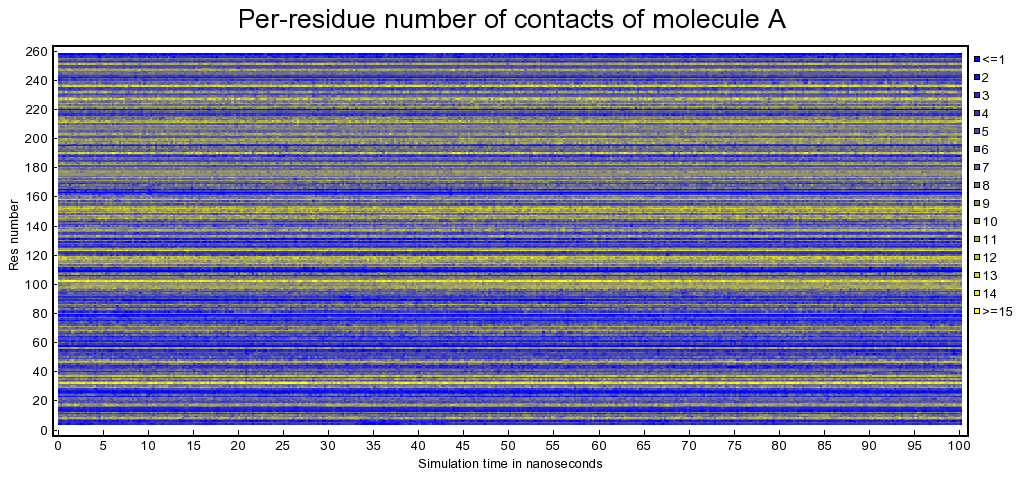

Supplement: S7 File — (ZIP) [file pone.0338211.s007.zip › S6.Molecular Dynamic Simulation/S6.Molecular Dynamic Simulation/Result napitane+roluperidone/Result 2/Complex two/Complex two_report_figure12.png]

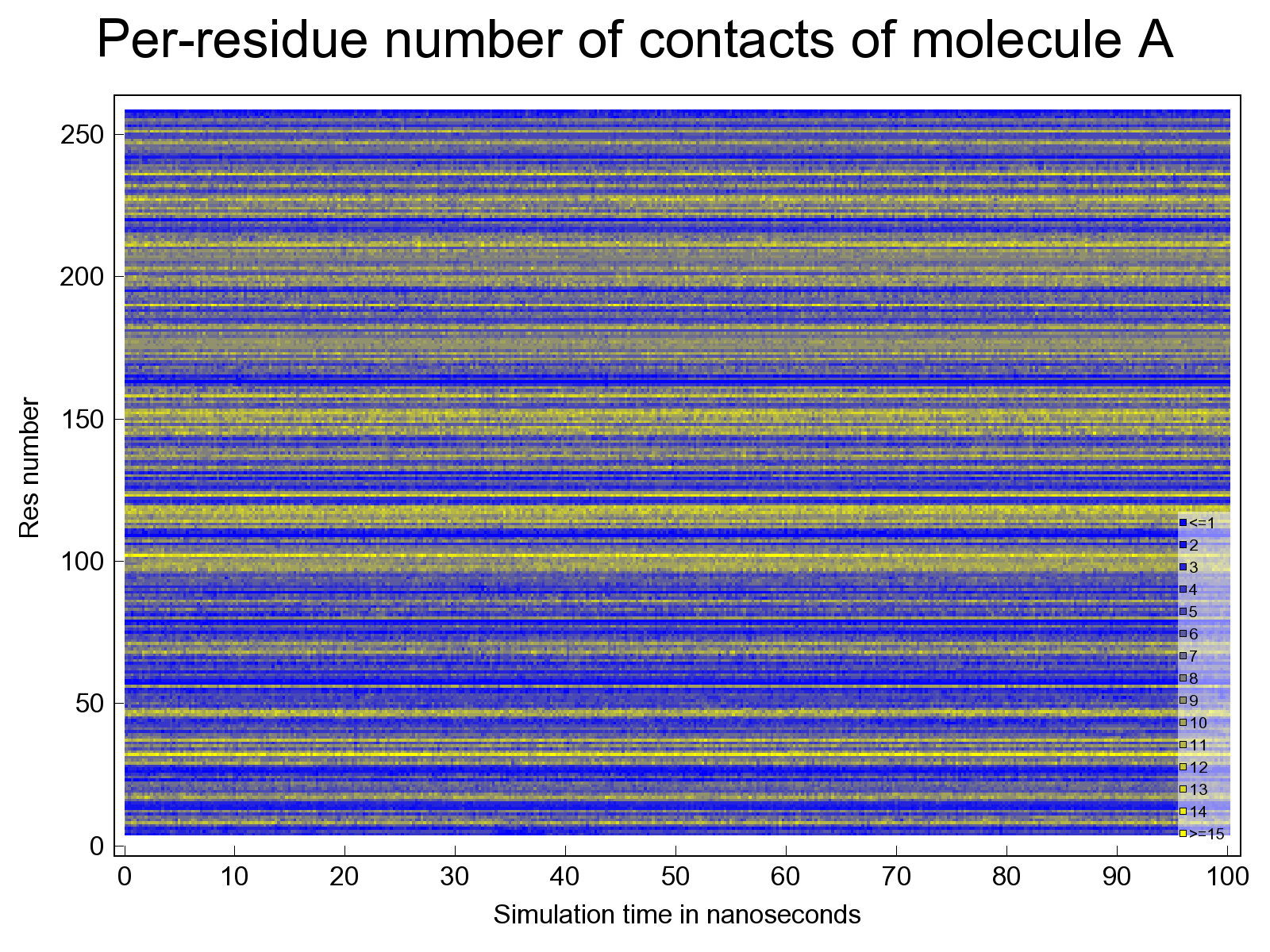

Supplement: S7 File — (ZIP) [file pone.0338211.s007.zip › S6.Molecular Dynamic Simulation/S6.Molecular Dynamic Simulation/Result napitane+roluperidone/Result 2/Complex two/Complex two_report_figure12_hires.png]

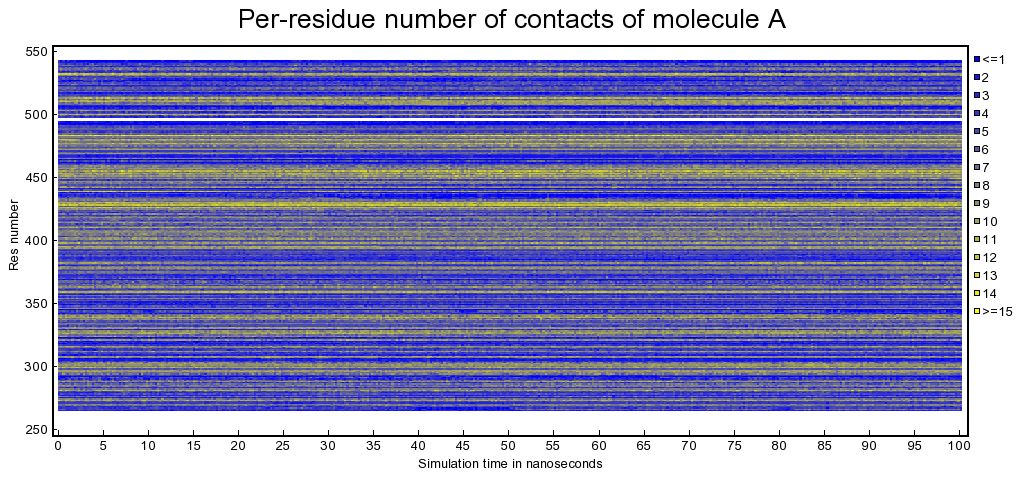

Supplement: S7 File — (ZIP) [file pone.0338211.s007.zip › S6.Molecular Dynamic Simulation/S6.Molecular Dynamic Simulation/Result napitane+roluperidone/Result 2/Complex two/Complex two_report_figure13.png]

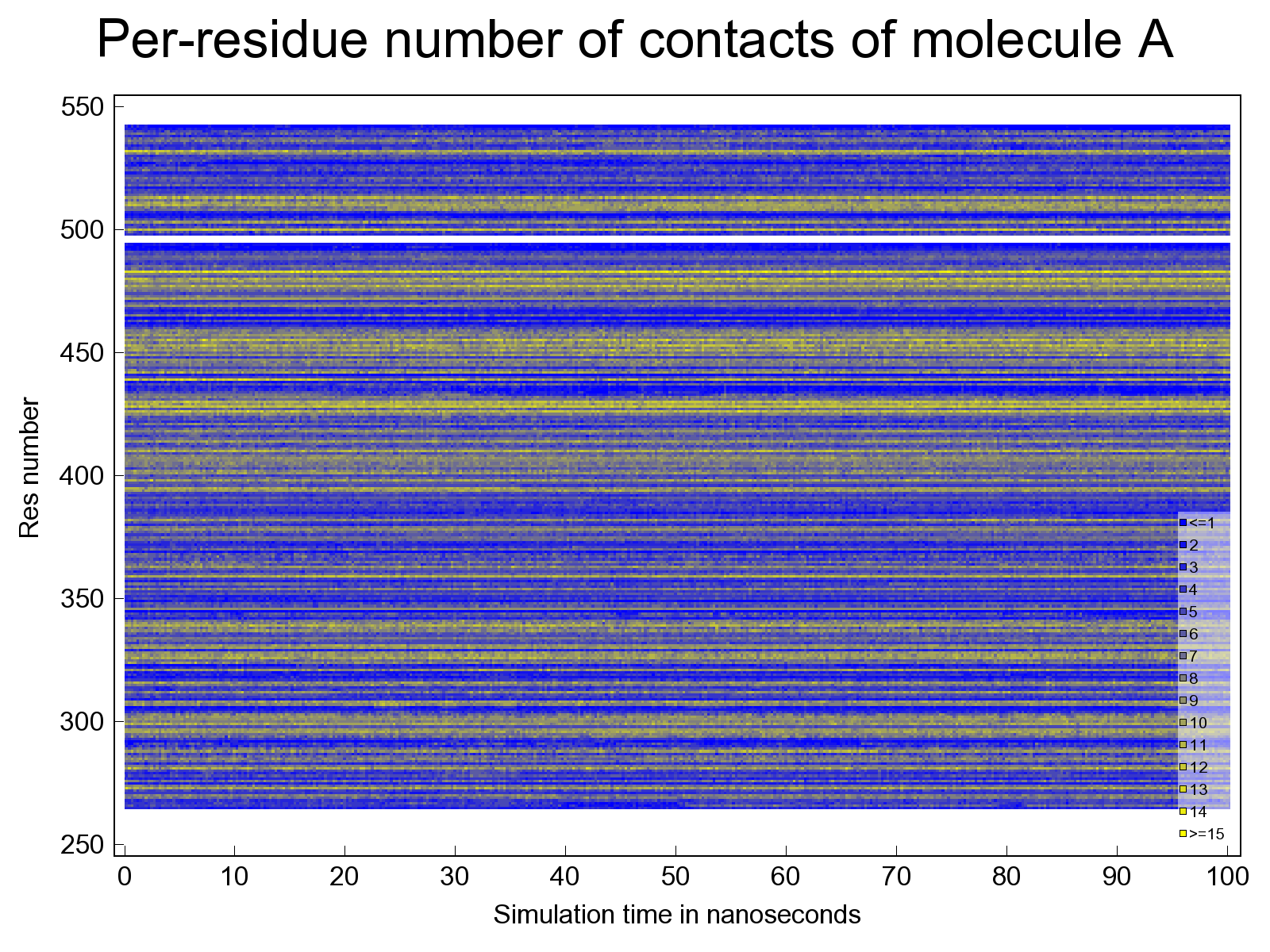

Supplement: S7 File — (ZIP) [file pone.0338211.s007.zip › S6.Molecular Dynamic Simulation/S6.Molecular Dynamic Simulation/Result napitane+roluperidone/Result 2/Complex two/Complex two_report_figure13_hires.png]

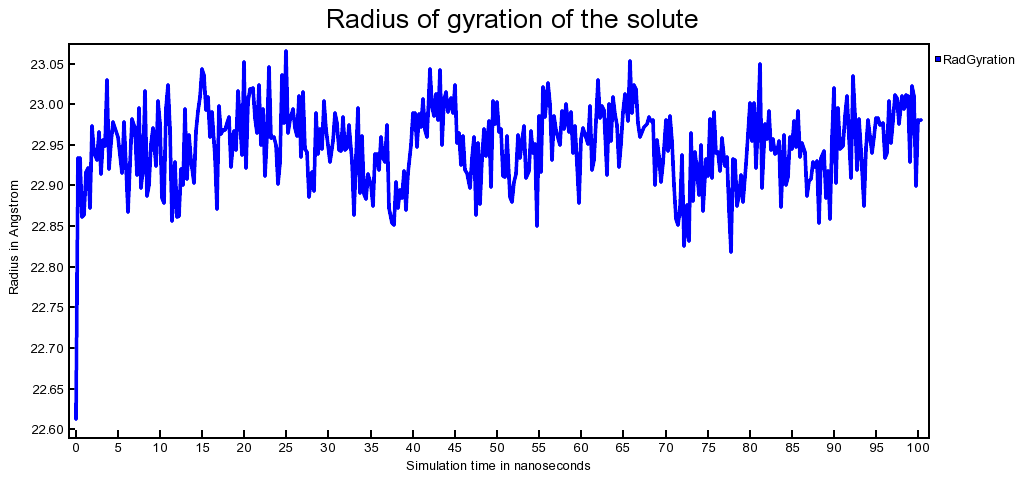

Supplement: S7 File — (ZIP) [file pone.0338211.s007.zip › S6.Molecular Dynamic Simulation/S6.Molecular Dynamic Simulation/Result napitane+roluperidone/Result 2/Complex two/Complex two_report_figure14.png]

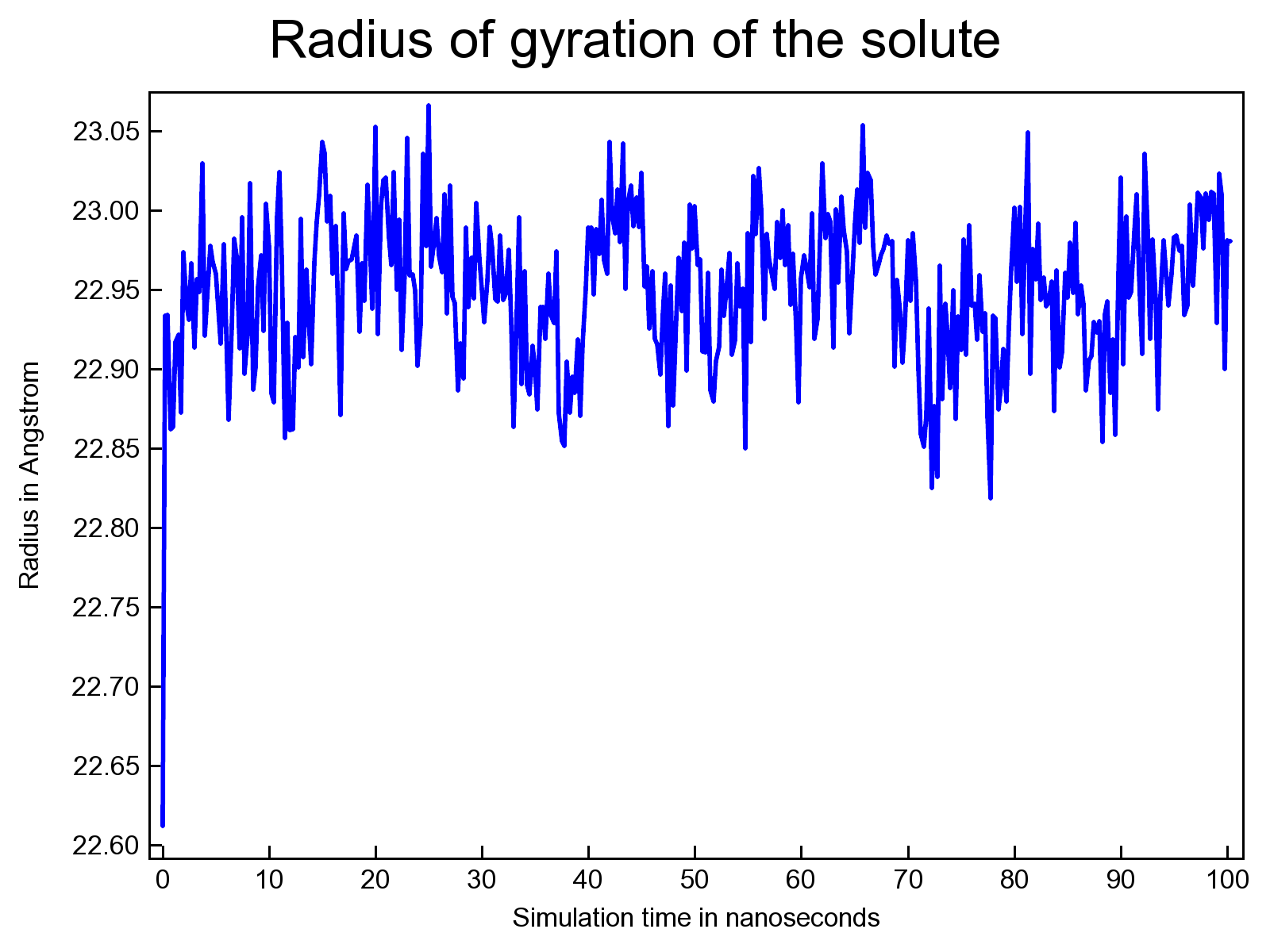

Supplement: S7 File — (ZIP) [file pone.0338211.s007.zip › S6.Molecular Dynamic Simulation/S6.Molecular Dynamic Simulation/Result napitane+roluperidone/Result 2/Complex two/Complex two_report_figure14_hires.png]

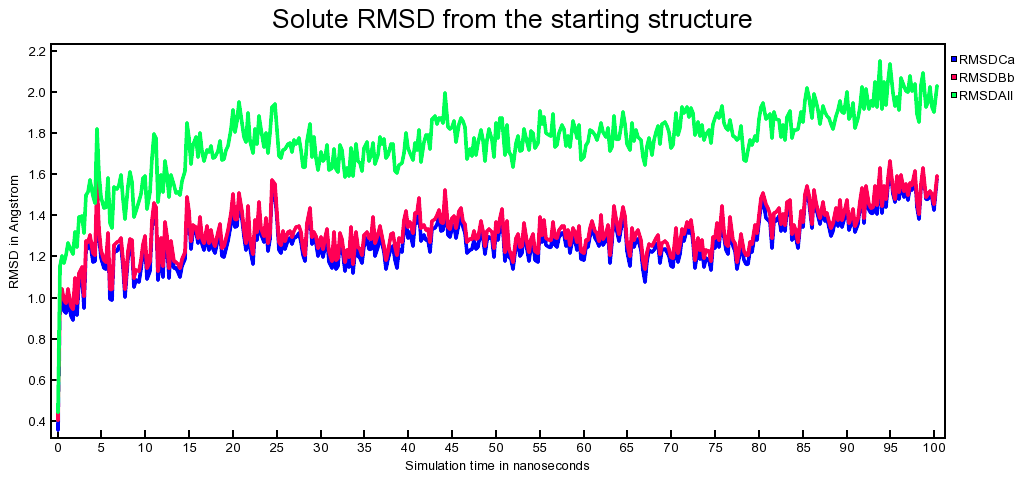

Supplement: S7 File — (ZIP) [file pone.0338211.s007.zip › S6.Molecular Dynamic Simulation/S6.Molecular Dynamic Simulation/Result napitane+roluperidone/Result 2/Complex two/Complex two_report_figure15.png]

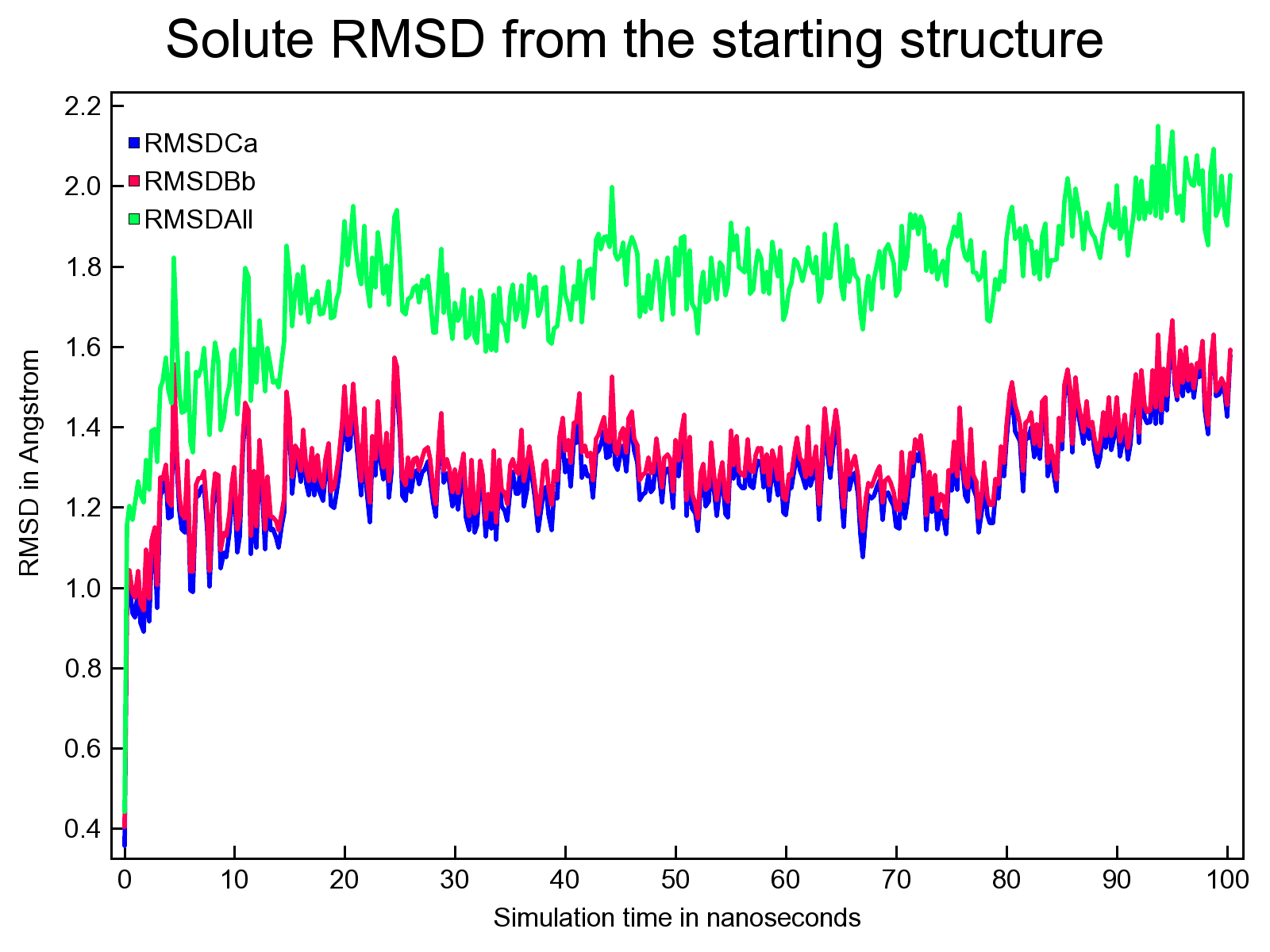

Supplement: S7 File — (ZIP) [file pone.0338211.s007.zip › S6.Molecular Dynamic Simulation/S6.Molecular Dynamic Simulation/Result napitane+roluperidone/Result 2/Complex two/Complex two_report_figure15_hires.png]
