# Supplementary material for: An evaluation of Roluperidone as a promising repurposing candidate for Alzheimer’s Disease: A Computational Investigation
Source: PLoS One. 2025 Dec 17;20(12):e0338211. doi: 10.1371/journal.pone.0338211 (PMC12711050; doi:10.1371/journal.pone.0338211)
Supplement: S7 File — (ZIP) [file pone.0338211.s007.zip › S6.Molecular Dynamic Simulation/S6.Molecular Dynamic Simulation/Result napitane+roluperidone/Result 2/Complex two/Complex two_report.html]

YASARA Molecular Dynamics Trajectory Analysis for Complex two 


# YASARA Molecular Dynamics Trajectory Analysis for Complex two

## 1. About the simulation

The trajectory **F:\Simulation\9 kustia\Complex two\Complex two** has been analyzed with YASARA version 23.9.29.W.64 over a period of 100.25 nanoseconds with 402 snapshots and the AMBER14 force field. Note that the MD simulation may have been run with a different force field, but AMBER14 was used to calculate the energies in this report. To change this, edit the ForceField setting at the start of this macro.

All plots and pictures in this report [like the simulated system below] are 1024 pixels wide, you can change the **figurewidth** variable in this macro as needed.

|  |
| --- |
|  |
**Figure-1**: A ray-traced picture of the simulated system. The simulation cell boundary is set to periodic. Atoms that stick out of the simulation cell will be wrapped to the opposite side of the cell during the simulation.

### 1.1. Composition of the system

The components of the system are shown in the table below.

|  |  |
| --- | --- |
| **Type** | **Number** |
| Protein molecules | 2 |
| Protein residues | 530 |
| Protein atoms | 8154 |
| Nucleic acid molecules | 0 |
| Nucleic acid residues | 0 |
| Nucleic acid atoms | 0 |
| Residue UNK with 51 atoms | 1 |
| Residue CIP with 1 atom of element Na | 81 |
| Residue CIM with 1 atom of element Cl | 76 |
| Water residues | 27677 |
| Total number of atoms | 91393 |
**Table-1**: Composition of the simulated system

Object 1 with name **Complex\_tw\_1** has been identified as the solute and is shown below. If this is not the intended solute, please change the **soluteobj** variable in this macro.

|  |
| --- |
|  |
**Figure-2**: The solute oriented along the major axes.

## 2. Analyses inside the simulation cell

This section shows all analyses that have been performed inside the simulation cell, when all atoms share the common coordinate system of the simulation cell.

Periodic boundaries are active and considered for distance measurements. Calculations that involve groups of atoms [center of mass, regression lines, enclosing spheres..] are ambiguous and should be placed in the next section, unless it is known that the atom group does not drift through a periodic boundary.

### 2.1. Simulation cell lengths

Conformational changes of the simulated solute molecules lead to fluctuations in density. If the simulation box has a constant size, changes in density lead to changes in pressure. This is not realistic, because molecules normally "live" in a constant pressure environment. During the simulation the cell is therefore rescaled to maintain a constant cell pressure. Depending on the chosen pressure control mode, the three cell axes are either rescaled together [Manometer1D], partly together [X- and Z-axes, Manometer2D, used for membrane simulations], independently [Manometer3D], or not at all [Off]. You can deduce the pressure control mode from the plot below.

|  |
| --- |
|  |
**Figure-3**: Simulation cell lengths [vertical axis] as a function of simulation time [horizontal axis]. Note: Graph **CellLengthZ** completely covers graph **CellLengthY** and graph **CellLengthX**, they share the same values.

### 2.2. Total potential energy of the system

The total potential energy of the system is plotted, according to the AMBER14 force field. If you ran the simulation with a different force field, you need to adapt the **ForceField** command at the top of this macro accordingly.

When the simulation is started from an energy-minimized "frozen" conformation, there is usually a sharp increase in energy during the first picoseconds, since the added kinetic energy is partly stored as potential energy. Also on a larger time-scale, the potential energy will often not decrease. A common reason are counter ions. These are initially placed at the positions with the lowest potential energy, usually close to charged solute groups, from where they detach to gain entropy, but also potential energy.

|  |
| --- |
|  |
**Figure-4**: Total potential energy of the system [vertical axis] as a function of simulation time [horizontal axis]. Note: The first value of the plot [-1524431.85], coming from the energy minimized starting structure, has been replaced with the second value of the plot [-1233462.08] to show this plot with a smaller energy range and thus a higher resolution.

### 2.3. Potential energy components

The following individual components of the total potential energy are plotted: bond energies [Bond], bond angle energies [Angle], dihedral angle energies [Dihedral], planarity or improper dihedral energies [Planarity], Van der Waals energies [VdW] and electrostatic energies [Coulomb]. Force field energies help to judge the structural quality of a protein: distortions of local covalent geometry can be found by looking at the bond, angle and planarity energies. Unrealistically close contacts [bumps] lead to a high Van der Waals energy, just like a large number of hydrogen bonds [since they pull the atoms closer than their normal Van der Waals contact distance]. The Coulomb energy is the least informative, because it strongly depends on the amino acid composition [e.g. proteins with a net charge have a higher Coulomb energy].

|  |
| --- |
|  |
**Figure-5**: Potential energy components [vertical axis] as a function of simulation time [horizontal axis].

### 2.4. Surface areas of the solute

The Van der Waals [SurfVdW], molecular [SurfMol] and solvent accessible [SurfAcc] surface areas of the solute in A^2 are plotted. The difference between these surface types can be summarized as follows:

**Van der Waals surface**: if you think of atoms as spheres with a given Van der Waals radius, then the Van der Waals surface consists of all the points on these spheres that are not inside another sphere. In practice, the Van der Waals surface is of limited use, because it can be found throughout a protein and does not tell much about the interaction with the solvent.

**Molecular surface**: this is the Van der Waals surface from the viewpoint of a solvent molecule, which is a much more useful concept. The water is assumed to be a sphere of a given radius [also called the water probe], that rolls over the solute. Those parts of the Van der Waals surface that the water probe can touch are simply copied to the molecular surface [and called the contact surface]. Clefts in the Van der Waals surface that are too narrow for the water probe to enter are replaced by the Van der Waals surface of the water probe itself [and called the reentrant surface]. So the molecular surface is a smooth composition of two Van der Waals surfaces: the one of the solute and the one of the solvent molecule while it traces the contours of the solute. Other common names for the molecular surface are solvent excluded surface or Connolly surface.

**Solvent accessible surface**: this surface consists of all the points that the center of the water probe [i.e. the nucleus of the oxygen atom in the water molecule] can reach while rolling over the solute. The shortest possible distance between the water oxygen nucleus and a solute atom is simply the sum of the Van der Waals radii of the solute atom and the water probe.

|  |
| --- |
|  |
**Figure-6**: Surface areas of the solute [vertical axis] as a function of simulation time [horizontal axis], obtained with the command "SurfObj Solute".

### 2.5. Number of hydrogen bonds in the solute

The number of hydrogen bonds inside the solute is plotted below. One hydrogen bond per hydrogen atom is assigned at most, picking the better one if two acceptors are available.The following formula yields the bond energy in [kJ/mol] as a function of the Hydrogen-Acceptor distance in [A] and two scaling factors:

The first scaling factor depends on the angle formed by Donor-Hydrogen-Acceptor:

The second scaling factor is derived from the angle formed by Hydrogen-Acceptor-X, where X is the atom covalently bound to the acceptor. If X is a heavy atom:

If X is a hydrogen, slightly smaller angles are allowed:

A hydrogen bond is counted if the hydrogen bond energy obtained with this formula is better than 6.25 kJ/mol [or 1.5 kcal/mol], which is 25% of the optimum value 25 kJ/mol.

|  |
| --- |
|  |
**Figure-7**: Number of hydrogen bonds in the solute [vertical axis] as a function of simulation time [horizontal axis].

### 2.6. Number of hydrogen bonds between solute and solvent

The plot shows the number of hydrogen bonds between solute and solvent. Together with the plot above, it is a good indicator for successful protein folding, indicated by a decreasing number of bonds with the solvent and a growing number of bonds within the solute.

|  |
| --- |
|  |
**Figure-8**: Number of hydrogen bonds between solute and solvent [vertical axis] as a function of simulation time [horizontal axis].

### 2.7. Protein secondary structure content

The total percentages of alpha helices, beta sheets, turns, coils, 3-10 helices and pi helices are calculated and plotted. For clarification, a turn is simply a stretch of four residues that are not part of other secondary structure elements and form a hydrogen bond between the O of the first and the NH of the last residue. A coil is anything that does not fit into the other categories. Note that pi-helices [helices with hydrogen bonds between residues N and N+5] are rather unstable and thus do not normally occur in proteins, except for short bulges in alpha helices [which are often the result of single residue insertions and prolines].

|  |
| --- |
|  |
**Figure-9**: Protein secondary structure content [vertical axis] as a function of simulation time [horizontal axis], obtained with the command "SecStr". Note: Graph **HelixPi** has all zero values.

### 2.8. Per-residue protein secondary structure

The following plots show the protein secondary structure per residue as a function of simulation time. They are helpful to monitor protein folding and all other kinds of structural changes. The default secondary structure colors are used, you can change them at View > Color > Parameters > Secondary structure colors. One plot per protein molecule is shown.

|  |
| --- |
|  |
**Figure-10**: Per-residue protein secondary structure as a function of simulation time [horizontal axis] for each Res number [vertical axis]. A table with the raw data including percentages is available here: Complex two\_plotres\_secstrMolA.tab. Values 1-6 in the table correspond to the 6 labels in the plot legend.

|  |
| --- |
|  |
**Figure-11**: Per-residue protein secondary structure as a function of simulation time [horizontal axis] for each Res number [vertical axis]. A table with the raw data including percentages is available here: Complex two\_plotres\_secstrMolA.tab. Values 1-6 in the table correspond to the 6 labels in the plot legend.

### 2.9. Per-residue number of contacts

The number of contacts per residue as a function of simulation time is shown in the following plots. There is one plot for each protein or nucleic acid molecule. Even though contacts between atoms separated by up to four chemical bonds are excluded, neighboring residues in the molecule usually have enough close atoms to be counted as a contact. Consequently residues with zero contacts are very rare and often glycines. The number of contacts tells you how densely a certain residue range is packed and allows to identify structurally very important residues, e.g. a phenylalanine in the hydrophobic core can contact 15 or more other residues.

|  |
| --- |
|  |
**Figure-12**: Per-residue number of contacts as a function of simulation time [horizontal axis] for each Res number [vertical axis]. A table with the raw data including percentages is available here: Complex two\_plotres\_conMolA.tab

|  |
| --- |
|  |
**Figure-13**: Per-residue number of contacts as a function of simulation time [horizontal axis] for each Res number [vertical axis]. A table with the raw data including percentages is available here: Complex two\_plotres\_conMolA.tab

## 3. Analyses outside the simulation cell

The following section presents data gathered outside the simulation cell, where each object has its own local coordinate system and no periodic boundaries are present. Calculations that involve the interaction between objects [common surface areas, contacts between objects..] must be placed in the previous section.

### 3.1. Radius of gyration of the solute

After determining the center of mass of the solute, the radius of gyration is calculated and plotted according to this formula:

In this formula, **C** is the center of mass, and **Ri** is the position of atom **i** of **N**.

|  |
| --- |
|  |
**Figure-14**: Radius of gyration of the solute [vertical axis] as a function of simulation time [horizontal axis], obtained with the command "RadiusObj Solute,Center=Mass,Type=Gyration".

## 4. Analyses performed with respect to the starting structure

Analyses performed with respect to the starting structure are shown in this section. These are also done outside the simulation cell, where each object has its own local coordinate systems and no periodic boundaries are present. To choose another reference snapshot than 0, edit the **refsnapshot** variable at the beginning of this macro.

### 4.1. Solute RMSD from the starting structure

The plot shows Calpha [RMSDCa], backbone [RMSDBb] and all-heavy atom [RMSDAll] RMSDs calculated according to this formula, where **Ri** is the vector linking the positions of atom **i** [of **N** atoms] in the reference snapshot and the current snapshot after optimal superposition:

The selection for the Calpha RMSD calculation is **CA Protein or C1\* NucAcid and Obj Solute**, this matched 530 atoms. The Calpha selection thus includes the main backbone carbon C1\* of nucleic acids, so the plot also shows a Calpha RMSD if you simulate just nucleic acids. In simulations of protein-DNA complexes, the Calpha RMSD therefore considers the DNA too. To change the Calpha selection, edit the **casel** variable at the beginning of this macro.

|  |
| --- |
|  |
**Figure-15**: Solute RMSD from the starting structure [vertical axis] as a function of simulation time [horizontal axis].

## 5. Solute residue RMSF

The Root Mean Square Fluctuation [RMSF] per solute residue is calculated from the average RMSF of its constituting atoms. The RMSF of atom i with j runing from 1 to 3 for the x, y, and z coordinate of the atom position vector P and k runing over the set of N evaluated snapshots is given by following formula:

Each graph in the following plot represents one molecule, so that you can easily see differences between molecules. Note: Residue numbers are not unique, so graphs can overlap.

|  |
| --- |
|  |
**Figure-16**: The Root Mean Square Fluctuation [vertical axis] per solute protein/nucleic acid residue [horizontal axis] calculated from the average RMSF of the atoms constituting the residue. A RMSF of exactly zero means that that residue number is not present in the molecule. Atom RMSF table: Complex two\_rmsf.tab, residue RMSF table: Complex two\_rmsfres.tab

In case the plot above is too crowded, the per-residue RMSF values are shown separately for all 2 molecules in the following plots:

|  |
| --- |
|  |
**Figure-17**: The Root Mean Square Fluctuation [vertical axis] per solute protein/nucleic acid residue [horizontal axis] calculated from the average RMSF of the atoms constituting the residue. A RMSF of exactly zero means that that residue number is not present in the molecule. Atom RMSF table: Complex two\_rmsf.tab, residue RMSF table: Complex two\_rmsfres.tab

|  |
| --- |
|  |
**Figure-18**: The Root Mean Square Fluctuation [vertical axis] per solute protein/nucleic acid residue [horizontal axis] calculated from the average RMSF of the atoms constituting the residue. A RMSF of exactly zero means that that residue number is not present in the molecule. Atom RMSF table: Complex two\_rmsf.tab, residue RMSF table: Complex two\_rmsfres.tab

## 6. Dynamic Cross-Correlation Matrix

The dynamic cross-correlation matrix [DCCM] is a square matrix, whose rows and columns match the selected units **Atom CA Protein or C1\* NucAcid**. To change this selection, edit the **dccmsel** variable at the beginning of this macro. The DCCM shows how the movements of all selected pairs correlate. The values in the DCCM range from -1 [perfectly anti-correlated] to +1 [perfectly correlated]. The values along the diagonal are always +1 [because the motion of an atom is perfectly correlated to itself]. The DCCM element for units i and j is obtained with the following formula:

Here **d** is the displacement between the current position and the average position of the selected unit, and the angle brackets indicate the average over all samples. The highest correlations off the diagonal can often be found for bridged cysteines.

The image below shows the correlation directly in the solute object:

|  |
| --- |
|  |
**Figure-19**: Blue and red lines are shown between 11 strongly anti- and correlated residue pairs. To change the threshold value for the correlation lines edit the **dccmcut** variable at the beginning of this macro. To look at this structure interactively, open the file Complex two\_dccm.yob in YASARA.

In the image below, the DCCM is visualized with colors ranging from blue [-1, fully anti-correlated] to yellow [+1, fully correlated].

|  |
| --- |
|  |
**Figure-20**: Visualization of the dynamic cross-correlation matrix. Open the file Complex two\_dccm.sce in YASARA to look at this matrix visualization interactively. In the scene file, the zero level [0, not correlated] is indicated with a wire-frame grid.

|  |  |  |  |  |  |  |  |  |  |  |  |  |  |  |  |  |  |  |  |  |  |  |  |  |  |  |  |  |  |  |  |  |  |  |  |  |  |  |  |  |  |  |  |  |  |  |  |  |  |  |  |  |  |  |  |  |  |  |  |  |  |  |  |  |  |  |  |  |  |  |  |  |  |  |  |  |  |  |  |  |  |  |  |  |  |  |  |  |  |  |  |  |  |  |  |  |  |  |  |  |  |  |  |  |  |  |  |  |  |  |  |  |  |  |  |  |  |  |  |  |  |  |  |  |  |  |  |  |  |  |  |  |  |  |  |  |  |  |  |  |  |  |  |  |  |  |  |  |  |  |  |  |  |  |  |  |  |  |  |  |  |  |  |  |  |  |  |  |  |  |  |  |  |  |  |  |  |  |  |  |  |  |  |  |  |  |  |  |  |  |  |  |  |  |  |  |  |  |  |  |  |  |  |  |  |  |  |  |  |  |  |  |  |  |  |  |  |  |  |  |  |  |  |  |  |  |  |  |  |  |  |  |  |  |  |  |  |  |  |  |  |  |  |  |  |  |  |  |  |  |  |  |  |  |  |  |  |  |  |  |  |  |  |  |  |  |  |  |  |  |  |  |  |  |  |  |  |  |  |  |  |  |  |  |  |  |  |  |  |  |  |  |  |  |  |  |  |  |  |  |  |  |  |  |  |  |  |  |  |  |  |  |  |  |  |  |  |  |  |  |  |  |  |  |  |  |  |  |  |  |  |  |  |  |  |  |  |  |  |  |  |  |  |  |  |  |  |  |  |  |  |  |  |  |  |  |  |  |  |  |  |  |  |  |  |  |  |  |  |  |  |  |  |  |  |  |  |  |  |  |  |  |  |  |  |  |  |  |  |  |  |  |  |  |  |  |  |  |  |  |  |  |  |  |  |  |  |  |  |  |  |  |  |  |  |  |  |  |  |  |  |  |  |  |  |  |  |  |  |  |  |  |  |  |  |  |  |  |  |  |  |  |  |  |  |  |  |  |  |  |  |  |  |  |  |  |  |  |  |  |  |  |  |  |  |  |  |  |  |  |  |  |  |  |  |  |  |  |  |  |  |  |  |  |  |  |  |  |  |  |  |  |  |  |  |  |  |  |  |  |  |  |  |  |  |  |  |  |  |  |  |  |  |  |  |  |  |  |  |  |  |  |  |  |  |  |  |  |  |  |
| --- | --- | --- | --- | --- | --- | --- | --- | --- | --- | --- | --- | --- | --- | --- | --- | --- | --- | --- | --- | --- | --- | --- | --- | --- | --- | --- | --- | --- | --- | --- | --- | --- | --- | --- | --- | --- | --- | --- | --- | --- | --- | --- | --- | --- | --- | --- | --- | --- | --- | --- | --- | --- | --- | --- | --- | --- | --- | --- | --- | --- | --- | --- | --- | --- | --- | --- | --- | --- | --- | --- | --- | --- | --- | --- | --- | --- | --- | --- | --- | --- | --- | --- | --- | --- | --- | --- | --- | --- | --- | --- | --- | --- | --- | --- | --- | --- | --- | --- | --- | --- | --- | --- | --- | --- | --- | --- | --- | --- | --- | --- | --- | --- | --- | --- | --- | --- | --- | --- | --- | --- | --- | --- | --- | --- | --- | --- | --- | --- | --- | --- | --- | --- | --- | --- | --- | --- | --- | --- | --- | --- | --- | --- | --- | --- | --- | --- | --- | --- | --- | --- | --- | --- | --- | --- | --- | --- | --- | --- | --- | --- | --- | --- | --- | --- | --- | --- | --- | --- | --- | --- | --- | --- | --- | --- | --- | --- | --- | --- | --- | --- | --- | --- | --- | --- | --- | --- | --- | --- | --- | --- | --- | --- | --- | --- | --- | --- | --- | --- | --- | --- | --- | --- | --- | --- | --- | --- | --- | --- | --- | --- | --- | --- | --- | --- | --- | --- | --- | --- | --- | --- | --- | --- | --- | --- | --- | --- | --- | --- | --- | --- | --- | --- | --- | --- | --- | --- | --- | --- | --- | --- | --- | --- | --- | --- | --- | --- | --- | --- | --- | --- | --- | --- | --- | --- | --- | --- | --- | --- | --- | --- | --- | --- | --- | --- | --- | --- | --- | --- | --- | --- | --- | --- | --- | --- | --- | --- | --- | --- | --- | --- | --- | --- | --- | --- | --- | --- | --- | --- | --- | --- | --- | --- | --- | --- | --- | --- | --- | --- | --- | --- | --- | --- | --- | --- | --- | --- | --- | --- | --- | --- | --- | --- | --- | --- | --- | --- | --- | --- | --- | --- | --- | --- | --- | --- | --- | --- | --- | --- | --- | --- | --- | --- | --- | --- | --- | --- | --- | --- | --- | --- | --- | --- | --- | --- | --- | --- | --- | --- | --- | --- | --- | --- | --- | --- | --- | --- | --- | --- | --- | --- | --- | --- | --- | --- | --- | --- | --- | --- | --- | --- | --- | --- | --- | --- | --- | --- | --- | --- | --- | --- | --- | --- | --- | --- | --- | --- | --- | --- | --- | --- | --- | --- | --- | --- | --- | --- | --- | --- | --- | --- | --- | --- | --- | --- | --- | --- | --- | --- | --- | --- | --- | --- | --- | --- | --- | --- | --- | --- | --- | --- | --- | --- | --- | --- | --- | --- | --- | --- | --- | --- | --- | --- | --- | --- | --- | --- | --- | --- | --- | --- | --- | --- | --- | --- | --- | --- | --- | --- | --- | --- | --- | --- | --- | --- | --- | --- | --- | --- | --- | --- | --- | --- | --- | --- | --- | --- | --- | --- | --- | --- | --- | --- | --- | --- | --- | --- | --- | --- | --- | --- | --- | --- | --- | --- | --- | --- | --- | --- | --- | --- | --- | --- | --- | --- | --- | --- | --- | --- | --- | --- | --- | --- | --- | --- | --- | --- | --- | --- | --- | --- | --- | --- | --- | --- | --- | --- | --- | --- | --- | --- | --- | --- | --- | --- | --- | --- | --- | --- | --- | --- |
| DCCM | A  Glu  4 | A  Asp  5 | A  Ala  6 | A  Glu  7 | A  Leu  8 | A  Leu  9 | A  Val  10 | A  Thr  11 | A  Val  12 | A  Arg  13 | A  Gly  14 | A  Gly  15 | A  Arg  16 | A  Leu  17 | A  Arg  18 | A  Gly  19 | A  Ile  20 | A  Arg  21 | A  Leu  22 | A  Lys  23 | A  Thr  24 | A  Pro  25 | A  Gly  26 | A  Gly  27 | A  Pro  28 | A  Val  29 | A  Ser  30 | A  Ala  31 | A  Phe  32 | A  Leu  33 | A  Gly  34 | A  Ile  35 | A  Pro  36 | A  Phe  37 | A  Ala  38 | A  Glu  39 | A  Pro  40 | A  Pro  41 | A  Met  42 | A  Gly  43 | A  Pro  44 | A  Arg  45 | A  Arg  46 | A  Phe  47 | A  Leu  48 | A  Pro  49 | A  Pro  50 | A  Glu  51 | A  Pro  52 | A  Lys  53 | A  Gln  54 | A  Pro  55 | A  Trp  56 | A  Ser  57 | A  Gly  58 | A  Val  59 | A  Val  60 | A  Asp  61 | A  Ala  62 | A  Thr  63 | A  Thr  64 | A  Phe  65 | A  Gln  66 | A  Ser  67 | A  Val  68 | A  Cys  69 | A  Tyr  70 | A  Gln  71 | A  Tyr  72 | A  Val  73 | A  Asp  74 | A  Thr  75 | A  Leu  76 | A  Tyr  77 | A  Pro  78 | A  Gly  79 | A  Phe  80 | A  Glu  81 | A  Gly  82 | A  Thr  83 | A  Glu  84 | A  Met  85 | A  Trp  86 | A  Asn  87 | A  Pro  88 | A  Asn  89 | A  Arg  90 | A  Glu  91 | A  Leu  92 | A  Ser  93 | A  Glu  94 | A  Asp  95 | A  Cys  96 | A  Leu  97 | A  Tyr  98 | A  Leu  99 | A  Asn  100 | A  Val  101 | A  Trp  102 | A  Thr  103 | A  Pro  104 | A  Tyr  105 | A  Pro  106 | A  Arg  107 | A  Pro  108 | A  Thr  109 | A  Ser  110 | A  Pro  111 | A  Thr  112 | A  Pro  113 | A  Val  114 | A  Leu  115 | A  Val  116 | A  Trp  117 | A  Ile  118 | A  Tyr  119 | A  Gly  120 | A  Gly  121 | A  Gly  122 | A  Phe  123 | A  Tyr  124 | A  Ser  125 | A  Gly  126 | A  Ala  127 | A  Ser  128 | A  Ser  129 | A  Leu  130 | A  Asp  131 | A  Val  132 | A  Tyr  133 | A  Asp  134 | A  Gly  135 | A  Arg  136 | A  Phe  137 | A  Leu  138 | A  Val  139 | A  Gln  140 | A  Ala  141 | A  Glu  142 | A  Arg  143 | A  Thr  144 | A  Val  145 | A  Leu  146 | A  Val  147 | A  Ser  148 | A  Met  149 | A  Asn  150 | A  Tyr  151 | A  Arg  152 | A  Val  153 | A  Gly  154 | A  Ala  155 | A  Phe  156 | A  Gly  157 | A  Phe  158 | A  Leu  159 | A  Ala  160 | A  Leu  161 | A  Pro  162 | A  Gly  163 | A  Ser  164 | A  Arg  165 | A  Glu  166 | A  Ala  167 | A  Pro  168 | A  Gly  169 | A  Asn  170 | A  Val  171 | A  Gly  172 | A  Leu  173 | A  Leu  174 | A  Asp  175 | A  Gln  176 | A  Arg  177 | A  Leu  178 | A  Ala  179 | A  Leu  180 | A  Gln  181 | A  Trp  182 | A  Val  183 | A  Gln  184 | A  Glu  185 | A  Asn  186 | A  Val  187 | A  Ala  188 | A  Ala  189 | A  Phe  190 | A  Gly  191 | A  Gly  192 | A  Asp  193 | A  Pro  194 | A  Thr  195 | A  Ser  196 | A  Val  197 | A  Thr  198 | A  Leu  199 | A  Phe  200 | A  Gly  201 | A  Glu  202 | A  Ser  203 | A  Ala  204 | A  Gly  205 | A  Ala  206 | A  Ala  207 | A  Ser  208 | A  Val  209 | A  Gly  210 | A  Met  211 | A  His  212 | A  Leu  213 | A  Leu  214 | A  Ser  215 | A  Pro  216 | A  Pro  217 | A  Ser  218 | A  Arg  219 | A  Gly  220 | A  Leu  221 | A  Phe  222 | A  His  223 | A  Arg  224 | A  Ala  225 | A  Val  226 | A  Leu  227 | A  Gln  228 | A  Ser  229 | A  Gly  230 | A  Ala  231 | A  Pro  232 | A  Asn  233 | A  Gly  234 | A  Pro  235 | A  Trp  236 | A  Ala  237 | A  Thr  238 | A  Val  239 | A  Gly  240 | A  Met  241 | A  Gly  242 | A  Glu  243 | A  Ala  244 | A  Arg  245 | A  Arg  246 | A  Arg  247 | A  Ala  248 | A  Thr  249 | A  Gln  250 | A  Leu  251 | A  Ala  252 | A  His  253 | A  Leu  254 | A  Val  255 | A  Gly  256 | A  Cys  257 | A  Pro  258 | A  Asn  265 | A  Asp  266 | A  Thr  267 | A  Glu  268 | A  Leu  269 | A  Val  270 | A  Ala  271 | A  Cys  272 | A  Leu  273 | A  Arg  274 | A  Thr  275 | A  Arg  276 | A  Pro  277 | A  Ala  278 | A  Gln  279 | A  Val  280 | A  Leu  281 | A  Val  282 | A  Asn  283 | A  His  284 | A  Glu  285 | A  Trp  286 | A  His  287 | A  Val  288 | A  Leu  289 | A  Pro  290 | A  Gln  291 | A  Glu  292 | A  Ser  293 | A  Val  294 | A  Phe  295 | A  Arg  296 | A  Phe  297 | A  Ser  298 | A  Phe  299 | A  Val  300 | A  Pro  301 | A  Val  302 | A  Val  303 | A  Asp  304 | A  Gly  305 | A  Asp  306 | A  Phe  307 | A  Leu  308 | A  Ser  309 | A  Asp  310 | A  Thr  311 | A  Pro  312 | A  Glu  313 | A  Ala  314 | A  Leu  315 | A  Ile  316 | A  Asn  317 | A  Ala  318 | A  Gly  319 | A  Asp  320 | A  Phe  321 | A  His  322 | A  Gly  323 | A  Leu  324 | A  Gln  325 | A  Val  326 | A  Leu  327 | A  Val  328 | A  Gly  329 | A  Val  330 | A  Val  331 | A  Lys  332 | A  Asp  333 | A  Glu  334 | A  Gly  335 | A  Ser  336 | A  Tyr  337 | A  Phe  338 | A  Leu  339 | A  Val  340 | A  Tyr  341 | A  Gly  342 | A  Ala  343 | A  Pro  344 | A  Gly  345 | A  Phe  346 | A  Ser  347 | A  Lys  348 | A  Asp  349 | A  Asn  350 | A  Glu  351 | A  Ser  352 | A  Leu  353 | A  Ile  354 | A  Ser  355 | A  Arg  356 | A  Ala  357 | A  Glu  358 | A  Phe  359 | A  Leu  360 | A  Ala  361 | A  Gly  362 | A  Val  363 | A  Arg  364 | A  Val  365 | A  Gly  366 | A  Val  367 | A  Pro  368 | A  Gln  369 | A  Val  370 | A  Ser  371 | A  Asp  372 | A  Leu  373 | A  Ala  374 | A  Ala  375 | A  Glu  376 | A  Ala  377 | A  Val  378 | A  Val  379 | A  Leu  380 | A  His  381 | A  Tyr  382 | A  Thr  383 | A  Asp  384 | A  Trp  385 | A  Leu  386 | A  His  387 | A  Pro  388 | A  Glu  389 | A  Asp  390 | A  Pro  391 | A  Ala  392 | A  Arg  393 | A  Leu  394 | A  Arg  395 | A  Glu  396 | A  Ala  397 | A  Leu  398 | A  Ser  399 | A  Asp  400 | A  Val  401 | A  Val  402 | A  Gly  403 | A  Asp  404 | A  His  405 | A  Asn  406 | A  Val  407 | A  Val  408 | A  Cys  409 | A  Pro  410 | A  Val  411 | A  Ala  412 | A  Gln  413 | A  Leu  414 | A  Ala  415 | A  Gly  416 | A  Arg  417 | A  Leu  418 | A  Ala  419 | A  Ala  420 | A  Gln  421 | A  Gly  422 | A  Ala  423 | A  Arg  424 | A  Val  425 | A  Tyr  426 | A  Ala  427 | A  Tyr  428 | A  Val  429 | A  Phe  430 | A  Glu  431 | A  His  432 | A  Arg  433 | A  Ala  434 | A  Ser  435 | A  Thr  436 | A  Leu  437 | A  Ser  438 | A  Trp  439 | A  Pro  440 | A  Leu  441 | A  Trp  442 | A  Met  443 | A  Gly  444 | A  Val  445 | A  Pro  446 | A  His  447 | A  Gly  448 | A  Tyr  449 | A  Glu  450 | A  Ile  451 | A  Glu  452 | A  Phe  453 | A  Ile  454 | A  Phe  455 | A  Gly  456 | A  Ile  457 | A  Pro  458 | A  Leu  459 | A  Asp  460 | A  Pro  461 | A  Ser  462 | A  Arg  463 | A  Asn  464 | A  Tyr  465 | A  Thr  466 | A  Ala  467 | A  Glu  468 | A  Glu  469 | A  Lys  470 | A  Ile  471 | A  Phe  472 | A  Ala  473 | A  Gln  474 | A  Arg  475 | A  Leu  476 | A  Met  477 | A  Arg  478 | A  Tyr  479 | A  Trp  480 | A  Ala  481 | A  Asn  482 | A  Phe  483 | A  Ala  484 | A  Arg  485 | A  Thr  486 | A  Gly  487 | A  Asp  488 | A  Pro  489 | A  Asn  490 | A  Glu  491 | A  Pro  492 | A  Arg  493 | A  Asp  494 | A  Pro  498 | A  Gln  499 | A  Trp  500 | A  Pro  501 | A  Pro  502 | A  Tyr  503 | A  Thr  504 | A  Ala  505 | A  Gly  506 | A  Ala  507 | A  Gln  508 | A  Gln  509 | A  Tyr  510 | A  Val  511 | A  Ser  512 | A  Leu  513 | A  Asp  514 | A  Leu  515 | A  Arg  516 | A  Pro  517 | A  Leu  518 | A  Glu  519 | A  Val  520 | A  Arg  521 | A  Arg  522 | A  Gly  523 | A  Leu  524 | A  Arg  525 | A  Ala  526 | A  Gln  527 | A  Ala  528 | A  Cys  529 | A  Ala  530 | A  Phe  531 | A  Trp  532 | A  Asn  533 | A  Arg  534 | A  Phe  535 | A  Leu  536 | A  Pro  537 | A  Lys  538 | A  Leu  539 | A  Leu  540 | A  Ser  541 | A  Ala  542 |
| A-Glu-4 | 1.00 | 0.57 | 0.31 | 0.23 | 0.24 | 0.21 | 0.13 | 0.05 | -0.01 | -0.03 | -0.02 | 0.04 | 0.11 | 0.16 | 0.16 | 0.17 | 0.14 | 0.15 | 0.10 | 0.07 | -0.03 | -0.03 | -0.04 | 0.01 | 0.10 | 0.13 | 0.12 | 0.09 | 0.11 | 0.09 | 0.04 | -0.02 | -0.05 | -0.05 | -0.10 | -0.09 | -0.08 | -0.13 | -0.11 | -0.07 | -0.05 | -0.05 | -0.04 | -0.03 | -0.05 | -0.06 | -0.08 | -0.08 | -0.07 | -0.06 | -0.04 | -0.02 | 0.01 | 0.04 | 0.09 | 0.11 | 0.10 | 0.20 | 0.12 | 0.10 | 0.09 | 0.01 | -0.02 | -0.06 | -0.07 | -0.12 | -0.13 | -0.09 | -0.09 | -0.01 | 0.00 | -0.04 | 0.01 | -0.03 | -0.04 | -0.03 | -0.01 | 0.09 | 0.08 | 0.05 | 0.07 | 0.06 | 0.06 | 0.00 | 0.03 | -0.10 | -0.09 | -0.08 | -0.07 | -0.10 | -0.13 | -0.09 | -0.09 | -0.09 | -0.07 | -0.10 | -0.04 | -0.03 | 0.06 | 0.06 | 0.07 | 0.10 | 0.10 | 0.11 | 0.05 | 0.00 | 0.02 | 0.00 | 0.01 | -0.07 | -0.09 | -0.07 | -0.07 | -0.08 | -0.06 | -0.06 | -0.09 | -0.10 | -0.05 | -0.05 | -0.06 | -0.07 | -0.03 | -0.02 | -0.04 | 0.00 | 0.02 | -0.02 | 0.04 | -0.01 | -0.03 | -0.03 | 0.01 | -0.04 | -0.09 | -0.07 | -0.09 | -0.02 | -0.06 | 0.02 | 0.07 | 0.05 | 0.02 | -0.05 | -0.05 | -0.04 | -0.05 | -0.06 | -0.08 | -0.03 | -0.00 | 0.00 | 0.02 | -0.01 | -0.03 | 0.01 | -0.01 | -0.02 | -0.08 | -0.06 | -0.07 | -0.06 | -0.09 | -0.06 | -0.06 | -0.05 | -0.11 | -0.06 | -0.07 | -0.05 | -0.07 | -0.06 | -0.07 | -0.06 | -0.07 | -0.05 | -0.07 | -0.06 | -0.04 | -0.04 | -0.03 | -0.01 | 0.02 | 0.01 | 0.03 | 0.06 | 0.05 | 0.03 | -0.00 | -0.01 | -0.00 | -0.04 | -0.08 | -0.05 | -0.10 | -0.07 | -0.08 | -0.08 | -0.06 | -0.05 | -0.06 | -0.04 | -0.05 | -0.06 | -0.05 | -0.04 | -0.03 | -0.06 | -0.10 | -0.08 | -0.04 | -0.02 | -0.01 | -0.02 | -0.02 | -0.02 | 0.04 | -0.03 | -0.03 | -0.04 | -0.06 | -0.04 | -0.05 | -0.07 | -0.02 | -0.01 | -0.06 | -0.07 | -0.07 | -0.08 | -0.05 | -0.02 | -0.01 | -0.03 | -0.07 | -0.08 | -0.12 | -0.15 | -0.12 | -0.11 | -0.11 | -0.11 | -0.10 | -0.07 | -0.05 | -0.06 | -0.05 | -0.02 | -0.01 | -0.01 | -0.04 | -0.03 | -0.01 | 0.01 | 0.05 | -0.19 | -0.18 | -0.13 | -0.08 | -0.05 | -0.04 | -0.02 | -0.03 | -0.03 | -0.04 | -0.11 | -0.07 | -0.02 | -0.01 | 0.01 | -0.02 | -0.01 | -0.02 | -0.05 | 0.06 | 0.07 | -0.02 | -0.06 | -0.04 | -0.07 | -0.08 | -0.09 | 0.05 | 0.06 | 0.09 | 0.09 | 0.04 | 0.01 | -0.04 | -0.06 | -0.05 | -0.09 | -0.02 | -0.02 | 0.02 | 0.01 | -0.04 | -0.05 | -0.03 | 0.02 | -0.02 | -0.03 | -0.07 | -0.04 | 0.01 | 0.02 | -0.00 | 0.01 | 0.05 | 0.07 | 0.06 | -0.00 | -0.04 | -0.02 | 0.03 | -0.02 | -0.05 | -0.08 | -0.03 | -0.01 | -0.03 | -0.02 | 0.04 | 0.04 | 0.00 | 0.01 | 0.08 | 0.06 | 0.06 | 0.01 | -0.06 | -0.01 | -0.02 | 0.10 | 0.06 | 0.03 | 0.05 | 0.04 | 0.02 | -0.06 | -0.04 | -0.09 | -0.03 | -0.04 | 0.01 | 0.01 | 0.02 | 0.05 | 0.09 | 0.10 | 0.09 | 0.13 | 0.18 | 0.17 | 0.13 | 0.12 | 0.16 | 0.06 | -0.01 | -0.07 | -0.01 | 0.04 | 0.06 | 0.07 | 0.08 | 0.06 | 0.06 | 0.07 | 0.07 | 0.04 | 0.05 | 0.05 | -0.03 | -0.01 | -0.02 | -0.04 | -0.03 | -0.05 | -0.01 | -0.00 | -0.11 | -0.10 | -0.09 | -0.08 | -0.01 | 0.02 | 0.01 | 0.02 | 0.07 | 0.02 | 0.00 | 0.01 | 0.04 | 0.01 | 0.03 | 0.02 | 0.01 | 0.02 | -0.02 | -0.03 | -0.01 | 0.01 | -0.07 | -0.07 | -0.01 | 0.02 | -0.04 | 0.00 | 0.02 | -0.02 | -0.02 | -0.05 | -0.05 | -0.01 | -0.05 | 0.01 | 0.02 | -0.00 | -0.00 | 0.02 | -0.01 | -0.02 | -0.03 | -0.00 | -0.16 | -0.08 | 0.00 | -0.13 | -0.14 | -0.10 | -0.17 | -0.25 | -0.25 | -0.15 | -0.06 | -0.05 | 0.03 | -0.04 | -0.01 | -0.02 | 0.03 | 0.04 | 0.02 | 0.06 | -0.01 | -0.03 | -0.09 | -0.10 | 0.00 | -0.03 | -0.03 | -0.11 | -0.10 | -0.10 | 0.07 | 0.08 | 0.10 | 0.10 | 0.09 | 0.05 | 0.03 | 0.03 | -0.01 | -0.05 | -0.09 | -0.10 | -0.07 | -0.11 | -0.13 | -0.13 | -0.12 | -0.13 | -0.14 | -0.10 | -0.08 | -0.09 | -0.05 | -0.06 | -0.05 | -0.06 | -0.05 | 0.02 | -0.01 | -0.06 | -0.03 | -0.01 | -0.03 | -0.05 | -0.00 | -0.04 | 0.02 | 0.06 | 0.11 | 0.04 | 0.02 | 0.04 | 0.06 | 0.03 | 0.01 | 0.01 | -0.03 | -0.05 | -0.03 | -0.02 | -0.02 | -0.06 | -0.03 | 0.04 | 0.10 | 0.10 | 0.11 | 0.11 | 0.13 | 0.19 | 0.12 | 0.12 | 0.09 | 0.07 | 0.08 | 0.07 | 0.08 | 0.10 | 0.10 | 0.09 | 0.05 | 0.05 | 0.05 | 0.04 | 0.03 | -0.02 |
| A-Asp-5 | 0.57 | 1.00 | 0.70 | 0.55 | 0.50 | 0.40 | 0.29 | 0.18 | 0.09 | 0.02 | 0.05 | 0.14 | 0.21 | 0.29 | 0.30 | 0.33 | 0.31 | 0.30 | 0.20 | 0.13 | 0.05 | -0.00 | -0.01 | 0.07 | 0.16 | 0.22 | 0.25 | 0.20 | 0.24 | 0.16 | 0.09 | 0.06 | -0.03 | -0.03 | -0.10 | -0.10 | -0.07 | -0.20 | -0.13 | -0.12 | -0.13 | -0.16 | -0.15 | -0.15 | -0.14 | -0.13 | -0.12 | -0.06 | -0.04 | -0.03 | 0.02 | 0.06 | 0.10 | 0.12 | 0.19 | 0.23 | 0.18 | 0.21 | 0.20 | 0.23 | 0.11 | 0.03 | -0.00 | -0.06 | -0.08 | -0.13 | -0.16 | -0.13 | -0.11 | -0.12 | -0.09 | -0.12 | -0.12 | -0.04 | -0.04 | -0.07 | -0.05 | -0.02 | -0.01 | -0.05 | -0.08 | -0.10 | -0.12 | -0.16 | -0.11 | 0.01 | -0.08 | -0.10 | -0.13 | -0.14 | -0.16 | -0.16 | -0.13 | -0.12 | -0.07 | -0.06 | 0.05 | 0.05 | 0.15 | 0.17 | 0.22 | 0.31 | 0.36 | 0.31 | 0.19 | 0.09 | 0.10 | 0.07 | 0.09 | -0.01 | -0.04 | -0.06 | -0.06 | -0.09 | -0.09 | -0.10 | -0.09 | -0.15 | -0.15 | -0.17 | -0.13 | -0.07 | -0.01 | -0.08 | -0.05 | 0.02 | -0.02 | -0.00 | 0.02 | 0.02 | 0.06 | 0.04 | 0.11 | 0.06 | 0.03 | 0.05 | -0.00 | 0.02 | 0.02 | 0.06 | 0.08 | 0.07 | 0.05 | -0.01 | -0.04 | -0.09 | -0.11 | -0.13 | -0.17 | -0.13 | -0.14 | -0.14 | -0.10 | -0.11 | -0.14 | -0.10 | -0.10 | -0.06 | -0.02 | -0.05 | -0.10 | -0.09 | -0.12 | -0.10 | -0.13 | -0.09 | -0.16 | -0.16 | -0.13 | -0.13 | -0.13 | -0.11 | -0.10 | -0.09 | -0.07 | -0.01 | -0.03 | -0.00 | 0.01 | 0.03 | 0.02 | 0.06 | 0.12 | 0.13 | 0.14 | 0.18 | 0.17 | 0.12 | 0.07 | 0.03 | 0.05 | 0.01 | -0.03 | -0.02 | -0.10 | -0.07 | -0.10 | -0.12 | -0.12 | -0.14 | -0.11 | -0.14 | -0.15 | -0.15 | -0.15 | -0.13 | -0.12 | -0.13 | -0.12 | -0.11 | -0.07 | -0.03 | -0.01 | -0.01 | -0.02 | -0.02 | 0.02 | 0.02 | -0.02 | -0.04 | -0.10 | -0.10 | -0.13 | -0.18 | -0.14 | -0.20 | -0.18 | -0.17 | -0.16 | -0.13 | -0.11 | -0.08 | -0.10 | -0.13 | -0.11 | -0.08 | -0.07 | -0.12 | -0.08 | -0.08 | -0.10 | -0.09 | -0.09 | -0.11 | -0.10 | -0.07 | -0.08 | -0.08 | -0.06 | -0.05 | -0.07 | -0.05 | -0.04 | -0.03 | 0.02 | -0.08 | -0.11 | -0.13 | -0.16 | -0.13 | -0.13 | -0.11 | -0.11 | -0.14 | -0.14 | -0.14 | -0.14 | -0.06 | -0.06 | -0.05 | -0.09 | -0.06 | -0.05 | -0.08 | -0.07 | -0.10 | -0.10 | -0.08 | -0.07 | -0.06 | -0.03 | -0.02 | 0.02 | 0.08 | 0.09 | 0.00 | -0.04 | -0.05 | -0.10 | -0.19 | -0.15 | -0.15 | -0.10 | -0.11 | -0.07 | -0.06 | -0.05 | -0.06 | -0.11 | -0.05 | -0.08 | -0.07 | -0.11 | -0.06 | -0.07 | -0.08 | -0.06 | -0.05 | -0.04 | -0.02 | -0.00 | -0.05 | -0.02 | 0.02 | 0.03 | -0.06 | -0.15 | -0.17 | -0.20 | -0.20 | -0.20 | -0.22 | -0.17 | -0.15 | -0.18 | -0.15 | -0.13 | -0.17 | -0.13 | -0.01 | 0.07 | 0.02 | 0.01 | 0.02 | -0.00 | 0.06 | 0.07 | 0.05 | -0.02 | 0.03 | 0.08 | 0.04 | 0.09 | 0.11 | 0.17 | 0.20 | 0.23 | 0.26 | 0.25 | 0.25 | 0.28 | 0.26 | 0.24 | 0.26 | 0.24 | 0.17 | 0.19 | 0.18 | 0.15 | 0.12 | 0.19 | 0.19 | 0.19 | 0.18 | 0.21 | 0.23 | 0.24 | 0.24 | 0.25 | 0.22 | 0.22 | 0.21 | 0.12 | 0.14 | 0.09 | 0.04 | 0.06 | 0.11 | 0.14 | 0.20 | 0.11 | 0.10 | 0.03 | 0.05 | 0.15 | 0.09 | 0.04 | 0.08 | 0.10 | -0.06 | -0.11 | -0.02 | -0.08 | -0.18 | -0.11 | -0.05 | -0.13 | -0.17 | -0.19 | -0.12 | -0.09 | -0.10 | -0.12 | -0.12 | -0.11 | -0.08 | -0.08 | -0.08 | -0.02 | -0.03 | -0.04 | -0.03 | -0.03 | 0.00 | -0.10 | -0.10 | -0.09 | -0.20 | -0.22 | -0.18 | -0.18 | -0.17 | -0.13 | -0.03 | -0.06 | 0.02 | 0.00 | -0.14 | -0.14 | -0.12 | -0.09 | -0.19 | -0.17 | -0.20 | -0.01 | -0.16 | -0.10 | -0.19 | -0.20 | -0.11 | -0.04 | -0.07 | -0.04 | 0.03 | 0.03 | 0.03 | -0.00 | -0.05 | -0.03 | -0.02 | 0.00 | -0.05 | -0.02 | -0.08 | -0.05 | -0.08 | -0.08 | -0.04 | -0.05 | -0.08 | -0.04 | -0.02 | -0.03 | -0.04 | -0.05 | -0.06 | -0.00 | -0.02 | -0.04 | -0.05 | -0.05 | -0.05 | -0.09 | -0.11 | -0.09 | -0.07 | -0.08 | -0.11 | -0.08 | -0.05 | -0.04 | -0.04 | -0.08 | -0.12 | -0.14 | -0.18 | -0.09 | -0.09 | -0.11 | -0.14 | -0.14 | -0.03 | 0.02 | -0.09 | -0.09 | -0.12 | -0.12 | -0.18 | -0.19 | -0.14 | -0.12 | -0.10 | -0.08 | -0.10 | -0.04 | -0.13 | -0.14 | -0.14 | -0.11 | -0.11 | -0.08 | -0.09 | -0.07 | 0.05 | 0.01 | -0.09 | -0.04 | 0.12 | 0.18 | 0.12 | 0.08 | 0.16 | 0.18 | 0.16 | 0.10 | 0.12 | 0.12 | 0.08 | 0.04 | -0.05 |
| A-Ala-6 | 0.31 | 0.70 | 1.00 | 0.78 | 0.60 | 0.55 | 0.44 | 0.32 | 0.21 | 0.14 | 0.14 | 0.25 | 0.33 | 0.39 | 0.43 | 0.43 | 0.38 | 0.37 | 0.20 | 0.14 | 0.05 | -0.01 | 0.01 | 0.10 | 0.19 | 0.25 | 0.28 | 0.22 | 0.30 | 0.20 | 0.12 | 0.09 | 0.00 | -0.03 | -0.08 | -0.09 | -0.07 | -0.21 | -0.14 | -0.13 | -0.15 | -0.21 | -0.19 | -0.18 | -0.19 | -0.15 | -0.13 | -0.08 | -0.06 | -0.02 | 0.08 | 0.13 | 0.16 | 0.20 | 0.21 | 0.28 | 0.23 | 0.28 | 0.28 | 0.26 | 0.13 | 0.05 | 0.02 | -0.03 | -0.04 | -0.09 | -0.12 | -0.11 | -0.07 | -0.09 | -0.12 | -0.12 | -0.15 | -0.05 | -0.06 | -0.10 | -0.06 | -0.03 | -0.01 | -0.09 | -0.12 | -0.15 | -0.17 | -0.17 | -0.10 | -0.01 | -0.05 | -0.08 | -0.12 | -0.13 | -0.15 | -0.14 | -0.12 | -0.11 | -0.07 | -0.00 | 0.06 | 0.08 | 0.18 | 0.23 | 0.29 | 0.39 | 0.49 | 0.43 | 0.28 | 0.11 | 0.16 | 0.10 | 0.14 | 0.04 | 0.02 | -0.04 | -0.03 | -0.06 | -0.08 | -0.07 | -0.05 | -0.14 | -0.15 | -0.15 | -0.14 | -0.09 | -0.02 | -0.11 | -0.06 | -0.04 | -0.06 | -0.01 | -0.04 | 0.03 | 0.07 | 0.03 | 0.09 | 0.05 | 0.05 | 0.08 | 0.01 | 0.01 | -0.00 | 0.05 | 0.08 | 0.10 | 0.06 | 0.02 | 0.01 | -0.09 | -0.12 | -0.12 | -0.16 | -0.11 | -0.13 | -0.11 | -0.10 | -0.10 | -0.14 | -0.11 | -0.12 | -0.07 | -0.08 | -0.09 | -0.10 | -0.07 | -0.09 | -0.10 | -0.15 | -0.13 | -0.18 | -0.22 | -0.14 | -0.15 | -0.16 | -0.12 | -0.10 | -0.10 | -0.08 | -0.02 | -0.04 | 0.00 | 0.04 | 0.04 | 0.07 | 0.11 | 0.17 | 0.16 | 0.19 | 0.24 | 0.25 | 0.16 | 0.10 | 0.08 | 0.09 | 0.05 | 0.03 | 0.03 | -0.05 | -0.02 | -0.08 | -0.11 | -0.13 | -0.17 | -0.14 | -0.16 | -0.18 | -0.16 | -0.14 | -0.14 | -0.13 | -0.15 | -0.13 | -0.12 | -0.13 | -0.08 | -0.03 | -0.01 | -0.03 | -0.05 | 0.03 | 0.05 | 0.01 | -0.00 | -0.07 | -0.09 | -0.14 | -0.20 | -0.19 | -0.26 | -0.22 | -0.17 | -0.17 | -0.14 | -0.10 | -0.03 | -0.06 | -0.12 | -0.13 | -0.11 | -0.11 | -0.17 | -0.09 | -0.10 | -0.14 | -0.13 | -0.10 | -0.12 | -0.13 | -0.09 | -0.09 | -0.10 | -0.08 | -0.05 | -0.07 | -0.07 | -0.05 | -0.01 | 0.03 | -0.10 | -0.12 | -0.15 | -0.19 | -0.12 | -0.13 | -0.11 | -0.11 | -0.13 | -0.13 | -0.11 | -0.11 | -0.07 | -0.08 | -0.07 | -0.12 | -0.10 | -0.05 | -0.07 | -0.08 | -0.09 | -0.07 | -0.07 | -0.08 | -0.05 | -0.01 | 0.02 | 0.01 | 0.07 | 0.06 | -0.03 | -0.04 | -0.08 | -0.11 | -0.20 | -0.18 | -0.20 | -0.15 | -0.13 | -0.10 | -0.07 | -0.08 | -0.09 | -0.12 | -0.07 | -0.10 | -0.09 | -0.13 | -0.10 | -0.10 | -0.09 | -0.08 | -0.07 | -0.06 | -0.02 | 0.01 | -0.07 | -0.02 | 0.02 | 0.01 | -0.03 | -0.14 | -0.18 | -0.24 | -0.24 | -0.25 | -0.26 | -0.20 | -0.17 | -0.19 | -0.13 | -0.12 | -0.17 | -0.15 | 0.02 | 0.09 | 0.01 | 0.02 | 0.04 | 0.04 | 0.12 | 0.11 | 0.09 | 0.01 | 0.06 | 0.13 | 0.09 | 0.14 | 0.18 | 0.22 | 0.25 | 0.27 | 0.29 | 0.28 | 0.27 | 0.28 | 0.25 | 0.23 | 0.27 | 0.25 | 0.17 | 0.16 | 0.19 | 0.19 | 0.14 | 0.21 | 0.22 | 0.23 | 0.21 | 0.24 | 0.27 | 0.27 | 0.27 | 0.28 | 0.27 | 0.27 | 0.25 | 0.20 | 0.21 | 0.16 | 0.14 | 0.11 | 0.16 | 0.23 | 0.25 | 0.20 | 0.19 | 0.14 | 0.16 | 0.22 | 0.15 | 0.10 | 0.13 | 0.14 | -0.07 | -0.11 | 0.02 | -0.06 | -0.21 | -0.16 | -0.08 | -0.17 | -0.21 | -0.23 | -0.18 | -0.15 | -0.16 | -0.17 | -0.16 | -0.13 | -0.13 | -0.12 | -0.09 | 0.00 | -0.03 | -0.02 | 0.04 | 0.01 | -0.00 | -0.11 | -0.13 | -0.15 | -0.26 | -0.26 | -0.23 | -0.24 | -0.23 | -0.19 | -0.03 | -0.03 | 0.01 | -0.04 | -0.19 | -0.15 | -0.12 | -0.08 | -0.17 | -0.13 | -0.15 | 0.01 | -0.19 | -0.12 | -0.22 | -0.27 | -0.17 | -0.10 | -0.13 | -0.08 | -0.03 | -0.00 | 0.03 | 0.00 | -0.04 | -0.09 | -0.06 | -0.02 | -0.04 | -0.02 | -0.07 | -0.06 | -0.10 | -0.13 | -0.10 | -0.12 | -0.15 | -0.11 | -0.09 | -0.11 | -0.08 | -0.10 | -0.11 | -0.05 | -0.06 | -0.08 | -0.10 | -0.09 | -0.08 | -0.09 | -0.12 | -0.11 | -0.07 | -0.09 | -0.12 | -0.10 | -0.06 | -0.08 | -0.08 | -0.13 | -0.19 | -0.21 | -0.22 | -0.20 | -0.18 | -0.18 | -0.16 | -0.17 | -0.11 | -0.05 | -0.11 | -0.18 | -0.20 | -0.19 | -0.23 | -0.25 | -0.22 | -0.20 | -0.18 | -0.18 | -0.17 | -0.09 | -0.18 | -0.17 | -0.15 | -0.11 | -0.08 | -0.05 | -0.06 | -0.08 | 0.06 | 0.00 | -0.07 | -0.07 | 0.08 | 0.17 | 0.08 | 0.01 | 0.09 | 0.14 | 0.12 | 0.06 | 0.09 | 0.10 | 0.07 | 0.02 | -0.03 |
| A-Glu-7 | 0.23 | 0.55 | 0.78 | 1.00 | 0.79 | 0.64 | 0.51 | 0.37 | 0.23 | 0.13 | 0.14 | 0.26 | 0.36 | 0.43 | 0.50 | 0.52 | 0.47 | 0.46 | 0.29 | 0.19 | 0.10 | 0.01 | 0.06 | 0.14 | 0.27 | 0.34 | 0.36 | 0.28 | 0.37 | 0.24 | 0.14 | 0.09 | -0.01 | -0.05 | -0.12 | -0.13 | -0.08 | -0.22 | -0.14 | -0.14 | -0.14 | -0.21 | -0.21 | -0.20 | -0.21 | -0.17 | -0.18 | -0.12 | -0.08 | -0.05 | 0.05 | 0.13 | 0.16 | 0.20 | 0.22 | 0.31 | 0.26 | 0.31 | 0.31 | 0.30 | 0.16 | 0.08 | 0.04 | -0.01 | -0.04 | -0.10 | -0.15 | -0.10 | -0.04 | -0.06 | -0.09 | -0.13 | -0.11 | -0.03 | -0.05 | -0.08 | -0.05 | -0.03 | -0.03 | -0.08 | -0.11 | -0.14 | -0.16 | -0.15 | -0.05 | -0.02 | -0.04 | -0.09 | -0.13 | -0.14 | -0.17 | -0.16 | -0.14 | -0.13 | -0.06 | -0.00 | 0.09 | 0.10 | 0.26 | 0.34 | 0.42 | 0.51 | 0.59 | 0.52 | 0.36 | 0.17 | 0.20 | 0.14 | 0.17 | 0.08 | 0.05 | -0.02 | -0.01 | -0.06 | -0.08 | -0.10 | -0.06 | -0.16 | -0.14 | -0.16 | -0.14 | -0.11 | -0.04 | -0.11 | -0.07 | -0.07 | -0.09 | -0.02 | -0.03 | 0.04 | 0.08 | 0.04 | 0.12 | 0.08 | 0.08 | 0.14 | 0.05 | 0.02 | 0.01 | 0.10 | 0.12 | 0.12 | 0.07 | 0.05 | 0.03 | -0.09 | -0.14 | -0.14 | -0.18 | -0.14 | -0.15 | -0.16 | -0.14 | -0.11 | -0.18 | -0.14 | -0.15 | -0.08 | -0.10 | -0.10 | -0.10 | -0.10 | -0.08 | -0.11 | -0.20 | -0.15 | -0.18 | -0.23 | -0.15 | -0.18 | -0.17 | -0.14 | -0.12 | -0.13 | -0.09 | -0.02 | -0.05 | 0.00 | 0.08 | 0.06 | 0.06 | 0.11 | 0.18 | 0.18 | 0.24 | 0.29 | 0.31 | 0.21 | 0.14 | 0.11 | 0.13 | 0.06 | 0.04 | 0.03 | -0.05 | -0.01 | -0.12 | -0.17 | -0.19 | -0.21 | -0.16 | -0.18 | -0.21 | -0.19 | -0.17 | -0.16 | -0.15 | -0.17 | -0.16 | -0.13 | -0.14 | -0.10 | -0.03 | 0.01 | -0.03 | -0.02 | 0.06 | 0.07 | 0.03 | 0.01 | -0.09 | -0.13 | -0.20 | -0.23 | -0.22 | -0.29 | -0.25 | -0.20 | -0.18 | -0.17 | -0.13 | -0.05 | -0.07 | -0.11 | -0.13 | -0.14 | -0.14 | -0.21 | -0.12 | -0.14 | -0.17 | -0.15 | -0.12 | -0.12 | -0.13 | -0.08 | -0.08 | -0.09 | -0.06 | -0.02 | -0.03 | -0.03 | -0.02 | -0.00 | 0.04 | -0.15 | -0.19 | -0.18 | -0.19 | -0.11 | -0.13 | -0.13 | -0.11 | -0.10 | -0.11 | -0.09 | -0.10 | -0.07 | -0.09 | -0.05 | -0.09 | -0.08 | -0.01 | -0.03 | -0.04 | -0.07 | -0.06 | -0.05 | -0.07 | -0.07 | -0.06 | -0.01 | 0.01 | 0.12 | 0.14 | 0.03 | 0.01 | -0.04 | -0.10 | -0.22 | -0.19 | -0.21 | -0.13 | -0.11 | -0.08 | -0.06 | -0.06 | -0.08 | -0.12 | -0.09 | -0.08 | -0.07 | -0.12 | -0.06 | -0.08 | -0.09 | -0.08 | -0.06 | -0.04 | -0.02 | 0.02 | -0.08 | -0.02 | 0.03 | 0.03 | -0.08 | -0.17 | -0.21 | -0.27 | -0.26 | -0.29 | -0.32 | -0.22 | -0.19 | -0.20 | -0.14 | -0.13 | -0.15 | -0.12 | 0.04 | 0.10 | 0.04 | 0.04 | 0.06 | 0.03 | 0.15 | 0.14 | 0.13 | 0.06 | 0.10 | 0.16 | 0.10 | 0.16 | 0.18 | 0.21 | 0.25 | 0.27 | 0.30 | 0.31 | 0.29 | 0.30 | 0.28 | 0.26 | 0.28 | 0.28 | 0.22 | 0.17 | 0.18 | 0.21 | 0.19 | 0.23 | 0.25 | 0.26 | 0.25 | 0.26 | 0.27 | 0.29 | 0.28 | 0.28 | 0.27 | 0.26 | 0.22 | 0.17 | 0.18 | 0.13 | 0.10 | 0.08 | 0.13 | 0.20 | 0.23 | 0.19 | 0.17 | 0.14 | 0.16 | 0.22 | 0.16 | 0.10 | 0.13 | 0.17 | -0.04 | -0.13 | -0.01 | -0.09 | -0.22 | -0.19 | -0.09 | -0.18 | -0.24 | -0.28 | -0.23 | -0.18 | -0.20 | -0.22 | -0.18 | -0.15 | -0.16 | -0.13 | -0.09 | -0.02 | -0.02 | 0.01 | 0.02 | -0.01 | -0.00 | -0.14 | -0.15 | -0.15 | -0.30 | -0.31 | -0.29 | -0.30 | -0.25 | -0.24 | -0.04 | -0.01 | 0.05 | -0.01 | -0.20 | -0.14 | -0.11 | -0.06 | -0.17 | -0.14 | -0.17 | -0.05 | -0.20 | -0.09 | -0.29 | -0.31 | -0.23 | -0.11 | -0.13 | -0.14 | -0.08 | -0.01 | 0.03 | 0.01 | -0.05 | -0.10 | -0.06 | 0.00 | -0.05 | -0.03 | -0.07 | -0.03 | -0.11 | -0.15 | -0.10 | -0.16 | -0.19 | -0.12 | -0.13 | -0.15 | -0.10 | -0.11 | -0.14 | -0.08 | -0.09 | -0.11 | -0.14 | -0.12 | -0.09 | -0.10 | -0.13 | -0.12 | -0.09 | -0.08 | -0.12 | -0.11 | -0.07 | -0.06 | -0.07 | -0.14 | -0.24 | -0.24 | -0.26 | -0.25 | -0.21 | -0.19 | -0.18 | -0.19 | -0.13 | -0.06 | -0.11 | -0.20 | -0.23 | -0.22 | -0.25 | -0.29 | -0.26 | -0.25 | -0.23 | -0.23 | -0.20 | -0.09 | -0.20 | -0.20 | -0.17 | -0.16 | -0.11 | -0.05 | -0.08 | -0.10 | 0.06 | 0.02 | -0.08 | -0.12 | 0.05 | 0.15 | 0.09 | 0.03 | 0.10 | 0.15 | 0.15 | 0.08 | 0.12 | 0.13 | 0.07 | 0.00 | -0.05 |
| A-Leu-8 | 0.24 | 0.50 | 0.60 | 0.79 | 1.00 | 0.77 | 0.62 | 0.46 | 0.32 | 0.19 | 0.21 | 0.37 | 0.49 | 0.57 | 0.68 | 0.71 | 0.63 | 0.56 | 0.38 | 0.27 | 0.14 | 0.02 | 0.04 | 0.12 | 0.28 | 0.36 | 0.45 | 0.37 | 0.50 | 0.31 | 0.21 | 0.16 | 0.02 | -0.02 | -0.08 | -0.11 | -0.07 | -0.22 | -0.14 | -0.13 | -0.15 | -0.19 | -0.18 | -0.17 | -0.17 | -0.16 | -0.15 | -0.10 | -0.06 | -0.01 | 0.10 | 0.20 | 0.24 | 0.27 | 0.29 | 0.43 | 0.37 | 0.45 | 0.45 | 0.43 | 0.25 | 0.15 | 0.10 | 0.02 | -0.02 | -0.12 | -0.15 | -0.12 | -0.08 | -0.10 | -0.12 | -0.15 | -0.12 | -0.07 | -0.06 | -0.08 | -0.05 | -0.02 | -0.03 | -0.09 | -0.12 | -0.16 | -0.17 | -0.16 | -0.08 | -0.04 | -0.04 | -0.07 | -0.11 | -0.15 | -0.20 | -0.16 | -0.14 | -0.10 | -0.01 | 0.04 | 0.14 | 0.18 | 0.30 | 0.35 | 0.37 | 0.45 | 0.48 | 0.42 | 0.28 | 0.17 | 0.17 | 0.14 | 0.16 | 0.07 | 0.03 | -0.02 | -0.00 | -0.05 | -0.10 | -0.09 | -0.07 | -0.16 | -0.15 | -0.17 | -0.12 | -0.10 | -0.04 | -0.06 | -0.03 | -0.03 | -0.04 | 0.02 | -0.02 | 0.03 | 0.06 | 0.01 | 0.11 | 0.04 | 0.01 | 0.10 | -0.00 | -0.06 | -0.09 | 0.04 | 0.09 | 0.13 | 0.11 | 0.07 | 0.04 | -0.07 | -0.11 | -0.12 | -0.16 | -0.15 | -0.14 | -0.15 | -0.13 | -0.12 | -0.18 | -0.14 | -0.12 | -0.06 | -0.08 | -0.07 | -0.08 | -0.07 | -0.07 | -0.11 | -0.17 | -0.12 | -0.17 | -0.21 | -0.16 | -0.18 | -0.17 | -0.13 | -0.13 | -0.13 | -0.09 | -0.01 | -0.04 | 0.00 | 0.09 | 0.07 | 0.05 | 0.12 | 0.22 | 0.21 | 0.24 | 0.32 | 0.33 | 0.21 | 0.14 | 0.09 | 0.12 | 0.04 | 0.03 | 0.03 | -0.04 | 0.02 | -0.10 | -0.14 | -0.16 | -0.16 | -0.15 | -0.15 | -0.17 | -0.17 | -0.18 | -0.13 | -0.16 | -0.18 | -0.18 | -0.13 | -0.14 | -0.10 | -0.04 | -0.01 | -0.04 | -0.03 | 0.04 | 0.06 | 0.00 | -0.01 | -0.07 | -0.13 | -0.20 | -0.18 | -0.17 | -0.26 | -0.22 | -0.17 | -0.15 | -0.13 | -0.11 | -0.06 | -0.10 | -0.10 | -0.12 | -0.16 | -0.15 | -0.21 | -0.13 | -0.17 | -0.20 | -0.17 | -0.17 | -0.17 | -0.15 | -0.12 | -0.13 | -0.13 | -0.10 | -0.10 | -0.11 | -0.08 | -0.06 | -0.05 | 0.00 | -0.14 | -0.17 | -0.14 | -0.14 | -0.13 | -0.16 | -0.14 | -0.14 | -0.14 | -0.14 | -0.10 | -0.12 | -0.09 | -0.09 | -0.08 | -0.13 | -0.11 | -0.05 | -0.08 | -0.12 | -0.13 | -0.11 | -0.10 | -0.12 | -0.10 | -0.11 | -0.07 | -0.04 | 0.07 | 0.06 | -0.03 | -0.04 | -0.09 | -0.15 | -0.22 | -0.20 | -0.19 | -0.10 | -0.08 | -0.04 | -0.05 | -0.06 | -0.07 | -0.09 | -0.07 | -0.05 | -0.04 | -0.09 | -0.06 | -0.07 | -0.10 | -0.10 | -0.07 | -0.05 | -0.05 | -0.01 | -0.12 | -0.06 | 0.01 | -0.01 | -0.11 | -0.17 | -0.22 | -0.23 | -0.22 | -0.26 | -0.30 | -0.19 | -0.12 | -0.16 | -0.11 | -0.11 | -0.15 | -0.15 | 0.02 | 0.07 | 0.01 | 0.00 | 0.02 | -0.00 | 0.11 | 0.11 | 0.10 | 0.01 | 0.07 | 0.13 | 0.08 | 0.11 | 0.16 | 0.19 | 0.25 | 0.27 | 0.28 | 0.28 | 0.29 | 0.29 | 0.26 | 0.24 | 0.27 | 0.25 | 0.16 | 0.16 | 0.17 | 0.19 | 0.17 | 0.19 | 0.24 | 0.27 | 0.28 | 0.26 | 0.27 | 0.30 | 0.29 | 0.30 | 0.27 | 0.26 | 0.25 | 0.20 | 0.22 | 0.17 | 0.14 | 0.13 | 0.18 | 0.24 | 0.25 | 0.22 | 0.19 | 0.15 | 0.19 | 0.23 | 0.15 | 0.11 | 0.14 | 0.16 | -0.05 | -0.10 | -0.00 | -0.12 | -0.20 | -0.13 | -0.06 | -0.16 | -0.19 | -0.22 | -0.18 | -0.14 | -0.14 | -0.15 | -0.13 | -0.12 | -0.12 | -0.10 | -0.07 | -0.03 | -0.04 | -0.01 | -0.03 | -0.04 | -0.03 | -0.14 | -0.15 | -0.15 | -0.27 | -0.27 | -0.27 | -0.29 | -0.27 | -0.27 | -0.07 | -0.02 | 0.03 | -0.05 | -0.27 | -0.21 | -0.18 | -0.13 | -0.23 | -0.19 | -0.21 | -0.02 | -0.17 | -0.15 | -0.26 | -0.27 | -0.19 | -0.08 | -0.09 | -0.11 | -0.09 | -0.03 | 0.01 | -0.03 | -0.06 | -0.12 | -0.07 | -0.01 | -0.06 | -0.01 | -0.07 | -0.04 | -0.12 | -0.14 | -0.09 | -0.14 | -0.19 | -0.13 | -0.12 | -0.17 | -0.12 | -0.11 | -0.15 | -0.11 | -0.13 | -0.15 | -0.16 | -0.14 | -0.15 | -0.18 | -0.18 | -0.15 | -0.16 | -0.16 | -0.18 | -0.18 | -0.14 | -0.13 | -0.14 | -0.16 | -0.21 | -0.26 | -0.25 | -0.27 | -0.23 | -0.22 | -0.18 | -0.16 | -0.12 | -0.05 | -0.12 | -0.21 | -0.21 | -0.20 | -0.24 | -0.28 | -0.27 | -0.27 | -0.25 | -0.24 | -0.21 | -0.13 | -0.23 | -0.23 | -0.19 | -0.16 | -0.09 | -0.02 | -0.04 | -0.02 | 0.07 | 0.05 | -0.01 | -0.07 | 0.10 | 0.18 | 0.08 | 0.03 | 0.10 | 0.17 | 0.15 | 0.06 | 0.10 | 0.11 | 0.03 | -0.03 | -0.10 |
| A-Leu-9 | 0.21 | 0.40 | 0.55 | 0.64 | 0.77 | 1.00 | 0.82 | 0.61 | 0.44 | 0.30 | 0.29 | 0.48 | 0.63 | 0.65 | 0.69 | 0.61 | 0.48 | 0.43 | 0.25 | 0.18 | 0.06 | -0.02 | -0.00 | 0.08 | 0.20 | 0.25 | 0.36 | 0.31 | 0.42 | 0.27 | 0.18 | 0.16 | 0.03 | 0.01 | -0.05 | -0.07 | -0.06 | -0.20 | -0.13 | -0.09 | -0.12 | -0.20 | -0.18 | -0.16 | -0.15 | -0.12 | -0.11 | -0.05 | -0.03 | 0.02 | 0.16 | 0.27 | 0.29 | 0.31 | 0.35 | 0.47 | 0.38 | 0.46 | 0.42 | 0.37 | 0.21 | 0.11 | 0.06 | -0.01 | -0.04 | -0.12 | -0.15 | -0.12 | -0.10 | -0.14 | -0.14 | -0.15 | -0.12 | -0.09 | -0.08 | -0.08 | -0.07 | -0.04 | -0.05 | -0.12 | -0.14 | -0.18 | -0.16 | -0.15 | -0.08 | -0.07 | -0.08 | -0.11 | -0.13 | -0.15 | -0.17 | -0.11 | -0.11 | -0.07 | 0.00 | 0.05 | 0.10 | 0.13 | 0.20 | 0.25 | 0.26 | 0.35 | 0.40 | 0.38 | 0.27 | 0.16 | 0.15 | 0.11 | 0.13 | 0.03 | 0.00 | -0.07 | -0.04 | -0.09 | -0.10 | -0.08 | -0.04 | -0.13 | -0.13 | -0.14 | -0.11 | -0.08 | -0.05 | -0.07 | -0.05 | -0.06 | -0.10 | -0.04 | -0.09 | -0.02 | -0.00 | -0.07 | 0.04 | -0.03 | -0.06 | -0.01 | -0.06 | -0.10 | -0.15 | -0.05 | 0.01 | 0.07 | 0.06 | 0.02 | -0.00 | -0.07 | -0.10 | -0.11 | -0.14 | -0.12 | -0.13 | -0.15 | -0.13 | -0.10 | -0.15 | -0.12 | -0.12 | -0.07 | -0.11 | -0.08 | -0.06 | -0.07 | -0.05 | -0.10 | -0.13 | -0.12 | -0.17 | -0.20 | -0.13 | -0.13 | -0.13 | -0.08 | -0.10 | -0.10 | -0.06 | 0.01 | -0.01 | 0.04 | 0.13 | 0.10 | 0.08 | 0.19 | 0.29 | 0.23 | 0.30 | 0.38 | 0.34 | 0.19 | 0.13 | 0.08 | 0.13 | 0.03 | 0.01 | 0.01 | -0.08 | -0.02 | -0.10 | -0.12 | -0.13 | -0.14 | -0.14 | -0.13 | -0.16 | -0.15 | -0.15 | -0.13 | -0.15 | -0.16 | -0.15 | -0.12 | -0.12 | -0.06 | -0.01 | -0.01 | -0.01 | -0.01 | 0.01 | 0.04 | 0.00 | -0.01 | -0.09 | -0.15 | -0.18 | -0.18 | -0.17 | -0.20 | -0.18 | -0.17 | -0.16 | -0.12 | -0.09 | -0.06 | -0.09 | -0.06 | -0.08 | -0.10 | -0.12 | -0.16 | -0.11 | -0.14 | -0.15 | -0.14 | -0.14 | -0.14 | -0.14 | -0.10 | -0.11 | -0.11 | -0.09 | -0.09 | -0.09 | -0.05 | -0.05 | -0.04 | 0.01 | -0.12 | -0.16 | -0.12 | -0.11 | -0.13 | -0.14 | -0.14 | -0.13 | -0.12 | -0.12 | -0.12 | -0.10 | -0.07 | -0.09 | -0.06 | -0.10 | -0.08 | -0.03 | -0.06 | -0.10 | -0.11 | -0.08 | -0.08 | -0.10 | -0.08 | -0.08 | -0.07 | -0.05 | -0.00 | 0.00 | -0.03 | -0.04 | -0.09 | -0.11 | -0.17 | -0.15 | -0.15 | -0.09 | -0.08 | -0.06 | -0.05 | -0.05 | -0.05 | -0.09 | -0.08 | -0.05 | -0.03 | -0.07 | -0.05 | -0.05 | -0.08 | -0.09 | -0.06 | -0.03 | -0.04 | 0.00 | -0.10 | -0.08 | -0.03 | -0.06 | -0.11 | -0.17 | -0.21 | -0.23 | -0.20 | -0.22 | -0.24 | -0.13 | -0.08 | -0.13 | -0.10 | -0.08 | -0.13 | -0.14 | -0.02 | 0.04 | -0.01 | -0.02 | 0.02 | 0.03 | 0.10 | 0.10 | 0.09 | 0.04 | 0.07 | 0.13 | 0.11 | 0.13 | 0.17 | 0.18 | 0.21 | 0.24 | 0.24 | 0.24 | 0.24 | 0.23 | 0.21 | 0.20 | 0.24 | 0.21 | 0.12 | 0.14 | 0.17 | 0.18 | 0.16 | 0.19 | 0.24 | 0.26 | 0.25 | 0.24 | 0.27 | 0.27 | 0.25 | 0.27 | 0.27 | 0.26 | 0.23 | 0.20 | 0.22 | 0.19 | 0.14 | 0.15 | 0.19 | 0.24 | 0.25 | 0.23 | 0.18 | 0.16 | 0.19 | 0.22 | 0.15 | 0.13 | 0.14 | 0.14 | -0.02 | -0.04 | 0.02 | -0.09 | -0.16 | -0.09 | -0.06 | -0.14 | -0.16 | -0.19 | -0.14 | -0.13 | -0.14 | -0.15 | -0.13 | -0.14 | -0.13 | -0.10 | -0.10 | -0.06 | -0.06 | -0.03 | -0.04 | -0.06 | -0.06 | -0.13 | -0.16 | -0.17 | -0.23 | -0.22 | -0.20 | -0.22 | -0.22 | -0.24 | -0.08 | -0.02 | 0.02 | -0.04 | -0.21 | -0.16 | -0.14 | -0.10 | -0.17 | -0.16 | -0.16 | 0.02 | -0.16 | -0.13 | -0.20 | -0.19 | -0.14 | -0.08 | -0.11 | -0.13 | -0.14 | -0.08 | -0.05 | -0.11 | -0.11 | -0.17 | -0.10 | -0.05 | -0.06 | -0.04 | -0.11 | -0.07 | -0.14 | -0.14 | -0.11 | -0.13 | -0.19 | -0.17 | -0.15 | -0.17 | -0.17 | -0.18 | -0.19 | -0.16 | -0.17 | -0.18 | -0.18 | -0.17 | -0.17 | -0.20 | -0.19 | -0.16 | -0.17 | -0.15 | -0.15 | -0.18 | -0.16 | -0.15 | -0.14 | -0.15 | -0.18 | -0.23 | -0.21 | -0.23 | -0.22 | -0.22 | -0.17 | -0.15 | -0.11 | -0.05 | -0.10 | -0.17 | -0.17 | -0.17 | -0.20 | -0.23 | -0.23 | -0.21 | -0.20 | -0.21 | -0.21 | -0.14 | -0.20 | -0.19 | -0.16 | -0.14 | -0.07 | -0.04 | -0.04 | -0.02 | 0.06 | 0.04 | -0.03 | -0.06 | 0.09 | 0.16 | 0.04 | -0.02 | 0.06 | 0.15 | 0.11 | 0.03 | 0.08 | 0.11 | 0.03 | -0.01 | -0.05 |
| A-Val-10 | 0.13 | 0.29 | 0.44 | 0.51 | 0.62 | 0.82 | 1.00 | 0.76 | 0.56 | 0.40 | 0.34 | 0.54 | 0.68 | 0.68 | 0.62 | 0.54 | 0.42 | 0.34 | 0.18 | 0.10 | -0.00 | -0.07 | -0.03 | 0.04 | 0.15 | 0.20 | 0.30 | 0.29 | 0.42 | 0.26 | 0.17 | 0.19 | 0.06 | 0.03 | -0.02 | -0.04 | -0.06 | -0.18 | -0.15 | -0.12 | -0.12 | -0.20 | -0.19 | -0.17 | -0.16 | -0.14 | -0.10 | -0.04 | -0.01 | 0.05 | 0.22 | 0.33 | 0.31 | 0.33 | 0.35 | 0.44 | 0.35 | 0.40 | 0.37 | 0.33 | 0.18 | 0.09 | 0.06 | 0.01 | -0.02 | -0.09 | -0.12 | -0.10 | -0.09 | -0.12 | -0.11 | -0.11 | -0.08 | -0.06 | -0.06 | -0.06 | -0.04 | -0.03 | -0.04 | -0.07 | -0.10 | -0.13 | -0.11 | -0.09 | -0.04 | -0.04 | -0.05 | -0.07 | -0.09 | -0.12 | -0.14 | -0.07 | -0.06 | -0.03 | 0.05 | 0.09 | 0.15 | 0.18 | 0.22 | 0.27 | 0.23 | 0.27 | 0.31 | 0.33 | 0.26 | 0.15 | 0.17 | 0.13 | 0.13 | 0.07 | 0.06 | -0.02 | -0.00 | -0.04 | -0.06 | -0.06 | -0.03 | -0.10 | -0.11 | -0.12 | -0.11 | -0.08 | -0.04 | -0.05 | -0.03 | -0.04 | -0.08 | -0.06 | -0.09 | -0.03 | -0.03 | -0.07 | 0.03 | -0.04 | -0.06 | 0.00 | -0.06 | -0.10 | -0.14 | -0.04 | 0.02 | 0.10 | 0.10 | 0.09 | 0.04 | -0.02 | -0.04 | -0.08 | -0.10 | -0.09 | -0.13 | -0.14 | -0.12 | -0.09 | -0.13 | -0.11 | -0.12 | -0.07 | -0.10 | -0.07 | -0.06 | -0.08 | -0.06 | -0.09 | -0.13 | -0.11 | -0.15 | -0.16 | -0.11 | -0.09 | -0.07 | -0.05 | -0.05 | -0.03 | -0.01 | 0.05 | 0.05 | 0.12 | 0.18 | 0.13 | 0.16 | 0.27 | 0.36 | 0.27 | 0.34 | 0.43 | 0.38 | 0.21 | 0.18 | 0.13 | 0.16 | 0.08 | 0.06 | 0.07 | -0.04 | -0.01 | -0.12 | -0.12 | -0.14 | -0.13 | -0.09 | -0.10 | -0.14 | -0.12 | -0.11 | -0.10 | -0.13 | -0.14 | -0.12 | -0.11 | -0.11 | -0.05 | -0.01 | -0.00 | 0.02 | 0.01 | 0.04 | 0.08 | 0.04 | 0.01 | -0.07 | -0.12 | -0.18 | -0.17 | -0.14 | -0.18 | -0.15 | -0.15 | -0.13 | -0.10 | -0.07 | -0.04 | -0.08 | -0.07 | -0.10 | -0.11 | -0.12 | -0.16 | -0.10 | -0.15 | -0.16 | -0.16 | -0.15 | -0.15 | -0.14 | -0.12 | -0.13 | -0.12 | -0.11 | -0.10 | -0.11 | -0.08 | -0.06 | -0.07 | -0.02 | -0.12 | -0.16 | -0.13 | -0.11 | -0.13 | -0.15 | -0.17 | -0.16 | -0.13 | -0.14 | -0.13 | -0.11 | -0.06 | -0.07 | -0.05 | -0.09 | -0.08 | -0.02 | -0.06 | -0.07 | -0.06 | -0.06 | -0.08 | -0.10 | -0.08 | -0.09 | -0.07 | -0.07 | -0.02 | 0.01 | -0.04 | -0.04 | -0.07 | -0.10 | -0.18 | -0.15 | -0.14 | -0.09 | -0.08 | -0.07 | -0.06 | -0.08 | -0.07 | -0.10 | -0.08 | -0.05 | -0.02 | -0.06 | -0.02 | -0.04 | -0.05 | -0.08 | -0.06 | -0.03 | -0.04 | 0.00 | -0.10 | -0.06 | -0.02 | -0.06 | -0.12 | -0.14 | -0.19 | -0.21 | -0.19 | -0.21 | -0.21 | -0.11 | -0.05 | -0.10 | -0.07 | -0.05 | -0.10 | -0.12 | -0.01 | 0.05 | 0.01 | 0.01 | 0.04 | 0.04 | 0.10 | 0.09 | 0.07 | 0.03 | 0.08 | 0.14 | 0.14 | 0.14 | 0.18 | 0.20 | 0.21 | 0.21 | 0.21 | 0.21 | 0.23 | 0.21 | 0.18 | 0.15 | 0.20 | 0.16 | 0.08 | 0.11 | 0.16 | 0.16 | 0.15 | 0.16 | 0.19 | 0.20 | 0.19 | 0.17 | 0.21 | 0.23 | 0.19 | 0.20 | 0.24 | 0.23 | 0.19 | 0.15 | 0.19 | 0.17 | 0.12 | 0.12 | 0.17 | 0.22 | 0.24 | 0.23 | 0.20 | 0.18 | 0.22 | 0.23 | 0.18 | 0.16 | 0.17 | 0.14 | 0.01 | -0.02 | 0.01 | -0.10 | -0.15 | -0.11 | -0.05 | -0.13 | -0.15 | -0.19 | -0.15 | -0.11 | -0.14 | -0.16 | -0.12 | -0.13 | -0.13 | -0.07 | -0.08 | -0.05 | -0.04 | -0.02 | -0.04 | -0.05 | -0.06 | -0.12 | -0.14 | -0.15 | -0.20 | -0.21 | -0.17 | -0.20 | -0.19 | -0.23 | -0.06 | -0.00 | 0.04 | -0.02 | -0.18 | -0.11 | -0.08 | -0.04 | -0.11 | -0.09 | -0.10 | 0.05 | -0.10 | -0.10 | -0.19 | -0.17 | -0.14 | -0.07 | -0.11 | -0.14 | -0.15 | -0.09 | -0.07 | -0.14 | -0.14 | -0.19 | -0.12 | -0.07 | -0.10 | -0.07 | -0.12 | -0.10 | -0.17 | -0.16 | -0.14 | -0.16 | -0.21 | -0.18 | -0.17 | -0.22 | -0.20 | -0.20 | -0.22 | -0.18 | -0.20 | -0.20 | -0.20 | -0.15 | -0.14 | -0.18 | -0.19 | -0.14 | -0.14 | -0.15 | -0.14 | -0.18 | -0.17 | -0.18 | -0.18 | -0.19 | -0.19 | -0.22 | -0.21 | -0.24 | -0.22 | -0.20 | -0.15 | -0.13 | -0.06 | 0.00 | -0.07 | -0.12 | -0.13 | -0.13 | -0.18 | -0.22 | -0.20 | -0.19 | -0.19 | -0.21 | -0.22 | -0.15 | -0.22 | -0.20 | -0.17 | -0.14 | -0.07 | -0.03 | -0.05 | -0.02 | 0.05 | 0.04 | -0.02 | -0.09 | 0.03 | 0.09 | -0.02 | -0.07 | 0.01 | 0.08 | 0.05 | -0.02 | 0.03 | 0.06 | -0.00 | -0.02 | -0.03 |
| A-Thr-11 | 0.05 | 0.18 | 0.32 | 0.37 | 0.46 | 0.61 | 0.76 | 1.00 | 0.80 | 0.65 | 0.58 | 0.72 | 0.75 | 0.63 | 0.55 | 0.44 | 0.34 | 0.24 | 0.08 | -0.00 | -0.07 | -0.13 | -0.09 | -0.01 | 0.05 | 0.09 | 0.22 | 0.21 | 0.33 | 0.18 | 0.11 | 0.11 | -0.02 | 0.04 | 0.08 | 0.05 | -0.03 | -0.12 | -0.10 | -0.06 | -0.10 | -0.18 | -0.17 | -0.14 | -0.10 | -0.08 | -0.04 | -0.00 | 0.06 | 0.19 | 0.36 | 0.48 | 0.45 | 0.42 | 0.49 | 0.53 | 0.38 | 0.39 | 0.33 | 0.26 | 0.11 | -0.01 | -0.05 | -0.08 | -0.09 | -0.13 | -0.16 | -0.14 | -0.11 | -0.13 | -0.13 | -0.11 | -0.08 | -0.09 | -0.09 | -0.08 | -0.09 | -0.08 | -0.09 | -0.11 | -0.15 | -0.18 | -0.15 | -0.13 | -0.10 | -0.04 | -0.08 | -0.10 | -0.14 | -0.17 | -0.14 | -0.09 | -0.06 | -0.03 | 0.01 | 0.08 | 0.11 | 0.14 | 0.15 | 0.20 | 0.15 | 0.17 | 0.21 | 0.23 | 0.19 | 0.11 | 0.11 | 0.11 | 0.10 | 0.03 | 0.04 | -0.03 | -0.02 | -0.06 | -0.06 | -0.06 | -0.02 | -0.11 | -0.12 | -0.11 | -0.12 | -0.10 | -0.08 | -0.09 | -0.06 | -0.07 | -0.12 | -0.12 | -0.18 | -0.09 | -0.07 | -0.10 | 0.01 | -0.07 | -0.06 | -0.02 | -0.08 | -0.13 | -0.14 | -0.04 | -0.01 | 0.07 | 0.07 | 0.07 | 0.03 | -0.01 | -0.05 | -0.09 | -0.11 | -0.10 | -0.15 | -0.16 | -0.15 | -0.11 | -0.14 | -0.12 | -0.09 | -0.03 | -0.02 | -0.05 | -0.03 | -0.02 | -0.02 | -0.04 | -0.08 | -0.07 | -0.08 | -0.11 | -0.10 | -0.07 | -0.06 | -0.04 | -0.03 | -0.00 | 0.02 | 0.07 | 0.08 | 0.13 | 0.20 | 0.15 | 0.17 | 0.30 | 0.40 | 0.25 | 0.31 | 0.38 | 0.34 | 0.18 | 0.15 | 0.11 | 0.15 | 0.09 | 0.04 | 0.06 | -0.04 | -0.02 | -0.09 | -0.10 | -0.11 | -0.09 | -0.08 | -0.09 | -0.13 | -0.12 | -0.10 | -0.08 | -0.12 | -0.11 | -0.08 | -0.06 | -0.07 | -0.04 | -0.03 | -0.03 | -0.02 | 0.02 | 0.03 | 0.03 | 0.01 | 0.01 | -0.06 | -0.11 | -0.16 | -0.14 | -0.11 | -0.14 | -0.12 | -0.11 | -0.10 | -0.08 | -0.08 | -0.06 | -0.09 | -0.07 | -0.08 | -0.09 | -0.09 | -0.11 | -0.08 | -0.12 | -0.13 | -0.14 | -0.13 | -0.14 | -0.14 | -0.12 | -0.14 | -0.14 | -0.14 | -0.14 | -0.14 | -0.12 | -0.10 | -0.13 | -0.09 | -0.05 | -0.07 | -0.07 | -0.10 | -0.11 | -0.15 | -0.20 | -0.20 | -0.16 | -0.15 | -0.12 | -0.16 | -0.14 | -0.12 | -0.11 | -0.13 | -0.09 | -0.06 | -0.11 | -0.12 | -0.09 | -0.06 | -0.10 | -0.12 | -0.05 | -0.03 | -0.02 | -0.04 | -0.03 | -0.03 | -0.06 | -0.07 | -0.11 | -0.13 | -0.18 | -0.15 | -0.12 | -0.07 | -0.05 | -0.03 | -0.02 | -0.05 | -0.06 | -0.06 | -0.04 | -0.01 | -0.01 | -0.06 | -0.06 | -0.05 | -0.04 | -0.07 | -0.05 | -0.04 | -0.04 | -0.03 | -0.09 | -0.04 | -0.01 | -0.05 | -0.13 | -0.13 | -0.16 | -0.16 | -0.15 | -0.16 | -0.16 | -0.06 | -0.00 | -0.08 | -0.04 | -0.05 | -0.08 | -0.12 | -0.06 | 0.00 | -0.02 | -0.04 | 0.02 | 0.06 | 0.11 | 0.10 | 0.06 | 0.04 | 0.08 | 0.14 | 0.15 | 0.17 | 0.18 | 0.18 | 0.21 | 0.19 | 0.16 | 0.16 | 0.19 | 0.16 | 0.11 | 0.10 | 0.17 | 0.14 | 0.05 | 0.09 | 0.13 | 0.11 | 0.13 | 0.15 | 0.18 | 0.18 | 0.17 | 0.16 | 0.21 | 0.21 | 0.18 | 0.19 | 0.23 | 0.22 | 0.19 | 0.18 | 0.24 | 0.21 | 0.13 | 0.12 | 0.17 | 0.21 | 0.21 | 0.24 | 0.20 | 0.20 | 0.23 | 0.22 | 0.17 | 0.16 | 0.17 | 0.16 | 0.06 | 0.04 | 0.04 | -0.05 | -0.09 | -0.05 | -0.02 | -0.07 | -0.08 | -0.10 | -0.10 | -0.07 | -0.08 | -0.09 | -0.08 | -0.08 | -0.08 | -0.02 | -0.05 | -0.03 | 0.01 | 0.02 | -0.05 | -0.04 | -0.05 | -0.14 | -0.16 | -0.16 | -0.18 | -0.17 | -0.14 | -0.14 | -0.10 | -0.12 | -0.00 | 0.09 | 0.11 | 0.03 | -0.07 | -0.01 | -0.01 | 0.06 | 0.01 | -0.02 | -0.04 | 0.13 | 0.02 | -0.06 | -0.18 | -0.12 | -0.16 | -0.13 | -0.16 | -0.17 | -0.22 | -0.14 | -0.11 | -0.15 | -0.12 | -0.24 | -0.15 | -0.09 | -0.10 | -0.06 | -0.13 | -0.15 | -0.20 | -0.18 | -0.16 | -0.15 | -0.21 | -0.21 | -0.17 | -0.20 | -0.22 | -0.23 | -0.21 | -0.18 | -0.18 | -0.19 | -0.20 | -0.16 | -0.15 | -0.18 | -0.17 | -0.12 | -0.15 | -0.16 | -0.15 | -0.19 | -0.16 | -0.18 | -0.18 | -0.16 | -0.13 | -0.18 | -0.16 | -0.23 | -0.23 | -0.22 | -0.19 | -0.17 | -0.12 | -0.05 | -0.06 | -0.11 | -0.14 | -0.15 | -0.17 | -0.19 | -0.16 | -0.13 | -0.15 | -0.16 | -0.19 | -0.12 | -0.19 | -0.17 | -0.16 | -0.14 | -0.11 | -0.06 | -0.09 | -0.05 | -0.01 | -0.02 | -0.04 | -0.08 | 0.01 | 0.06 | -0.03 | -0.10 | -0.02 | 0.05 | 0.03 | -0.04 | 0.00 | 0.04 | -0.01 | -0.02 | -0.03 |
| A-Val-12 | -0.01 | 0.09 | 0.21 | 0.23 | 0.32 | 0.44 | 0.56 | 0.80 | 1.00 | 0.86 | 0.71 | 0.74 | 0.65 | 0.55 | 0.43 | 0.32 | 0.23 | 0.15 | 0.02 | -0.05 | -0.10 | -0.13 | -0.10 | -0.05 | -0.01 | 0.02 | 0.13 | 0.15 | 0.26 | 0.17 | 0.14 | 0.14 | 0.01 | 0.07 | 0.12 | 0.10 | -0.01 | -0.09 | -0.09 | -0.04 | -0.08 | -0.13 | -0.13 | -0.12 | -0.08 | -0.05 | -0.02 | 0.02 | 0.10 | 0.26 | 0.45 | 0.57 | 0.46 | 0.37 | 0.36 | 0.39 | 0.34 | 0.31 | 0.31 | 0.22 | 0.15 | 0.03 | -0.01 | -0.04 | -0.04 | -0.06 | -0.10 | -0.09 | -0.08 | -0.10 | -0.10 | -0.07 | -0.06 | -0.05 | -0.03 | -0.03 | -0.06 | -0.10 | -0.07 | -0.08 | -0.13 | -0.16 | -0.10 | -0.09 | -0.04 | -0.02 | -0.04 | -0.05 | -0.07 | -0.09 | -0.08 | -0.03 | -0.01 | 0.03 | 0.05 | 0.11 | 0.12 | 0.12 | 0.08 | 0.14 | 0.09 | 0.08 | 0.09 | 0.14 | 0.14 | 0.10 | 0.12 | 0.11 | 0.11 | 0.08 | 0.10 | 0.02 | 0.04 | -0.00 | -0.03 | -0.03 | -0.04 | -0.07 | -0.08 | -0.09 | -0.09 | -0.06 | -0.05 | -0.05 | -0.03 | -0.04 | -0.09 | -0.11 | -0.15 | -0.09 | -0.09 | -0.08 | -0.03 | -0.11 | -0.10 | -0.05 | -0.04 | -0.10 | -0.10 | -0.02 | 0.02 | 0.06 | 0.04 | 0.10 | 0.07 | 0.04 | -0.02 | -0.07 | -0.08 | -0.08 | -0.12 | -0.14 | -0.13 | -0.08 | -0.10 | -0.08 | -0.07 | -0.02 | 0.00 | -0.04 | -0.04 | -0.03 | -0.03 | -0.06 | -0.09 | -0.07 | -0.06 | -0.11 | -0.09 | -0.06 | -0.03 | -0.02 | -0.02 | 0.02 | 0.06 | 0.10 | 0.13 | 0.19 | 0.26 | 0.20 | 0.24 | 0.38 | 0.45 | 0.28 | 0.29 | 0.28 | 0.26 | 0.12 | 0.14 | 0.11 | 0.15 | 0.12 | 0.10 | 0.12 | 0.03 | 0.06 | -0.02 | -0.05 | -0.08 | -0.11 | -0.11 | -0.09 | -0.11 | -0.11 | -0.10 | -0.06 | -0.09 | -0.10 | -0.06 | -0.06 | -0.06 | -0.03 | -0.00 | -0.01 | 0.01 | 0.04 | 0.06 | 0.08 | 0.06 | 0.08 | 0.02 | -0.03 | -0.09 | -0.08 | -0.08 | -0.13 | -0.10 | -0.10 | -0.09 | -0.07 | -0.06 | -0.05 | -0.07 | -0.06 | -0.05 | -0.06 | -0.08 | -0.10 | -0.07 | -0.10 | -0.10 | -0.11 | -0.10 | -0.11 | -0.11 | -0.10 | -0.12 | -0.13 | -0.12 | -0.11 | -0.13 | -0.11 | -0.09 | -0.11 | -0.06 | -0.04 | -0.07 | -0.06 | -0.09 | -0.08 | -0.14 | -0.19 | -0.17 | -0.16 | -0.16 | -0.10 | -0.14 | -0.12 | -0.12 | -0.13 | -0.13 | -0.11 | -0.08 | -0.11 | -0.11 | -0.07 | -0.04 | -0.09 | -0.10 | -0.04 | -0.02 | -0.02 | -0.02 | -0.02 | -0.02 | -0.04 | -0.06 | -0.09 | -0.10 | -0.15 | -0.11 | -0.10 | -0.07 | -0.07 | -0.03 | -0.02 | -0.04 | -0.05 | -0.08 | -0.06 | -0.03 | -0.04 | -0.08 | -0.07 | -0.06 | -0.06 | -0.06 | -0.04 | -0.04 | -0.03 | -0.01 | -0.05 | -0.00 | 0.01 | -0.02 | -0.09 | -0.09 | -0.11 | -0.11 | -0.11 | -0.14 | -0.12 | -0.03 | 0.03 | -0.04 | -0.02 | -0.00 | -0.04 | -0.07 | -0.00 | 0.02 | -0.01 | 0.00 | 0.05 | 0.08 | 0.10 | 0.10 | 0.06 | 0.04 | 0.08 | 0.12 | 0.16 | 0.16 | 0.17 | 0.14 | 0.17 | 0.15 | 0.11 | 0.11 | 0.13 | 0.11 | 0.07 | 0.06 | 0.11 | 0.10 | 0.05 | 0.08 | 0.11 | 0.07 | 0.10 | 0.10 | 0.11 | 0.13 | 0.11 | 0.09 | 0.14 | 0.14 | 0.11 | 0.13 | 0.18 | 0.18 | 0.15 | 0.18 | 0.21 | 0.20 | 0.11 | 0.12 | 0.17 | 0.18 | 0.18 | 0.22 | 0.20 | 0.22 | 0.23 | 0.20 | 0.16 | 0.17 | 0.18 | 0.14 | 0.05 | 0.04 | 0.03 | -0.04 | -0.07 | -0.06 | -0.03 | -0.06 | -0.06 | -0.09 | -0.09 | -0.05 | -0.06 | -0.05 | -0.04 | -0.05 | -0.06 | 0.02 | -0.01 | -0.02 | 0.03 | 0.03 | -0.04 | -0.01 | -0.03 | -0.13 | -0.12 | -0.13 | -0.16 | -0.14 | -0.12 | -0.13 | -0.08 | -0.09 | 0.01 | 0.10 | 0.11 | 0.04 | -0.04 | 0.00 | 0.03 | 0.09 | 0.05 | 0.04 | 0.02 | 0.14 | 0.06 | -0.01 | -0.15 | -0.10 | -0.13 | -0.08 | -0.12 | -0.12 | -0.18 | -0.14 | -0.11 | -0.16 | -0.12 | -0.24 | -0.19 | -0.13 | -0.13 | -0.10 | -0.16 | -0.20 | -0.24 | -0.22 | -0.19 | -0.19 | -0.23 | -0.23 | -0.19 | -0.21 | -0.22 | -0.19 | -0.19 | -0.16 | -0.17 | -0.15 | -0.16 | -0.13 | -0.13 | -0.12 | -0.11 | -0.07 | -0.10 | -0.11 | -0.12 | -0.15 | -0.14 | -0.15 | -0.16 | -0.15 | -0.12 | -0.10 | -0.06 | -0.15 | -0.16 | -0.17 | -0.14 | -0.17 | -0.16 | -0.14 | -0.06 | -0.11 | -0.16 | -0.17 | -0.15 | -0.16 | -0.16 | -0.12 | -0.14 | -0.17 | -0.17 | -0.12 | -0.15 | -0.13 | -0.13 | -0.13 | -0.11 | -0.09 | -0.11 | -0.07 | -0.08 | -0.08 | -0.03 | -0.07 | -0.06 | -0.01 | -0.08 | -0.14 | -0.07 | -0.02 | -0.04 | -0.10 | -0.06 | -0.02 | -0.05 | -0.06 | -0.03 |
**Table-2**: Dynamic cross-correlation matrix. The full table is also available in text format, you need a proper text editor without line wrapping to look at this file: Complex two\_dccm.tab. Note: At most 10 rows of the DCCM are shown above. Change the **tabrowsmax** variable in the macro to adjust this number.

## 7. Additional files

The following additional files have been created:

### 7.1. The main data table

The main table contains all collected data in a single file. The column names match the names used above for graphs in plots and columns in tables. You can find a more detailed explanation of this table in the user manual at Recipes > Run a molecular dynamics simulation > Analyzing a trajectory. If you parse this file automatically, keep in mind that the number of columns can change any time, so you have to use the names in the first table row to find the columns of interest: Complex two\_analysis.tab

### 7.2. Per-atom and per-residue data tables

Data of the per-atom and per-residue plots:

Complex two\_plotres\_secstrMolA.tab

Complex two\_plotres\_secstrMolA.tab

Complex two\_plotres\_conMolA.tab

Complex two\_plotres\_conMolA.tab

### 7.3. The structures

The **time averaged structure** in PDB format: Complex two\_average.pdb

The **snapshot with the minimum solute energy**. Either just the solute in PDB format Complex two\_energymin.pdb, or the complete system including solvent as a YASARA scene Complex two\_energymin.sce.

The **last snapshot** of the simulation. Either just the solute in PDB format Complex two\_last.pdb, or the complete system including solvent as a YASARA scene Complex two\_last.sce

### 7.4. The RMSF tables

A table that lists the Root Mean Square Fluctuations [RMSFs] of all atoms in [A] is available here: Complex two\_rmsf.tab. The RMSFs have also been converted to B-factors and stored in the B-factor field of the time-average structure above.

A table with average atom RMSFs per residue can be found here: Complex two\_rmsfres.tab.

### 7.5. High resolution plots

To facilitate publication, high resolution versions of the plots above have been created with a 4:3 aspect ratio suited for printing in a single column of a typical journal article. Just look at the figure number above to find the right file:

Complex two\_report\_figure3\_hires.png

Complex two\_report\_figure4\_hires.png

Complex two\_report\_figure5\_hires.png

Complex two\_report\_figure6\_hires.png

Complex two\_report\_figure7\_hires.png

Complex two\_report\_figure8\_hires.png

Complex two\_report\_figure9\_hires.png

Complex two\_report\_figure10\_hires.png

Complex two\_report\_figure11\_hires.png

Complex two\_report\_figure12\_hires.png

Complex two\_report\_figure13\_hires.png

Complex two\_report\_figure14\_hires.png

Complex two\_report\_figure15\_hires.png

Complex two\_report\_figure16\_hires.png

Complex two\_report\_figure17\_hires.png

Complex two\_report\_figure18\_hires.png
