# Supplementary figures and images for: An evaluation of Roluperidone as a promising repurposing candidate for Alzheimer’s Disease: A Computational Investigation (part 2 of 2)
Source: PLoS One. 2025 Dec 17;20(12):e0338211. doi: 10.1371/journal.pone.0338211 (PMC12711050; doi:10.1371/journal.pone.0338211)

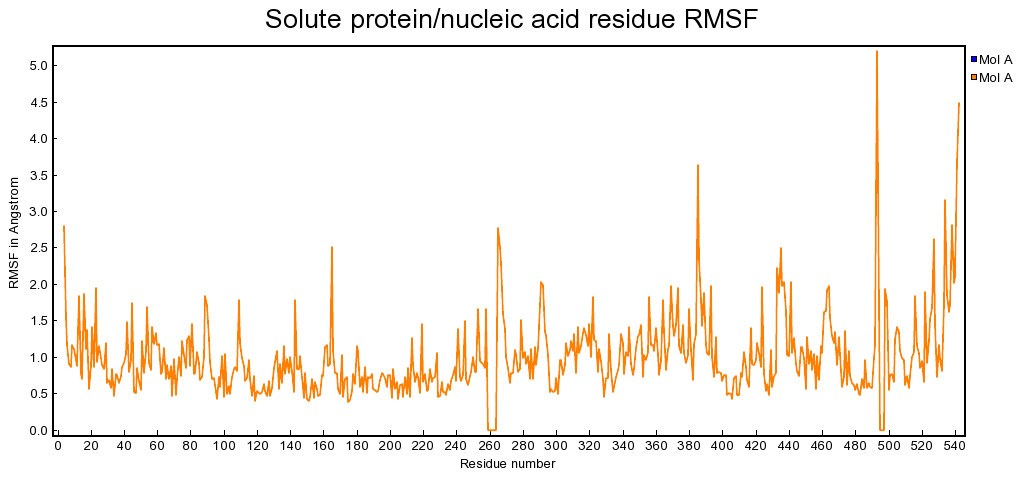

Supplement: S7 File — (ZIP) [file pone.0338211.s007.zip › S6.Molecular Dynamic Simulation/S6.Molecular Dynamic Simulation/Result napitane+roluperidone/Result 2/Complex two/Complex two_report_figure16.png]

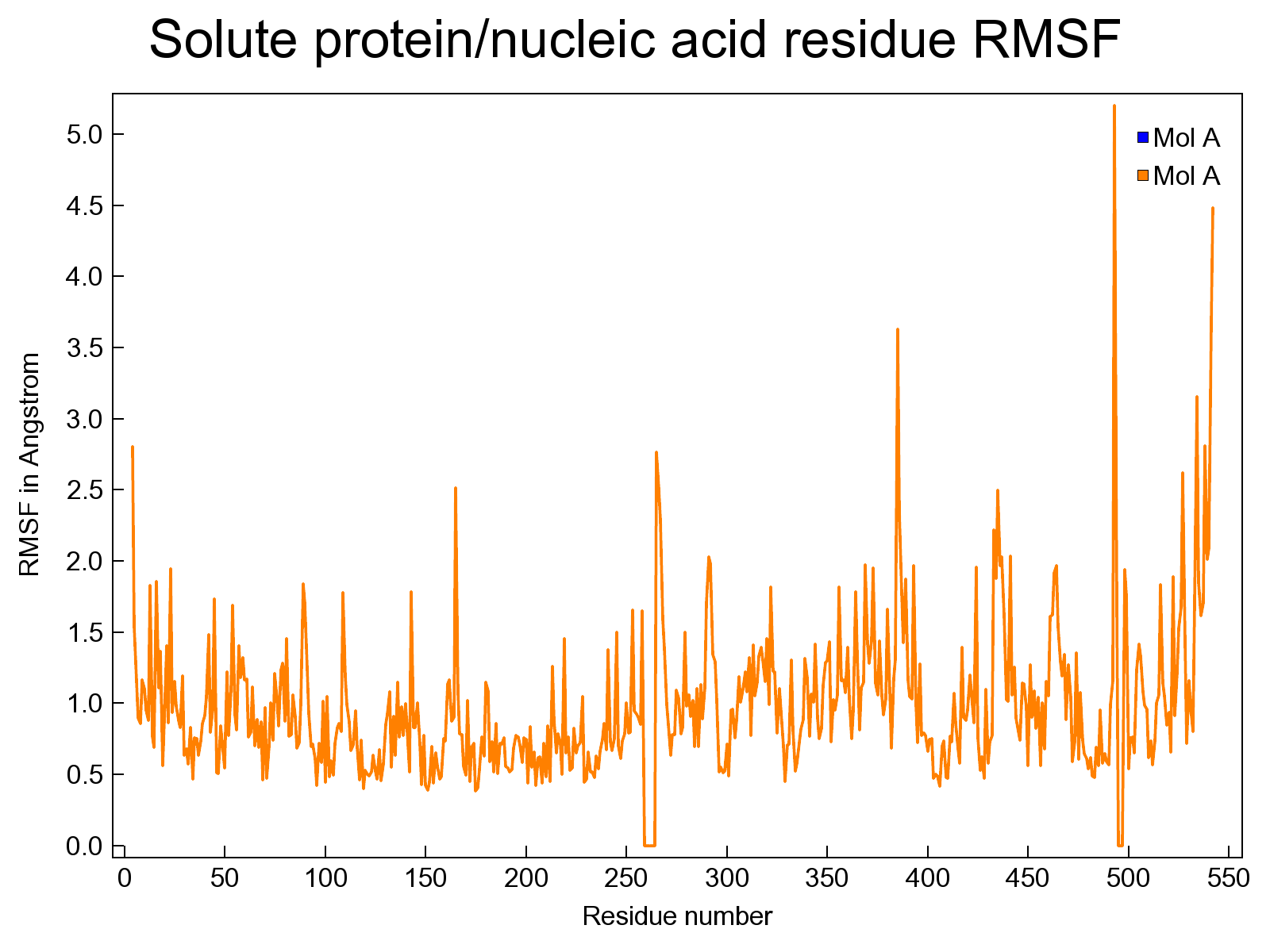

Supplement: S7 File — (ZIP) [file pone.0338211.s007.zip › S6.Molecular Dynamic Simulation/S6.Molecular Dynamic Simulation/Result napitane+roluperidone/Result 2/Complex two/Complex two_report_figure16_hires.png]

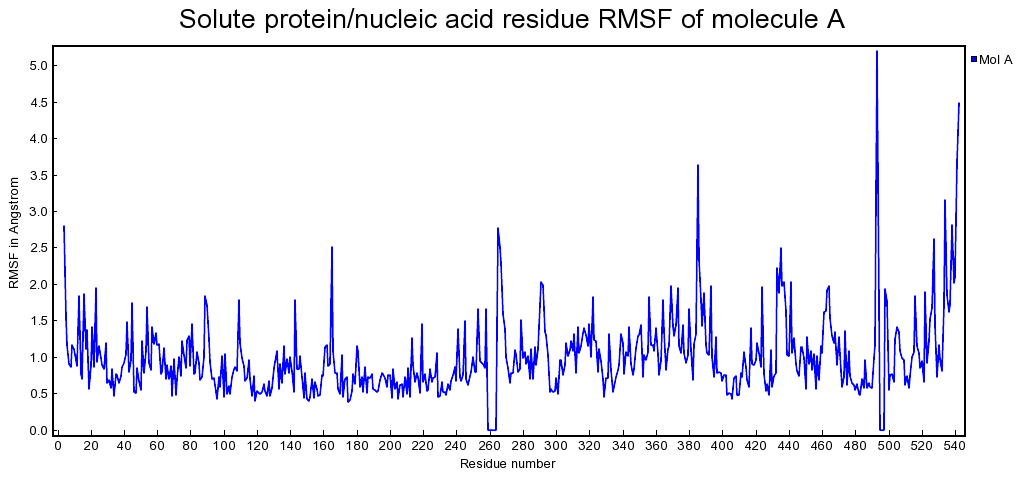

Supplement: S7 File — (ZIP) [file pone.0338211.s007.zip › S6.Molecular Dynamic Simulation/S6.Molecular Dynamic Simulation/Result napitane+roluperidone/Result 2/Complex two/Complex two_report_figure17.png]

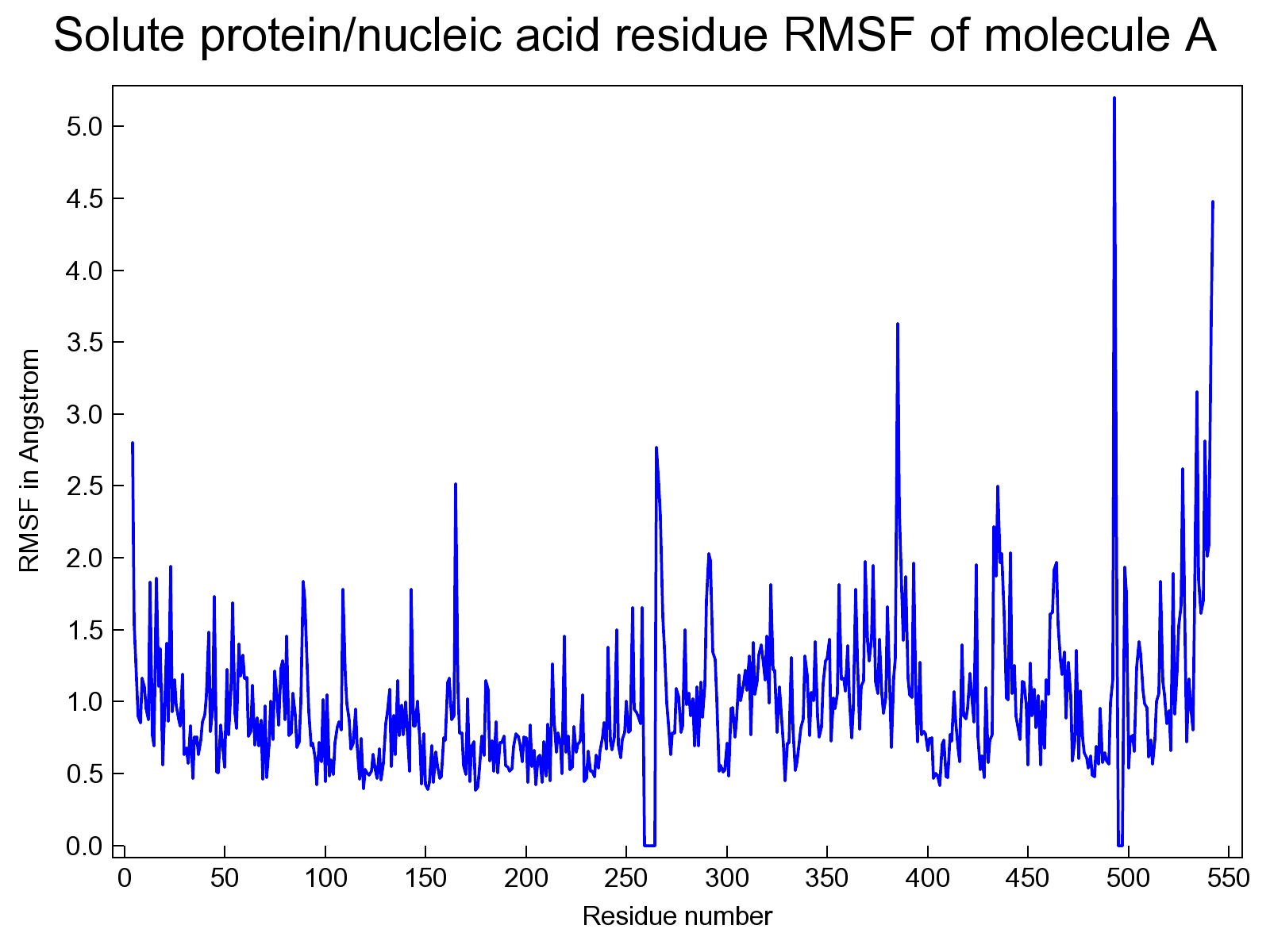

Supplement: S7 File — (ZIP) [file pone.0338211.s007.zip › S6.Molecular Dynamic Simulation/S6.Molecular Dynamic Simulation/Result napitane+roluperidone/Result 2/Complex two/Complex two_report_figure17_hires.png]

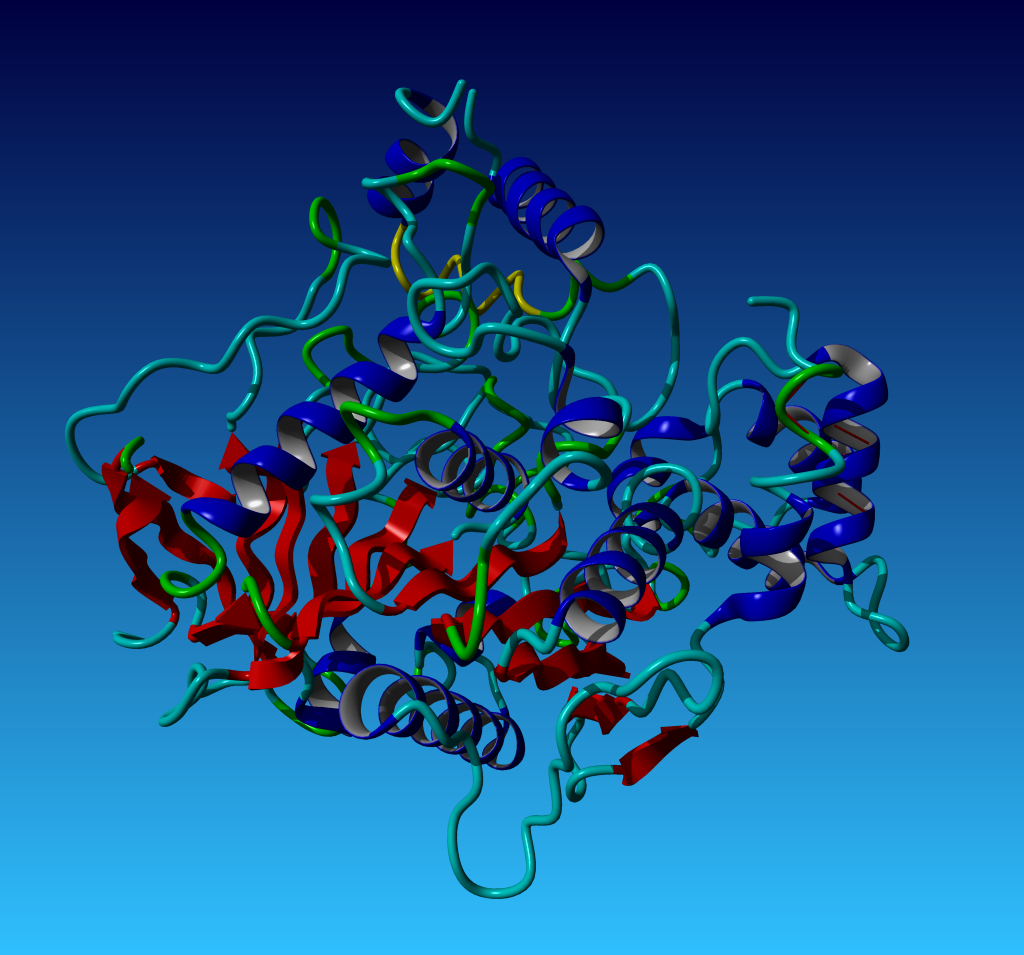

Supplement: S7 File — (ZIP) [file pone.0338211.s007.zip › S6.Molecular Dynamic Simulation/S6.Molecular Dynamic Simulation/Result napitane+roluperidone/Result 2/Complex two/Complex two_report_figure19.png]

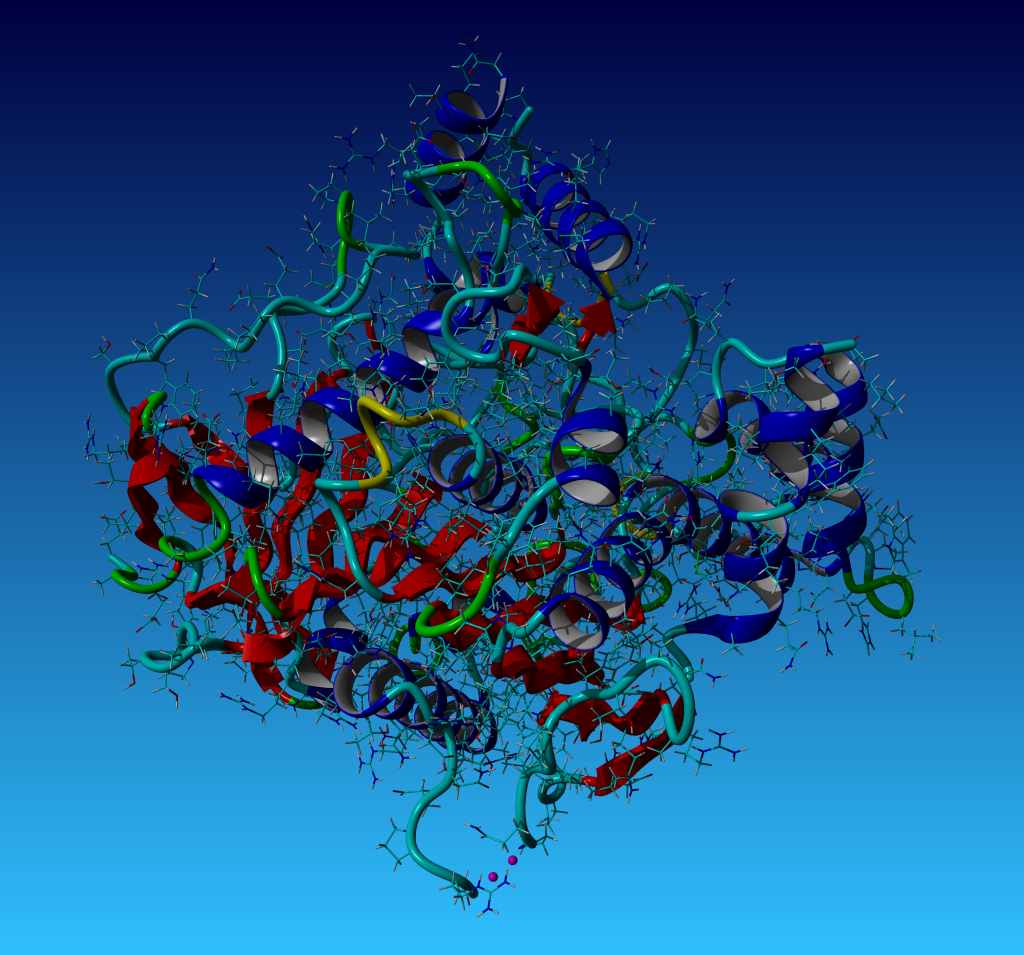

Supplement: S7 File — (ZIP) [file pone.0338211.s007.zip › S6.Molecular Dynamic Simulation/S6.Molecular Dynamic Simulation/Result napitane+roluperidone/Result 2/Complex two/Complex two_report_figure2.png]

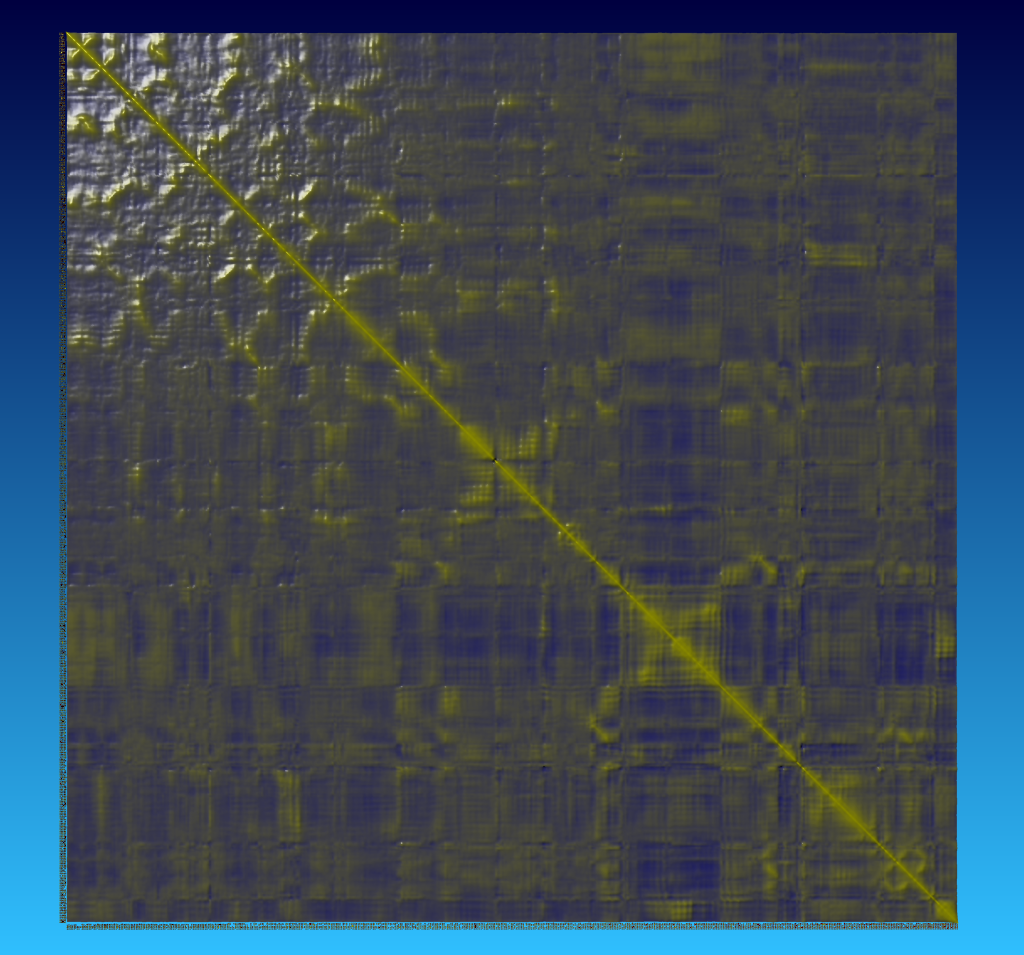

Supplement: S7 File — (ZIP) [file pone.0338211.s007.zip › S6.Molecular Dynamic Simulation/S6.Molecular Dynamic Simulation/Result napitane+roluperidone/Result 2/Complex two/Complex two_report_figure20.png]

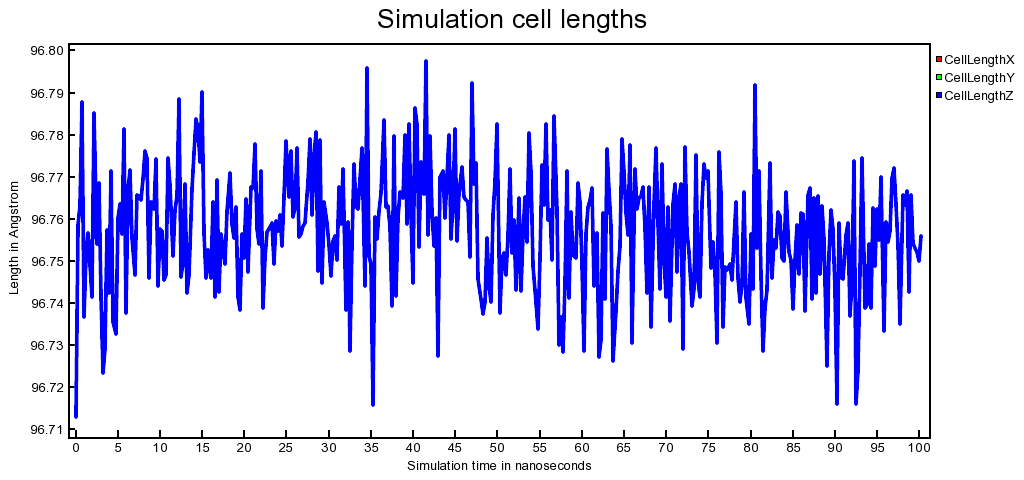

Supplement: S7 File — (ZIP) [file pone.0338211.s007.zip › S6.Molecular Dynamic Simulation/S6.Molecular Dynamic Simulation/Result napitane+roluperidone/Result 2/Complex two/Complex two_report_figure3.png]

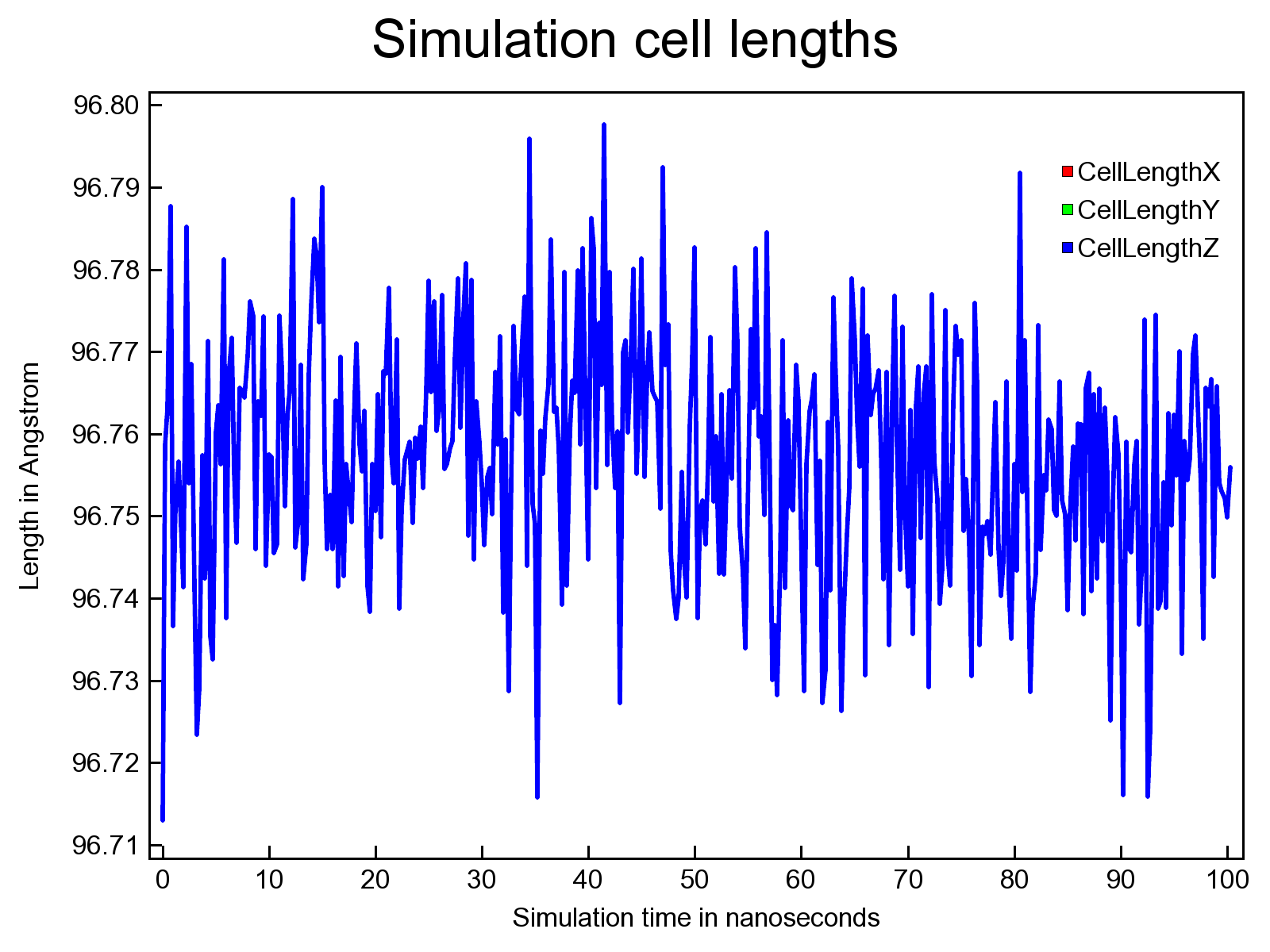

Supplement: S7 File — (ZIP) [file pone.0338211.s007.zip › S6.Molecular Dynamic Simulation/S6.Molecular Dynamic Simulation/Result napitane+roluperidone/Result 2/Complex two/Complex two_report_figure3_hires.png]

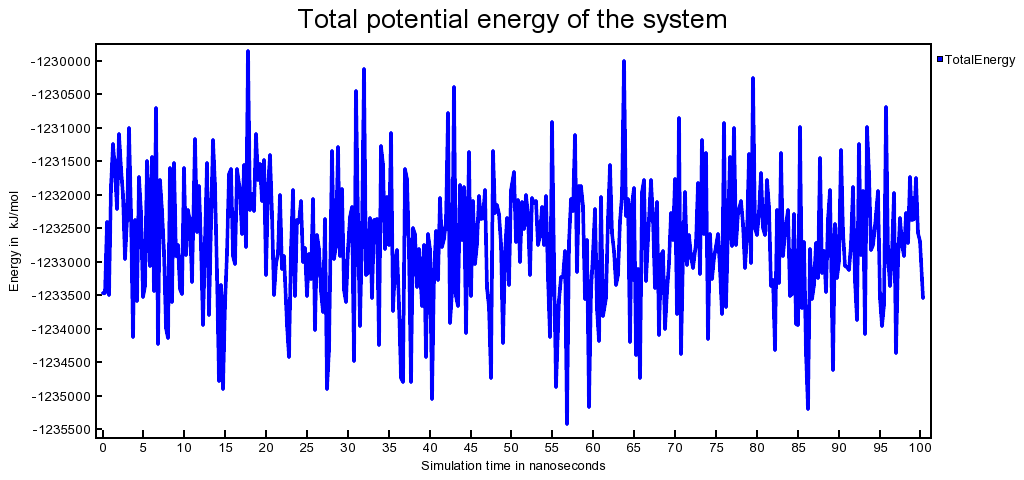

Supplement: S7 File — (ZIP) [file pone.0338211.s007.zip › S6.Molecular Dynamic Simulation/S6.Molecular Dynamic Simulation/Result napitane+roluperidone/Result 2/Complex two/Complex two_report_figure4.png]

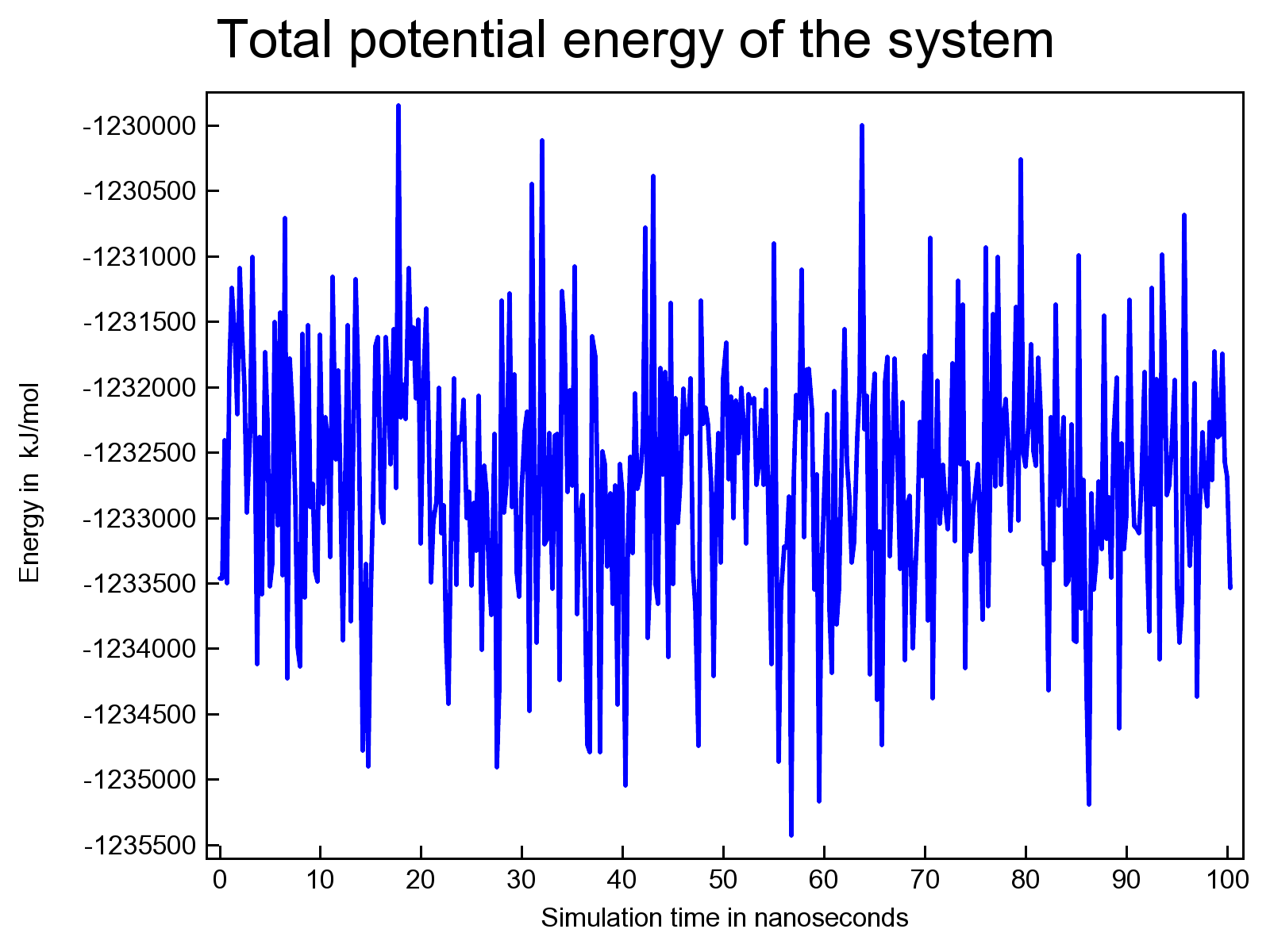

Supplement: S7 File — (ZIP) [file pone.0338211.s007.zip › S6.Molecular Dynamic Simulation/S6.Molecular Dynamic Simulation/Result napitane+roluperidone/Result 2/Complex two/Complex two_report_figure4_hires.png]

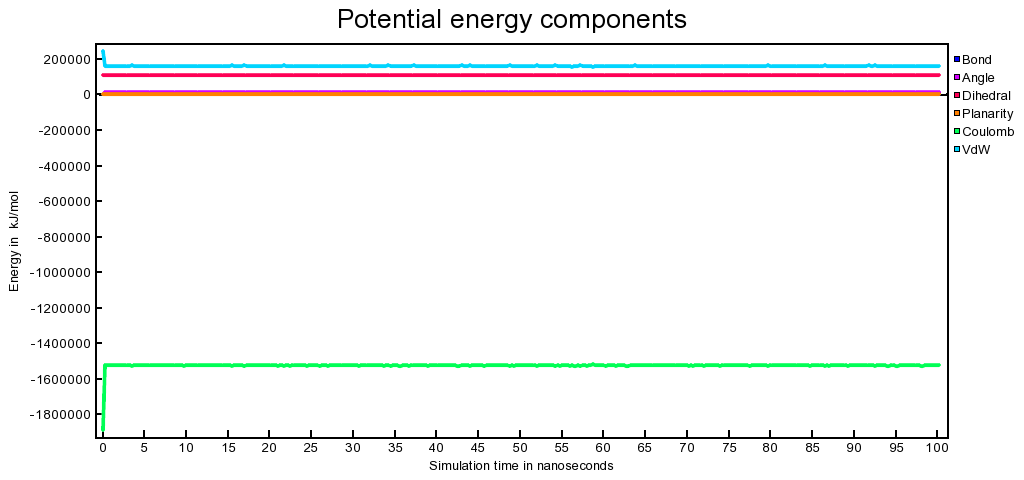

Supplement: S7 File — (ZIP) [file pone.0338211.s007.zip › S6.Molecular Dynamic Simulation/S6.Molecular Dynamic Simulation/Result napitane+roluperidone/Result 2/Complex two/Complex two_report_figure5.png]

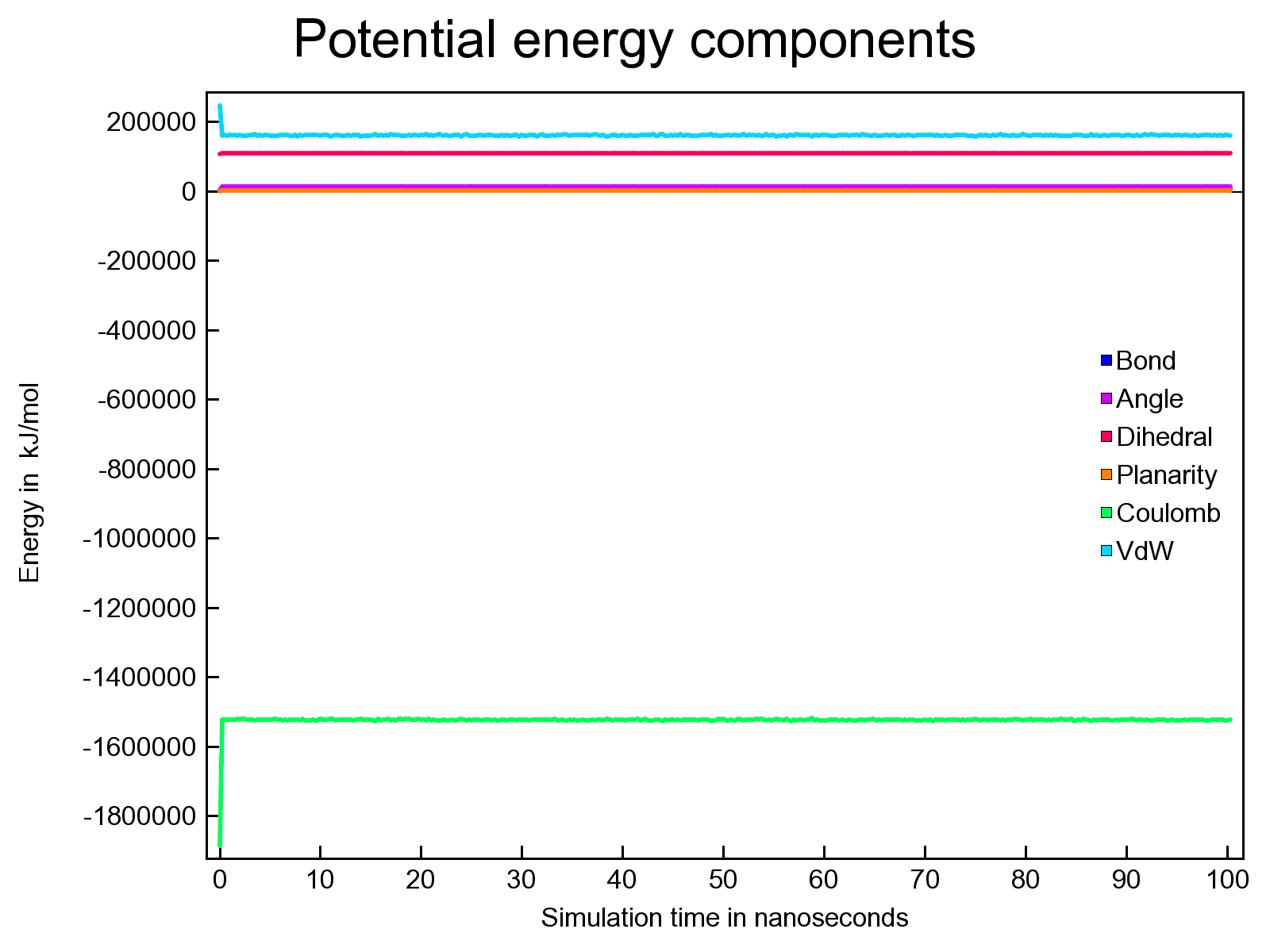

Supplement: S7 File — (ZIP) [file pone.0338211.s007.zip › S6.Molecular Dynamic Simulation/S6.Molecular Dynamic Simulation/Result napitane+roluperidone/Result 2/Complex two/Complex two_report_figure5_hires.png]

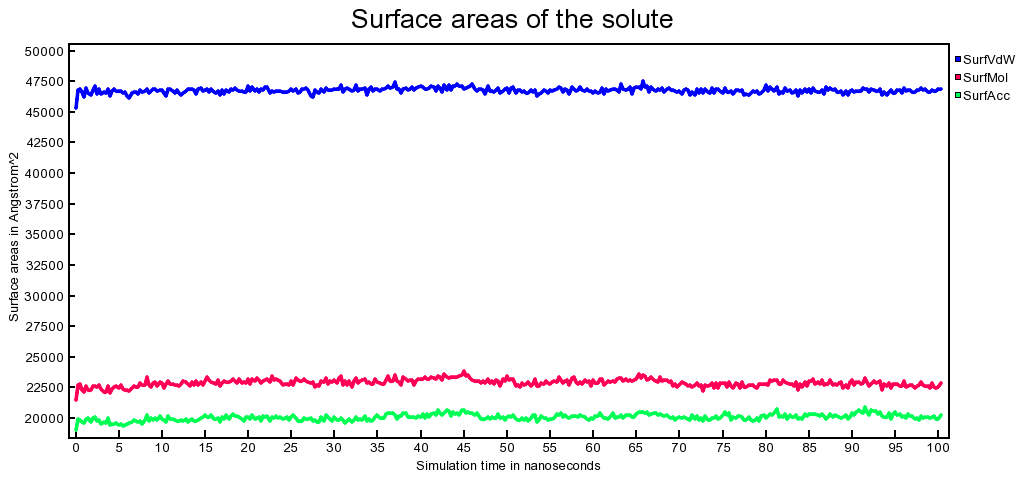

Supplement: S7 File — (ZIP) [file pone.0338211.s007.zip › S6.Molecular Dynamic Simulation/S6.Molecular Dynamic Simulation/Result napitane+roluperidone/Result 2/Complex two/Complex two_report_figure6.png]

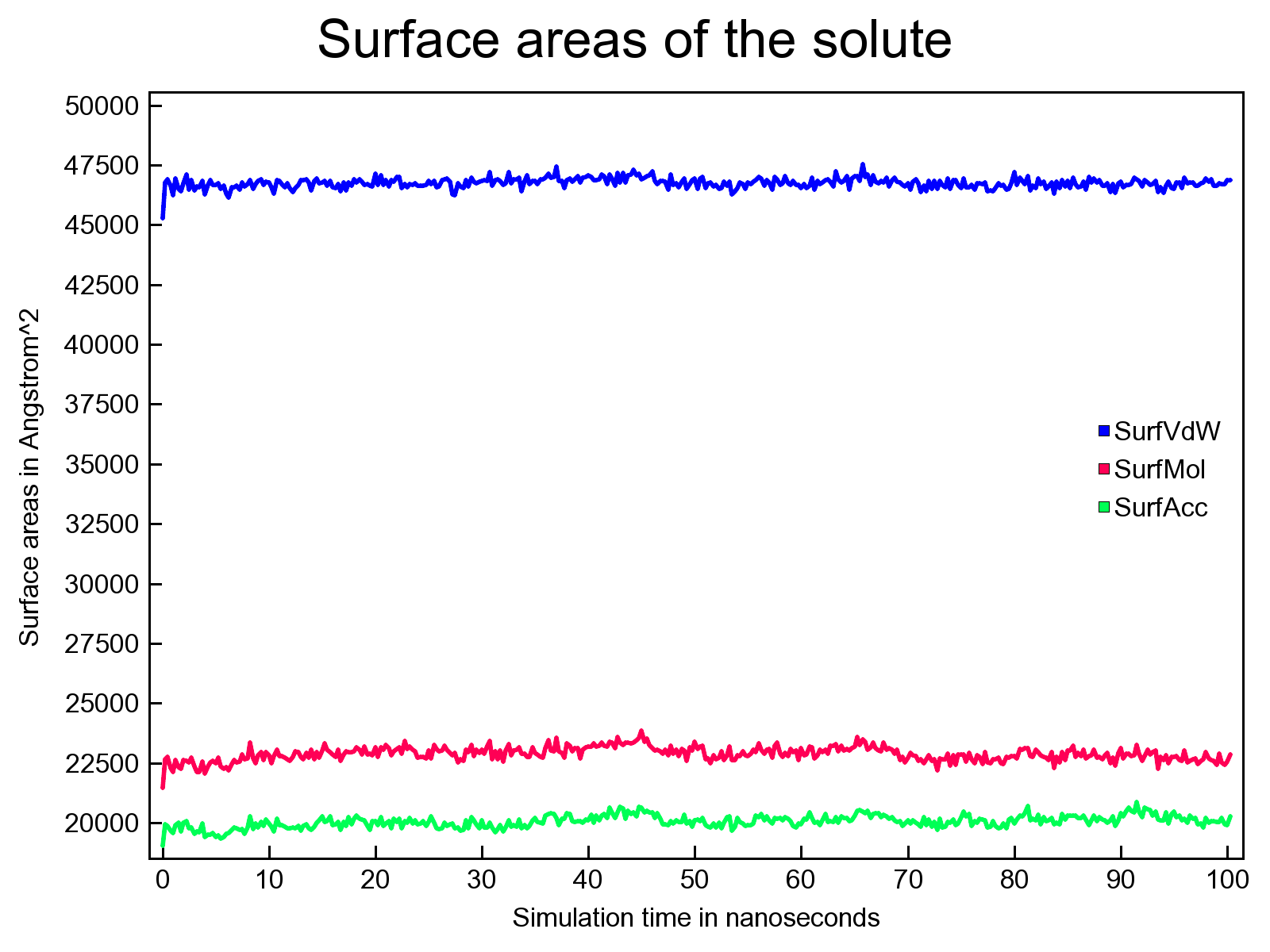

Supplement: S7 File — (ZIP) [file pone.0338211.s007.zip › S6.Molecular Dynamic Simulation/S6.Molecular Dynamic Simulation/Result napitane+roluperidone/Result 2/Complex two/Complex two_report_figure6_hires.png]

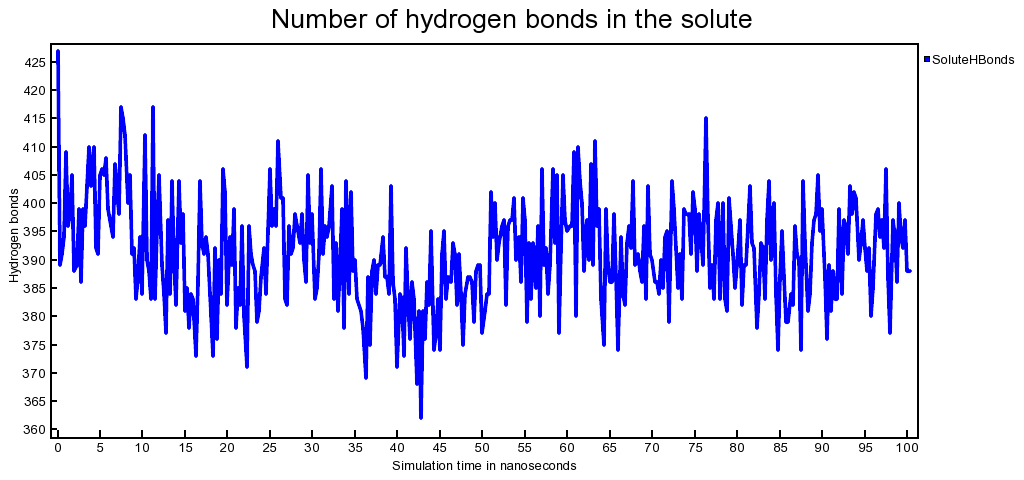

Supplement: S7 File — (ZIP) [file pone.0338211.s007.zip › S6.Molecular Dynamic Simulation/S6.Molecular Dynamic Simulation/Result napitane+roluperidone/Result 2/Complex two/Complex two_report_figure7.png]

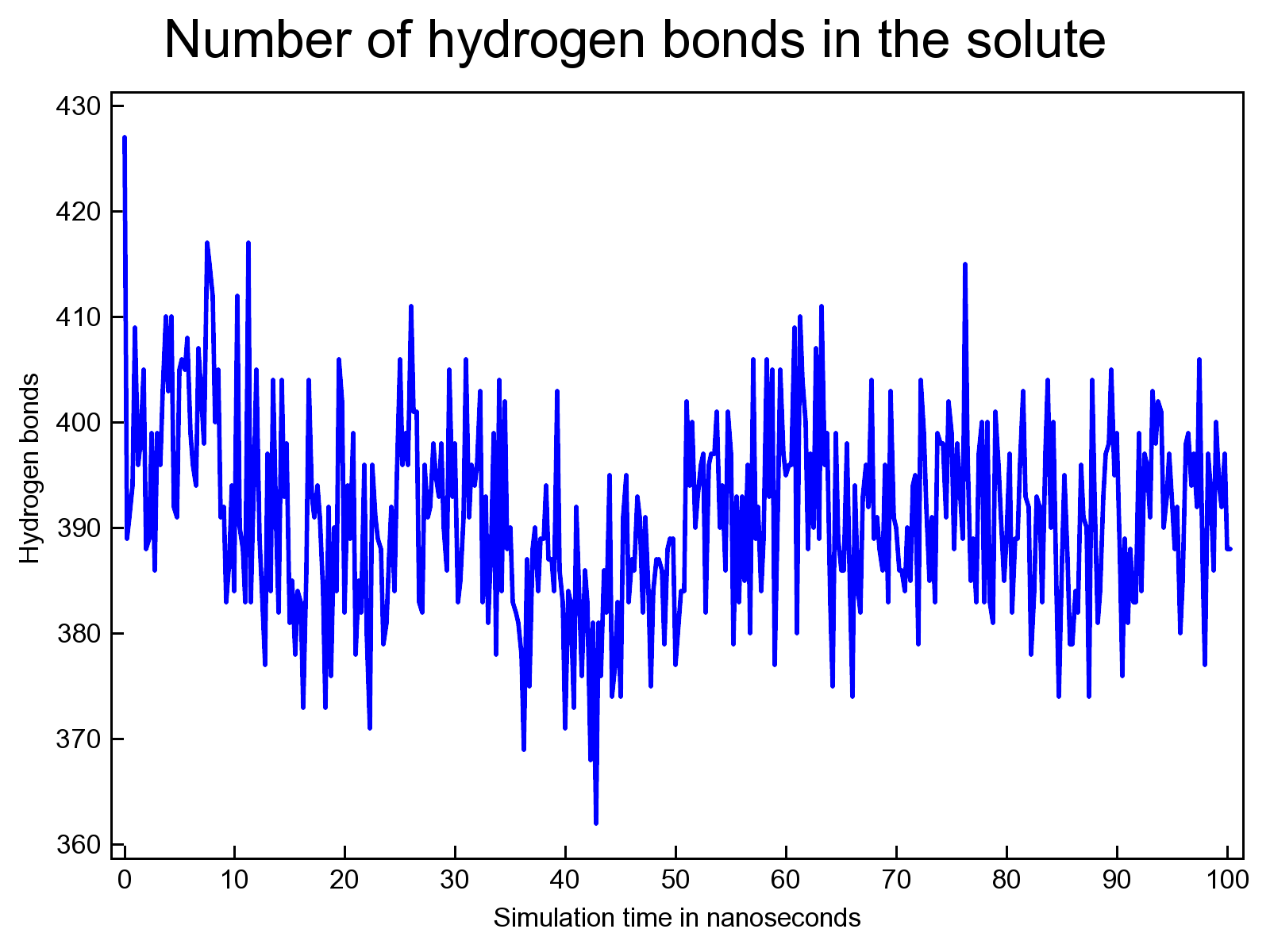

Supplement: S7 File — (ZIP) [file pone.0338211.s007.zip › S6.Molecular Dynamic Simulation/S6.Molecular Dynamic Simulation/Result napitane+roluperidone/Result 2/Complex two/Complex two_report_figure7_hires.png]

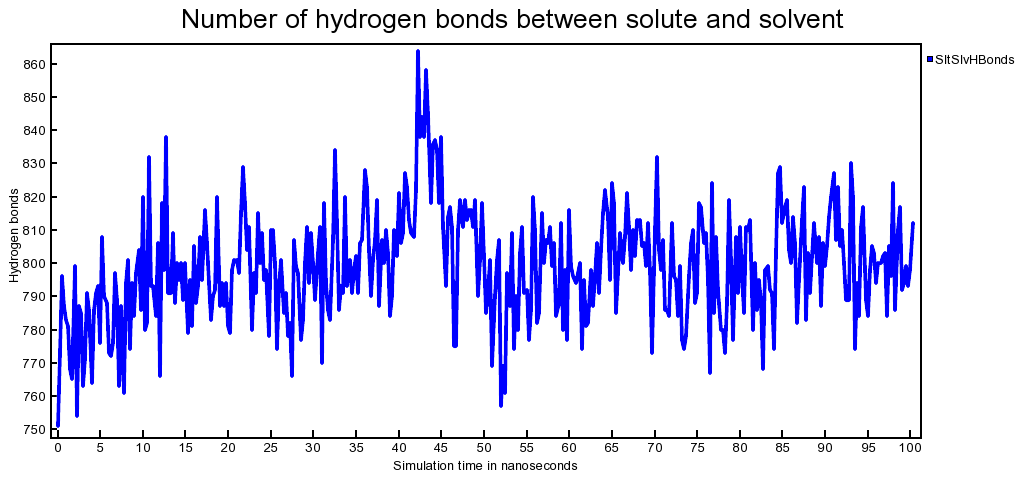

Supplement: S7 File — (ZIP) [file pone.0338211.s007.zip › S6.Molecular Dynamic Simulation/S6.Molecular Dynamic Simulation/Result napitane+roluperidone/Result 2/Complex two/Complex two_report_figure8.png]

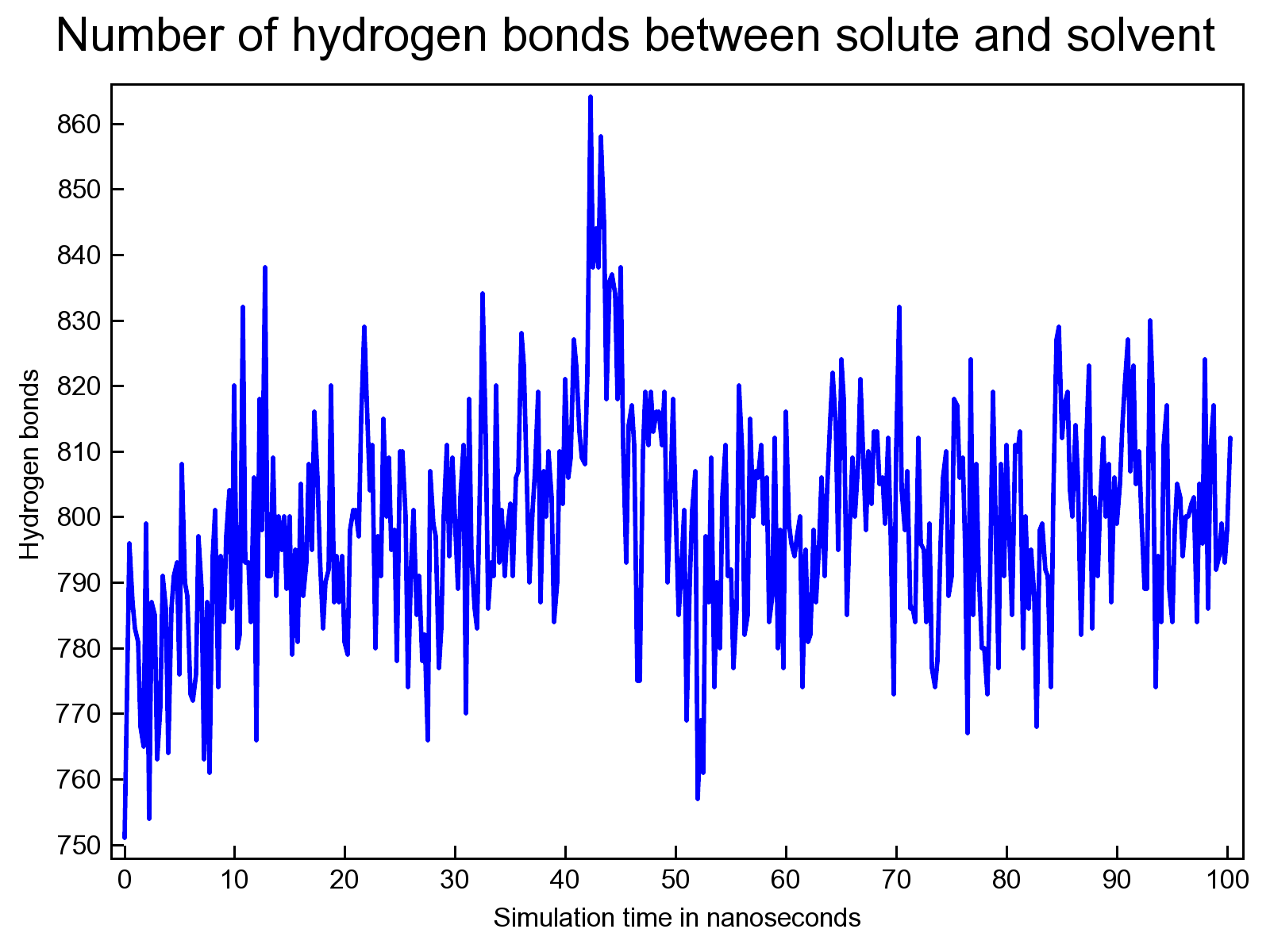

Supplement: S7 File — (ZIP) [file pone.0338211.s007.zip › S6.Molecular Dynamic Simulation/S6.Molecular Dynamic Simulation/Result napitane+roluperidone/Result 2/Complex two/Complex two_report_figure8_hires.png]

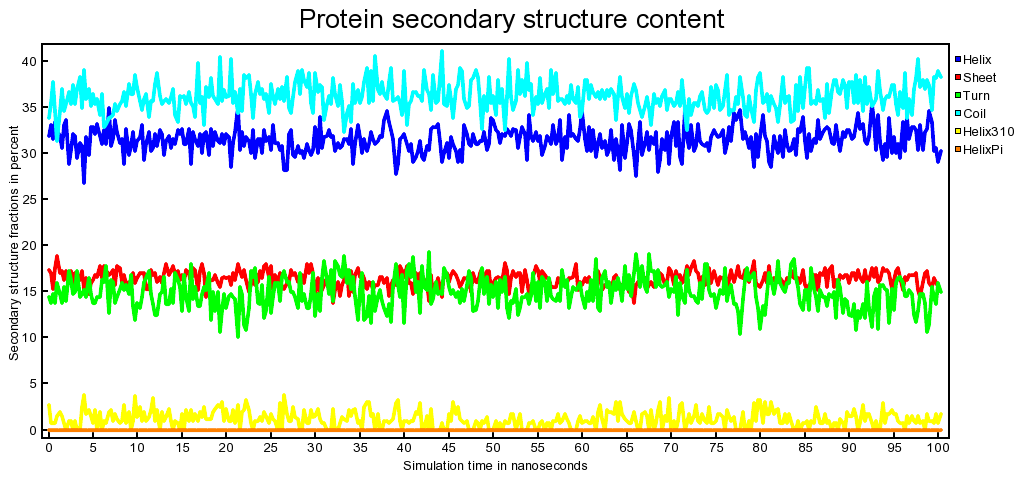

Supplement: S7 File — (ZIP) [file pone.0338211.s007.zip › S6.Molecular Dynamic Simulation/S6.Molecular Dynamic Simulation/Result napitane+roluperidone/Result 2/Complex two/Complex two_report_figure9.png]

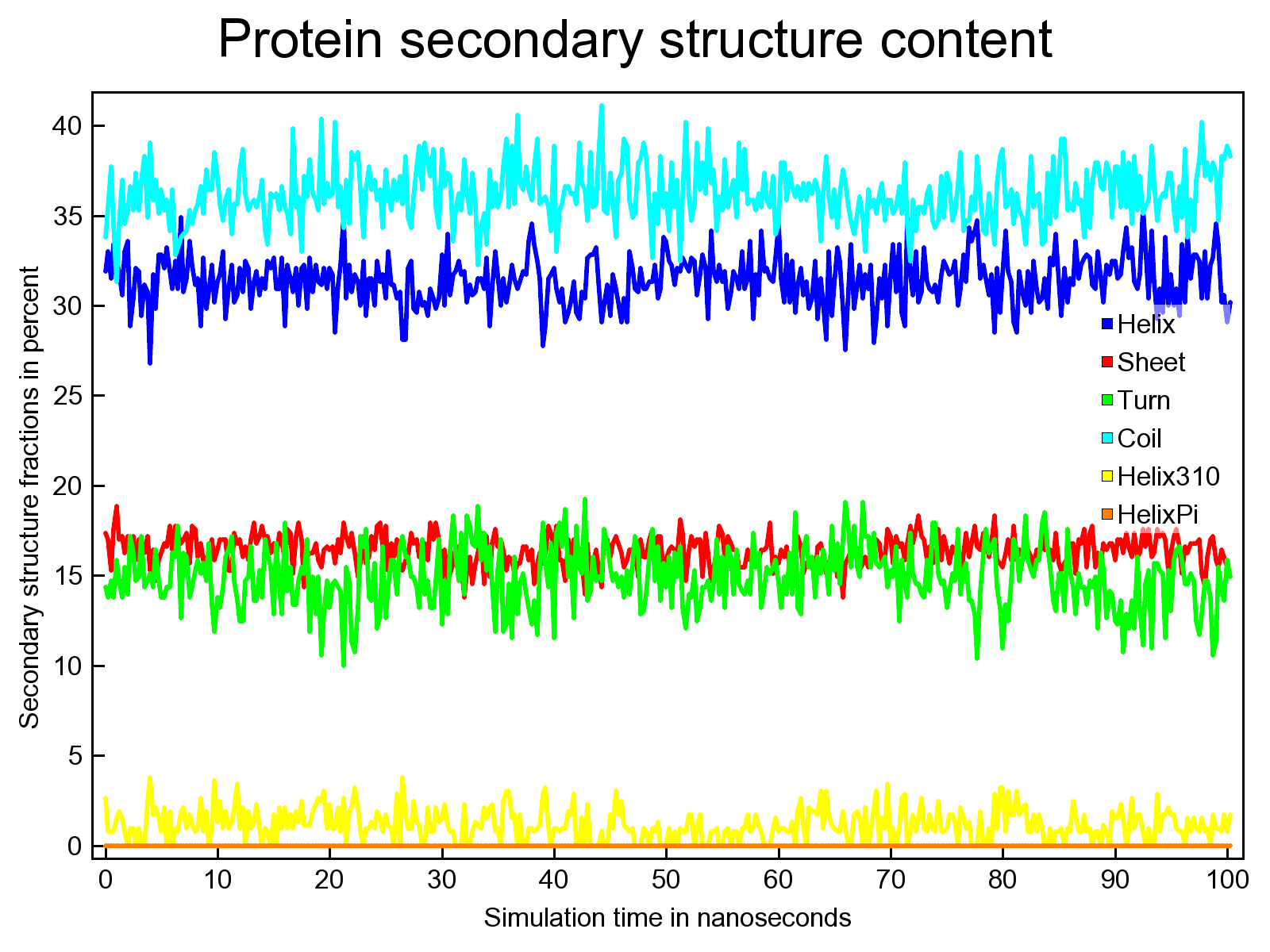

Supplement: S7 File — (ZIP) [file pone.0338211.s007.zip › S6.Molecular Dynamic Simulation/S6.Molecular Dynamic Simulation/Result napitane+roluperidone/Result 2/Complex two/Complex two_report_figure9_hires.png]
