# Supplementary material for: An evaluation of Roluperidone as a promising repurposing candidate for Alzheimer’s Disease: A Computational Investigation
Source: PLoS One. 2025 Dec 17;20(12):e0338211. doi: 10.1371/journal.pone.0338211 (PMC12711050; doi:10.1371/journal.pone.0338211)
Supplement: Attachment — Submitted filename: Response to Reviewers.docx [file pone.0338211.s009.docx]

**Dear Editor & Reviewers,**

We sincerely appreciate the time and effort you invested in reviewing our manuscript. We have carefully addressed all your instructions and comments. A detailed list of the corrections implemented is presented in the right column of the following table.

-Md. Al-Amin (Correspondent Author)

| **Serial No.** | **Editor/Reviewers' comments** | **Responses** |
| --- | --- | --- |
| 1. | Researchers performed an extensive virtual screening to reconsider different drugs likely linked to Alzheimer's disease. They refined these to nine molecules, ultimately identifying Roluperidone as a prospective candidate for Alzheimer's disease treatment. This screening technique presented literature examples of both virtual screening and the in vitro experimental efficacy of particular drugs in preventing Alzheimer's disease. At this point, more extensive screening may be performed, and research previously assessed in vitro or in vivo against AD could be examined and integrated into the article. Furthermore, necessary revisions must be implemented in accordance with the referee's recommendations. | Thank you for your insightful suggestion. We have added several citations containing previously assessed in vitro or in vivo against AD could be examined and integrated into the article.in the revised section of the manuscript |
| 2. | In the introduction, the computer-aided drug design section was briefly explained with a very good introduction; however, a figure explaining the structure and ligand-based design could be added. | Thank you for your insightful instruction. We have added a figure in that section that explains the structure and ligand-based design. |
| 3. | Abbreviations should be written according to the same standards. | Thank you. Abbreviations have been written according to the same standards. |
| 4. | Abbreviations that appear as "Ach" should be corrected to "ACh." | Thank you. "Ach" has been corrected to "ACh." |
| 5. | IC50 spellings should be written according to the rules. | Thank you. The IC_50_ spellings have been corrected. |
| 6. | Words that should be italicized should be corrected. (For example, in vitro, etc.) | Thank you. It has been corrected |
| 7. | Figure and supporting information citations must be written correctly. In many places, citations were indicated as Figure X and SX. | Thank you for the careful revision. We have corrected it. |
| 8. | It can be explained in detail which tools they use in fingerprint calculations. | Thank you. We have explained in detail which tools they use in fingerprint calculations in the revised section of the manuscript. |
| 9. | The docking method was quite simple and straightforward. However, the 25x25x25 area determined during the docking process should be explained in detail. | Thank you. We have elaborated this section in the revised manuscript. |
| 10. | Bond lengths are also given in Table 3. The study focuses on other important aspects, but bond lengths are not mentioned. Are physicochemical parameters used in the calculation, or where are they important for comparison? | Thank you. We have corrected it. We have added a figure instead of the table where bond lengths are depicted in the revised manuscript. |
| 11. | The molecular dynamics studies were well interpreted. The tools used were different, but are the underlying equations in their calculations the same? This needs to be explained. | Thank you. We have revised the molecular dynamics studies. The equations for calculating the molecular dynamic parameter are given. |
| 12. | The reasons for selecting these drugs based on the similarity graph in 3.3.1 should be detailed. | Thank you. We described it in the manuscript. |
| 13. | The basis for the machine learning elimination, which parameters stood out, and so on, should be specified. | Thank you for your careful concern for the manuscript. Initially, we performed similarity analysis before machine learning. But, later, according to the project administration instruction, we conducted machine learning before similarity analysis. The previous section of similarity analysis was not correct unconsciously. We did not eliminate the machine learning. We made a mistake. We have felt sorrow for that. However, we have corrected these in the revised section. |
